# New Oxygenated Methoxy-*p*-Cymene Derivatives from Leopard’s Bane (*Doronicum columnae* Ten., Asteraceae) Essential Oil: Synthesis Facilitating the Identification of Isomeric Minor Constituents in Complex Matrices

**DOI:** 10.3390/molecules30020302

**Published:** 2025-01-14

**Authors:** Milan Ž. Dimitrijević, Marko Z. Mladenović, Milica D. Nešić, Milan S. Dekić, Vidak N. Raičević, Niko S. Radulović

**Affiliations:** 1 Department of Chemistry, Faculty of Sciences and Mathematics, University of Niš, Višegradska 33, 18000 Niš, Serbia; milan.dimitrijevic@pmf.edu.rs (M.Ž.D.); markohem87@gmail.com (M.Z.M.); milica.stevanovic992@gmail.com (M.D.N.); 2 Department of Sciences and Mathematics, State University of Novi Pazar, Vuka Karadžića 9, 36300 Novi Pazar, Serbia; mdekic@np.ac.rs; 3 Faculty of Medicine, University of Novi Sad, Hajduk Veljkova 3, 21000 Novi Sad, Serbia; vidak.raicevic@mf.uns.ac.rs

**Keywords:** *Doronicum columnae*, volatile constituents, NMR, GC-MS, structure elucidation

## Abstract

Species of the genus *Doronicum* are known for their pharmacological properties and essential oils, the chemical composition of which remains inadequately studied. In this work, GC-MS analysis, synthesis, and spectral techniques (UV, IR, MS, and NMR) were employed to identify 83 constituents in the essential oil from *D. columnae* roots, which accounted for 98.1% of the total GC-peak area. The major components were thymyl isobutyrate (32.8%) and thymyl 2-methylbutyrate (22.8%), while the minor constituents were methoxy-*p*-cymene derivatives. Six new natural products were identified through synthesis, GC co-injection experiments and spectral characterization, including esters (isobutyrate, 2-methylbutyrate, and/or isovalerate) of 2-methoxycuminol, 6-methoxythymol, and 6-hydroxythymyl methyl ether, as well as methyl 3-methoxycuminate. Their identification was made possible by synthesis efforts, as isolating pure compounds was impracticable because of their low abundance and the overall structural similarity within the highly complex mixture that was the essential oil.

## 1. Introduction

The genus *Doronicum*, belonging to the tribe Senecioneae (family Asteraceae), comprises twenty-six species and four subspecies, distributed across Europe, North Africa and Asia, from Iran to Turkey, with some species also growing in the Himalayas [1]. Only five taxa of the *Doronicum* genus are described in the Serbian flora: *D. austriacum* Jacq., *D. austriacum* subsp. *giganteum* (Griseb.) Stoj. & Stef., *D. orientale* Hoffm., *D. columnae* Ten., and *D. hungaricum* Rchb.f. [2]. Plant species of the *Doronicum* genus are used in traditional medicine, primarily as antiparasitic, cardiotonic, analgesic, and anti-inflammatory agents [1]. Extracts from the aerial parts and roots of *Doronicum* species are rich in bioactive compounds, including pyrrolizidine alkaloids, flavonoids, terpenes, and aromatic compounds such as benzofurans and thymol derivatives [1]. Up to now, the composition of essential oils from the aerial parts of six taxa within the *Doronicum* genus has been investigated [3,4,5,6,7], including one collected in Serbia (*D. austriacum* subsp. *giganteum*) [4]. The primary constituents of these essential oils were sesquiterpene hydrocarbons, oxygenated monoterpenes (predominantly thymol derivatives), and oxygenated sesquiterpenes.

Thymol derivatives are known to occur within the Asteraceae family, particularly in the tribes Senecioneae, Eupatorieae, Inuleae, and Helenieae [8]. For instance, compounds such as thymyl and 8,9-dehydrothymyl isobutyrates have been previously identified in extracts of *Arnica amplexicaulis* [9], while 10-acetoxy-8,9-epoxythymyl isobutyrate has been reported in *A. sachalinensis* [10,11,12]. These species are morphologically closely related to the genus *Doronicum*. Additionally, other thymol derivatives, featuring groups such as acetyl, isobutyryl, angeloyl, 2-methylbutyryl, and isovaleryl, with varying degrees of oxygenation, have been found in several genera within Asteraceae, including *Schkuhria*, *Inula*, *Stoebe*, *Marshallia*, *Eriophyllum*, *Mikania*, *Ageratina*, and *Sclerolepis* [1]. Thymol and carvacrol exhibit significant antimicrobial activity by disrupting microbial membranes, effectively inhibiting the growth of bacteria, fungi, and viruses [13]. Within the genus *Doronicum*, thymol derivatives have been detected in species such as *D. austriacum* [14], *D. corsicum* [6], *D. hungaricum* [15], *D. macrophyllum* [16], and *D. pardalianches* [17]. Conversely, these compounds have not been identified in species such as *D. grandiflorum* [18], *D. hookeri* [19], *D. oblongifolium* [20], and *D. orientale* [21,22]. Notably, tremetone derivatives and pyrrolizidine alkaloids have been reported in certain species, including *D. austriacum* [14], *D. hungaricum* [15], and *D. macrophyllum* [16].

The heart-leaved leopard’s-bane (*Doronicum columnae* Ten.) is a perennial herbaceous species native to central and southern Europe, extending into the Caucasus. It thrives in shaded environments, particularly on calcareous soils, and is commonly found in deciduous, coniferous, and mixed forests, as well as in shrublands across hilly and mountainous regions [23]. Phytochemical studies on this species have been limited despite belonging to a family and genus well-known for the chemical diversity of their secondary metabolites. For instance, the pyrrolizidine alkaloid senkirkine, typically associated with the genus *Senecio* within the same family, has been identified in this species [24]. Additionally, two screening studies have investigated *D. columnae* for its antioxidant content [25] and catalase activity [26] concerning changes in these properties in plant species growing in post-fire environments. To the best of our knowledge, the composition of *D. columnae* essential oil has not been studied.

The analysis of minor constituents of essential oils is an often overlooked yet equally important aspect of understanding their comprehensive chemical and biological profiles. These minor compounds, despite their low abundances, may significantly contribute to the overall olfactory and biological effects of the oil [27]. With this study, we aimed to investigate, for the first time, the chemical composition of essential oil obtained from *D. columnae* roots. Specifically, we sought to examine previously unknown trace components, which proved to be multiply oxygenated aromatic compounds related to some of the more dominant constituents of the essential oil. Research addressing isomeric or otherwise closely related compounds that cannot be adequately distinguished by mass spectra or retention indices alone faces significant analytical challenges, in part because of the possibility that some of the isomers may represent novel chemical entities. To address this, we opted for a multifaceted approach, leveraging structure-retention index correlations and reasonable biosynthetic insights, and finally utilizing authentic samples prepared by chemical synthesis, which led us to the identification of a number of methoxy-*p*-cymene derivatives, six of which constituted new natural products.

## 2. Results and Discussion

### Composition of D. columnae Essential Oil

**Figure 1:**
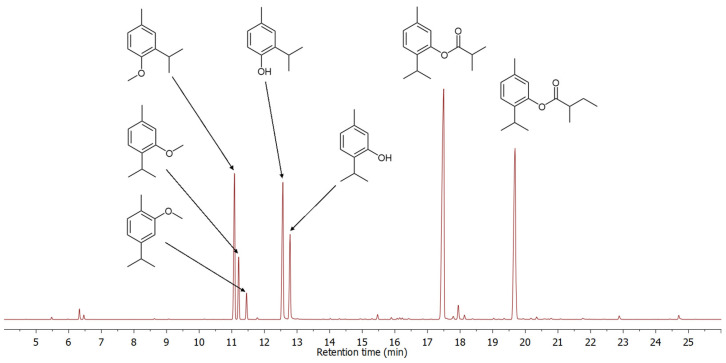
A typical TIC (total ion current) chromatogram of the essential oil of *D*. *columnae* roots.

The dry roots of *D. columnae* yielded a yellowish essential oil (0.56%, *w*/*w*). A combination of GC-MS, UV, IR, and NMR, along with chromatographic separation and synthetic work, led to the identification of 83 constituents (Table 1) of the *D. columnae* essential oil, amounting to 98.1% of the total detected GC-peak areas (Figure 1). Oxygenated monoterpenoids and sesquiterpene hydrocarbons are the dominant compound classes, comprising 95.0% and 2.2%, respectively. The major constituents were thymyl isobutyrate and thymyl 2-methylbutyrate, constituting 32.8% and 22.8% of the essential oil, respectively.

**Figure 2:**
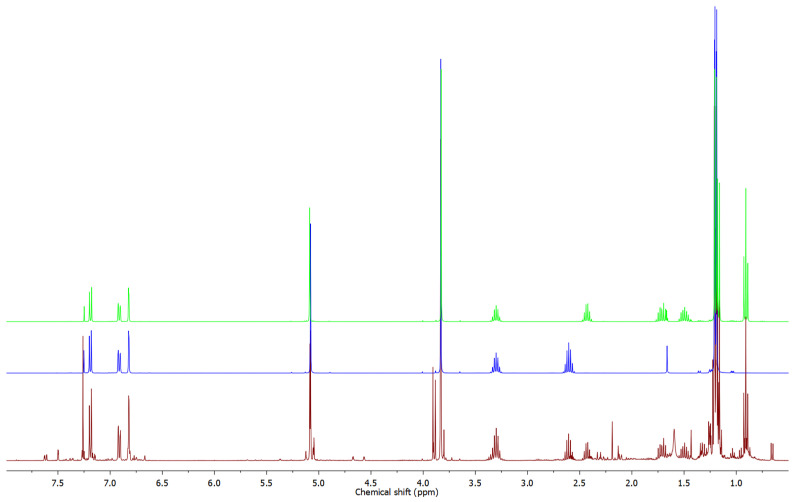
^1^H NMR spectra of the tenth chromatographic fraction (eluted with 5% ether in hexane), shown in brown, of a standard of 3-methoxycuminyl isobutyrate, shown in blue, and of 3-methoxycuminyl 2-methylbutyrate, shown in light green.

While most of the identified compounds are common essential oil constituents previously detected in other *Doronicum* species (e.g., *D. austriacum* [4] and *D. corsicum* [6]), one series of constituents, although present in trace amounts, caught our attention. A total of 14 compounds, observed within the retention index (RI) range of 1435 to 1845 (Table 1), were presumed to be thymol-like compounds based on their fragmentation patterns visible in the mass spectra. To isolate these trace constituents, chromatographic separation of the essential oil was undertaken. A dry-column vacuum chromatography of the essential oil on silica gel, using 5% (*v*/*v*) diethyl ether in hexane as the eluent, resulted in a fraction enriched with these trace constituents, which proved hardly detectable in the initial GC-MS analysis of the unfractionated essential oil. Direct NMR analysis of the total fraction (Figure 2) strongly suggested that major fraction constituents were oxygenated derivates of methoxy-*p*-cymene.

A detailed GC-MS analysis of the fraction revealed the presence of twelve compounds, including five with a molecular ion at *m*/*z* 250 and seven with a molecular ion at *m*/*z* 264. Based on mass spectral data, due to their similar fragmentation patterns—dominant fragmentation ions at *m*/*z* 163 and 179 or 180, *m*/*z* 165 and 180, and *m*/*z* 147 and 162—it was concluded that the detected compounds belong to one of five different groups of isomers (Figure S1). The presence of distinct pairs of intense ions at *m*/*z* 43 and 71, characteristic of (iso)butyrates (C_4_H_7_O⁺), and peaks at *m*/*z* 57 and 85, indicative of isomeric valerates (C_5_H_9_O⁺), provided crucial information supporting the tentative identification. A comparison of the retention indices (RI) and mass spectrometry (MS) data for one homologous series of compounds detected at RI 1723, 1808, and 1817, with compounds recently identified as constituents of *Pulicaria dysenterica* essential oil [29] led to the identification of the major fraction constituents as 3-methoxycuminyl isobutyrate, 3-methoxycuminyl 2-methylbutyrate, and 3-methoxycuminyl isovalerate. This tentative identification was further confirmed by a GC co-injection experiment of the fraction and pure synthesized compounds from our in-house natural product library, as well as by comparison of their NMR data [29] with that of the essential oil fraction (Figure 2 and Figures S2–S16, and Tables S1–S3).

**Figure 3:**
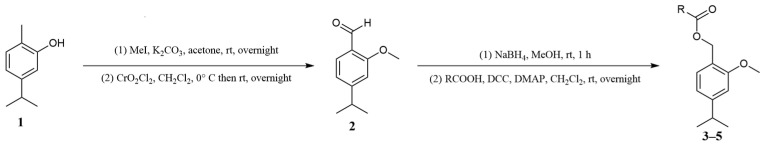
Synthesis of 2-methoxycuminyl isobutyrate (**3**), 2-methylbutyrate (**4**), and isovalerate (**5**) (R = isopropyl, *sec*-butyl, or isobutyl).

Additionally, the ^1^H NMR spectrum (Figure 2) revealed signals corresponding to CH_2_ groups with chemical shift and multiplicity closely resembling those of the benzylic oxymethylene group in 3-methoxycuminyl esters, observed as a singlet at 5.08 ppm. This suggested that one homologous series of the detected constituents (at RI 1750, 1835, and 1845) could be esters of a 3-methoxycuminol regioisomer, specifically the esters of 2-methoxycuminol. It is logical to expect that an essential oil that contains biosynthetically related compounds (e.g., thymol and carvacrol along with their ethers and esters) could contain, alongside 3-methoxycuminyl esters, the regioisomeric 2-methoxycuminyl esters as well. To confirm the stated hypothesis, we synthesized these compounds using a two-step approach: the synthesis of 2-methoxycuminol and the subsequent preparation of its esters (2-methoxycuminyl isobutyrate, 2-methylbutyrate, and isovalerate). The synthesis of 2-methoxycuminol involved the methylation of carvacrol (**1**) with methyl iodide, followed by benzylic oxidation of the resulting carvacryl methyl ether with chromyl chloride (Figure 3). The obtained reaction mixture predominantly contained thymoquinone in addition to a certain amount of 2-methoxycuminaldehyde (**2**). The confirmation of the structure of the synthesized 2-methoxycuminaldehyde (**2**) was based on HMBC interactions of the C-6 proton at 7.76 ppm, with a carbon atom at 189.67 ppm, corresponding to the aldehyde group (Figure S17 and Table S4). Additionally, NOESY interactions between the C-6 proton and the aldehyde proton at 10.40 ppm, as well as interactions of this proton with the protons from the methoxy group, indicate their spatial proximity. The obtained 2-methoxycuminaldehyde (**2**) was then reduced to 2-methoxycuminol (Figure S18 and Table S5) and subsequently esterified with isobutyric (**3**), 2-methylbutyric (**4**), and isovaleric acid (**5**) through the Steglich esterification method. The chemical shift of the protons from the methylene group in the synthesized esters (Figures S19–S36 and Tables S6–S8) is identical to the shift of the methylene group in the unknown component of the fraction (*δ*_H_ = 5.12 ppm). Initial comparisons of ^1^H NMR spectra of the synthesized standards to that of the chromatographic fraction showed satisfactory congruence; however, GC co-injection experiments definitively confirmed that all synthesized 2-methoxycuminyl esters were present in the essential oil of *D. columnae*, representing both new natural products and new chemical entities.

**Figure 4:**
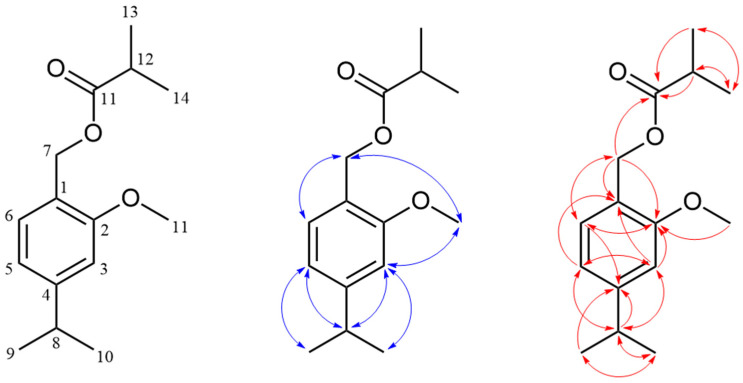
From **left** to **right**: the structure of 2-methoxycuminyl isobutyrate (**3**) with the carbon atom numbering scheme, important NOESY interactions (marked with blue arrows), and important HMBC interactions (marked with red arrows).

The assignment of signals is discussed in detail for one of the newly identified natural products, 2-methoxycuminyl isobutyrate (**3**) (Figure 4), while the same approach was used for structure elucidation and signal assignment of all other synthesized compounds in an analogous fashion. The ^1^H and ^13^C NMR spectra of 2-methoxycuminyl isobutyrate (**3**) showed the expected number of signals (Figures S21–S23). A doublet at 7.23 ppm (*J* = 7.7 Hz) was assigned to the proton on the aromatic ring at position 6. The HSQC spectrum enabled the assignment of the ^13^C NMR signal for the carbon atom (C-6) at 129.65 ppm. ^1^H NMR spectra with homonuclear decoupling confirmed the interaction of this proton with a proton at position 5 (attached directly to the carbon atom at 118.35 ppm), which appeared as a doublet of doublets (*J* = 7.7 and 1.6 Hz) at 6.82 ppm. The presence of an additional coupling constant (1.6 Hz) suggested a further interaction with a proton at position 3 (6.75 ppm), with its corresponding ^13^C NMR signal at 109.02 ppm. A singlet at 5.12 ppm was assigned to the methylene group at position 7, with the HSQC spectrum linking it to the ^13^C NMR signal at 61.76 ppm, that of C-7. The HMBC spectrum displayed correlations between C-7 protons and four ^13^C NMR signals at 122.06, 129.65, 157.65, and 177.33 ppm, assigned to C-1, C-6, C-5, and C-11, respectively (Figure S24 and Table S6). Additionally, a doublet at 1.26 ppm (*J* = 6.9 Hz) corresponded to the two methyl groups in the isopropyl fragment (protons of C-9 and C-10), which were coupled to a one-proton septuplet at 2.90 ppm. The HSQC spectrum allowed for the assignment of ^13^C resonances for the carbon atoms in this structural segment (C-8 at 34.50 ppm and C-9 and C-10 at 24.13 ppm). In the case of the methoxy group, the protons (H-15) appeared as a singlet at 3.84 ppm, corresponding (according to the HSQC spectrum) to the carbon atom at 55.49 ppm (C-15). Only one correlation of the protons from the methoxy group was visible in the HMBC spectrum, and that was with the ^13^C NMR signal of the C-2 carbon atom. The assignment of the acidic moiety in 2-methoxycuminyl isobutyrate (**3**), along with the 2-methylbutyrate and isovalerate derivatives, was based on prior analyses performed by our group [29].

The three remaining groups of detected, yet unidentified, constituents were found to possess the same molecular ions (*m*/*z* 250 and 264) and exhibit similar fragmentation patterns in the mass spectra, as well as close RI values to those of the identified 2-methoxycuminyl and 3-methoxycuminyl esters. These similarities suggested that these constituents might be their regioisomers. As expected, spectral and GC retention data for such regioisomeric esters is quite scarce in the literature. An additional challenge in the unambiguous GC–MS identification of such regioisomeric compounds stems from the fact that they display very similar MS fragmentation patterns, and some have nearly identical RI values. As a result, final structural confirmation cannot rely solely on MS and/or RI criteria. In such cases, even a small discrepancy in RI values from the literature, likely because some researchers fall into the trap of seemingly straightforward identifications, adds even more confusion. For example, for the 6-methoxythymyl isobutyrate (**12**), there are two RI values—1659 [30] and 1671 [31] on the non-polar CP-Sil 5 and CP-Sil 5 CB columns, respectively. The only worthwhile approach was to synthesize the possible isomeric esters and confirm the identity of the naturally occurring isomers through GC co-chromatography of mixtures containing *D. columnae* essential oil and individual authentic standards obtained by synthesis.

**Figure 5:**
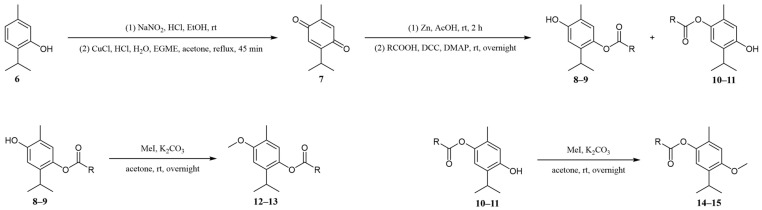
Synthesis of 6-methoxythymyl (**12** and **13**) and/or 6-hydroxythymyl methyl ether (**14** and **15**) esters (R = isopropyl or *sec*-butyl).

The presence of one isomeric dimethoxy-*p*-cymene, specifically 2,5-dimethoxy-*p*-cymene, in the essential oil sample, also indicated that some of these isomeric series could be the esters of the isobutyric or 2-methylbutyric acid with 6-methoxythymol and/or 6-hydroxythymyl methyl ether. To confirm our assumption, we synthesized the mentioned esters starting from thymol, following the reaction scheme presented in Figure 5. Thymol (**6**) was first subjected to nitrosation with a mixture of NaNO_2_/HCl to yield 6-nitrosothymol, which was subsequently hydrolyzed to thymoquinone (**7**). The reduction of thymoquinone with zinc powder in acetic acid afforded thymohydroquinone. Esterification of thymohydroquinone was carried out using isobutyric acid and 2-methylbutyric acid via the Steglich esterification method. The reaction produced a mixture of monoesters (**8**–**11**), which were then successfully separated using column chromatography. The structure of the synthesized monoesters could be easily determined based on NOESY interactions in the NMR spectrum (Figures S37–S40 and Tables S9–S12). For example, the signal of the C-3 proton at 6.72 ppm exhibited a NOESY interaction with the proton from the OH group (at 4.82 ppm). This OH proton also displayed an NOE interaction with protons from a methyl group at 2.17 ppm. Similarly, the structure of the second monoester was determined as follows: the C-6 proton at 6.44 ppm exhibited an NOE interaction with the OH proton at 5.50 ppm. This OH proton was found to further interact with the protons of the methyl groups from the isopropyl moiety (signal at 1.19 ppm). These data suggest that, in the first case, the OH group is in the *ortho* position relative to the methyl group (6-hydroxythymyl esters (**8** and **9**), while in the second case, the OH group is *ortho* to the isopropyl group (e.g., 6-isobutyryloxythymol (**10**)). Additional confirmation of these structures comes from the differing chemical shifts of the methyl groups, depending on the identity of the *ortho* substituent. When the ester group is in the *ortho* position, the methyl groups shift upfield compared with when the OH group occupies the *ortho* position, likely due to the anisotropic shielding effect of the carbonyl group. Finally, obtained monoesters were additionally methylated using methyl iodide. All prepared compounds were structurally characterized by MS, IR, UV-Vis, and NMR (Figures S37–S64, Tables S9–S16). Among the synthesized esters, 6-methoxythymyl 2-methylbutyrate (**13**) and 6-(2-methylbutyryloxy)thymyl methyl ether (**15**) represents new compounds, whereas GC co-injection confirmed that the *D. columnae* essential oil constituents were isobutyrates and 2-methylbutyrates (new natural products) of 6-methoxythymol (**12** and **13**) and 6-hydroxythymyl methyl ether (**14** and **15**).

**Figure 6:**
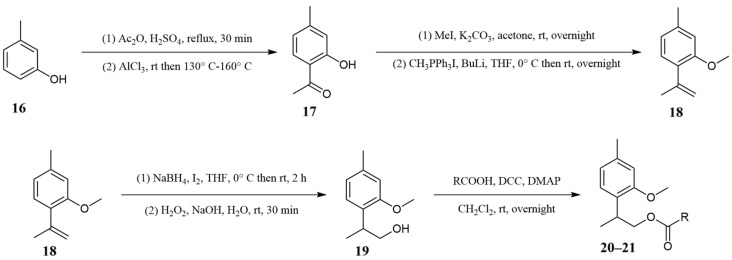
Synthesis of 9-hydroxythymyl methyl ether esters (**20** and **21**) (R = isopropyl or *sec*-butyl).

Oxygenated derivatives of methoxy-*p*-cymene, particularly esters of 9-hydroxythymyl methyl ether (**20** and **21**), have been previously identified in the essential oil of *Arnica montana* L. [30]. Noted similarities of the fragmentation pattern in the mass spectra of 9-hydroxythymyl methyl ether esters from the literature with those of the constituents present in our samples suggested that the detected remaining series represents esters of 9-hydroxythymyl methyl ether; however, since the literature retention indices did not match our retention indices and the fact that there is theoretically another possible isomer with a hydroxy group in position 8, we decided to perform their synthesis to confirm their presence in the essential oil. The synthesis of these compounds was based firstly on the synthesis of 9-hydroxythymyl methyl ether (**19**, Figure S71 and Table S18) and the subsequent preparation of esters (9-isobutyryloxythymyl and 9-(2-methylbutyryloxy)thymyl methyl ethers, **20** and **21,** Figure 6). The synthesis of 9-hydroxythymyl methyl ether involved the esterification of *m*-cresol (**16**) achieved with acetic anhydride, followed by a Fries rearrangement of the acetyl group using anhydrous aluminum chloride. The resulting 3-hydroxy-4-methylacetophenone (**17**) was methylated with methyl iodide. A Wittig reaction between 4-methyl-3-methoxyacetophenone (**17**) and an in situ generated ylide yielded 8,9-dehydrothymyl methyl ether (**18**) (Figures S65–S70 and Table S17). This compound was then subjected to hydroboration-oxidation to form 9-hydroxythymyl methyl ether (**19**), followed by esterification with isobutyric and 2-methylbutyric acid (Figures S72–S83 and Tables S19 and S20). Subsequent GC co-injection experiments confirmed the presence of 9-isobutyryloxythymyl methyl ether (**20**) and 9-(2-methylbutyryloxy)thymyl methyl ether (**21**) in the *D. columnae* essential oil. Since 9-hydroxythymyl methyl ether (**19**) is chiral, its esterification with commercial racemic 2-methylbutyric acid yielded a diastereomeric mixture of 9-(2-methylbutyryloxy)thymyl methyl ethers (**21**). This mixture could not be observed as two separate peaks in the TIC chromatogram under our operating conditions; however, the presence of such a mixture was confirmed through NMR.

**Figure 7:**
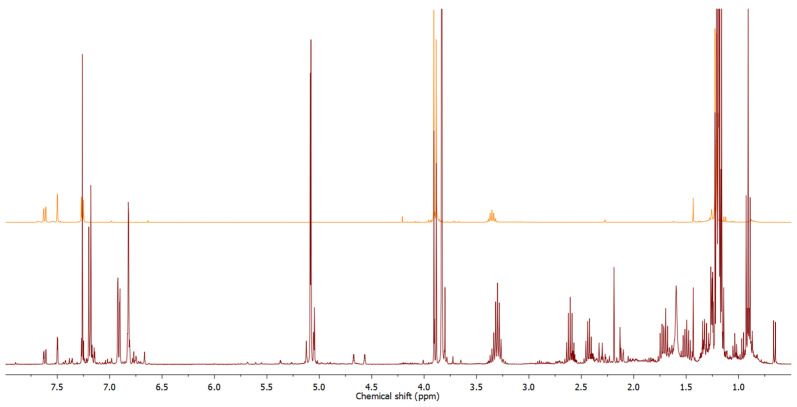
^1^H NMR spectra of the tenth chromatographic fraction (eluted with 5% ether in hexane), shown in brown, and methyl 4-isopropyl-3-methoxybenzoate (*syn*. methyl 3-methoxycuminate (**23**)), shown in orange.

**Figure 8:**
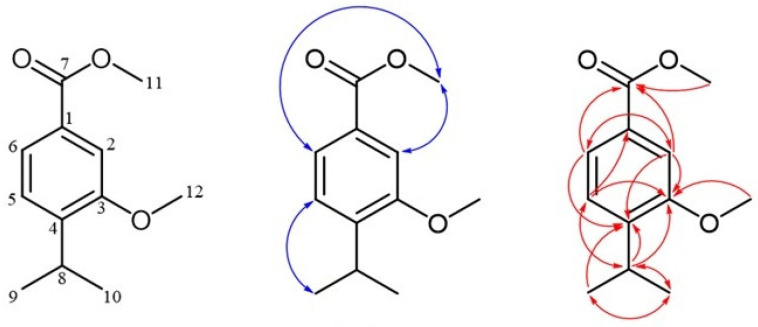
From **left** to **right**: the structure of methyl 3-methoxycuminate (**23**) with the carbon atom numbering scheme, important NOESY interactions (marked with blue arrows), and important HMBC interactions (marked with red arrows).

Apart from regioisomeric oxygenated derivatives of methoxy-*p*-cymene, certain signals in the ^1^H NMR spectrum of the fraction (Figure 7) suggested the presence of an additional, possibly oxygenated derivative of methoxy-*p*-cymene among the fraction constituents. Proton signals at 7.62 ppm (*J* = 7.9 and 1.6 Hz), 7.50 ppm (*J* = 1.6 Hz), and 7.26 ppm (*J* = 7.9 Hz) were assigned to protons H-6, H-5, and H-2, respectively, based on ^1^H-^1^H-COSY and HMBC interactions. The HMBC spectrum displayed correlations between H-2 and H-6 protons with a ^13^C NMR signal at 167.38, assigned as C-7, indicating the presence of a carbonyl group directly attached to the aromatic ring. Protons of one of the methoxy groups appeared as a singlet at 3.91 ppm (H-11) and were directly connected (according to the HSQC spectrum) to the carbon atom at 52.19 ppm (C-11). In addition, correlations with C-7 were displayed and were therefore identified as methyl ester protons. Additionally, a doublet at 1.22 ppm (*J* = 6.9 Hz) corresponded to the two methyl groups in the isopropyl fragment (protons of C-9 and C-10), which were coupled to a one-proton septuplet at 3.35 ppm. The HMBC spectrum displayed correlations between the H-8 proton and ^13^C NMR signals originating from five carbon atoms at 22.53 (two carbons), 126.02, 142.79, and 156.76 ppm, assigned to C-9 and C-10, C-5, C-4, and C-3, respectively. In the case of the remaining methoxy group, the protons (H-12) appeared as a singlet at 3.88 ppm that was directly connected (according to the HSQC spectrum) to the carbon atom at 55.64 ppm (C-12). The HMBC spectrum displayed only one correlation of these protons, and that was with the signal of the C-3 carbon atom. Based on NMR analysis, this compound was identified as methyl 4-isopropyl-3-methoxybenzoate (*syn*. methyl 3-methoxycuminate (**23**), Figure 8).

**Figure 9:**
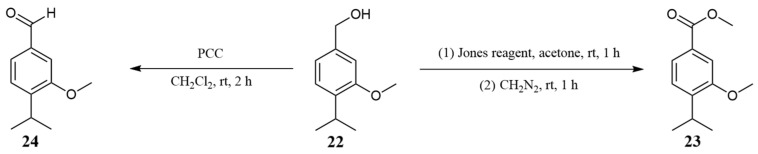
Synthesis of methyl 3-methoxycuminate (**23**) and 3-methoxycuminaldehyde (**24**).

To confirm our assumption, we synthesized the mentioned ester starting from 3-methoxycuminol (**22**) (Figure 9). The synthesis involved oxidizing 3-methoxycuminol (**22**) to the corresponding acid (Figure S84 and Table S21), followed by methylation of the resulting acid to produce the methyl ester (**23**). The synthesized methyl 4-isopropyl-3-methoxybenzoate (**23**) was fully spectrally characterized using MS, IR, UV-Vis, and NMR (Figues S85–S90 and Table S22). A comparison of NMR data from the tenth fraction and the pure methyl ester, together with a GC co-injection experiment, confirmed that the synthesized methyl ester (**23**) was a constituent of the essential oil and represents a new natural product.

In the NMR spectrum of the tenth fraction, an additional signal, based on its chemical shift, was attributable to a proton from an aldehyde group, suggesting the possible presence of an aldehyde related to methyl 3-methoxycuminate (**23**). Consequently, the aldehyde was synthesized starting from 3-methoxycuminol (**22**) (Figure 9 and Figures S91–S96, and Table S23). Although the synthesized 3-methoxycuminaldehyde (**24**) is a known compound, a GC co-injection confirmed that this aldehyde is a constituent of the essential oil of *D. columnae*.

Based on the results presented above, the isolated essential oil of *D. columnae* shows potential applications in pharmaceutical, cosmetic, and agricultural industries because of its unique chemical profile, particularly the high concentration of thymyl derivatives. Given the antimicrobial and anti-inflammatory properties previously associated with thymol derivatives, the essential oil could be explored for use in the development of natural preservatives or therapeutic agents [13,29]. Furthermore, its complex aromatic composition suggests potential utility in fragrance formulations [27] or as a bioactive agent in pest management strategies.

## 3. Materials and Methods

### 3.1. General Experimental Procedures

All used chemicals and solvents were obtained from commercial sources (Sigma-Aldrich, St. Louis, MO, USA; Merck, Darmstadt, Germany; Fisher Scientific, Waltham, MA, USA) and used as received, except for the solvents, which were distilled and dried before use. Silica gel 60, particle size distribution 40–63 mm (Acros Organics, Geel, Belgium), was used for dry-column vacuum chromatography, whereas precoated Al silica gel plates (Merck, Darmstadt, Germany), Kieselgel 60 F_254_, 0.2 mm) were used for analytical TLC analyses. The TLC plates were visualized by spraying with 50% (*v*/*v*) aq. H_2_SO_4_ followed by brief heating. Elemental analyses (microanalysis of carbon, hydrogen, and oxygen) were carried out with a Carlo Erba Elemental Analyzer model 1106 (Carlo Erba Strumentazione, Milan, Italy). ATR-IR measurements (attenuated total reflectance) were carried out using a Thermo Nicolet model 6700 FTIR instrument (Waltham, MA, USA). UV spectra (in acetonitrile) were measured using a UV-1800 PC Shimadzu spectrophotometer (Tokyo, Japan). The melting points were determined on a Büchi Melting Point B-540 apparatus (Büchi Labortechnik AG, Flawil, Switzerland) and were uncorrected.

### 3.2. Gas Chromatography-Mass Spectrometry (GC-MS) Analyses

GC-MS analyses (3 repetitions) were carried out using a Hewlett-Packard 6890 N gas chromatograph equipped with a fused silica capillary column DB-5MS (5% polydiphenyl-siloxane and 95% polydimethylsiloxane, 30 m × 0.25 mm, film thickness 0.25 µm, Agilent Technologies, Palo Alto, CA, USA) and coupled with a 5975B mass selective detector from the same company. The injector and interface were operated at 250 and 300 °C, respectively. The oven temperature was raised from 70 to 290 °C at a heating rate of 5 °C/min, and the program ended with an isothermal period of 10 min. Helium was used as a carrier gas at 1.0 mL/min. The samples, 1.0 µL of essential oil/essential oil fraction/pure compound solutions in diethyl ether (ca. 1 mg of an essential oil sample per 1.0 mL of solvent), were injected in a pulsed split mode (the flow was 1.5 mL/min for the first 0.5 min and then set to 1.0 mL/min throughout the remainder of the analysis; split ratio 40:1). MS conditions were as follows: ionization voltage 70 eV, acquisition mass range *m*/*z* 35–650, scan time 0.32 s. The constituents were identified by comparison of their linear retention indices (relative to C_9_–C_19_ *n*-alkanes on a DB-5MS column) with the literature values and their mass spectra with those of authentic standards, as well as those from Wiley 11, NIST17, MassFinder 2.3, and a homemade MS library, with the spectra corresponding to pure substances and components of known oils, and wherever possible, by co-injection with an authentic sample [32]. The GC-MS analyses were also performed on an Agilent Technologies 7890B gas chromatograph equipped with an HP-Innowax capillary column (polyethylene glycol stationary phase, 30 m × 0.25 mm, film thickness 0.25 μm, Agilent Technologies, Santa Clara, CA, USA) and coupled with a 240-MS ion trap detector (Agilent Technologies, Santa Clara, CA, USA). The injector and interface were operated at 220 and 230 °C, respectively. The oven temperature was raised from 40 to 220 °C at a heating rate of 7 °C/min and then isothermally held for 10 min. Helium was used as a carrier gas at 1.0 mL/min. The samples were injected with a split ratio of 40:1. The MS conditions were as follows: trap, ion source, and manifold temperatures were 100, 180, and 50 °C, respectively, ionization energy 70 eV, acquisition mass range 35–500 amu, and scan data rate of 2.08 Hz. The linear retention indices on an HP-Innowax column were determined relative to the retention times of C_10_–C_25_ *n*-alkanes.

### 3.3. NMR Measurements

^1^H and ^13^C NMR spectra were recorded on a Bruker Avance III 400 MHz NMR spectrometer (Fällanden, Switzerland; ^1^H at 400 MHz, ^13^C at 100.6 MHz), equipped with a 5 mm dual ^13^C/^1^H probe head at 20 °C. All the NMR spectra were recorded in CDCl_3_ (Sigma-Aldrich, St. Louis, MO, USA) with tetramethylsilane (TMS) as an internal standard. Chemical shifts (δ) are reported in ppm and referenced to tetramethylsilane (δ_H_ = 0.00 ppm), or the (residual) solvent signal (CHCl_3_), and ^13^CDCl_3_, in ^1^H NMR and ^13^C NMR and heteronuclear 2D spectra, respectively. Scalar couplings are reported in Hertz (Hz). The acquired NMR experiments, both 1D and 2D, were recorded using standard Bruker built-in pulse sequences. ^1^H NMR full spin analysis of all products was performed by manually adjusting δ_H_ and *J* values to fit the experimentally available values and further optimized using MestReNova 11.0.3 software (Tools/Spin Simulation) [33]. This procedure led to a systematic refinement of all calculated NMR parameters until the simulation outcome was in excellent agreement (NRMSD < 0.05%) with these experimental data from the synthesized compounds. The values of chemical shifts and coupling constants were determined by a simulation of the ^1^H NMR spectrum (manual iterative full spin analysis).

### 3.4. Plant Material

The roots of *Doronicum columnae* were collected during the flowering stage in May 2021 in the Šar Mountains, Republic of Serbia, from a wild population. Voucher specimens were deposited in the Herbarium of the Faculty of Sciences and Mathematics, University of Niš, Serbia, under the acquisition number HMN-15655. The identity of the plant material was confirmed by a trained botanist, the herbarium custodian. The underground parts were separated, washed from the soil, dried, and weighed. The mass of the roots was 250 g.

### 3.5. Hydrodistillation

The dry underground parts (250 g) of *D. columnae* were subjected to hydrodistillation for 2.5 h using the original Clevenger-type apparatus and yielded 0.56% (*w*/*w*) of essential oil. The obtained essential oil was recovered with diethyl ether and dried with anhydrous magnesium sulfate. The solvent was evaporated under a gentle stream of nitrogen at room temperature, and the essential oil was then immediately analyzed by GC–MS.

### 3.6. Chromatography of the Essential Oil

The essential oil sample (1.35 g) was fractionated using gradient dry-column vacuum chromatography (starting from pure *n*-hexane and ending with a 4:1 mixture of *n*-hexane and Et_2_O, *v*/*v*). The fractions were pooled based on GC-MS and TLC analysis, and sixteen different fractions were obtained, with masses of: 5.0 mg (fr. 1), 3.4 mg (fr. 2), 4.2 mg (fr. 3), 2.6 mg (fr. 4), 97 mg (fr. 5), 54 mg (fr. 6), 1.01 g (fr. 7), 82 mg (fr. 8), 25 mg (fr. 9), 10 mg (fr. 10), 3.1 mg (fr. 11), 10 mg (fr. 12), 8.3 mg (fr. 13), 7 mg (fr. 14), 4.9 mg (fr. 15), and 15.6 mg (fr. 16).

### 3.7. Synthesis of 2-Methoxycuminol ((4-Isopropyl-2-methoxyphenyl)methanol)

#### 3.7.1. O-Methylation of Carvacrol (**1**)

Carvacrol (**1**) (3.0 g, 0.02 mol) was dissolved in 50 mL of acetone, and 6.0 g (0.04 mol) of anhydrous potassium carbonate was added to the solution. Then, 3 mL of methyl iodide was added to the suspension, and the reaction mixture was stirred at room temperature for 24 h [29]. Acetone was removed under reduced pressure, and water was added to the residue. The mixture was extracted three times with diethyl ether; the combined organic layers were washed with an aqueous solution of sodium hydroxide and dried over anhydrous MgSO_4_. The solvent was removed under reduced pressure, and carvacryl methyl ether was obtained as a colorless liquid (3.0 g, 91%). The purity of the product was checked by TLC and GC–MS.

Carvacryl methyl ether: retention index (RI) = 1246 (DB-5MS column), MS (EI)*:* in complete agreement with the values published by Schreiner et al., 2019 [34].

#### 3.7.2. Benzylic Oxidation of Carvacryl Methyl Ether

A solution of carvacryl methyl ether (1.0 g, 6 mmol) in 20 mL of CH_2_Cl_2_ was cooled to 0 °C and to it was slowly added a solution of chromyl chloride (1.9 g, 12 mmol) in 20 mL of CH_2_Cl_2_ [35]. When all of the chromyl chloride was added, the mixture was stirred overnight at room temperature. The reaction mixture was quenched by pouring it into a stirred mixture of ice and a saturated aqueous solution of sodium sulfite. The mixture was extracted three times with CH_2_Cl_2_, and the combined organic layers were washed with an aqueous sodium chloride solution and dried over anhydrous MgSO_4_. The solvent was removed under reduced pressure, and the resulting residue was purified by column chromatography on silica gel using an *n*-hexane/Et_2_O mixture (19:1, *v*/*v*) as the eluent. 4-Isopropyl-2-methoxybenzaldehyde (**2**) was obtained as a yellowish liquid (178 mg, 16%). The purity of the product was checked by TLC and GC–MS.

4-Isopropyl-2-methoxybenzaldehyde (**2**): retention index (RI) = 1505 (DB-5MS column); UV (CH_3_CN) λ_max_(log ε) 317 (3.82), 259 (4.24), 217 (4.39), 203 (4.39) nm; FTIR (neat; cm^−1^) 2960, 2870, 1690, 1600, 1460, 1420, 1400, 1240, 1030, 820, 790; MS (EI), *m*/*z* (%) 178 (100), 177 (41), 163 (100), 160 (36), 135 (29), 119 (36), 105 (43), 103 (43), 91 (55), 77 (38); analyzed C 74.12, H 7.94, calculated for C_11_H_14_O_2_, C 74.13, H 7.92, O 17.95%; ^1^H NMR (CDCl_3_) δ 1.2761 (d, ^3^ *J*_8,9/10_ = 6.9, 6 H, CH_3_-9 and CH_3_-10), 2.9444 (septddd, ^3^ *J*_8,9/10_ = 6.9, ^4^ *J*_3,8_ = −0.6, ^4^ *J*_5,8_ = −0.5, ^5^ *J*_6,8_ = 0.3, 1 H, CH-8), 3.9365 (s, 3 H, CH_3_-11), 6.8201 (ddd, ^4^ *J*_3,5_ = 1.5, ^4^ *J*_3,8_ = −0.6, ^5^ *J*_3,6_ = 0.3, 1 H, CH-3), 6.9066 (dddd, ^3^ *J*_5,6_ = 8.0, ^4^ *J*_3,5_ = 1.5, ^5^ *J*_5,7_ = 0.7, ^4^ *J*_5,8_ = −0.5, 1 H, CH-5), 7.7633 (ddd, ^3^ *J*_5,6_ = 8.0, ^5^ *J*_3,6_ = 0.3, ^5^ *J*_6,8_ = 0.3, 1 H, CH-6), 10.4032 (d, ^5^ *J*_5,7_ = 0.7, 1 H, CH-7); ^13^C NMR (CDCl_3_) δ 23.72 (C-9, and C-10), 35.08 (C-8), 55.68 (C-11), 109.77 (C-3), 119.11 (C-5), 123.12 (C-1), 128.90 (C-6), 158.38 (C-4), 162.17 (C-2), 189.67 (C-7).

#### 3.7.3. Reduction of 4-Isopropyl-2-methoxybenzaldehyde (**2**)

A mixture of 4-isopropyl-2-methoxybenzaldehyde (**2**) (100 mg, 0.56 mmol) and NaBH_4_ (115 mg, 3 mmol) in anhydrous methanol (20 mL) was stirred at room temperature for 2 h [29]. Then, the methanol was removed under reduced pressure, and the aqueous solution of hydrochloric acid (1 M) was added to the residue and the mixture was extracted with diethyl ether three times. The combined organic layers were washed with aqueous sodium chloride solution and dried over anhydrous MgSO_4_. The solvent was removed under reduced pressure, and 2-methoxycuminol ((4-isopropyl-2-methoxyphenyl)methanol) was obtained as a yellowish liquid (90 mg, 89%). The purity of the product was checked by TLC and GC–MS.

(4-Isopropyl-2-methoxyphenyl)methanol (*syn*. 2-methoxycuminol): retention index (RI) = 1611 (DB-5MS column); UV (CH_3_CN) λ_max_(log ε) 273 (3.17), 220 (3.74), 200 (4.33) nm; FTIR (neat; cm^−1^) 3370, 2960, 2370, 1610, 1590, 1470, 1420, 1250, 1040, 860, 830; MS (EI), *m*/*z* (%) 180 (100), 164 (44), 163 (49), 149 (63), 137 (78), 115 (56), 105 (35), 104 (41), 94 (38), 40 (33); analyzed C 73.32, H 8.96, calculated for C_11_H_16_O_2_, C 73.30, H 8.95, O 17.75%; ^1^H NMR (CDCl_3_) δ 1.2552 (d, ^3^ *J*_8,9/10_ = 6.9, 6 H, CH_3_-9 and CH_3_-10), 2.2814 (brs, 1 H, OH), 2.9004 (septddd, ^3^ *J*_8,9/10_ = 6.9, ^4^ *J*_3,8_ = −0.6, ^4^ *J*_5,8_ = −0.6, ^5^ *J*_6,8_ = 0.3, 1 H, CH-8), 3.8786 (s, 3 H, CH_3_-11), 4.6505 (ddd, ^4^ *J*_6,7_ = −0.6, ^5^ *J*_3,7_ = 0.3, ^5^ *J*_5,7_ = 0.3, 2 H, CH_2_-7), 6.7549 (dddt, ^4^ *J*_3,5_ = 1.6, ^4^ *J*_3,8_ = −0.6, ^5^ *J*_3,6_ = 0.3, ^5^ *J*_3,7_ = 0.3, 1 H, CH-3), 6.8141 (dddt, ^3^ *J*_5,6_ = 7.6, ^4^ *J*_3,5_ = 1.6, ^4^ *J*_5,8_ = −0.6, ^5^ *J*_5,7_ = 0.3, 1 H, CH-5), 7.1799 (dtdd, ^3^ *J*_5,6_ = 7.6, ^4^ *J*_6,7_ = −0.6, ^5^ *J*_3,6_ = 0.3, ^5^ *J*_6,8_ = 0.3, 1 H, CH-6); ^13^C NMR (CDCl_3_) δ 24.18 (C-9, and C-10), 34.49 (C-8), 55.33 (C-11), 62.27 (C-7), 108.80 (C-3), 118.50 (C-5), 126.58 (C-1), 128.99 (C-6), 150.53 (C-4), 157.64 (C-2).

### 3.8. Synthesis of 2-Methoxycuminol Esters

Esters of 2-methoxycuminol and isobutyric, 2-methylbutyric, and isovaleric acids were prepared according to the general Steglich approach (*N*,*N*′-dicyclohexylcarbodiimide (DCC)/4-(dimethylamino)pyridine (DMAP)) [29]. A solution of 2-methoxycuminol (20 mg, 0.11 mmol), the appropriate carboxylic acid (0.11 mmol), DMAP (5 mg, 0.04 mmol), and DCC (23 mg, 0.11 mmol) in 20 mL of dry CH_2_Cl_2_ were stirred overnight at room temperature. Then, the precipitated urea was filtered off, and the filtrate was concentrated under reduced pressure. The resulting residue was purified by column chromatography on silica gel using an *n*-hexane/Et_2_O mixture (19:1, *v*/*v*) as the eluent. The yield of the synthesized 2-methoxycuminyl isobutyrate (**3**) was 91% (25.2 mg), 2-methoxycuminyl 2-methylbutyrate (**4**) 87% (25.5 mg), and 2-methoxycuminyl isovalerate (**5**) 92% (27.0 mg). The purity of the product was checked by TLC and GC–MS.

4-Isopropyl-2-methoxybenzyl isobutyrate (*syn*. 2-methoxycuminyl isobutyrate (**3**)): retention index (RI) = 1750 (DB-5MS column); UV (CH_3_CN) λ_max_(log ε) 274 (3.48), 199 (4.76) nm; FTIR (neat; cm^−1^) 2950, 1740, 1610, 1590, 1470, 1260, 1190, 1150, 1030, 810; MS (EI), *m*/*z* (%) 250 (17), 179 (50), 163 (100), 147 (15), 133 (33), 117 (15), 105 (23), 91 (26), 43 (64), 41 (36); analyzed C 71.96, H 8.85, calculated for C_15_H_22_O_3_, C 71.97, H 8.86, O 19.17%; ^1^H NMR (CDCl_3_) δ 1.1854 (d, ^3^ *J*_12,13/14_ = 7.0, 6 H, CH_3_-13 and CH_3_-14), 1.2565 (d, ^3^ *J*_8,9/10_ = 6.9, 6 H, CH_3_-9 and CH_3_-10), 2.5921 (sept, ^3^ *J*_12,13/14_ = 7.0, 1 H, CH-12), 2.8997 (septddd, ^3^ *J*_8,9/10_ = 6.9, ^4^ *J*_3,8_ = −0.6, ^4^ *J*_5,8_ = −0.5, ^5^ *J*_6,8_ = 0.3, 1 H, CH-8), 3.8389 (s, 3 H, CH_3_-15), 5.1236 (ddd, ^4^ *J*_6,7_ = −0.5, ^5^ *J*_3,7_ = 0.3, ^5^ *J*_5,7_ = 0.3, 2 H, CH_2_-7), 6.7494 (ddt, ^4^ *J*_3,5_ = 1.6, ^4^ *J*_3,8_ = −0.6, ^5^ *J*_3,7_ = 0.3, 1 H, CH-3), 6.8199 (dddt, ^3^ *J*_5,6_ = 7.7, ^4^ *J*_3,5_ = 1.6, ^4^ *J*_5,8_ = −0.5, ^5^ *J*_5,7_ = 0.3, 1 H, CH-5), 7.2303 (dtd, ^3^ *J*_5,6_ = 7.7, ^4^ *J*_6,7_ = −0.5, ^5^ *J*_6,8_ = 0.3, 1 H, CH-6); ^13^C NMR (CDCl_3_) δ 19.21 (C-13, and C-14), 24.13 (C-9, and C-10), 34.19 (C-12), 34.50 (C-8), 55.49 (C-15), 61.76 (C-7), 109.02 (C-3), 118.35 (C-5), 122.06 (C-1), 129.65 (C-6), 150.91 (C-4), 157.65 (C-2), 177.33 (C-11).

4-Isopropyl-2-methoxybenzyl 2-methylbutyrate (*syn*. 2-methoxycuminyl 2-methylbutyrate (**4**)): retention index (RI) = 1835 (DB-5MS column); UV (CH_3_CN) λ_max_(log ε) 274 (3.36), 221 (3.92), 198 (5.36) nm; FTIR (neat; cm^−1^) 2960, 2360, 1730, 1614, 1581, 1460, 1419, 1257, 1180, 1147, 1039, 819; MS (EI), *m*/*z* (%) 264 (14), 179 (44), 164 (13), 163 (100), 133 (25), 117 (14), 105 (19), 91 (16), 57 (26), 41 (22); analyzed C 72.67, H 9.16, calculated for C_16_H_24_O_3_, C 72.69, H 9.15, O 18.16%; ^1^H NMR (CDCl_3_) δ 0.9017 (ddd, ^3^ *J*_13A,14_ = 7.5, ^3^ *J*_13B,14_ = 7.5, ^4^ *J*_12,14_ = 0.3, 3 H, CH_3_-14), 1.1578 (d, ^3^ *J*_12,15_ = 7.0, 3 H, CH_3_-15), 1.2553 (d, ^3^ *J*_8,9/10_ = 6.9, 6 H, CH_3_-9 and CH_3_-10), 1.4801 (dqd, ^2^ *J*_13A,13B_ = −13.7, ^3^ *J*_13B,14_ = 7.5, ^3^ *J*_12,13B_ = 6.5, 1 H, CH-13B), 1.7004 (ddq, ^2^ *J*_13A,13B_ = −13.7, ^3^ *J*_12,13A_ = 7.5, ^3^ *J*_13A,14_ = 7.5, 1 H, CH-13A), 2.4147 (dqdq, ^3^ *J*_12,13A_ = 7.5, ^3^ *J*_12,15_ = 7.0, ^3^ *J*_12,13B_ = 6.5, ^4^ *J*_12,14_ = 0.3, 1 H, CH-12), 2.8983 (septddd, ^3^ *J*_8,9/10_ = 6.9, ^4^ *J*_3,8_ = −0.6, ^4^ *J*_5,8_ = −0.5, ^5^ *J*_6,8_ = 0.3, 1 H, CH-8), 3.8342 (s, 3 H, CH_3_-16), 5.1252 (ddd, ^4^ *J*_6,7_ = −0.5, ^5^ *J*_3,7_ = 0.3, ^5^ *J*_5,7_ = 0.3, 2 H, CH_2_-7), 6.7470 (ddt, ^4^ *J*_3,5_ = 1.6, ^4^ *J*_3,8_ = −0.6, ^5^ *J*_3,7_ = 0.3, 1 H, CH-3), 6.8175 (dddt, ^3^ *J*_5,6_ = 7.7, ^4^ *J*_3,5_ = 1.6, ^4^ *J*_5,8_ = −0.5, ^5^ *J*_5,7_ = 0.3, 1 H, CH-5), 7.2316 (dtd, ^3^ *J*_5,6_ = 7.7, ^4^ *J*_6,7_ = −0.5, ^5^ *J*_6,8_ = 0.3, 1 H, CH-6); ^13^C NMR (CDCl_3_) δ 11.73 (C-14), 16.81 (C-15), 24.13 (C-9, and C-10), 27.00 (C-13), 34.50 (C-8), 41.26 (C-12), 55.45 (C-16), 61.71 (C-7), 109.00 (C-3), 118.33 (C-5), 122.06 (C-1), 129.76 (C-6), 150.94 (C-4), 157.68 (C-2), 176.93 (C-11).

4-Isopropyl-2-methoxybenzyl isovalerate (*syn*. 2-methoxycuminyl isovalerate (**5**)): retention index (RI) = 1845 (DB-5MS column); UV (CH_3_CN) λ_max_(log ε) 274 (3.36), 223 (3.89), 200 (5.19) nm; FTIR (neat; cm^−1^) 2960, 1730, 1600, 1580, 1460, 1420, 1290, 1260, 1170, 1040, 820; MS (EI), *m*/*z* (%) 264 (24), 180 (15), 179 (100), 163 (95), 147 (23), 137 (16), 133 (31), 117 (16), 105 (21), 91 (20); analyzed C 72.68, H 9.18, calculated for C_16_H_24_O_3_, C 72.69, H 9.15, O 18.16%; ^1^H NMR (CDCl_3_) δ 0.9535 (d, ^3^ *J*_13,14/15_ = 6.7, 6 H, CH_3_-14 and CH_3_-15), 1.2544 (d, ^3^ *J*_8,9/10_ = 6.9, 6 H, CH_3_-9 and CH_3_-10), 2.1232 (tsept, ^3^ *J*_12,13_ = 7.2, ^3^ *J*_13,14/15_ = 6.7, 1 H, CH-13), 2.2252 (d, ^3^ *J*_12,13_ = 7.2, 2 H, CH_2_-13), 2.8987 (septddd, ^3^ *J*_8,9/10_ = 6.9, ^4^ *J*_3,8_ = −0.6, ^4^ *J*_5,8_ = −0.5, ^5^ *J*_6,8_ = 0.3, 1 H, CH-8), 3.8380 (s, 3 H, CH_3_-16), 5.1237 (ddd, ^4^ *J*_6,7_ = −0.5, ^5^ *J*_3,7_ = 0.3, ^5^ *J*_5,7_ = 0.3, 2 H, CH_2_-7), 6.7494 (ddt, ^4^ *J*_3,5_ = 1.6, ^4^ *J*_3,8_ = −0.6, ^5^ *J*_3,7_ = 0.3, 1 H, CH-3), 6.8176 (dddt, ^3^ *J*_5,6_ = 7.7, ^4^ *J*_3,5_ = 1.6, ^4^ *J*_5,8_ = −0.5, ^5^ *J*_5,7_ = 0.3, 1 H, CH-5), 7.2350 (dtd, ^3^ *J*_5,6_ = 7.7, ^4^ *J*_6,7_ = −0.5, ^5^ *J*_6,8_ = 0.3, 1 H, CH-6); ^13^C NMR (CDCl_3_) δ 22.54 (C-14, and C-15), 24.12 (C-9, and C-10), 25.93 (C-13), 34.52 (C-8), 43.66 (C-12), 55.48 (C-16), 61.71 (C-7), 109.03 (C-3), 118.36 (C-5), 121.88 (C-1), 130.07 (C-6), 151.10 (C-4), 157.75 (C-2), 173.35 (C-11).

### 3.9. Synthesis of Thymohydroquinone

#### 3.9.1. Synthesis of Thymoquinone (**7**)

To a solution of 15 g (0.10 mol) of thymol (**6**) in 100 mL of 96% ethanol was added 100 mL of concentrated hydrochloric acid (37%, *w*/*w*). This mixture was cooled to 0° C in a beaker set in an ice-salt bath, and to it was added 10 g (0.15 mol) of sodium nitrite in portions with vigorous stirring. Then, the bulk of the product was transferred to a flask containing 200 mL of cold water. The light-yellow crude nitrosothymol product was vacuum-filtered and washed with water [36]. It was then dissolved in a mixture of 15 mL of acetone and 150 mL of 2-methoxyethanol. After the nitrosothymol was dissolved, 47 mL of concentrated hydrochloric acid, 60 mL of distilled water, and 20 g (0.20 mol) of copper(I) chloride were added to it, and the mixture was refluxed for 45 min [37]. The reaction mixture was cooled and extracted three times with *n*-hexane. The combined organic layers were washed with an aqueous sodium hydroxide solution (10%, *w*/*w*) until it was colorless, then washed with a saturated solution of sodium chloride and dried over anhydrous magnesium sulfate. Thymoquinone (**7**) was obtained after the removal of the solvent under reduced pressure as yellow crystals (10 g, 61%). The purity of the product was checked by TLC and GC–MS.

Thymoquinone (**7**): retention index (RI) = 1255 (DB-5MS column); MS (EI)*:* in complete agreement with the values published by Schreiner et al., 2019 [34].

#### 3.9.2. Synthesis of Thymohydroquinone

Thymoquinone (**7**) (2 g, 12 mmol) was dissolved in glacial acetic acid (100 mL), and the mixture was stirred at room temperature for 4 h under a nitrogen atmosphere. Afterwards, 60 g of zinc powder was added [34]. The mixture was filtered, and the solvent was removed under reduced pressure. The residue was dissolved in water, and the aqueous layer was extracted three times with diethyl ether. The organic phase was separated and washed with brine. The solvent was removed under reduced pressure, and the product was obtained as a white solid (1.7 g, 84%). The purity of the product was checked by TLC and GC–MS.

Thymohydroquinone: retention index (RI) = 1560 (DB-5MS column); MS (EI)*:* in complete agreement with the values published by Schreiner et al., 2019 [34].

### 3.10. Synthesis of Thymohydroquinone Esters

Mono- and diesters of thymohydroquinone with isobutyric and 2-methylbutyric acids were prepared in analogy to the synthesis of 2-methoxycuminyl esters. The amounts of substances used for synthesis: 500 mg (3 mmol) of thymohydroquinone, 3 mmol of the appropriate carboxylic acid, 80 mg (0.7 mmol) of DMAP, 620 mg (3 mmol) of DCC, and 30 mL of dry CH_2_Cl_2_ [29]. In this case, the eluent for chromatography was *n*-hexane/Et_2_O, 4:1 (*v*/*v*). The yield of 4-hydroxy-2-isopropyl-5-methylphenyl isobutyrate (**8**) was 10% (71 mg), 4-hydroxy-5-isopropyl-2-methylphenyl isobutyrate (**10**) was 15% (107 mg), 4-hydroxy-2-isopropyl-5-methylphenyl 2-methylbutyrate (**9**) was 8% (60 mg), and 4-hydroxy-5-isopropyl-2-methylphenyl 2-methylbutyrate (**11**) was 14% (105 mg).

4-Hydroxy-2-isopropyl-5-methylphenyl isobutyrate (*syn*. 6-hydroxythymyl isobutyrate (**8**)): yellowish liquid; retention index (RI) = 1769 (DB-5MS column); UV (CH_3_CN) λ_max_(log ε) 279 (3.32), 197 (4.51) nm; FTIR (neat; cm^−1^) 3439, 2963, 2873, 2359, 2341, 1752, 1727, 1627, 1517, 1456, 1412, 1387, 1342, 1254, 1171, 1140, 1059, 1032, 922, 886, 853, 819, 743, 585; MS (EI), *m*/*z* (%) 236 (8), 167 (10), 166 (100), 152 (8), 151 (82), 91 (7), 77 (9), 71 (8), 43 (26), 41 (10); analyzed C 71.15, H 8.55, calculated for C_14_H_20_O_3_, C 71.16, H 8.53, O 20.31%; ^1^H NMR (CDCl_3_) δ 1.1603 (d, ^3^ *J*_8,9/10_ = 6.9, 6 H, CH_3_-9 and CH_3_-10), 1.3271 (d, ^3^ *J*_13,14/15_ = 7.0, 6 H, CH_3_-13 and CH_3_-14), 2.1716 (dd, ^4^ *J*_6,7_ = −0.7, ^5^ *J*_3,7_ = 0.3, 3 H, CH_3_-7), 2.8132 (sept, ^3^ *J*_13,14/15_ = 7.0, 1 H, CH-12), 2.9050 (septdd, ^3^ *J*_8,9/10_ = 6.9, ^4^ *J*_3,8_ = −0.6, ^5^ *J*_6,8_ = 0.3, 1 H, CH-8), 4.8221 (brs, 1 H, OH), 6.6855 (qdd, ^4^ *J*_6,7_ = −0.7, ^5^ *J*_3,6_ = 0.3, ^5^ *J*_6,8_ = 0.3, 1 H, CH-6), 6.7164 (ddq, ^4^ *J*_3,8_ = −0.6, ^5^ *J*_3,6_ = 0.3, ^5^ *J*_3,7_ = 0.3, 1 H, CH-3); ^13^C NMR (CDCl_3_) δ 15.53 (C-7), 19.18 (C-13 and C-14), 23.03 (C-9, and C-10), 27.22 (C-8), 34.36 (C-12), 112.83 (C-3), 122.10 (C-1), 124.26 (C-6), 138.88 (C-4), 141.40 (C-5), 151.80 (C-2), 176.41 (C-11).

4-Hydroxy-5-isopropyl-2-methylphenyl isobutyrate (*syn*. 6-isobutyryloxythymol (**10**)): yellowish liquid; retention index (RI) = 1778 (DB-5MS column); UV (CH_3_CN) λ_max_(log ε) 279 (3.46), 197 (4.53) nm; FTIR (neat; cm^−1^) 3452, 2967, 2872, 2362, 2342, 1725, 1628, 1590, 1519, 1455, 1412, 1377, 1368, 1359, 1341, 1291, 1275, 1231, 1180, 1172, 1156, 1140, 1120, 1087, 1034, 997, 962, 925, 886, 821, 756, 734, 685, 571; MS (EI), *m*/*z* (%) 236 (6), 166 (100), 151 (86), 91 (6),79 (6), 77 (8), 71 (8), 43 (27), 41 (9); analyzed C 71.15, H 8.55, calculated for C_14_H_20_O_3_, C 71.16, H 8.53, O 20.31%; ^1^H NMR (CDCl_3_) δ 1.1853 (d, ^3^ *J*_8,9/10_ = 6.9, 6 H, CH_3_-9 and CH_3_-10), 1.3347 (d, ^3^ *J*_12,13/14_ = 7.0, 6 H, CH_3_-13 and CH_3_-14), 2.0268 (dd, ^4^ *J*_6,7_ = −0.7, ^5^ *J*_3,7_ = 0.3, 3 H, CH_3_-7), 2.8238 (sept, ^3^ *J*_12,13/14_ = 7.0, 1 H, CH-12), 3.1200 (septdd, ^3^ *J*_8,9/10_ = 6.9, ^4^ *J*_3,8_ = −0.6, ^5^ *J*_6,8_ = 0.3, 1 H, CH-8), 5.5035 (brs, 1 H, OH), 6.4482 (qdd, ^4^ *J*_6,7_ = −0.7, ^5^ *J*_3,6_ = 0.3, ^5^ *J*_6,8_ = 0.3, 1 H, CH-6), 6.7308 (ddq, ^4^ *J*_3,8_ = −0.6, ^5^ *J*_3,6_ = 0.3, ^5^ *J*_3,7_ = 0.3, 1 H, CH-3); ^13^C NMR (CDCl_3_) δ 15.84 (C-7), 19.22 (C-13 and C-14), 22.58 (C-9, and C-10), 26.90 (C-8), 34.32 (C-12), 117.58 (C-6), 119.31 (C-3), 127.61 (C-1), 133.44 (C-4), 142.77 (C-2), 150.70 (C-5), 176.49 (C-11).

4-Hydroxy-2-isopropyl-5-methylphenyl 2-methylbutyrate (*syn*. 6-hydroxythymyl 2-methylbutyrate (**9**)): yellowish liquid; retention index (RI) = 1861 (DB-5MS column); UV (CH_3_CN) λ_max_(log ε) 279 (3.43), 196 (4.71) nm; FTIR (neat; cm^−1^) 3432, 2964, 2933, 2875, 2360, 2342, 1751, 1724, 1626, 1592, 1500, 1456, 1410, 1380, 1363, 1262, 1233, 1165, 1129, 1065, 1034, 1006, 966, 907, 873, 818, 754; MS (EI), *m*/*z* (%) 250 (6), 167 (11), 166 (100), 152 (6), 151 (64), 137 (6), 91 (6), 77 (8), 57 (29), 41 (11); analyzed C 71.95, H 8.87, calculated for C_15_H_22_O_3_, C 71.97, H 8.86, O 19.17%. ^1^H NMR (CDCl_3_) δ 1.0299 (ddd, ^3^ *J*_13A,14_ = 7.5, ^3^ *J*_13B,14_ = 7.5, ^4^ *J*_12,14_ = 0.3, 3 H, CH_3_-14), 1.1607 (d, ^3^ *J*_8,9/10_ = 6.9, 6 H, CH_3_-9 and CH_3_-10), 1.3062 (d, ^3^ *J*_12,15_ = 7.0, 3 H, CH_3_-15), 1.6132 (dqd, ^2^ *J*_13A,13B_ = −13.7, ^3^ *J*_13B,14_ = 7.5, ^3^ *J*_12,13B_ = 6.5, 1 H, CH-13B), 1.8575 (ddq, ^2^ *J*_13A,13B_ = −13.7, ^3^ *J*_12,13A_ = 7.5, ^3^ *J*_13A,14_ = 7.5, 1 H, CH-13A), 2.1721 (dd, ^4^ *J*_6,7_ = −0.8, ^5^ *J*_3,7_ = 0.3, 3 H, CH_3_-7), 2.6268 (dqdq, ^3^ *J*_12,13A_ = 7.5, ^3^ *J*_12,15_ = 7.0, ^3^ *J*_12,13B_ = 6.5, ^4^ *J*_12,14_ = 0.3, 1 H, CH-12), 2.9189 (septdd, ^3^ *J*_8,9/10_ = 6.9, ^4^ *J*_3,8_ = −0.6, ^5^ *J*_6,8_ = 0.3, 1 H, CH-8), 4.7275 (brs, 1 H, OH), 6.6864 (ddq, ^4^ *J*_3,8_ = −0.6, ^5^ *J*_3,6_ = 0.3, ^5^ *J*_3,7_ = 0.3, 1 H, CH-3), 6.7059 (qdd, ^4^ *J*_6,7_ = −0.8, ^5^ *J*_3,6_ = 0.3, ^5^ *J*_6,8_ = 0.3, 1 H, CH-6); ^13^C NMR (CDCl_3_) δ 11.89 (C-14), 15.53 (C-7), 16.88 (C-15), 23.08 (C-9, and C-10), 26.89 (C-13), 27.16 (C-8), 41.45 (C-12), 112.81 (C-3), 122.08 (C-1), 124.26 (C-6), 138.93 (C-4), 141.38 (C-5), 151.79 (C-2), 175.99 (C-11).

4-Hydroxy-5-isopropyl-2-methylphenyl 2-methylbutyrate (*syn*. 6-(2-methylbutyryloxy)thymol (**11**)): yellowish liquid; retention index (RI) = 1876 (DB-5MS column); UV (CH_3_CN) λ_max_(log ε) 279 (3.42), 196 (4.63) nm; FTIR (neat; cm^−1^) 3451, 2963, 2932, 2875, 1752, 1725, 1626, 1517, 1456, 1412, 1379, 1363, 1339, 1296, 1255, 1227, 1168, 1140, 1066, 1007, 966, 909, 876, 853, 814, 742, 596; MS (EI), *m*/*z* (%) 250 (5), 167 (11), 166 (100), 152 (6), 151 (63), 91 (5), 79 (6), 77 (6), 57 (27), 41 (9); analyzed C 71.95, H 8.87, calculated for C_15_H_22_O_3_, C 71.97, H 8.86, O 19.17%; ^1^H NMR (CDCl_3_) δ 1.0354 (ddd, ^3^ *J*_13A,14_ = 7.5, ^3^ *J*_13B,14_ = 7.5, ^4^ *J*_12,14_ = 0.3, 3 H, CH_3_-14), 1.2178 (d, ^3^ *J*_8,9/10_ = 6.9, 6 H, CH_3_-9 and CH_3_-10), 1.3133 (d, ^3^ *J*_12,15_ = 7.0, 3 H, CH_3_-15), 1.6175 (dqd, ^2^ *J*_13A,13B_ = −13.7, ^3^ *J*_13B,14_ = 7.5, ^3^ *J*_12,13B_ = 6.5, 1 H, CH-13B), 1.8686 (ddq, ^2^ *J*_13A,13B_ = −13.7, ^3^ *J*_12,13A_ = 7.5, ^3^ *J*_13A,14_ = 7.5, 1 H, CH-13A), 2.0693 (dd, ^4^ *J*_6,7_ = −0.7, ^5^ *J*_3,7_ = 0.3, 3 H, CH_3_-7), 2.6302 (dqdq, ^3^ *J*_12,13A_ = 7.5, ^3^ *J*_12,15_ = 7.0, ^3^ *J*_12,13B_ = 6.5, ^4^ *J*_12,14_ = 0.3, 1 H, CH-12), 3.1252 (septdd, ^3^ *J*_8,9/10_ = 6.9, ^4^ *J*_3,8_ = −0.6, ^5^ *J*_6,8_ = 0.3, 1 H, CH-8), 4.7304 (brs, 1 H, OH), 6.5653 (qd, ^4^ *J*_6,7_ = −0.7, ^5^ *J*_6,8_ = 0.3, 1 H, CH-6), 6.7498 (dq, ^4^ *J*_3,8_ = −0.6, ^5^ *J*_3,7_ = 0.3, 1 H, CH-3); ^13^C NMR (CDCl_3_) δ 11.78 (C-14), 16.28 (C-7), 16.78 (C-15), 22.60 (C-9, and C-10), 26.59 (C-8), 26.78 (C-13), 41.23 (C-12), 112.75 (C-6), 119.41 (C-3), 127.39 (C-1), 135.69 (C-4), 142.70 (C-2), 154.30 (C-5), 175.25 (C-11).

### 3.11. Synthesis of O-Methyl Thymohydroquinone Monoesters

Methylation of the corresponding thymohydroquinone monoester was analogous to carvacrol. The amounts of substances used for synthesis: 20 mg (0.08 mmol) of thymohydroquinone monoester, 100 mg (0.72 mmol) of anhydrous potassium carbonate, 1 mL of methyl iodide and 20 mL of acetone [29]. The yield of 2-isopropyl-4-methoxy-5-methylphenyl isobutyrate (**12**) was 99% (21 mg), 5-isopropyl-4-methoxy-2-methylphenyl isobutyrate (**14**) was 94% (20 mg), 2-isopropyl-4-methoxy-5-methylphenyl 2-methylbutyrate (**13**) was 92% (19.5 mg), and 5-isopropyl-4-methoxy-2-methylphenyl 2-methylbutyrate (**15**) was 90% (19 mg).

2-Isopropyl-4-methoxy-5-methylphenyl isobutyrate (*syn*. 6-methoxythymyl isobutyrate (**12**)): white solid; retention index (RI) = 1691 (DB-5MS column); UV (CH_3_CN) λ_max_(log ε) 278 (3.39), 197 (4.65) nm; FTIR (neat; cm^−1^) 2961, 2916, 2873, 2850, 1744, 1619, 1504, 1468, 1458, 1441, 1399, 1387, 1369, 1346, 1303, 1267, 1199, 1176, 1152, 1135, 1089, 1051, 1004, 922, 895, 871‚ 847, 816, 756, 737, 675, 603; MS (EI): in complete agreement with the values published by Bolman et al., 1978 [38]; analyzed C 71.96, H 8.85, calculated for C_15_H_22_O_3_, C 71.97, H 8.86, O 19.17%; ^1^H NMR (CDCl_3_) δ 1.1988 (d, ^3^ *J*_8,9/10_ = 6.9, 6 H, CH_3_-9 and CH_3_-10), 1.3287 (d, ^3^ *J*_13,14/15_ = 7.0, 6 H, CH_3_-14 and CH_3_-15), 2.1616 (dd, ^4^ *J*_6,7_ = −0.8, ^5^ *J*_3,7_ = 0.4, 3 H, CH_3_-7), 2.8133 (sept, ^3^ *J*_13,14/15_ = 7.0, 1 H, CH-13), 2.9627 (septd, ^3^ *J*_8,9/10_ = 6.9, ^4^ *J*_3,8_ = −0.5, 1 H, CH-8), 3.8240 (s, 3 H, CH_3_-11), 6.7135 (dq, ^4^ *J*_3,8_ = −0.5, ^5^ *J*_3,7_ = 0.4, 1 H, CH-3), 6.7368 (q, ^4^ *J*_6,7_ = −0.8, 1 H, CH-6); ^13^C NMR (CDCl_3_) δ 15.92 (C-7), 19.19 (C-14 and C-15), 23.11 (C-9, and C-10), 27.54 (C-8), 34.35 (C-13), 55.75 (C-11), 107.81 (C-3), 124.18 (C-6), 125.12 (C-1), 138.02 (C-4), 141.08 (C-5), 155.75 (C-2), 176.28 (C-12).

5-Isopropyl-4-methoxy-2-methylphenyl isobutyrate (*syn*. 6-isobutyryloxythymyl methyl ether (**14**)): colorless liquid; retention index (RI) = 1711 (DB-5MS column); UV (CH_3_CN) λ_max_(log ε) 278 (3.37), 197 (4.62) nm; FTIR (neat; cm^−1^) 2961, 2872, 2364, 2338, 1975, 1751, 1620, 1504, 1464, 1400, 1386, 1344, 1249, 1194, 1173, 1130, 1090, 1060, 1047, 1007, 919, 887, 842, 815, 749, 620, 546; MS (EI): in complete agreement with the values published by Weyerstahl et al., 1993 [39]; analyzed C 71.96, H 8.85, calculated for C_15_H_22_O_3_, C 71.97, H 8.86, O 19.17%.; ^1^H NMR (CDCl_3_) δ 1.1762 (d, ^3^ *J*_8,9/10_ = 6.9, 6 H, CH_3_-9 and CH_3_-10), 1.3342 (d, ^3^ *J*_12,13/14_ = 7.0, 6 H, CH_3_-13 and CH_3_-14), 2.1210 (dd, ^4^ *J*_6,7_ = −0.7, ^5^ *J*_3,7_ = 0.3, 3 H, CH_3_-7), 2.8174 (sept, ^3^ *J*_12,13/14_ = 7.0, 1 H, CH-12), 3.2452 (septdd, ^3^ *J*_8,9/10_ = 6.9, ^4^ *J*_3,8_ = −0.6, ^5^ *J*_6,8_ = 0.3, 1 H, CH-8), 3.7984 (s, 3 H, CH_3_-15), 6.6652 (qdd, ^4^ *J*_6,7_ = −0.7, ^5^ *J*_3,6_ = 0.3, ^5^ *J*_6,8_ = 0.3, 1 H, CH-6), 6.7830 (ddq, ^4^ *J*_3,8_ = −0.6, ^5^ *J*_3,6_ = 0.3, ^5^ *J*_3,7_ = 0.3, 1 H, CH-6); ^13^C NMR (CDCl_3_) δ 16.30 (C-7), 19.26 (C-13 and C-14), 22.74 (C-9, and C-10), 26.70 (C-8), 34.30 (C-12), 55.81 (C-15), 112.86 (C-6), 119.50 (C-3), 127.49 (C-1), 135.83 (C-4), 142.81 (C-2), 154.42 (C-5), 175.78 (C-11).

2-Isopropyl-4-methoxy-5-methylphenyl 2-methylbutyrate (*syn*. 6-methoxythymyl 2-methylbutyrate (**13**)): colorless liquid; retention index (RI) = 1783 (DB-5MS column); UV (CH_3_CN) λ_max_(log ε) 278 (3.42), 197 (4.73) nm; FTIR (neat; cm^−1^) 2961, 2915, 2850, 2365, 2341, 2182, 2139, 2066, 2050, 2038, 2029, 2020, 2009, 2001, 1990, 1977, 1956, 1750, 1677, 1619, 1586, 1501, 1461, 1400, 1379, 1363, 1345, 1302, 1265, 1225, 1197, 1160, 1151, 1125, 1089, 1056, 1012, 964, 905, 870, 848, 813, 752, 720, 669, 548; MS (EI), *m*/*z* (%) 264 (5), 181 (12), 180 (100), 179 (5), 166 (6), 165 (56), 91 (8), 77 (5), 57 (19), 41 (7); analyzed C 72.70, H 9.12, calculated for C_16_H_24_O_3_, C 72.69, H 9.15, O 18.16%; ^1^H NMR (CDCl_3_) δ 1.0325 (ddd, ^3^ *J*_14A,15_ = 7.5, ^3^ *J*_14B,15_ = 7.5, ^4^ *J*_13,15_ = 0.3, 3 H, CH_3_-15), 1.1999 (d, ^3^ *J*_8,9/10_ = 6.9, 6 H, CH_3_-9 and CH_3_-10), 1.3092 (d, ^3^ *J*_13,16_ = 7.0, 3 H, CH_3_-16), 1.6148 (dqd, ^2^ *J*_14A,14B_ = −13.7, ^3^ *J*_14B,15_ = 7.5, ^3^ *J*_13,14B_ = 6.5, 1 H, CH-14B), 1.8620 (ddq, ^2^ *J*_14A,14B_ = −13.7, ^3^ *J*_13,14A_ = 7.5, ^3^ *J*_14A,15_ = 7.5, 1 H, CH-14A), 2.1631 (dd, ^4^ *J*_6,7_ = −0.8, ^5^ *J*_3,7_ = 0.4, 3 H, CH_3_-7), 2.6280 (dqdq, ^3^ *J*_13,14A_ = 7.5, ^3^ *J*_13,16_ = 7.0, ^3^ *J*_13,14B_ = 6.5, ^4^ *J*_13,15_ = 0.3, 1 H, CH-13), 2.9772 (septd, ^3^ *J*_8,9/10_ = 6.9, ^4^ *J*_3,8_ = −0.6, 1 H, CH-8), 3.8256 (s, 3 H, CH_3_-11), 6.7157 (dq, ^4^ *J*_3,8_ = −0.6, ^5^ *J*_3,7_ = 0.4, 1 H, CH-3), 6.7274 (q, ^4^ *J*_6,7_ = −0.8, 1 H, CH-6); ^13^C NMR (CDCl_3_) δ 11.90 (C-15), 15.93 (C-7), 16.90 (C-16), 23.16 (C-9, and C-10), 26.91 (C-14), 27.48 (C-8), 41.45 (C-13), 55.77 (C-11), 107.81 (C-3), 124.19 (C-6), 125.14 (C-1), 138.07 (C-4), 141.07 (C-5), 155.76 (C-2), 175.86 (C-12).

5-Isopropyl-4-methoxy-2-methylphenyl 2-methylbutyrate (*syn*. 6-(2-methylbutyryloxy)thymyl methyl ether (**15**)): colorless liquid; retention index (RI) = 1803 (DB-5MS column); UV (CH_3_CN) λ_max_(log ε) 278 (3.40), 197 (4.70) nm; FTIR (neat; cm^−1^) 2962, 2932, 2875, 1750, 1621, 1504, 1461, 1400, 1380, 1361, 1346, 1300, 1250, 1225, 1194, 1169, 1125, 1090, 1059, 1012, 965, 906, 876, 841, 812, 733, 546; MS (EI), *m*/*z* (%) 264 (3), 181 (11), 180 (100), 166 (2), 165 (62), 65 (1), 57 (18), 55 (2), 41 (4); analyzed C 72.70, H 9.14, calculated for C_16_H_24_O_3_, C 72.69, H 9.15, O 18.16%; ^1^H NMR (CDCl_3_) δ 1.0352 (ddd, ^3^ *J*_13A,14_ = 7.5, ^3^ *J*_13B,14_ = 7.5, ^4^ *J*_12,14_ = 0.3, 3 H, CH_3_-14), 1.1769 (d, ^3^ *J*_8,9/10_ = 6.9, 6 H, CH_3_-9 and CH_3_-10), 1.3133 (d, ^3^ *J*_12,15_ = 7.0, 3 H, CH_3_-15), 1.6169 (dqd, ^2^ *J*_13A,13B_ = −13.7, ^3^ *J*_13B,14_ = 7.5, ^3^ *J*_12,13B_ = 6.5, 1 H, CH-13B), 1.8704 (ddq, ^2^ *J*_13A,13B_ = −13.7, ^3^ *J*_12,13A_ = 7.5, ^3^ *J*_13A,14_ = 7.5, 1 H, CH-13A), 2.1303 (dd, ^4^ *J*_6,7_ = −0.7, ^5^ *J*_3,7_ = 0.4, 3 H, CH_3_-7), 2.6293 (dqdq, ^3^ *J*_12,13A_ = 7.5, ^3^ *J*_12,15_ = 7.0, ^3^ *J*_12,13B_ = 6.5, ^4^ *J*_12,14_ = 0.3, 1 H, CH-12), 3.2436 (septdd, ^3^ *J*_8,9/10_ = 6.9, ^4^ *J*_3,8_ = −0.6, ^5^ *J*_6,8_ = 0.4, 1 H, CH-8), 3.7968 (s, 3 H, CH_3_-16), 6.6660 (qdd, ^4^ *J*_6,7_ = −0.7, ^5^ *J*_6,8_ = 0.4, ^5^ *J*_3,6_ = 0.3, 1 H, CH-6), 6.7701 (dqd, ^4^ *J*_3,8_ = −0.6, ^5^ *J*_3,7_ = 0.4, ^5^ *J*_3,6_ = 0.3, 1 H, CH-3); ^13^C NMR (CDCl_3_) δ 11.90 (C-14), 16.40 (C-7), 16.91 (C-15), 22.71/22.73 (C-9, and C-10), 26.72 (C-8), 26.91 (C-13), 41.36 (C-12), 55.80 (C-16), 112.87 (C-6), 119.53 (C-3), 127.51 (C-1), 135.80 (C-4), 142.81 (C-2), 154.42 (C-5), 175.36 (C-11).

### 3.12. Synthesis of 2-(2-Methoxy-4-methylphenyl)propan-1-ol (***19***)

#### 3.12.1. Acetylation of *m*-Cresol

The mixture of *m*-cresol (**16**) (10 g, 93 mmol), acetic anhydride (15 g, 147 mmol), and *p*-toluenesulfonic acid (0.5 g, 3 mmol) was refluxed for 30 min. Then, the reaction mixture was cooled, and the excess acetic anhydride was destroyed with an aqueous solution of sodium hydroxide (10%, *w*/*w*). The mixture was extracted three times with diethyl ether, and the combined organic layers were washed with a saturated aqueous sodium chloride solution and dried over anhydrous MgSO_4_. The solvent was removed under reduced pressure, and *m*-tolyl acetate was obtained as a colorless liquid (12.94 g, 93%). The purity of the product was checked by TLC and GC–MS.

*m*-Tolyl acetate: colorless liquid; MS (EI), *m*/*z* (%) 150 (16), 109 (7), 108 (100), 107 (45), 90 (7), 80 (7), 79 (14), 77 (18), 51 (6), 43 (13).

#### 3.12.2. Fries Rearrangements of *m*-Tolyl Acetate

*m*-Tolyl acetate (11 g, 0.07 mol) was cooled in an ice/water bath, and 12 g (0.09 mol) of anhydrous aluminum chloride was added in portions. The mixture was then heated to 130 °C in an oil bath [40]. After all of the hydrogen chloride was released, the temperature was increased to 160 °C and maintained for 1.5 h. After cooling, a mixture of ice water and concentrated hydrochloric acid was added to the reaction mixture. The resulting mixture was extracted three times with *n*-hexane. The combined organic layers were washed with a saturated aqueous sodium chloride solution and dried over anhydrous MgSO_4_. The solvent was removed under reduced pressure, and the residue was purified by column chromatography on silica gel using an *n*-hexane/Et_2_O mixture (9:1, *v*/*v*) as the eluent. The yield of 2-hydroxy-4-methylacetophenone (**17**) was 8 g (73%). The purity of the product was confirmed by TLC and GC–MS.

2-Hydroxy-4-methylacetophenone: colorless liquid (**17**); MS (EI)*:* in complete agreement with the values published by Schreiner et al., 2019 [34].

#### 3.12.3. *O*-Methylation of 2-Hydroxy-4-methylacetophenone (**17**)

Methylation of 2-hydroxy-4-methylacetophenone (**17**) was analogous to that of carvacrol. The amounts of substances used for synthesis: 2 g (0.013 mol) of 2-hydroxy-4-methylacetophenone (**17**), 6 g (0.04 mol) of anhydrous potassium carbonate, 2 mL of methyl iodide, and 50 mL of acetone [29]. The compound 2-methoxy-4-methylacetophenone was obtained as a colorless liquid (2 g, 92%). The purity of the product was checked by TLC and GC–MS.

2-Methoxy-4-methylacetophenone: colorless liquid; MS (EI): in complete agreement with the values published by Montel et al., 2010 [41].

#### 3.12.4. Synthesis of Methyltriphenylphosphonium Iodide

Triphenylphosphine (10 g, 0.04 mol) was dissolved in 100 mL of benzene, and 3 mL of methyl iodide was added to the solution dropwise [42]. The mixture was stirred for 24 h under an inert atmosphere of argon. Then, the mixture was vacuum filtered, and the precipitate was washed several times with *n*-hexane. The precipitate of methyltriphenylphosphonium iodide was dried in an oven at 145 °C and stored in a desiccator. The yield of methyltriphenylphosphonium iodide was 14.8 g (96%), Mp = 186.8 °C.

#### 3.12.5. Synthesis of Dehydrothymyl Methyl Ether (2-Methoxy-4-methyl-1-(prop-1-en-2-yl)benzene)

An oven-dried 100 mL three-neck flask, equipped with a magnetic stirring bar and a rubber septum, was charged with methyltriphenylphosphonium iodide (6.1 g, 15 mmol). The flask was evacuated and backfilled with nitrogen. THF (40 mL) was added, and the mixture was cooled to 0 °C in an ice/water bath. *n*-BuLi (6.5 mL, 2.3 M in hexane) was added to the flask, followed by the dropwise addition of 2-methoxy-4-methylacetophenone (1.7 g, 10 mmol) [43]. The ice/water bath was then removed, and the reaction mixture was stirred overnight at room temperature. A saturated aqueous sodium chloride solution was added to the flask, and the mixture was extracted three times with diethyl ether. The combined organic layers were washed with a saturated aqueous sodium chloride solution and dried over anhydrous MgSO_4_. The solvent was removed under reduced pressure, and the residue was purified by column chromatography on silica gel using an *n*-hexane/Et_2_O mixture (19:1, *v*/*v*) as the eluent. The yield of dehydrothymyl methyl ether (**18**) was 1.2 g (71%). The purity of the product was confirmed by TLC and GC–MS.

8,9-Dehydrothymyl methyl ether (**18**): colorless liquid; retention index (RI) = 1252 (DB-5MS column); UV (CH_3_CN) λ_max_(log ε) 282 (3.39), 237 (3.90), 204 (4.45) nm; FTIR (neat; cm^−1^) 3078, 2918, 2362, 2342, 1631, 1608, 1570, 1503, 1463, 1405, 1369, 1307, 1271, 1250, 1191, 1169, 1138, 1096, 1039, 1005, 935, 891, 847, 813, 755, 727, 594, 580; MS (EI): in complete agreement with the values published by Du et al., 2004 [44]; analyzed C 81.45, H 8.71, calculated for C_11_H_14_O, C 81.44, H 8.70, O 9.86%; ^1^H NMR (CDCl_3_) δ 2.1036 (dd, ^3^ *J*_9A,10_ = −1.5, ^3^ *J*_9B,10_ = −0.9, 3 H, CH_3_-10), 2.3461 (ddd, ^4^ *J*_6,7_ = −0.8, ^4^ *J*_2,7_ = −0.6, ^5^ *J*_5,7_ = 0.3, 3 H, CH_3_-7), 3.8152 (s, 3 H, CH_3_-11), 5.0462 (dq, ^2^ *J*_9A,9B_ = 2.4, ^3^ *J*_9B,10_ = −0.9, 1 H, CH-9B), 5.1203 (dq, ^2^ *J*_9A,9B_ = 2.4, ^3^ *J*_9A,10_ = −1.5, 1 H, CH-9A), 6.6951 (dq, ^4^ *J*_2,6_ = 1.5, ^4^ *J*_2,7_ = −0.6, 1 H, CH-2), 6.7314 (ddq, ^3^ *J*_5,6_ = 7.6, ^4^ *J*_2,6_ = 1.5, ^4^ *J*_6,7_ = −0.8, 1 H, CH-6), 7.0739 (dq, ^3^ *J*_5,6_ = 7.6, ^5^ *J*_5,7_ = 0.3, 1 H, CH-5); ^13^C NMR (CDCl_3_) δ 21.61 (C-7), 23.44 (C-10), 55.51 (C-11), 111.87 (C-2), 114.89 (C-9), 121.18 (C-6), 129.27 (C-5), 129.95 (C-4), 138.48 (C-1), 144.24 (C-8), 156.60 (C-3).

#### 3.12.6. Synthesis of 2-(2-Methoxy-4-methylphenyl)propan-1-ol (Syn. 9-Hydroxythymyl Methyl Ether (**19**))

In a 100 mL dry three-necked flask filled with argon and equipped with an internal thermometer, a magnetic stirrer and a dropping funnel, 10 mL of dry tetrahydrofuran (THF) was added, followed by 2.5 mmol (100 mg) of finely powdered sodium borohydride. The mixture was stirred until a fine suspension formed. After cooling with an ice-salt mixture to −5 °C, a solution of 1 mmol (254 mg) of iodine in 10 mL of dry THF was added dropwise. Subsequently, a solution of 2 mmol (324 mg) of dehydrothymol methyl ether (**18**) in 10 mL of dry THF was added over 10 min, keeping the temperature below 30 °C. The reaction mixture was stirred for 2 h at 25 °C, then cooled in an ice bath to 0 °C and mixed with 8 mL of water. To the mixture, 10 mL of THF and 10 mL of 3 M sodium hydroxide solution were added, followed by the dropwise addition of 4 mmol (136 mg, 0.45 mL) of 30% hydrogen peroxide with stirring [45]. Stirring was continued for 20 min after the addition was complete. The reaction mixture was transferred to a separatory funnel, and the reaction vessel was rinsed with 30 mL of diethyl ether. The aqueous phase was saturated with sodium chloride and extracted three times with 30 mL portions of diethyl ether. The combined organic phases were washed twice with 30 mL portions of saturated sodium chloride solution and dried over anhydrous magnesium sulfate. The solvent was removed under reduced pressure, and the residue was purified by column chromatography on silica gel using an *n*-hexane/Et_2_O mixture (4:1, *v*/*v*) as the eluent. The yield of 2-(2-methoxy-4-methylphenyl)propan-1-ol (**19**) was 200 mg (56%). The purity of the product was confirmed by TLC and GC–MS.

2-(2-Methoxy-4-methylphenyl)propan-1-ol (*syn*. 9-hydroxythymyl methyl ether (**19**)): colorless liquid; retention index (RI) = 1485 (DB-5MS column); UV (CH_3_CN) λ_max_(log ε) 281 (3.32), 275 (3.34), 221 (3.88), 200 (4.64) nm; FTIR (neat; cm^−1^) 3352, 2933, 2872, 2358, 2341, 1611, 1578, 1505, 1463, 1409, 1286, 1255, 1190, 1158, 1136, 1101, 1038, 1014, 976, 927, 846, 809, 714, 594; MS (EI), *m*/*z* (%) 180 (15), 150 (10), 149 (100), 134 (8), 119 (16), 117 (11), 115 (11), 105 (9), 91 (29), 77 (11); analyzed C 73.25, H 8.98, calculated for C_11_H_16_O_2_, C 73.30, H 8.95, O 17.75%; ^1^H NMR (CDCl_3_) δ 1.2410 (d, ^3^ *J*_8,10_ = 7.1, 3 H, CH_3_-10), 1.5521 (dd, ^3^ *J*_9A,OH_ = 0.5, ^3^ *J*_9B,OH_ = 0.5, 1 H, OH), 2.3376 (ddd, ^4^ *J*_6,7_ = −0.7, ^4^ *J*_2,7_ = −0.6, ^5^ *J*_5,7_ = 0.3, 3 H, CH_3_-7), 3.3846 (qdddd, ^3^ *J*_8,10_ = 7.1, ^3^ *J*_8,9A_ = 7.1, ^3^ *J*_8,9B_ = 6.1, ^4^ *J*_5,8_ = −0.5, ^5^ *J*_2,8_ = 0.4, ^5^ *J*_6,8_ = 0.4, 1 H, CH-8), 3.6713 (ddd, ^2^ *J*_9A,9B_ = −10.5, ^3^ *J*_8,9B_ = 6.1, ^3^ *J*_9B,OH_ = 0.5, 1 H, CH-9B), 3.7077 (ddd, ^2^ *J*_9A,9B_ = −10.5, ^3^ *J*_8,9A_ = 7.1, ^3^ *J*_9A,OH_ = 0.5, 1 H, CH-9A), 3.8137 (s, 3 H, CH_3_-11), 6.7008 (dqd, ^4^ *J*_2,6_ = 1.6, ^4^ *J*_2,7_ = −0.6, ^5^ *J*_2,8_ = 0.4, 1 H, CH-2), 6.7706 (ddqd, ^3^ *J*_5,6_ = 7.7, ^4^ *J*_2,6_ = 1.6, ^4^ *J*_6,7_ = −0.7, ^5^ *J*_6,8_ = 0.4, 1 H, CH-6), 7.0788 (ddq, ^3^ *J*_5,6_ = 7.7, ^4^ *J*_5,8_ = −0.5, ^5^ *J*_5,7_ = 0.3, 1 H, CH-5); ^13^C NMR (CDCl_3_) δ 16.77 (C-10), 21.56 (C-7), 35.08 (C-8), 55.49 (C-11), 68.08 (C-9), 111.73 (C-2), 121.49 (C-6), 127.26 (C-5), 128.78 (C-4), 137.47 (C-1), 157.33 (C-3).

### 3.13. Synthesis of 2-(2-Methoxy-4-methylphenyl)propan-1-ol Esters

Esters of 2-(2-methoxy-4-methylphenyl)propan-1-ol (**19**) with isobutyric and 2-methylbutyric acids were prepared in analogy to the synthesis of 2-methoxycuminyl esters [29]. The yield of 2-(2-methoxy-4-methylphenyl)propyl isobutyrate (**20**) was 95% (26.4 mg), and of 2-(2-methoxy-4-methylphenyl)propyl 2-methylbutyrate (**21**) was 91% (26.7 mg).

2-(2-Methoxy-4-methylphenyl)propyl isobutyrate (*syn*. 9-isobutyryloxythymyl methyl ether (**20**)): colorless liquid; retention index (RI) = 1716 (DB-5MS column); UV (CH_3_CN) λ_max_(log ε) 281 (3.32), 274 (3.33), 220 (3.88), 199 (4.64) nm; FTIR (neat; cm^−1^) 2969, 2934, 2874, 2363, 2341, 2042, 1991, 1974, 1731, 1612, 1580, 1507, 1465, 1411, 1389, 1342, 1287, 1259, 1190, 1157, 1096, 1072, 1041, 984, 928, 846, 809, 752, 713, 595; MS (EI): in complete agreement with the values published by Weremczuk-Jezyna et al., 2011 [30]; analyzed C 71.96, H 8.85, calculated for C_15_H_22_O_3_, C 71.97, H 8.86, O 19.17%; ^1^H NMR (CDCl_3_) δ 1.1174/1.1228 (d, ^3^ *J*_12,13/14_ = 7.0, 6 H, CH_3_-13 and CH_3_-14), 1.2548 (d, ^3^ *J*_8,10_ = 7.0, 3 H, CH_3_-10), 2.3289 (ddd, ^4^ *J*_6,7_ = −0.7, ^4^ *J*_2,7_ = −0.6, ^5^ *J*_5,7_ = 0.3, 3 H, CH_3_-7), 2.5054 (sept, ^3^ *J*_12,13/14_ = 7.0, 1 H, CH-12), 3.4845 (dqdddd, ^3^ *J*_8,9B_ = 7.7, ^3^ *J*_8,10_ = 7.0, ^3^ *J*_8,9A_ = 5.9, ^4^ *J*_5,8_ = −0.5, ^5^ *J*_2,8_ = 0.4, ^5^ *J*_6,8_ = 0.3, 1 H, CH-8), 3.8033 (s, 3 H, CH_3_-15), 4.1083 (dd, ^2^ *J*_9A,9B_ = −10.6, ^3^ *J*_8,9B_ = 7.7, 1 H, CH-9B), 4.1977 (dd, ^2^ *J*_9A,9B_ = −10.6, ^3^ *J*_8,9A_ = 5.9, 1 H, CH-9A), 6.6744 (dqd, ^4^ *J*_2,6_ = 1.6, ^4^ *J*_2,7_ = −0.6, ^5^ *J*_2,8_ = 0.4, 1 H, CH-2), 6.7352 (ddqd, ^3^ *J*_5,6_ = 7.7, ^4^ *J*_2,6_ = 1.6, ^4^ *J*_6,7_ = −0.7, ^5^ *J*_6,8_ = 0.3, 1 H, CH-6), 7.0569 (ddq, ^3^ *J*_5,6_ = 7.7, ^4^ *J*_5,8_ = −0.5, ^5^ *J*_5,7_ = 0.3, 1 H, CH-5); ^13^C NMR (CDCl_3_) δ 17.07 (C-10), 19.10/19.12 (C-13 and C-14), 21.57 (C-7), 31.98 (C-8), 34.20 (C-12), 55.39 (C-15), 68.39 (C-9), 111.55 (C-2), 121.16 (C-6), 127.35 (C-5), 128.30 (C-4), 137.45 (C-1), 157.12 (C-3), 177.28 (C-11).

2-(2-Methoxy-4-methylphenyl)propyl 2-methylbutyrate (*syn*. 9-(2-methylbutyryloxy)thymyl methyl ether (**21**)): colorless liquid; retention index (RI) = 1801 (DB-5MS column); UV (CH_3_CN) λ_max_(log ε) 281 (3.29), 274 (3.30), 220 (3.85), 199 (4.61) nm; FTIR (neat; cm^−1^) 2966, 2934, 2876, 2362, 2339, 1730, 1612, 1580, 1507, 1461, 1412, 1386, 1287, 1260, 1181, 1146, 1080, 1041, 1012, 979, 928, 846, 809, 751, 669, 595; MS (EI), in complete agreement with the values published by Weremczuk-Jezyna et al., 2011 [30]; analyzed C 72.70, H 9.12, calculated for C_16_H_24_O_3_, C 72.69, H 9.15, O 18.16%; ^1^H NMR (CDCl_3_) δ 0.8560/0.8460 (ddd, ^3^ *J*_13A,14_ = 7.5, ^3^ *J*_13B,14_ = 7.5, ^4^ *J*_12,14_ = 0.3, 3 H, CH_3_-14), 1.0962/1.0927 (d, ^3^ *J*_12,15_ = 7.0, 3 H, CH_3_-15), 1.2559 (d, ^3^ *J*_8,10_ = 7.0, 3 H, CH_3_-10), 1.4272/1.4199 (dqd, ^2^ *J*_13A,13B_ = −13.7, ^3^ *J*_13B,14_ = 7.5, ^3^ *J*_12,13B_ = 6.5, 1 H, CH-13B), 1.6333/1.6276 (ddq, ^2^ *J*_13A,13B_ = −13.7, ^3^ *J*_12,13A_ = 7.5, ^3^ *J*_13A,14_ = 7.5, 1 H, CH-13A), 2.3276 (ddd, ^4^ *J*_6,7_ = −0.7, ^4^ *J*_2,7_ = −0.6, ^5^ *J*_5,7_ = 0.3, 3 H, CH_3_-7), 2.3326/2.3288 (dqdq, ^3^ *J*_12,13A_ = 7.5, ^3^ *J*_12,15_ = 7.0, ^3^ *J*_12,13B_ = 6.5, ^4^ *J*_12,14_ = 0.3, 1 H, CH-12), 3.4820 (dqdddd, ^3^ *J*_8,9B_ = 7.7, ^3^ *J*_8,10_ = 7.0, ^3^ *J*_8,9A_ = 6.0, ^4^ *J*_5,8_ = −0.5, ^5^ *J*_2,8_ = 0.4, ^5^ *J*_6,8_ = 0.3, 1 H, CH-8), 3.8032 (s, 3 H, CH_3_-16), 4.1167/4.1099 (dd, ^2^ *J*_9A,9B_ = −10.6, ^3^ *J*_8,9B_ = 7.7, 1 H, CH-9B), 4.2219/4.2078 (dd, ^2^ *J*_9A,9B_ = −10.6, ^3^ *J*_8,9A_ = 6.0, 1 H, CH-9A), 6.6729 (dqd, ^4^ *J*_2,6_ = 1.6, ^4^ *J*_2,7_ = −0.6, ^5^ *J*_2,8_ = 0.4, 1 H, CH-2), 6.7331 (ddqd, ^3^ *J*_5,6_ = 7.7, ^4^ *J*_2,6_ = 1.6, ^4^ *J*_6,7_ = −0.7, ^5^ *J*_6,8_ = 0.3, 1 H, CH-6), 7.0559 (ddq, ^3^ *J*_5,6_ = 7.7, ^4^ *J*_5,8_ = −0.5, ^5^ *J*_5,7_ = 0.3, 1 H, CH-5); ^13^C NMR (CDCl_3_) δ 11.72/11.69 (C-14), 16.72 (C-15), 17.16/17.14 (C-10), 21.56 (C-7), 26.87/26.85 (C-13), 32.02 (C-8), 41.29/41.27 (C-12), 55.39 (C-16), 68.31 (C-9), 111.55 (C-2), 121.17 (C-6), 127.37 (C-5), 128.33/128.31 (C-4), 137.44 (C-1), 157.12 (C-3), 176.89 (C-11).

### 3.14. Synthesis of 4-Isopropyl-3-methoxybenzaldehyde (Syn. 3-Methoxycuminaldehyde (***24***))

A solution of 3-methoxycuminol (**22**) (50 mg, 0.28 mmol) and PCC (90 mg, 0.42 mmol) in 20 mL of dry CH_2_Cl_2_ was stirred at room temperature for 2 h [46]. Then, the mixture was filtered over silica gel, and the filtrate was concentrated under reduced pressure. The yield of 4-isopropyl-3-methoxybenzaldehyde (**24**) (40 mg) was 81%.

4-Isopropyl-3-methoxybenzaldehyde (*syn*. 3-methoxycuminaldehyde (**24**)): yellowish liquid; retention index (RI) = 1435 (DB-5MS column); UV (CH_3_CN) λ_max_(log ε) 310 (3.57), 262 (4.02), 222 (4.22), 200 (4.08) nm; FTIR (neat; cm^−1^) 2960, 2927, 2870, 2722, 2364, 2338, 1687, 1602, 1577, 1497, 1461, 1419, 1386, 1361, 1300, 1285, 1257, 1190, 1151, 1115, 1091, 1061, 1037, 964, 866, 820, 780, 734, 627, 593, 565; MS (EI), in complete agreement with the values published by Warning et al., 1988 [47]; analyzed C 74.14, H 7.93, calculated for C_11_H_14_O_2_, C 74.13, H 7.92, O 17.95%; ^1^H NMR (CDCl_3_) δ 1.2329 (d, ^3^ *J*_8,9/10_ = 6.9, 6 H, CH_3_-9 and CH_3_-10), 3.3799 (septddd, ^3^ *J*_8,9/10_ = 6.9, ^4^ *J*_5,8_ = −0.5, ^5^ *J*_2,8_ = 0.3, ^5^ *J*_6,8_ = 0.3, 1 H, CH-8), 3.8997 (s, 3 H, CH_3-_11), 7.3568 (dddd, ^4^ *J*_2,6_ = 1.6, ^5^ *J*_2,5_ = 0.3, ^4^ *J*_2,7_ = −0.3, ^5^ *J*_2,8_ = 0.3, 1 H, CH-2), 7.3752 (ddd, ^3^ *J*_5,6_ = 7.7, ^4^ *J*_5,8_ = −0.5, ^5^ *J*_2,5_ = 0.3, 1 H, CH-5), 7.4301 (dddd, ^3^ *J*_5,6_ = 7.7, ^4^ *J*_2,6_ = 1.6, ^4^ *J*_6,7_ = −0.3, ^5^ *J*_6,8_ = 0.3, 1 H, CH-6), 9.9366 (dd, ^4^ *J*_2,7_ = −0.3, ^4^ *J*_6,7_ = −0.3, 1 H, CH-7); ^13^C NMR (CDCl_3_) δ 22.45 (C-9, and C-10), 27.32 (C-8), 55.63 (C-11), 108.47 (C-2), 124.92 (C-6), 126.52 (C-5), 135.54 (C-1), 145.06 (C-4), 157.47 (C-3), 192.19 (C-7).

### 3.15. Synthesis of Methyl-4-isopropyl-3-methoxybenzoate (Syn. Methyl 3-Methoxycuminate (***23***))

#### 3.15.1. Oxidation of 3-Methoxycuminol with the Jones Reagent

Chromium trioxide (2.5 g, 25 mmol) was dissolved in 7.5 mL water in an Erlenmeyer flask, and concentrated sulfuric acid (2.5 mL) was added slowly with careful stirring in an ice/water bath. The compound 3-methoxycuminol (**22**) (33 mg, 0.18 mmol) was dissolved in 10 mL acetone, and to this solution was added dropwise the Jones reagent (0.1 mL, 0.25 mmol) at 0 °C [48]. After being stirred for 2 h at room temperature, the mixture was diluted with water, and most of the acetone was evaporated under reduced pressure. The residue was extracted with diethyl ether, and the combined organic layers were washed with a saturated aqueous NaHCO_3_ solution. The combined aqueous layers were acidified with a 2 M aqueous HCl solution to pH 3 and then extracted again with diethyl ether. The extract was successively washed with water and brine, dried (MgSO_4_), and concentrated under reduced pressure. The yield of 4-isopropyl-3-methoxybenzoic acid (28 mg) was 79%.

4-Isopropyl-3-methoxybenzoic acid (*syn*. 3-methoxycumic acid): white solid; analyzed C 68.04, H 7.26, calculated for C_11_H_14_O_3_, C 68.02, H 7.27, O 24.71%; ^1^H NMR (CDCl_3_) δ 1.2315 (d, ^3^ *J*_8,9/10_ = 6.9, 6 H, CH_3_-9 and CH_3_-10), 3.3770 (septddd, ^3^ *J*_8,9/10_ = 6.9, ^5^ *J*_2,8_ = 0.5, ^4^ *J*_5,8_ = −0.5, ^5^ *J*_6,8_ = 0.5, 1 H, CH-8), 3.9016 (s, 3 H, CH_3-_11), 7.3036 (ddd, ^3^ *J*_5,6_ = 7.9, ^4^ *J*_5,8_ = −0.5, ^5^ *J*_2,5_ = 0.3, 1 H, CH-5), 7.5560 (ddd, ^4^ *J*_2,6_ = 1.6, ^5^ *J*_2,8_ = 0.5, ^5^ *J*_2,5_ = 0.3, 1 H, CH-2), 7.7109 (ddd, ^3^ *J*_5,6_ = 7.9, ^4^ *J*_2,6_ = 1.6, ^5^ *J*_6,8_ = 0.5, 1 H, CH-6), 11.5910 (brs, 1 H, COOH); ^13^C NMR (CDCl_3_) δ 22.51 (C-9, and C-10), 27.19 (C-8), 55.66 (C-11), 111.43 (C-2), 123.13 (C-6), 126.18 (C-5), 127.65 (C-1), 143.89 (C-4), 156.84 (C-3), 171.73 (C-7).

#### 3.15.2. Esterification of 4-Isopropyl-3-methoxybenzoic Acid with Diazomethane

The solution of 4-isopropyl-3-methoxybenzoic acid (28 mg, 0.15 mmol) in diethyl ether was treated with a solution of diazomethane dropwise until the evolution of gas ceased [49]. After that, diethyl ether was removed under reduced pressure. The yield of methyl 4-isopropyl-3-methoxybenzoate (**23**) (30 mg) was quantitative.

Methyl 4-isopropyl-3-methoxybenzoate (*syn*. methyl 3-methoxycuminate (**23**)): yelowish liquid; retention index (RI) = 1564 (DB-5MS column); UV (CH_3_CN) λ_max_(log ε) 294 (2.60), 246 (3.07), 212 (3.46) nm; FTIR (neat; cm^−1^) 2958, 2870, 1719, 1637, 1608, 1578, 1502, 1451, 1434, 1409, 1351, 1288, 1268, 1227, 1190, 1176, 1106, 1091, 1037, 901, 899, 874, 837, 786, 765, 744, 630; MS (EI), *m*/*z* (%) 208 (31), 194 (12), 193 (100), 177 (10), 131 (8), 115 (8), 103 (9), 91 (22), 77 (8), 59 (8); analyzed C 69.22, H 7.75, calculated for C_12_H_16_O_3_, C 69.21, H 7.74, O 23.05%; ^1^H NMR (CDCl_3_) δ 1.2161 (d, ^3^ *J*_8,9/10_ = 6.9, 6 H, CH_3_-9 and CH_3_-10), 3.3523 (septddd, ^3^ *J*_8,9/10_ = 6.9, ^4^ *J*_5,8_ = −0.5, ^5^*J*_6,8_ = 0.5, ^5^ *J*_2,8_ = 0.5, 1 H, CH-8), 3.8837 (s, 3 H, CH_3-_12), 3.9067 (s, 3 H, CH_3-_11), 7.2595 (ddd, ^3^ *J*_5,6_ = 7.9, ^4^ *J*_5,8_ = −0.5, ^5^ *J*_2,5_ = 0.3, 1 H, CH-5), 7.4988 (ddd, ^4^ *J*_2,6_ = 1.6, ^5^ *J*_2,8_ = 0.5, ^5^ *J*_2,5_ = 0.3, 1 H, CH-2), 7.6186 (ddd, ^3^ *J*_5,6_ = 7.9, ^4^ *J*_2,6_ = 1.6, ^5^ *J*_6,8_ = 0.5, 1 H, CH-6); ^13^C NMR (CDCl_3_) δ 22.53 (C-9, and C-10), 27.09 (C-8), 52.19 (C-11), 55.64 (C-12), 111.06 (C-2), 122.32 (C-6), 126.02 (C-5), 128.63 (C-1), 142.79 (C-4), 156.76 (C-3), 167.38 (C-7).

## 4. Conclusions

In conclusion, the essential oil of *Doronicum columnae* roots was subjected to comprehensive chemical analysis, resulting in the identification of 83 components, representing 98.1% of the total composition. Oxygenated monoterpenoids and sesquiterpene hydrocarbons emerged as the predominant classes, with thymyl isobutyrate and thymyl 2-methylbutyrate identified as the principal constituents. In this study, our main focus was on a less abundant class of related and isomeric compounds that proved impossible to be distinguished by means of mass spectra or retention indices alone. These were, namely, a group of multiply oxygenated aromatic isomers related to *p*-cymene, thymol, and carvacrol—the dominant constituents of the essential oil. To identify as many essential oil constituents as possible in such a complex mixture, we opted for a multifaceted approach, starting from reasoning based on biosynthetic relationships and structure-retention index correlations, followed by chromatographic separation. After obtaining a fraction rich in the unknown constituents, detailed GC-MS and NMR analyses hinted towards the presence of certain structural motifs, which guided our synthesis efforts. As a result, a small synthetic library of esters of 2-methoxycuminol, 3-methoxycuminol, 6-methoxythymol, 6-hydroxythymyl methyl ether, and 9-hydroxythymyl methyl ether was prepared (12 compounds in total). Among them, the esters of 2-methoxycuminol (isobutyrate (**3**), 2-methylbutyrate (**4**), and isovalerate (**5**)), 6-methoxythymyl 2-methylbutyrate (**13**), and 6-(2-methylbutyryloxy)thymyl methyl ether (**15**) represent newly identified, synthesized, and spectrally characterized natural products. Additionally, methyl 3-methoxycuminate (**24**), which is another new natural product, was tentatively identified through detailed NMR analysis of an essential oil fraction and later definitively confirmed as a constituent by GC co-chromatography of the synthesized standard with an essential oil sample. Looking ahead, it seems that the investigation or reanalysis of *Doronicum* species using the proposed methodology has great potential, especially considering the critical role of minor constituents in essential oil chemistry and pharmacology. The results of this study further expand the already significant molecular diversity of *Doronicum* secondary metabolites and related compounds. It would not be surprising if future research on such essential oils returns further new oxygenated aromatic derivatives and other distinctive compounds.

## Figures and Tables

**Table 1:** Chemical composition of the essential oil of *Doronicum columnae* Ten. roots from Serbia.

No ^a^	RI ^b^	RI ^c^	Constituents	C ^d^	% ^e^	ID ^f^
1	938	1022	*α*-Pinene	MH	tr	MS, RI, CoI
2	950	1066	Camphene	MH	tr	MS, RI, CoI
3	969	1123	Sabinene	MH	tr	MS, RI, CoI
4	978	1109	*β*-Pinene	MH	0.1	MS, RI, CoI
5	1005	1167	*α*-Phellandrene	MH	tr	MS, RI, CoI
6	1019	1418	4-Methylanisole	O	tr	MS, RI, CoI
7	1022	1274	*p*-Cymene	MH	0.7	MS, RI, CoI
8	1026	1209	1,8-Cineole	MO	tr	MS, RI, CoI
9	1026	1202	Limonene	MH	tr	MS, RI, CoI
10	1035	1227	(*Z*)-*β*-Ocimene	MH	tr	MS, RI, CoI
11	1044	1250	(*E*)-*β*-Ocimene	MH	tr	MS, RI
12	1054	1248	*γ*-Terpinene	MH	tr	MS, RI, CoI
13	1100	1100	Undecane	FAD	tr	MS, RI, CoI
14	1112	1444	*β*-Thujone	MO	tr	MS, RI, CoI
15	1124	1615	*cis*-*p*-Menth-2-en-1-ol	MO	0.1	MS, RI, CoI
16	1137	1680	*cis*-*p*-Menth-2,8-dien-1-ol	MO	tr	MS, RI
17	1141	1518	Camphor	MO	tr	MS, RI, CoI
18	1142	1568	*trans*-*p*-Menth-2-en-1-ol	MO	tr	MS, RI
19	1146	1514	(*Z*)-2-Nonenal	FAD	tr	MS, RI
20	1178	1821	*trans*-2-Caren-4-ol	MO	tr	MS, RI
21	1179	1608	Terpinen-4-ol	MO	tr	MS, RI, CoI
22	1190	1860	*p*-Cymen-8-ol	MO	tr	MS, RI, CoI
23	1199	1753	*cis*-Piperitol	MO	tr	MS, RI
24	1200	1200	Dodecane	FAD	tr	MS, RI, CoI
25	1221	1961	8,9-Dehydrothymol	MO	tr	MS, RI, CoI
26	1231	1585	2-Isopropyl-4-methylanisole (*syn*. isothymyl methyl ether)	MO	12.0	MS, RI, CoI
27	1236	1600	Thymyl methyl ether	MO	4.5	MS, RI, CoI
28	1246	1612	Carvacryl methyl ether	MO	1.9	MS, RI, CoI
29	1252	1685	8,9-Dehydrothymyl methyl ether	MO	tr	MS, RI, CoI
30	1255	1557	Linalyl acetate	MO	tr	MS, RI
31	1259	1870	*trans*-Myrtanol	MO	tr	MS, RI
32	1286	2187	2-Isopropyl-4-methylphenol (*syn*. isothymol)	MO	12.7	MS, RI, CoI
33	1292	2203	Thymol	MO	6.9	MS, RI, CoI
34	1303	2232	Carvacrol	MO	tr	MS, RI, CoI
35	1331	1449	Silphiperfol-5-ene	SH	tr	MS, RI
36	1338	1405	Presilphiperfol-7-ene	SH	0.1	MS, RI
37	1349	1422	7-*epi*-silphiperfol-5-ene	SH	0.1	MS, RI
38	1355	1468	Silphin-1-ene	SH	tr	MS, RI
39	1359	1465	α-Longipinene	SH	tr	MS, RI
40	1376	1480	Cyclosativene	SH	tr	MS, RI
41	1380	1491	α-Copaene	SH	tr	MS, RI
42	1382	1495	Silphiperfol-6-ene	SH	tr	MS, RI
43	1391	1520	Modheph-2-ene	SH	0.1	MS, RI
44	1394	1534	α-Isocomene	SH	0.4	MS, RI
45	1403	1526	Petasitene	SH	tr	MS, RI
46	1410	1529	α-Gurjunene	SH	tr	MS, RI
47	1415	1549	*β*-Isocomene	SH	0.1	MS, RI
48	1423	1596	(*E*)-Caryophyllene	SH	0.1	MS, RI, CoI
49	1426	1878	2,5-Dimethoxy-*p*-cymene	MO	0.1	MS, RI, CoI
50	1435	2062	3-Methoxycuminaldehyde	MO	tr	MS, CoI
51	1445	1915	Thymyl propionate	MO	0.1	MS, RI, CoI
52	1450	1651	*cis*-*β*-Santalene	SH	tr	MS, RI
53	1456	1665	*α*-Humulene	SH	tr	MS, RI
54	1458	1673	(*E*)-*β*-Farnesene	SH	tr	MS, RI
55	1483	1948	8,9-Dehydrothymyl isobutyrate	MO	tr	MS, RI, CoI
56	1485	1686	*α*-Muurolene	SH	tr	MS, RI
57	1486	1908	Thymyl isobutyrate	MO	32.8	MS, RI, CoI
58	1492	1722	*β*-Selinene	SH	1.2	MS, RI
59	1500	1734	Bicyclogermacrene	SH	tr	MS, RI
60	1515	1944	Carvacryl isobutyrate	MO	0.3	MS, RI, CoI
61	1534	1892	10-*epi*-Cubebol	SO	tr	MS, RI
62	1545	1778	(*E*)-*α*-Bisabolene	SH	0.1	MS, RI
63	1564	2159	Methyl 3-methoxycuminate *	MO	tr	MS, RI, CoI
64	1565	1975	Longipinanol	SO	tr	MS, RI
65	1576	2036	8,9-Dehydrothymyl 2-methylbutyrate	MO	tr	MS, RI, CoI
66	1578	1994	Thymyl 2-methylbutyrate	MO	22.8	MS, RI, CoI
67	1581	2007	Thymyl isovalerate	MO	tr	MS, RI, CoI
68	1590	1982	Caryophyllene oxide	SO	0.1	MS, RI
69	1607	2031	Carvacryl 2-methylbutyrate	MO	0.2	MS, RI, CoI
70	1610	2072	*β*-Oplopenone	SO	tr	MS, RI
71	1621	2069	1,10-di-*epi*-Cubenol	SO	tr	MS, RI
72	1691	2212	6-Methoxythymyl isobutyrate	MO	tr	MS, RI, CoI
73	1711	2248	6-Isobutyryloxythymyl methyl ether	MO	tr	MS, RI, CoI
74	1716	2260	9-Isobutyryloxythymyl methyl ether	MO	tr	MS, RI, CoI
75	1723	2278	3-Methoxycuminyl isobutyrate	MO	0.3	MS, RI, CoI
76	1750	2322	2-Methoxycuminyl isobutyrate *	MO	tr	MS, RI, CoI
77	1783	2299	6-Methoxythymyl 2-methylbutyrate *	MO	tr	MS, RI, CoI
78	1801	2350	9-(2-Methylbutyryloxy)thymyl methyl ether	MO	tr	MS, RI, CoI
79	1803	2335	6-(2-Methylbutyryloxy)thymyl methyl ether *	MO	tr	MS, RI, CoI
80	1808	2356	3-Methoxycuminyl 2-methylbutyrate	MO	0.3	MS, RI, CoI
81	1817	2381	3-Methoxycuminyl isovalerate	MO	tr	MS, RI, CoI
82	1835	2402	2-Methoxycuminyl 2-methylbutyrate *	MO	tr	MS, RI, CoI
83	1845	2429	2-Methoxycuminyl isovalerate *	MO	tr	MS, RI, CoI
			Total identified [%]		98.1	
			Fatty acid and fatty acid-related compounds	FAD	tr	
			Monoterpene hydrocarbons	MH	0.8	
			Oxygenated monoterpenes	MO	95.0	
			Others	O	tr	
			Sesquiterpene hydrocarbons	SH	2.2	
			Oxygenated sesquiterpenes	SO	0.1	

^a^ Order of elution on DB-5MS column. ^b^ Retention indices determined relative to a homologous series of *n*-alkanes (C_9_–C_19_) on a non-polar DB-5MS column. ^c^ RI Experimental retention indices determined relative to a homologous series of *n*-alkanes (C_10_–C_25_) on a polar HP-Innowax GC column. ^d^ C = Class; for compound class abbreviations, cf. last rows of this table. ^e^ %: tr = trace amounts (<0.05%). ^f^ ID = identification method; MS = constituent identified by mass-spectra comparison with those listed in Wiley 11, NIST17 [28], MassFinder 2.3, and a homemade mass spectral library; RI = constituent identified by retention index matching with literature data; CoI = constituent identity confirmed by GC co-injection of an authentic sample. * New natural product and entirely new compound.

## Data Availability

Data are contained within the article and supplementary materials.

## Supplementary Materials

The following supporting information can be downloaded at: https://www.mdpi.com/article/10.3390/molecules30020302/s1, Figures S1–S96 and Tables S1–S23.

## References

[1] Badalamenti N., Modica A., Ilardi V., Bruno M. (2021). Chemical Constituents and Biological Properties of Genus *Doronicum* (Asteraceae). Chem. Biodivers..

[2] Josifović M. (1975). Flora SR Srbije.

[3] Shaimerdenova Z.R., Makubaeva A.I., Ozek T., Ozek G., Atazhanova G.A., Adekenov S.M. (2019). Volatile Constituents of *Doronicum altaicum*. Chem. Nat. Compd..

[4] Lazarević J., Radulović N., Palić R., Zlatković B. (2019). Chemical Composition of the Essential Oil of *Doronicum austriacum* Jacq. Subsp. *giganteum* (Griseb) Stoj. Et Stef. (Compositae) from Serbia. J. Essent. Oil Res..

[5] Akpinar K., Yildirim N., Ucuncu O., Yayli N., Terziogly S., Yayli N. (2009). Volatile Constituents of the Flowers and Leaves-Stems of Three *Doronicum* Taxa from Turkey. Asian J. Chem..

[6] Paolini J., Muselli A., Bernardini A.F., Bighelli A., Casanova J., Costa J. (2007). Thymol Derivatives from Essential Oil of *Doronicum corsicum* L. Flavour Fragr. J..

[7] Özcan K. (2020). Antibacterial, Antioxidant and Enzyme Inhibition Activity Capacities of *Doronicum macrolepis* (Freyn & Sint): An Endemic Plant from Turkey. Saudi Pharm. J..

[8] David J.R., Heywood V.H., Harborne J.B., Turner B.L. (1977). The Biology and Chemistry of the Compositae. The Biology and Chemistry of the Compositae.

[9] Bohlmann F., Zdero C. (1972). Neue Thymol-Derivate aus *Arnica amplexicaulis*. Tetrahedron Lett..

[10] Willuhn G., Junior I., Wendisch D. (1986). Desmethoxy-encecalin and Thymol Derivative from *Arnica sachalinensis*. Planta Med..

[11] Passreiter C.M., Matthiesen U., Willuhn G. (1998). 10-Acetoxy-9-chloro-8,9-dehydrothymol and Further Thymol Derivatives from *Arnica sachalinensis*. Phytochemistry.

[12] Passreiter C.M., Florack M., Willuhn G., Goerz G. (1988). Allergic Contact Dermatitis Caused by Asteraceae. Identification of an 8,9-Epoxythymol-Diester as the Contact Allergen of *Arnica sachalinensis*. Derm. Beruf Umwelt..

[13] Mączka W., Twardawska M., Grabarczyk M., Wińska K. (2023). Carvacrol—A Natural Phenolic Compound with Antimicrobial Properties. Antibiotics.

[14] Bohlmann F., Zdero C. (1970). Neue Benzofuranderivate aus *Doronicum austriacum* Jacq. Tetrahedron Lett..

[15] Bohlmann F., Dhar A.K., Ahmed M. (1980). Thymol Derivatives from *Doronicum hungaricum*. Phytochemistry.

[16] Bohlmann F., Grenz M. (1979). Neue Tremeton-derivate aus *Doronicum macrophyllum*. Phytochemistry.

[17] Bohlmann F., Abraham W.R. (1979). Ein neuer Sesquiterpenalkohol und andere Inhaltsstoffe aus *Doronicum pardalianches*. Phytochemistry.

[18] Reynaud J., Becchi M., Raynaud J. (1985). *p*-Hydroxyacetophenone Derivatives from *Doronicum grandiflorum*. J. Nat. Prod..

[19] Clarke C.B., Yadava R.N., Patil G. (2013). Isolation and Characterization of a New Allelochemical from the Flower of *Doronicum hookeri*. Res. J. Chem. Environ..

[20] Alieva S.A., Omurkamzinova V.B., Glyzin V.I. (1979). Flavonoids of *Doronicum macrophyllum* and *D. oblongifolium*. Chem. Nat. Compd..

[21] Reynaud J., Raynaud J. (1986). Les Flavonoïdes de *Doronicum grandiflorum*. Biochem. Syst. Ecol..

[22] Arumugam R., Sarikurkcu C., Ozer M.S. (2021). Comparison of Methanolic Extracts of *Doronicum orientale* and *Echium angustifolium* in Terms of Chemical Composition and Antioxidant Activities. Biocatal. Agric. Biotechnol..

[23] Šilić Č. (1990). Šumske Zeljaste Biljke.

[24] Mroczek T., Glowniak K., Wlaszcszyk A. (2002). Simultaneous Determination of *N*-Oxides and Free Bases of Pyrrolizidine Alkaloids by Cation-Exchange Solid-Phase Extraction and Ion-Pair High-Performance Liquid Chromatography. J. Chromatogr. A.

[25] Stojanovic G.S., Mitic V.D., Stankov-Jovanovic V.P., Ilic M.D., Jovanovic O.P., Petrovic G.M. (2013). Antioxidant Characteristics of Selected Plant Species Growing under Post-Fire Environmental Conditions. Oxid. Commun..

[26] Markovic M.S., Ilic B.S., Miladinovic D.L., Stamenkovic S.M., Trajkovic R., Stankov-Jovanovic V.P., Djelic G.T. (2015). Activity of a Catalase Enzyme in Plants from the Burned Areas of the Vidlic Mountain Beech Forest. Oxid. Commun..

[27] Cerutti-Delasalle C., Mehiri M., Cagliero C., Rubiolo P., Bicchi C., Meierhenrich U.J., Baldovini N. (2016). The (+)-*cis*- and (+)-*trans*-Olibanic Acids: Key Odorants of Frankincense. Angew. Chem. Int. Ed..

[28] (2017). Mass Spectral Library (NIST/EPA/NIH).

[29] Radulović N.S., Mladenović M.Z., Vukićević D.R., Stojanović N.M., Randjelović P.J., Stojanović-Radić Z.Z., Boylan F. (2022). *Pulicaria dysenterica* (L.) Bernh.—Rightfully Earned Name? Identification and Biological Activity of New 3-Methoxycuminyl Esters from *P. dysenterica* Essential Oil. Plants.

[30] Weremczuk-Jeżyna I., Wysokińska H., Kalemba D. (2011). Constituents of the Essential Oil from Hairy Roots and Plant Roots of *Arnica montana* L. J. Essent. Oil Res..

[31] Weyerstahl P., Marschall H., Seelmann I., Kaul V.K. (1997). Constituents of the Flower Essential Oil of *Ageratina adenophora* (Spreng.) K. et R. from India. Flavour Fragr. J..

[32] Mladenović M.Z., Radulović N.S. (2017). The Essential Oil of *Achillea ageratifolia* (Sm.) Boiss. subsp. *serbica* (Nyman) Heimerl (Asteraceae) Revisited: The Stereochemical Nomenclature Issues, Structural Elucidation, and Synthesis of (New) Sabinyl Esters. Flavour Fragr. J..

[33] Radulović N.S., Filipović S.I., Nešić M.S., Stojanović N.M., Mitić K.V., Mladenović M.Z., Ranđelović V.N. (2020). Immunomodulatory Constituents of *Conocephalum conicum* (Snake Liverwort) and the Relationship of Isolepidozenes to Germacranes and Humulanes. J. Nat. Prod..

[34] Schreiner L., Bauer J., Ortner E., Buettner A. (2020). Structure-Odor Activity Studies on Derivatives of Aromatic and Oxygenated Monoterpenoids Synthesized by Modifying *p*-Cymene. J. Nat. Prod..

[35] Wheeler O.H. (1958). Etard Reaction: I. Its Scope and Limitation with Substituted Toluenes. Can. J. Chem..

[36] Kremers E., Wakeman N., Hixon R.M. (1926). Thymoquinone. Org. Synth..

[37] Sumerford W.T., Dalton D.N. (1944). The Hydrolysis of Some Quinone Oximes. J. Am. Chem. Soc..

[38] Bohlmann F., Mahanta P.K., Jakupovic J., Rastogi R.C., Nath A.A. (1978). New Sesquiterpene Lactones from *Inula* Species. Phytochemistry.

[39] Weyerstahl P., Wahlburg H.-C., Marschall H., Rustaiyan A. (1993). New Cadinene and Bisabolene Derivatives from the Essential Oil of *Pulicaria gnaphalodes*. Liebigs Ann. Chem..

[40] Rosenmund K.W., Schnurr W. (1928). Über Acylwanderungen an Phenolen. Liebigs Ann. Chem..

[41] Montiel L.E., Zepeda L.G., Tamariz J.N. (2010). Efficient Total Synthesis of Racemic Bisabolane Sesquiterpenes Curcuphenol and Xanthorrhizol Starting from Substituted Acetophenones. Helv. Chim. Acta.

[42] Bestmann H.J. (1962). Reaktionen mit Phosphin-alkylenen, I. Intermolekulare Umylidierung zwischen Phosphoniumsalzen und Phosphin-alkylenen. Chem. Ber..

[43] Ohmura T., Kusaka S., Torigoe T., Suginome M. (2019). Iridium-Catalyzed C(sp^3^)−H Addition of Methyl Ethers across Intramolecular Carbon–Carbon Double Bonds Giving 2,3-Dihydrobenzofurans. Adv. Synth. Catal..

[44] Du Z.-T., Li A.-P., Wu T.-X., Peng K., Pan X.-F. (2004). Facile Synthesis of (±)-Parahigginone Methyl Ether and (±)-Curcuphenol. J. Chin. Chem. Soc..

[45] Prasad A.S.B., Kanth J.V.B., Periasamy M. (1992). Convenient Methods for the Reduction of Amides, Nitriles, Carboxylic Esters, Acids and Hydroboration of Alkenes Using NaBH_4_/I_2_ System. Tetrahedron.

[46] Corey E.J., Suggs J.W. (1975). Pyridinium Chlorochromate. An Efficient Reagent for Oxidation of Primary and Secondary Alcohols to Carbonyl Compounds. Tetrahedron Lett..

[47] Warning U., Bohlmann F., King R.M., Haegi L. (1988). Diterpenes from *Olearia* Species. J. Nat. Prod..

[48] Nakahata T., Itagaki N., Arai T., Sugie H., Kuwahara S. (2003). Synthesis of the Sex Pheromone of the Citrus Mealybug, *Pseudococcus cryptus*. Biosci. Biotechnol. Biochem..

[49] Lee H.J., Ravn M.R., Coates R.M. (2001). Synthesis and characterization of abietadiene, levopimaradiene, palustradiene, and neoabietadiene: Hydrocarbon precursors of the abietane diterpene resin acids. Tetrahedron.

