# Supplementary material for: New Oxygenated Methoxy-p-Cymene Derivatives from Leopard’s Bane (Doronicum columnae Ten., Asteraceae) Essential Oil: Synthesis Facilitating the Identification of Isomeric Minor Constituents in Complex Matrices
Source: Molecules. 2025 Jan 14;30(2):302. doi: 10.3390/molecules30020302 (PMC11767694; doi:10.3390/molecules30020302)
Supplement: Supplementary file 1 [file molecules-30-00302-s001.zip › molecules-3405229-supplementary.pdf]

Supplementary material

for

New Oxygenated Methoxy-*p*-Cymene Derivatives from Leopard's Bane (*Doronicum columnae* Ten., Asteraceae) Essential Oil: Synthesis Facilitating the Identification of Isomeric Minor Constituents in Complex Matrices

Milan Ž. Dimitrijević [1], Marko Z. Mladenović [1,2], Milica D. Nešić [1], Milan S. Dekić [2], Vidak N. Raičević [3], Niko S. Radulović [1,*]

[1] Department of Chemistry, Faculty of Sciences and Mathematics, University of Niš, Višegradska 33, 18000 Niš, Serbia
[2] Department of Chemical and Technological Sciences, State University of Novi Pazar, Vuka Karadžića bb, 36300 Novi Pazar, Serbia
[3] Faculty of Medicine, University of Novi Sad, Hajduk Veljkova 3, 21000 Novi Sad, Serbia
* Correspondence: nikoradulovic@yahoo.com; Tel.: +381-18-533-015; Fax: +381-18-533-014

Content:

Figure S1. Part of the chromatogram of the essential oil fraction (colored in black) and patrial ion current chromatograms (ion at *m/z* 163, 179, 165, and 162 (colored in red, blue, brown, and green, respectively))
Figure S2. Mass spectrum (EI, 70 eV) of 3-methoxycuminyl isobutyrate
Figure S3. ^{1}H NMR (400 MHz, $CDCl_3$) spectrum of 3-methoxycuminyl isobutyrate and the corresponding expansions with signal assignment
Figure S4. Simulated (manual iterative full spin, MestreNova 11.0.3) and experimental ^{1}H NMR spectrum of 3-methoxycuminyl isobutyrate
Figure S5. ^{13}C NMR (100.6 MHz, $CDCl_3$) spectrum of 3-methoxycuminyl isobutyrate
Table S1. ^{1}H (400 MHz) and ^{13}C (100.6 MHz) NMR data of 3-methoxycuminyl isobutyrate (chloroform-*d*), NMR parameters are derived from manual iterative full spin analysis, along with the observed gHMBC and NOESY correlations
Figure S6. NOESY (blue arrows) and HMBC (red arrows) interactions of 3-methoxycuminyl isobutyrate
Figure S7. Mass spectrum (EI, 70 eV) of 3-methoxycuminyl 2-methylbutyrate
Figure S8. ^{1}H NMR (400 MHz, $CDCl_3$) spectrum of 3-methoxycuminyl 2-methylbutyrate and the corresponding expansions with signal assignment
Figure S9. Simulated (manual iterative full spin, MestreNova 11.0.3) and experimental ^{1}H NMR spectrum of 3-methoxycuminyl 2-methylbutyrate
Figure S10. ^{13}C NMR (100.6 MHz, $CDCl_3$) spectrum of 3-methoxycuminyl 2-methylbutyrate
Table S2. ^{1}H (400 MHz) and ^{13}C (100.6 MHz) NMR data of 3-methoxycuminyl 2-methylbutyrate (chloroform-*d*), NMR parameters are derived from manual iterative full spin analysis, along with the observed gHMBC and NOESY correlations
Figure S11. NOESY (blue arrows) and HMBC (red arrows) interactions of 3-methoxycuminyl 2-methylbutyrate

Figure S12. Mass spectrum (EI, 70 eV) of 3-methoxycuminyl isovalerate
Figure S13. ^{1}H NMR (400 MHz, $CDCl_3$) spectrum of 3-methoxycuminyl isovalerate and the corresponding expansions with signal assignment
Figure S14. Simulated (manual iterative full spin, MestreNova 11.0.3) and experimental ^{1}H NMR spectrum of 3-methoxycuminyl isovalerate
Figure S15. ^{13}C NMR (100.6 MHz, $CDCl_3$) spectrum of 3-methoxycuminyl isovalerate
Table S3. ^{1}H (400 MHz) and ^{13}C (100.6 MHz) NMR data of 3-methoxycuminyl isovalerate (chloroform-*d*), NMR parameters are derived from manual iterative full spin analysis, along with the observed gHMBC and NOESY correlations
Figure S16. NOESY (blue arrows) and HMBC (red arrows) interactions of 3-methoxycuminyl isovalerate
Table S4. ^{1}H (400 MHz) and ^{13}C (100.6 MHz) NMR data of 2-methoxycuminaldehyde (**2**) (chloroform-*d*), NMR parameters are derived from manual iterative full spin analysis, along with the observed gHMBC and NOESY correlations
Figure S17. NOESY (blue arrows) and HMBC (red arrows) interactions of 2-methoxycuminaldehyde (**2**)
Table S5. ^{1}H (400 MHz) and ^{13}C (100.6 MHz) NMR data of 2-methoxycuminol (chloroform-*d*), NMR parameters are derived from manual iterative full spin analysis, along with the observed gHMBC and NOESY correlations
Figure S18. NOESY (blue arrows) and HMBC (red arrows) interactions of 2-methoxycuminol
Figure S19. Mass spectrum (EI, 70 eV) of 2-methoxycuminyl isobutyrate (**3**)
Figure S20. IR spectrum of 2-methoxycuminyl isobutyrate (**3**)
Figure S21. ^{1}H NMR (400 MHz, $CDCl_3$) spectrum of 2-methoxycuminyl isobutyrate (**3**) and the corresponding expansions with signal assignment
Figure S22. Simulated (manual iterative full spin, MestreNova 11.0.3) and experimental ^{1}H NMR spectrum of 2-methoxycuminyl isobutyrate (**3**)
Figure S23. ^{13}C NMR (100.6 MHz, $CDCl_3$) spectrum of 2-methoxycuminyl isobutyrate (**3**)
Table S6. ^{1}H (400 MHz) and ^{13}C (100.6 MHz) NMR data of 2-methoxycuminyl isobutyrate (**3**) (chloroform-*d*), NMR parameters are derived from manual iterative full spin analysis, along with the observed gHMBC and NOESY correlations
Figure S24. NOESY (blue arrows) and HMBC (red arrows) interactions of 2-methoxycuminyl isobutyrate (**3**)
Figure S25. Mass spectrum (EI, 70 eV) of 2-methoxycuminyl 2-methylbutyrate (**4**)
Figure S26. IR spectrum of 2-methoxycuminyl 2-methylbutyrate (**4**)
Figure S27. ^{1}H NMR (400 MHz, $CDCl_3$) spectrum of 2-methoxycuminyl 2-methylbutyrate (**4**) and the corresponding expansions with signal assignment
Figure S28. Simulated (manual iterative full spin, MestreNova 11.0.3) and experimental ^{1}H NMR spectrum of 2-methoxycuminyl 2-methylbutyrate (**4**)
Figure S29. ^{13}C NMR (100.6 MHz, $CDCl_3$) spectrum of 2-methoxycuminyl 2-methylbutyrate (**4**)
Table S7. ^{1}H (400 MHz) and ^{13}C (100.6 MHz) NMR data of 2-methoxycuminyl 2-methylbutyrate (**4**) (chloroform-*d*), NMR parameters are derived from manual iterative full spin analysis, along with the observed gHMBC and NOESY correlations
Figure S30. NOESY (blue arrows) and HMBC (red arrows) interactions of 2-methoxycuminyl 2-methylbutyrate (**4**)
Figure S31. Mass spectrum (EI, 70 eV) of 2-methoxycuminyl isovalerate (**5**)
Figure S32. IR spectrum of 2-methoxycuminyl isobutyrate (**5**)
Figure S33. ^{1}H NMR (400 MHz, $CDCl_3$) spectrum of 2-methoxycuminyl isovalerate (**5**) and the corresponding expansions with signal assignment
Figure S34. Simulated (manual iterative full spin, MestreNova 11.0.3) and experimental ^{1}H NMR spectrum of 2-methoxycuminyl isovalerate (**5**)

Figure S35. ^{13}C NMR (100.6 MHz, $CDCl_3$) spectrum of 2-methoxycuminyl isovalerate (**5**)

Table S8. ^{1}H (400 MHz) and ^{13}C (100.6 MHz) NMR data of 2-methoxycuminyl isovalerate (**5**) (chloroform-*d*), NMR parameters are derived from manual iterative full spin analysis, along with the observed gHMBC and NOESY correlations

Figure S36. NOESY (blue arrows) and HMBC (red arrows) interactions of 2-methoxycuminyl isovalerate (**5**)

Table S9. ^{1}H (400 MHz) and ^{13}C (100.6 MHz) NMR data of 6-hidroxythymyl isobutyrate (**8**) (chloroform-*d*), NMR parameters are derived from manual iterative full spin analysis, along with the observed gHMBC and NOESY correlations

Figure S37. NOESY (blue arrows) and HMBC (red arrows) interactions of 6-hidroxythymyl isobutyrate (**8**)

Table S10. ^{1}H (400 MHz) and ^{13}C (100.6 MHz) NMR data of 6-hidroxythymyl 2-methylbutyrate (**9**) (chloroform-*d*), NMR parameters are derived from manual iterative full spin analysis, along with the observed gHMBC and NOESY correlations

Figure S38. NOESY (blue arrows) and HMBC (red arrows) interactions of 6-hidroxythymyl 2-methylbutyrate (**9**)

Table S11. ^{1}H (400 MHz) and ^{13}C (100.6 MHz) NMR data of 6-isobutyryloxythymol (**10**) (chloroform-*d*), NMR parameters are derived from manual iterative full spin analysis, along with the observed gHMBC and NOESY correlations

Figure S39. NOESY (blue arrows) and HMBC (red arrows) interactions of 6-isobutyryloxythymol (**10**)

Table S12. ^{1}H (400 MHz) and ^{13}C (100.6 MHz) NMR data of 6-(2-methylbutyryloxy)thymol (**11**) (chloroform-*d*), NMR parameters are derived from manual iterative full spin analysis, along with the observed gHMBC and NOESY correlations

Figure S40. NOESY (blue arrows) and HMBC (red arrows) interactions of 6-(2-methylbutyryloxy)thymol (**11**)

Figure S41. Mass spectrum (EI, 70 eV) of 6-methoxythymyl isobutyrate (**12**)

Figure S42. IR spectrum of 6-methoxythymyl isobutyrate(**12**)

Figure S43. ^{1}H NMR (400 MHz, $CDCl_3$) spectrum of 6-methoxythymyl isobutyrate (**12**) and the corresponding expansions with signal assignment

Figure S44. Simulated (manual iterative full spin, MestreNova 11.0.3) and experimental ^{1}H NMR spectrum of 6-methoxythymyl isobutyrate (**12**)

Figure S45. ^{13}C NMR (100.6 MHz, $CDCl_3$) spectrum of 6-methoxythymyl isobutyrate (**12**)

Table S13. ^{1}H (400 MHz) and ^{13}C (100.6 MHz) NMR data of 6-methoxythymyl isobutyrate (**12**) (chloroform-*d*), NMR parameters are derived from manual iterative full spin analysis, along with the observed gHMBC and NOESY correlations

Figure S46. NOESY (blue arrows) and HMBC (red arrows) interactions of 6-methoxythymyl isobutyrate (**12**)

Figure S47. Mass spectrum (EI, 70 eV) of 6-methoxythymyl 2-methylbutyrate (**13**)

Figure S48. IR spectrum of 6-methoxythymyl 2-methylbutyrate (**13**)

Figure S49. ^{1}H NMR (400 MHz, $CDCl_3$) spectrum of 6-methoxythymyl 2-methylbutyrate (**13**) and the corresponding expansions with signal assignment

Figure S50. Simulated (manual iterative full spin, MestreNova 11.0.3) and experimental ^{1}H NMR spectrum of 6-methoxythymyl 2-methylbutyrate (**13**)

Figure S51. ^{13}C NMR (100.6 MHz, $CDCl_3$) spectrum of 6-methoxythymyl 2-methylbutyrate (**13**)

Table S14. ^{1}H (400 MHz) and ^{13}C (100.6 MHz) NMR data of 6-methoxythymyl 2-methylbutyrate (**13**) (chloroform-*d*), NMR parameters are derived from manual iterative full spin analysis, along with the observed gHMBC and NOESY correlations

Figure S52. NOESY (blue arrows) and HMBC (red arrows) interactions of 6-methoxythymyl 2-methylbutyrate (**13**)
Figure S53. Mass spectrum (EI, 70 eV) of 6-isobutyryloxythymyl methyl ether (**14**)
Figure S54. IR spectrum of 6-isobutyryloxythymyl methyl ether (**14**)
Figure S55. ^{1}H NMR (400 MHz, $CDCl_3$) spectrum of 6-isobutyryloxythymyl methyl ether (**14**) and the corresponding expansions with signal assignment
Figure S56. Simulated (manual iterative full spin, MestreNova 11.0.3) and experimental ^{1}H NMR spectrum of 6-isobutyryloxythymyl methyl ether (**14**)
Figure S57. ^{13}C NMR (100.6 MHz, $CDCl_3$) spectrum of 6-isobutyryloxythymyl methyl ether (**14**)
Table S15. ^{1}H (400 MHz) and ^{13}C (100.6 MHz) NMR data of 6-isobutyryloxythymyl methyl ether (**14**) (chloroform-*d*), NMR parameters are derived from manual iterative full spin analysis, along with the observed gHMBC and NOESY correlations
Figure S58. NOESY (blue arrows) and HMBC (red arrows) interactions of 6-isobutyryloxythymyl methyl ether (**14**)
Figure S59. Mass spectrum (EI, 70 eV) of 6-(2-methylbutyryloxy)thymyl methyl ether (**15**)
Figure S60. IR spectrum of 6-(2-methylbutyryloxy)thymyl methyl ether (**15**)
Figure S61. ^{1}H NMR (400 MHz, $CDCl_3$) spectrum of 6-(2-methylbutyryloxy)thymyl methyl ether (**15**) and the corresponding expansions with signal assignment
Figure S62. Simulated (manual iterative full spin, MestreNova 11.0.3) and experimental ^{1}H NMR spectrum of 6-(2-methylbutyryloxy)thymyl methyl ether (**15**)
Figure S63. ^{13}C NMR (100.6 MHz, $CDCl_3$) spectrum of 6-(2-methylbutyryloxy)thymyl methyl ether (**15**)
Table S16. ^{1}H (400 MHz) and ^{13}C (100.6 MHz) NMR data of 6-(2-methylbutyryloxy)thymyl methyl ether (**15**) (chloroform-*d*), NMR parameters are derived from manual iterative full spin analysis, along with the observed gHMBC and NOESY correlations
Figure S64. NOESY (blue arrows) and HMBC (red arrows) interactions of 6-(2-methylbutyryloxy)thymyl methyl ether (**15**)
Figure S65. Mass spectrum (EI, 70 eV) of 8,9-dehydrothymyl methyl ether (**18**)
Figure S66. IR spectrum of 8,9-dehydrothymyl methyl ether (**18**)
Figure S67. ^{1}H NMR (400 MHz, $CDCl_3$) spectrum of 8,9-dehydrothymyl methyl ether (**18**) and the corresponding expansions with signal assignment
Figure S68. Simulated (manual iterative full spin, MestreNova 11.0.3) and experimental ^{1}H NMR spectrum of 8,9-dehydrothymyl methyl ether (**18**)
Figure S69. ^{13}C NMR (100.6 MHz, $CDCl_3$) spectrum of 8,9-dehydrothymyl methyl ether (**18**)
Table S17. ^{1}H (400 MHz) and ^{13}C (100.6 MHz) NMR data of 8,9-dehydrothymyl methyl ether (**18**) (chloroform-*d*), NMR parameters are derived from manual iterative full spin analysis, along with the observed gHMBC and NOESY correlations
Figure S70. NOESY (blue arrows) and HMBC (red arrows) interactions of 8,9-dehydrothymyl methyl ether (**18**)
Table S18. ^{1}H (400 MHz) and ^{13}C (100.6 MHz) NMR data of 9-hydroxythymyl methyl ether (**19**) (chloroform-*d*), NMR parameters are derived from manual iterative full spin analysis, along with the observed gHMBC and NOESY correlations
Figure S71. NOESY (blue arrows) and HMBC (red arrows) interactions of 9-hydroxythymyl methyl ether (**19**)
Figure S72. Mass spectrum (EI, 70 eV) of 9-isobutyryloxyoxythymyl methyl ether (**20**)
Figure S73. IR spectrum of 9-isobutyryloxythymyl methyl ether (**20**)
Figure S74. ^{1}H NMR (400 MHz, $CDCl_3$) spectrum of 9-isobutyryloxythymyl methyl ether (**20**) and the corresponding expansions with signal assignment

Figure S75. Simulated (manual iterative full spin, MestreNova 11.0.3) and experimental ^{1}H NMR spectrum of 9-isobutyryloxyoxythymyl methyl ether (**20**)
Figure S76. ^{13}C NMR (100.6 MHz, $CDCl_3$) spectrum of 9-isobutyryloxythymyl methyl ether (**20**)
Table S19. ^{1}H (400 MHz) and ^{13}C (100.6 MHz) NMR data of 9-isobutyryloxythymyl methyl ether (**20**) (chloroform-*d*), NMR parameters are derived from manual iterative full spin analysis, along with the observed gHMBC and NOESY correlations
Figure S77. NOESY (blue arrows) and HMBC (red arrows) interactions 9-(2-methylbutyryloxy)thymyl methyl ether (**20**)
Figure S78. Mass spectrum (EI, 70 eV) of 9-(2-methylbutyryloxy)thymyl methyl ether (**21**)
Figure S79. IR spectrum of 9-(2-methylbutyryloxy)thymyl methyl ether (**21**)
Figure S80. ^{1}H NMR (400 MHz, $CDCl_3$) spectrum of 9-(2-methylbutyryloxy)thymyl methyl ether (**21**) and the corresponding expansions with signal assignment
Figure S81. Simulated (manual iterative full spin, MestreNova 11.0.3) and experimental ^{1}H NMR spectrum of 9-(2-methylbutyryloxy)thymyl methyl ether (**21**)
Figure S82. ^{13}C NMR (100.6 MHz, $CDCl_3$) spectrum of 9-(2-methylbutyryloxy)thymyl methyl ether (**21**)
Table S20. ^{1}H (400 MHz) and ^{13}C (100.6 MHz) NMR data of 9-(2-methylbutyryloxy)thymyl methyl ether (**21**) (chloroform-*d*), NMR parameters are derived from manual iterative full spin analysis, along with the observed gHMBC and NOESY correlations
Figure S83. NOESY (blue arrows) and HMBC (red arrows) interactions 9-(2-methylbutyryloxy)thymyl methyl ether (**21**)
Table S21. ^{1}H (400 MHz) and ^{13}C (100.6 MHz) NMR data of 3-methoxycumic acid (chloroform-*d*), NMR parameters are derived from manual iterative full spin analysis, along with the observed gHMBC and NOESY correlations
Figure S84. NOESY (blue arrows) and HMBC (red arrows) interactions 3-methoxycumic acid
Figure S85. Mass spectrum (EI, 70 eV) of methyl 3-methoxycuminate (**23**)
Figure S86. IR spectrum of methyl 3-methoxycuminate (**23**)
Figure S87. ^{1}H NMR (400 MHz, $CDCl_3$) spectrum of methyl 3-methoxycuminate (**23**) and the corresponding expansions with signal assignment
Figure S88. Simulated (manual iterative full spin, MestreNova 11.0.3) and experimental ^{1}H NMR spectrum of methyl 3-methoxycuminate (**23**)
Figure S89. ^{13}C NMR (100.6 MHz, $CDCl_3$) spectrum of methyl 3-methoxycuminate (**23**)
Table S22. ^{1}H (400 MHz) and ^{13}C (100.6 MHz) NMR data of methyl 3-methoxycuminate (**23**) (chloroform-*d*), NMR parameters are derived from manual iterative full spin analysis, along with the observed gHMBC and NOESY correlations
Figure S90. NOESY (blue arrows) and HMBC (red arrows) interactions methyl 3-methoxycuminate (**23**)
Figure S91. Mass spectrum (EI, 70 eV) of 3-methoxycuminaldehyde (**24**)
Figure S92. IR spectrum of 3-methoxycuminaldehyde (**24**)
Figure S93. ^{1}H NMR (400 MHz, $CDCl_3$) spectrum of 3-methoxycuminaldehyde (**24**) and the corresponding expansions with signal assignment
Figure S94. Simulated (manual iterative full spin, MestreNova 11.0.3) and experimental ^{1}H NMR spectrum of 3-methoxycuminaldehyde (**24**)
Figure S95. ^{13}C NMR (100.6 MHz, $CDCl_3$) spectrum of 3-methoxycuminaldehyde (**24**)
Table S23. ^{1}H (400 MHz) and ^{13}C (100.6 MHz) NMR data of 3-methoxycuminaldehyde (**24**) (chloroform-*d*), NMR parameters are derived from manual iterative full spin analysis, along with the observed gHMBC and NOESY correlations
Figure S96. NOESY (blue arrows) and HMBC (red arrows) interactions 3-methoxycuminaldehyde (**24**)

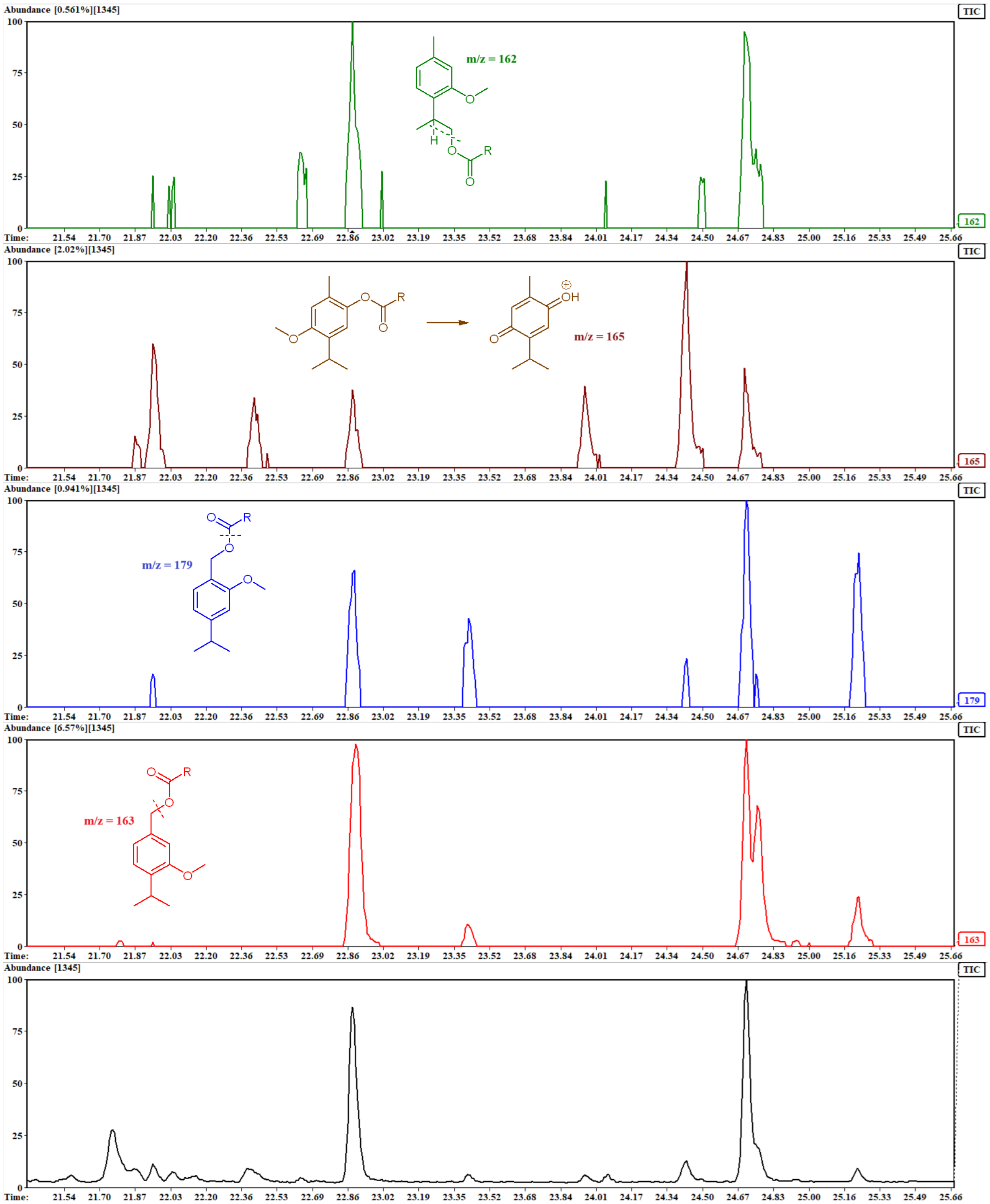

Figure S1. Part of the chromatogram of the essential oil fraction (colored in black) and patrial ion current chromatograms (ion at *m/z* 163, 179, 165, and 162 (colored in red, blue, brown, and green, respectively))

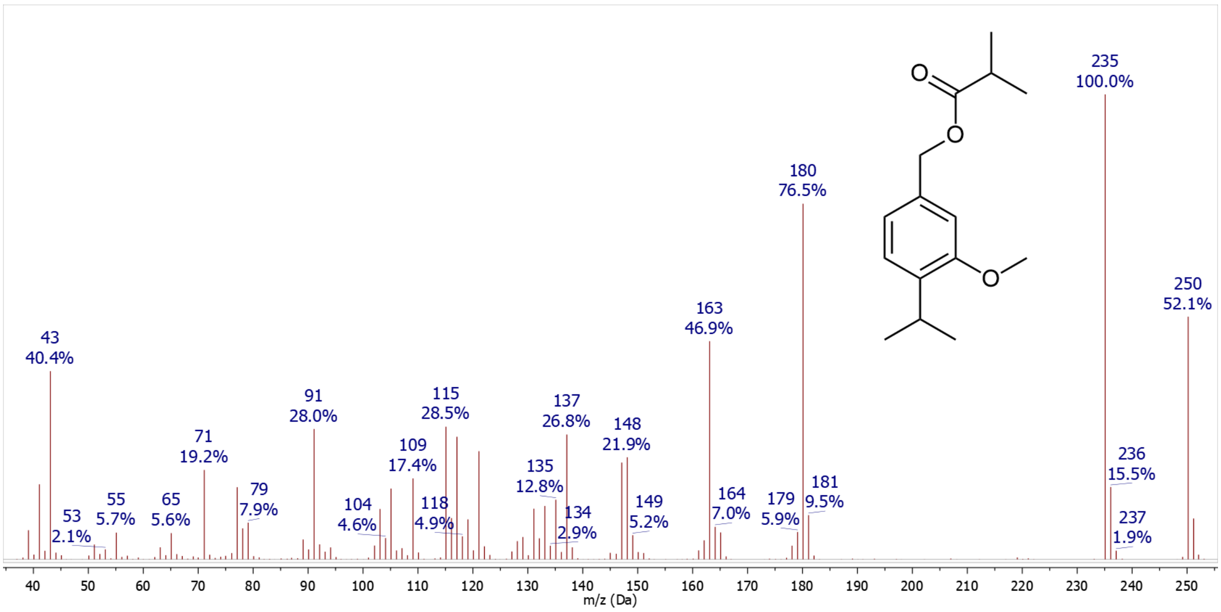

Figure S2. Mass spectrum (EI, 70 eV) of 3-methoxycuminyl isobutyrate

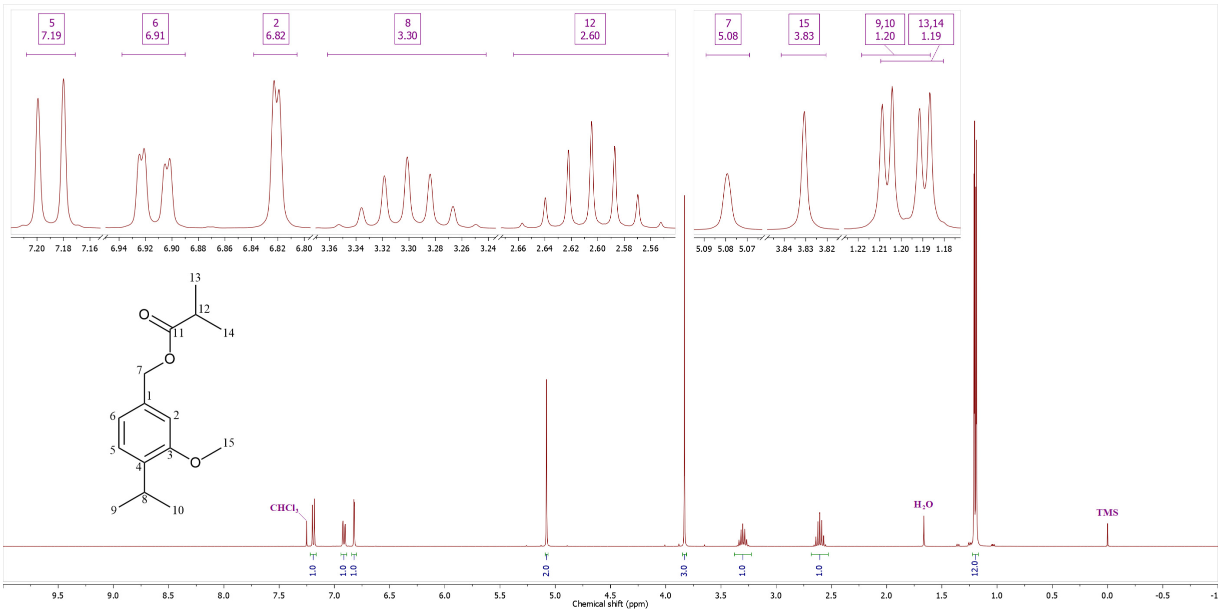

Figure S3. ^{1}H NMR (400 MHz, $CDCl_3$) spectrum of 3-methoxycuminyl isobutyrate and the corresponding expansions with signal assignment

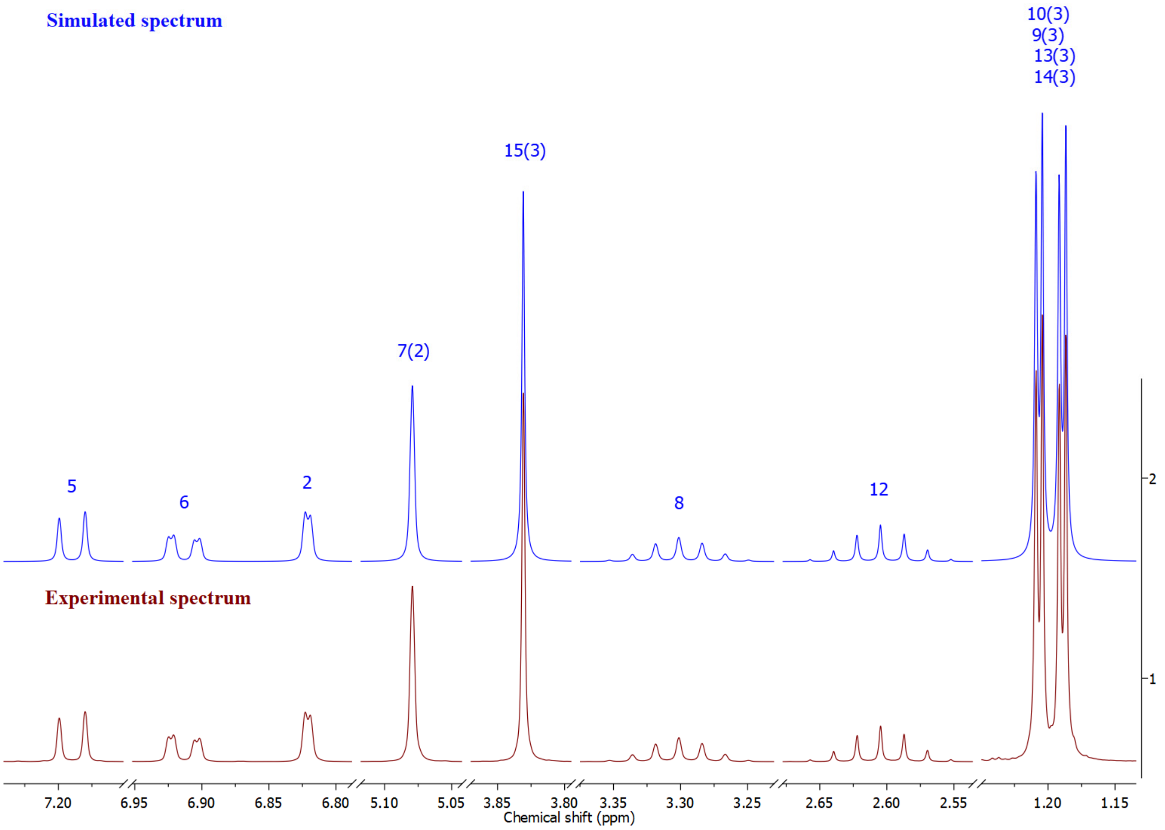

Figure S4. Simulated (manual iterative full spin, MestreNova 11.0.3) and experimental ^{1}H NMR spectrum of 3-methoxycuminyl isobutyrate

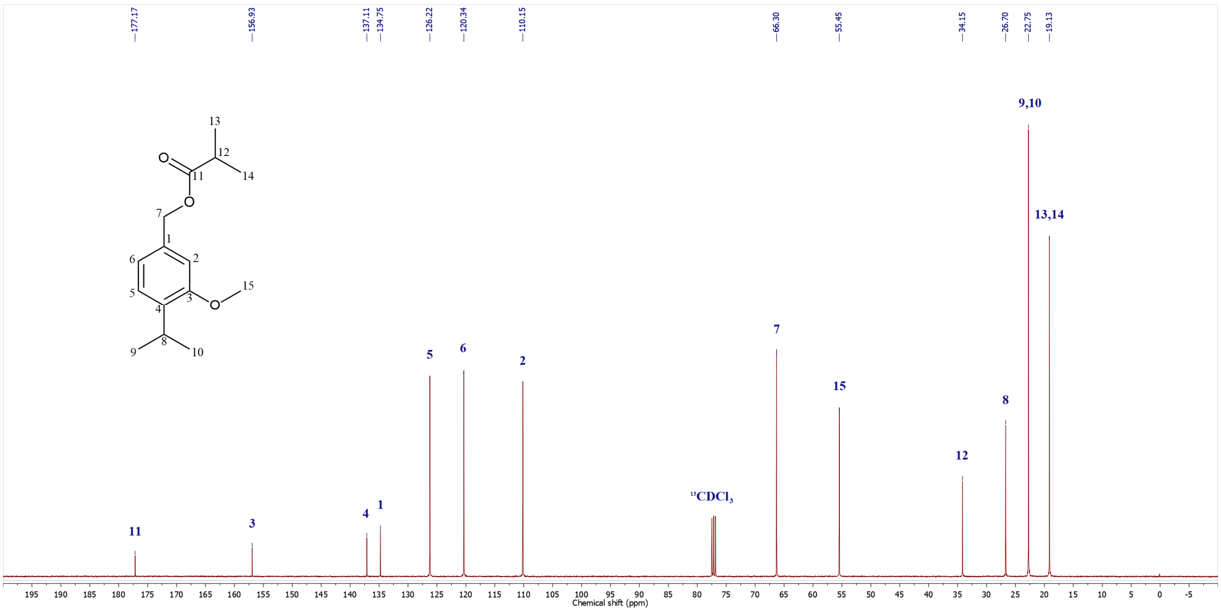

Figure S5. ^{13}C NMR (100.6 MHz, $CDCl_3$) spectrum of 3-methoxycuminyl isobutyrate

Table S1. ^{1}H (400 MHz) and ^{13}C (100.6 MHz) NMR data of 3-methoxycuminyl isobutyrate (chloroform-*d*), NMR parameters are derived from manual iterative full spin analysis, along with the observed gHMBC and NOESY correlations

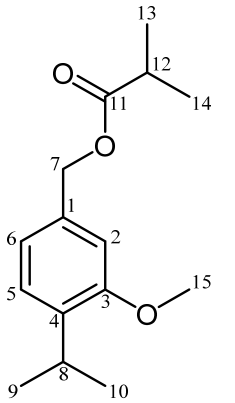

Position	δ_H (m, *J* (Hz), Integration)	δ_C	HMBC[a]	NOESY[b]
1	/	134.75	/	/
2	6.8211 (dtd, $^{4}J_{2,6} = 1.6$, $^{4}J_{2,7} = -0.5$, $^{5}J_{2,8} = 0.3$, 1 H)	110.15	3,4,6,7	7,15
3	/	156.93	/	/
4	/	137.11	/	/
5	7.1893 (ddt, $^{3}J_{5,6} = 7.7$, $^{4}J_{5,8} = -0.5$, $^{5}J_{5,7} = 0.3$, 1 H)	126.22	1,3,8	6,9,10
6	6.9135 (ddtd, $^{3}J_{5,6} = 7.7$, $^{4}J_{2,6} = 1.6$, $^{4}J_{6,7} = -0.6$, $^{5}J_{6,8} = 0.3$, 1 H)	120.34	2,4,7	5,7
7	5.0793 (ddd, $^{4}J_{6,7} = -0.6$, $^{4}J_{2,7} = -0.5$, $^{5}J_{5,7} = 0.3$, 2 H)	66.30	1,2,6,11	2,6
8	3.3011 (septddd, $^{3}J_{8,9/10} = 6.9$, $^{4}J_{5,8} = -0.5$, $^{5}J_{2,8} = 0.3$, $^{5}J_{6,8} = 0.3$, 1 H)	26.70	3,4,5,9,10	9,10
9 and 10	1.2003 (dd, $^{3}J_{8,9/10} = 6.9$, 6 H)	22.75	4,8,9,10	5,8
11	/	177.17	/	/
12	2.6044 (sept, $^{3}J_{12,13/14} = 7.0$, 1 H)	34.15	11,13,14	13,14
13 and 14	1.1955 (d, $^{3}J_{12,13/14} = 7.0$, 6 H)	19.13	11,12,13,14	12
15	3.8307 (s, 3 H)	55.45	3	2

[a] gHMBC correlations observed between the hydrogen in this row and the carbon in the listed position.
[b] Cross-peaks observed in the NOESY spectrum.

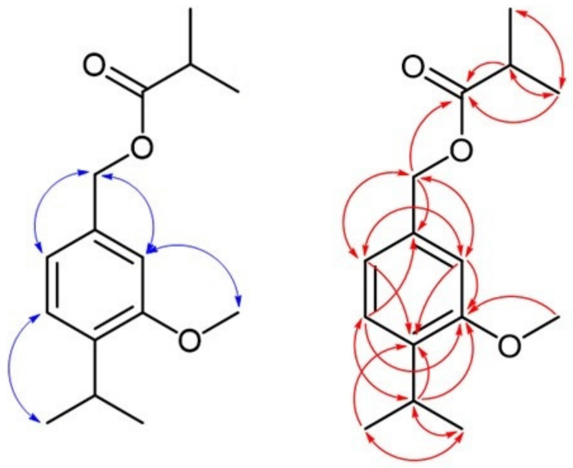

Figure S6. NOESY (blue arrows) and HMBC (red arrows) interactions of 3-methoxycuminyl isobutyrate

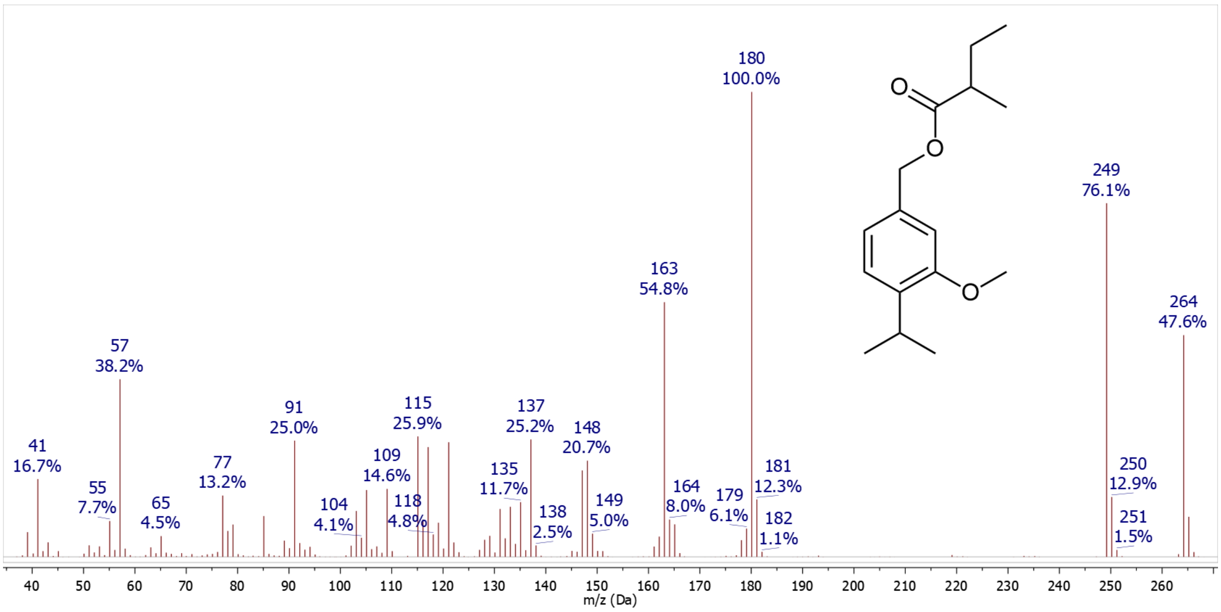

Figure S7. Mass spectrum (EI, 70 eV) of 3-methoxycuminyl 2-methylbutyrate

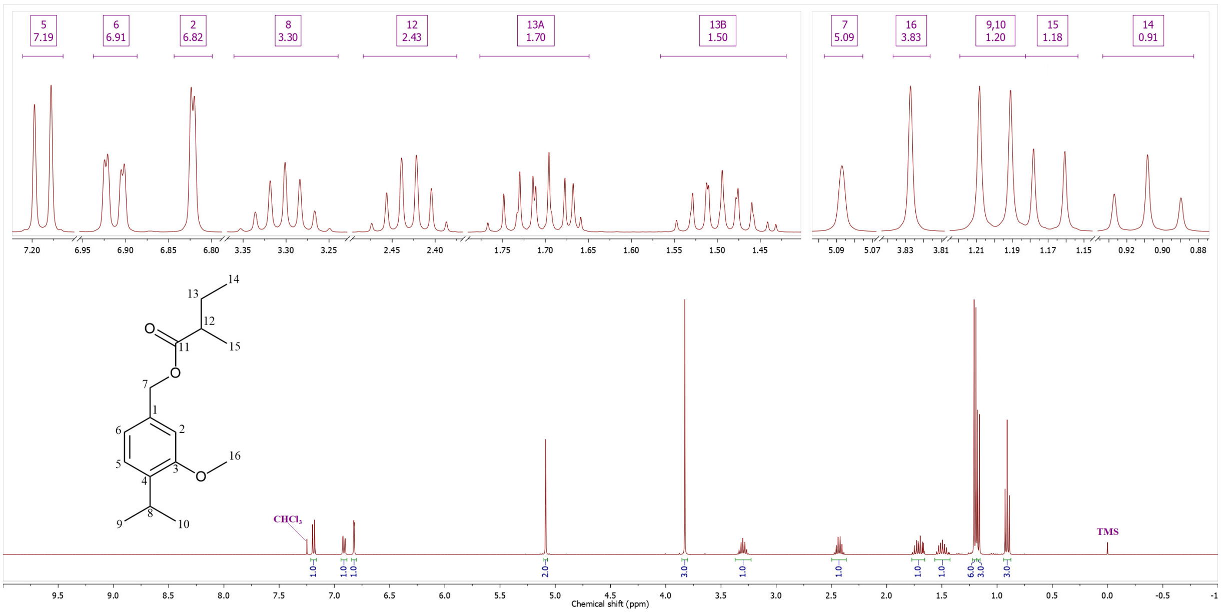

Figure S8. ^{1}H NMR (400 MHz, $CDCl_3$) spectrum of 3-methoxycuminyl 2-methylbutyrate and the corresponding expansions with signal assignment

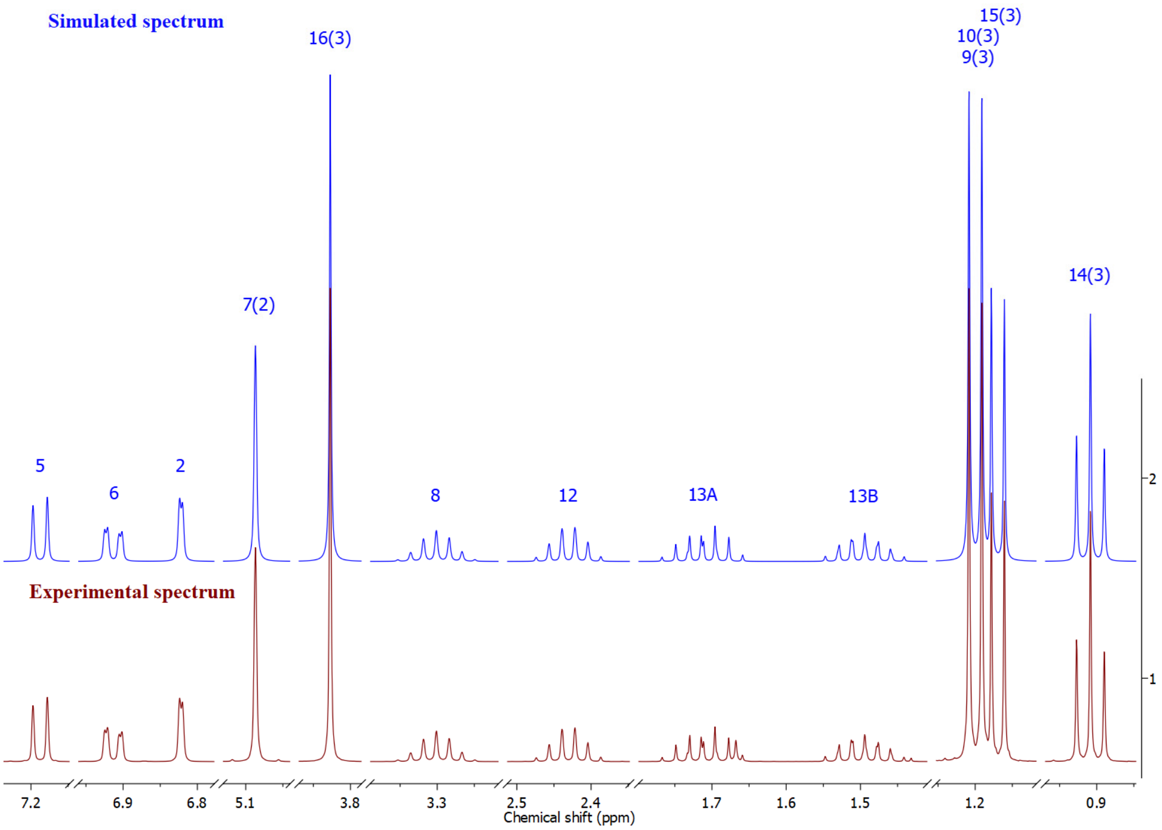

Figure S9. Simulated (manual iterative full spin, MestreNova 11.0.3) and experimental ^{1}H NMR spectrum of 3-methoxycuminyl 2-methylbutyrate

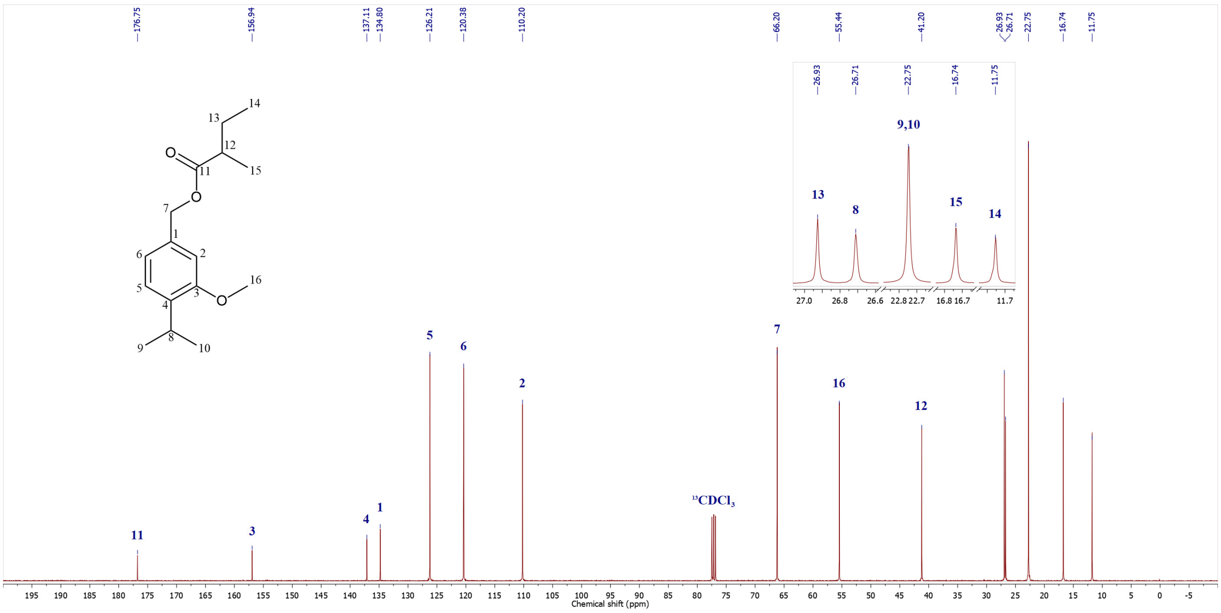

Figure S10. ^{13}C NMR (100.6 MHz, $CDCl_3$) spectrum of 3-methoxycuminyl 2-methylbutyrate

Table S2. ^{1}H (400 MHz) and ^{13}C (100.6 MHz) NMR data of 3-methoxycuminyl 2-methylbutyrate (chloroform-*d*), NMR parameters are derived from manual iterative full spin analysis, along with the observed gHMBC and NOESY correlations

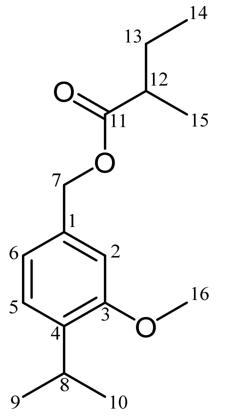

Position	δH (m, *J* (Hz), Integration)	δC	HMBC[a]	NOESY[b]
1	/	134.80	/	/
2	6.8220 (dtd, $^{4}J_{2,6}$ = 1.7, $^{4}J_{2,7}$ = -0.5, $^{5}J_{2,8}$ = 0.3, 1 H)	110.20	3,4,6,7	7,16
3	/	156.94	/	/
4	/	137.11	/	/
5	7.1873 (ddt, $^{3}J_{5,6}$ = 7.7, $^{4}J_{5,8}$ = -0.5, $^{5}J_{5,7}$ = 0.3, 1 H)	126.21	1,3,8	6,9,10
6	6.9135 (ddtd, $^{3}J_{5,6}$ = 7.7, $^{4}J_{2,6}$ = 1.7, $^{4}J_{6,7}$ = -0.5, $^{5}J_{6,8}$ = 0.3, 1 H)	120.38	2,4,7	5,7
7	5.0870 (ddd, $^{4}J_{2,7}$ = -0.5, $^{4}J_{6,7}$ = -0.5, $^{5}J_{5,7}$ = 0.3, 2 H)	66.20	1,2,6,11	2,6
8	3.3010 (septddd, $^{3}J_{8,9/10}$ = 6.9, $^{4}J_{5,8}$ = -0.5, $^{5}J_{2,8}$ = 0.3, $^{5}J_{6,8}$ = 0.3, 1 H)	26.71	3,4,5,9,10	9,10
9 and 10	1.1997 (d, $^{3}J_{8,9/10}$ = 6.9, 6 H)	22.75	4,8,9,10	5,8
11	/	176.75	/	/
12	2.4300 (dqdq, $^{3}J_{12,13A}$ = 7.5, $^{3}J_{12,15}$ = 7.0, $^{3}J_{12,13B}$ = 6.5, $^{4}J_{12,14}$ = 0.3, 1 H)	41.20	11,13,14,15	13A,13B,14,15
13A	1.7114 (ddq, $^{2}J_{13A,13B}$ = -13.7, $^{3}J_{12,13A}$ = 7.5, $^{3}J_{13A,14}$ = 7.5, 1 H)	26.93	11,12,14,15	12,14,15
13B	1.4952 (dqd, $^{2}J_{13A,13B}$ = -13.7, $^{3}J_{13B,14}$ = 7.5, $^{3}J_{12,13B}$ = 6.5, 1 H)	26.93	11,12,14,15	12,14,15
14	0.9087 (ddd, $^{3}J_{13A,14}$ = 7.5, $^{3}J_{13B,14}$ = 7.5, $^{4}J_{12,14}$ = 0.3, 3 H)	11.75	12,13	12,13A,13B
15	1.1695 (d, $^{3}J_{12,15}$ = 7.0, 3 H)	16.74	11,12,13	12,13A,13B
16	3.8272 (s, 3 H)	55.44	3	2

[a] gHMBC correlations observed between the hydrogen in this row and the carbon in the listed position.

[b] Cross-peaks observed in the NOESY spectrum.

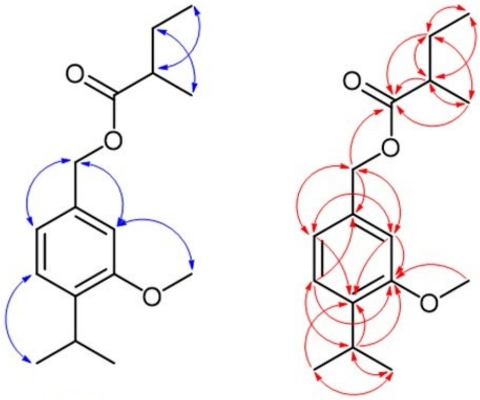

Figure S11. NOESY (blue arrows) and HMBC (red arrows) interactions of 3-methoxycuminyl 2-methylbutyrate

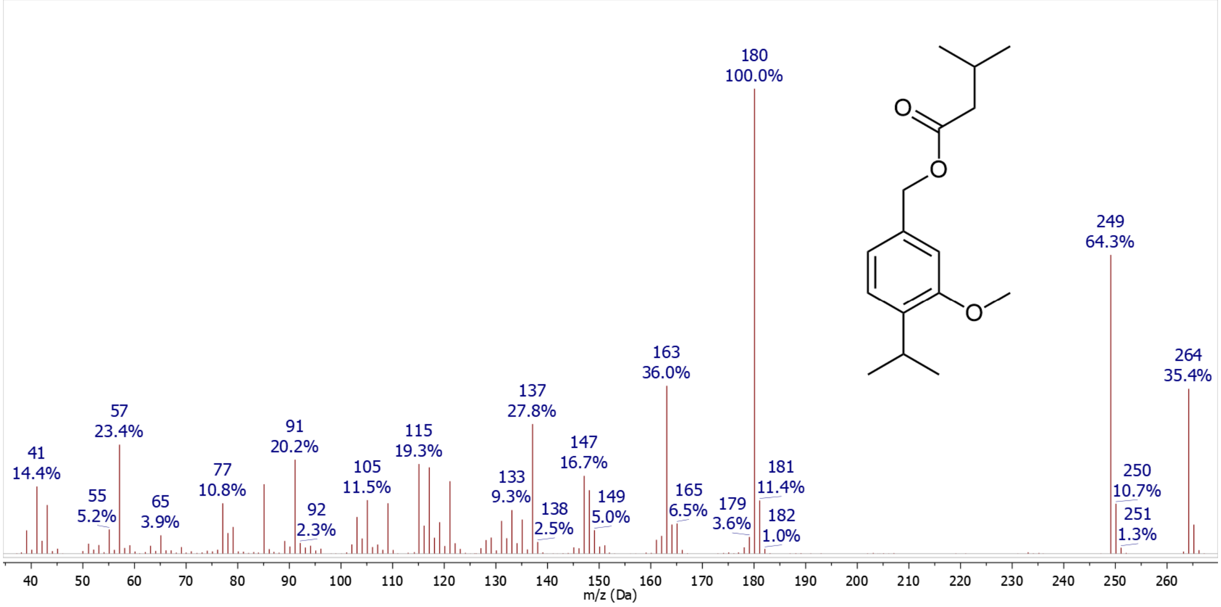

Figure S12. Mass spectrum (EI, 70 eV) of 3-methoxycuminyl isovalerate

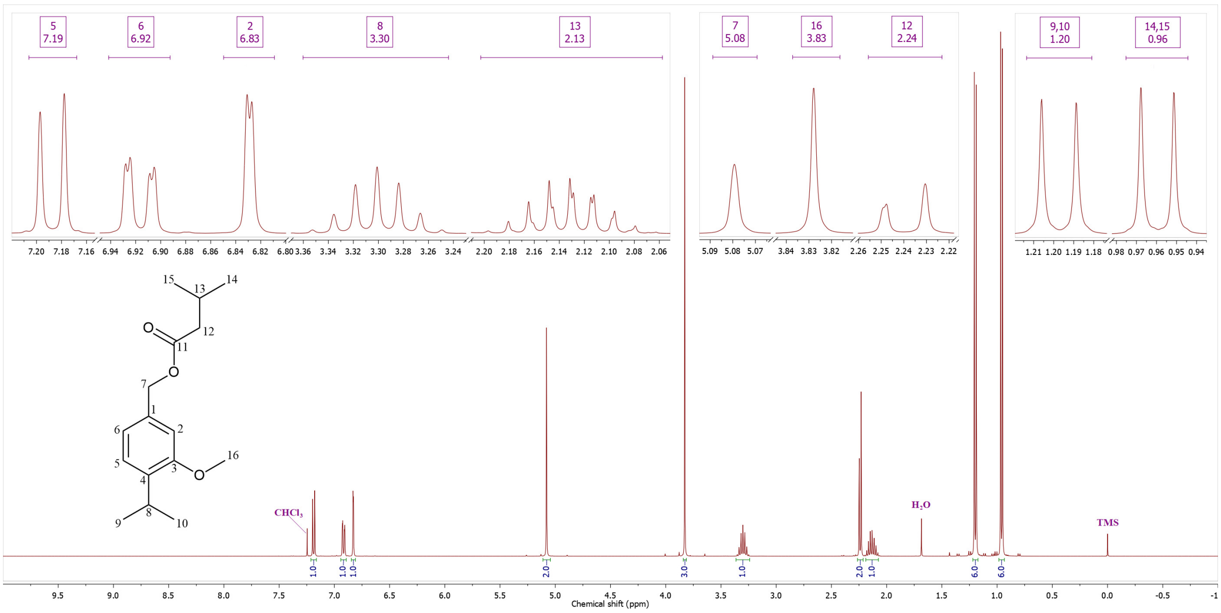

Figure S13. ^{1}H NMR (400 MHz, $CDCl_3$) spectrum of 3-methoxycuminyl isovalerate and the corresponding expansions with signal assignment

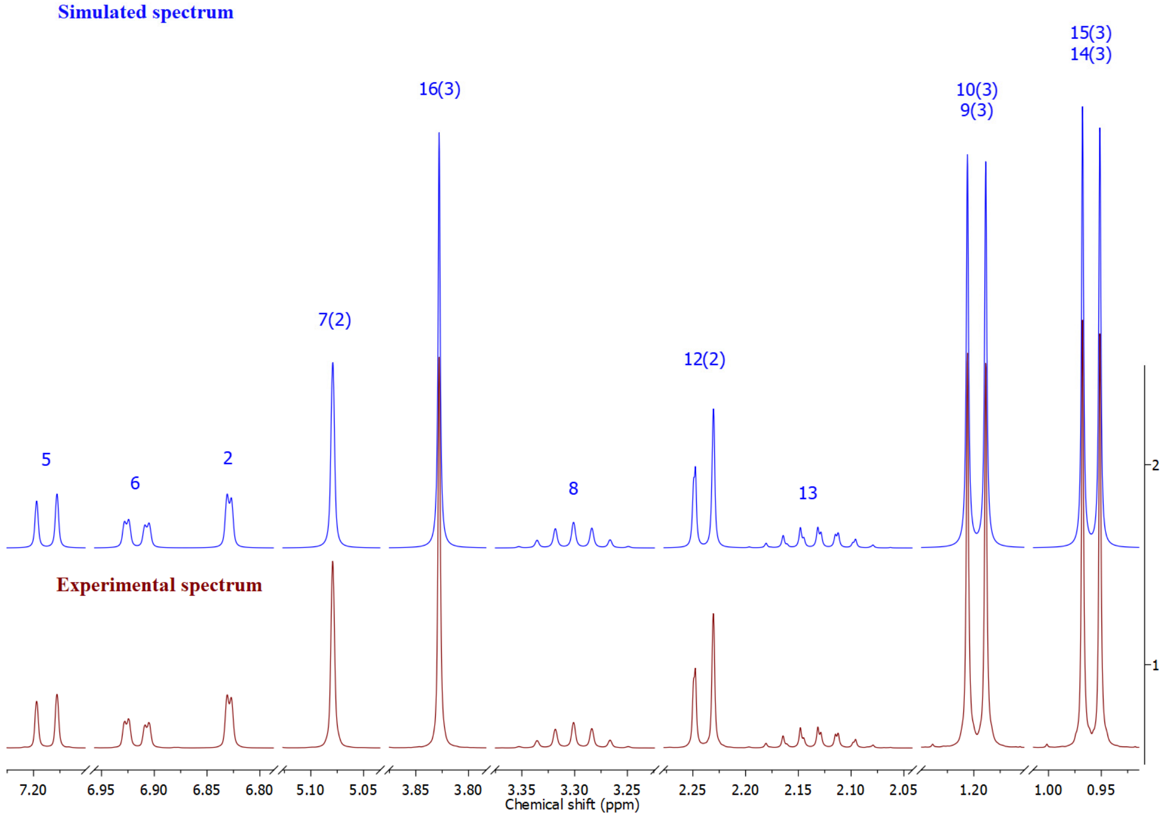

Figure S14. Simulated (manual iterative full spin, MestreNova 11.0.3) and experimental ^{1}H NMR spectrum of 3-methoxycuminyl isovalerate

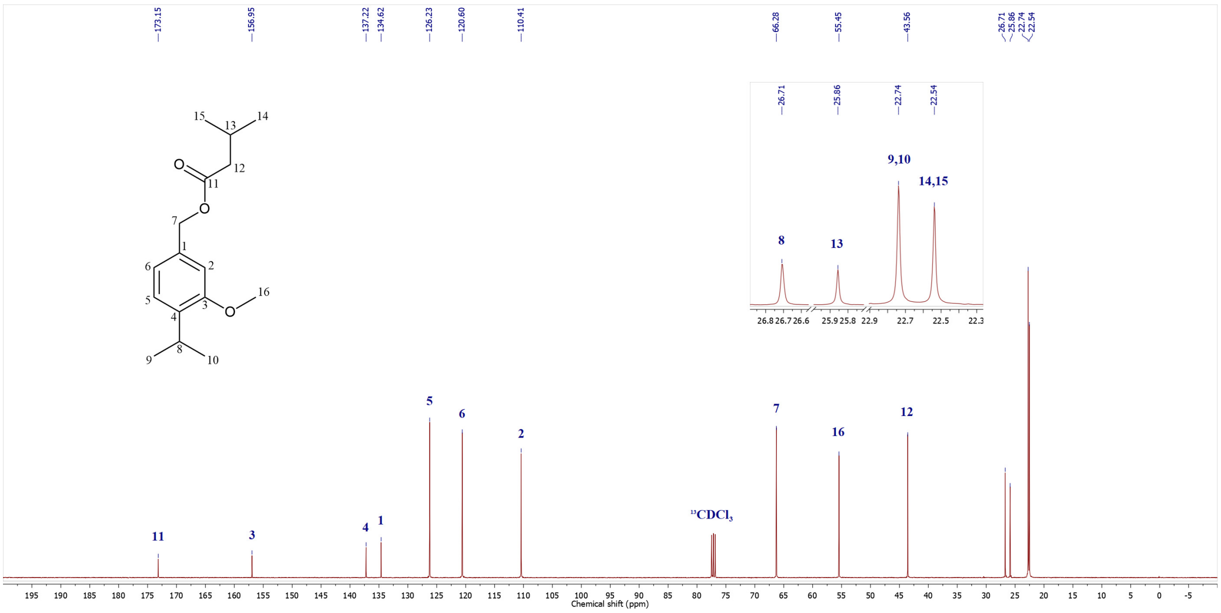

Figure S15. ^{13}C NMR (100.6 MHz, $CDCl_3$) spectrum of 3-methoxycuminyl isovalerate

Table S3. ^{1}H (400 MHz) and ^{13}C (100.6 MHz) NMR data of 3-methoxycuminyl isovalerate (chloroform-*d*), NMR parameters are derived from manual iterative full spin analysis, along with the observed gHMBC and NOESY correlations

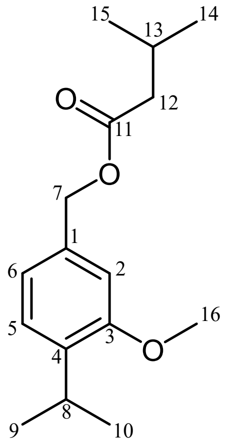

Position	δ_H (m, *J* (Hz), Integration)	δ_C	HMBC[a]	NOESY[b]
1	/	134.62	/	/
2	6.8290 (dtd, $^4J_{2,6}$ = 1.7, $^4J_{2,7}$ = -0.5, $^5J_{2,8}$ = 0.3, 1 H)	110.41	3,4,6,7	7,16
3	/	156.95	/	/
4	/	137.22	/	/
5	7.1871 (ddt, $^3J_{5,6}$ = 7.7, $^4J_{5,8}$ = -0.5, $^5J_{5,7}$ = 0.3, 1 H)	126.23	1,3,8	6,9,10
6	6.9169 (ddtd, $^3J_{5,6}$ = 7.7, $^4J_{2,6}$ = 1.7, $^4J_{6,7}$ = -0.5, $^5J_{6,8}$ = 0.3, 1 H)	120.60	2,4,7	5,7
7	5.0793 (ddd, $^4J_{2,7}$ = -0.5, $^4J_{6,7}$ = -0.5, $^5J_{5,7}$ = 0.3, 2 H)	66.28	1,2,6,11	2,6
8	3.3008 (septddd, $^3J_{8,9/10}$ = 6.9, $^4J_{5,8}$ = -0.5, $^5J_{2,8}$ = 0.3, $^5J_{6,8}$ = 0.3, 1 H)	26.71	3,4,5,9,10	9,10
9 and 10	1.1974 (d, $^3J_{8,9/10}$ = 6.9, 6 H)	22.74	4,8,9,10	5,8
11	/	173.15	/	/
12	2.2387 (d, $^3J_{12,13}$ = 7.2, 2 H)	43.56	11,13,14,15	13,14,15
13	2.1313 (tsept, $^3J_{12,13}$ = 7.2, $^3J_{13,14/15}$ = 6.7, 1 H)	25.86	11,12,14,15	12,14,15
14 and 15	0.9596 (d, $^3J_{13,14/15}$ = 6.7, 6 H)	22.54	12,13,14,15	12,13
16	3.8279 (s, 3 H)	55.44	3	2

[a] gHMBC correlations observed between the hydrogen in this row and the carbon in the listed position.
[b] Cross-peaks observed in the NOESY spectrum.

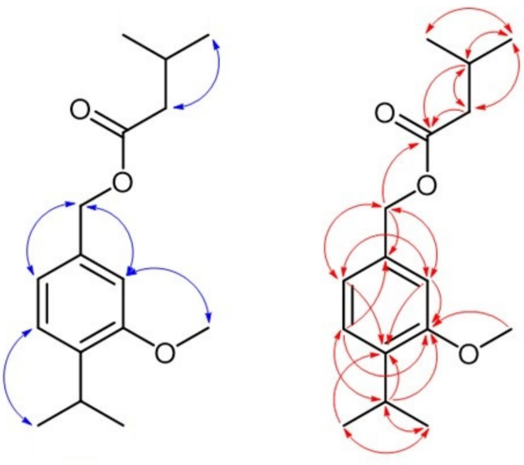

Figure S16. NOESY (blue arrows) and HMBC (red arrows) interactions of 3-methoxycuminyl isovalerate

Table S4. ^{1}H (400 MHz) and ^{13}C (100.6 MHz) NMR data of 2-methoxycuminaldehyde (**2**) (chloroform-*d*), NMR parameters are derived from manual iterative full spin analysis, along with the observed gHMBC and NOESY correlations

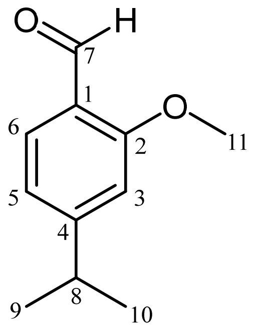

Position	δ_H (m, J (Hz), Integration)	δ_C	HMBC[a]	NOESY[b]
1	/	123.12	/	/
2	/	162.17	/	/
3	6.8201 (ddd, $^4J_{3,5}$ = 1.5, $^4J_{3,8}$ = -0.6, $^5J_{3,6}$ = 0.3, 1 H)	109.77	1,2,5,8	8,9,10,11
4	/	158.38	/	/
5	6.9066 (dddd, $^3J_{5,6}$ = 8.0, $^4J_{3,5}$ = 1.5, $^5J_{5,7}$ = 0.7, $^4J_{5,8}$ = -0.5, 1 H)	119.11	1,3,8	6,8,9,10
6	7.7633 (ddd, $^3J_{5,6}$ = 8.0, $^5J_{3,6}$ = 0.3, $^5J_{6,8}$ = 0.3, 1 H)	128.90	2,4,7	5,7
7	10.4032 (d, $^5J_{5,7}$ = 0.7, 1 H)	189.67	1,6	6,11
8	2.9444 (septddd, $^3J_{8,9/10}$ = 6.9, $^4J_{3,8}$ = -0.6, $^4J_{5,8}$ = -0.5, $^5J_{6,8}$ = 0.3, 1 H)	35.08	3,4,5,9,10	3,5,9,10
9 and 10	1.2761 (d, $^3J_{8,9/10}$ = 6.9, 6 H)	23.72	4,8,9,10	3,5,8
11	3.9365 (s, 3 H)	55.68	2	3,7

[a] gHMBC correlations observed between the hydrogen in this row and the carbon in the listed position.
[b] Cross-peaks observed in the NOESY spectrum.

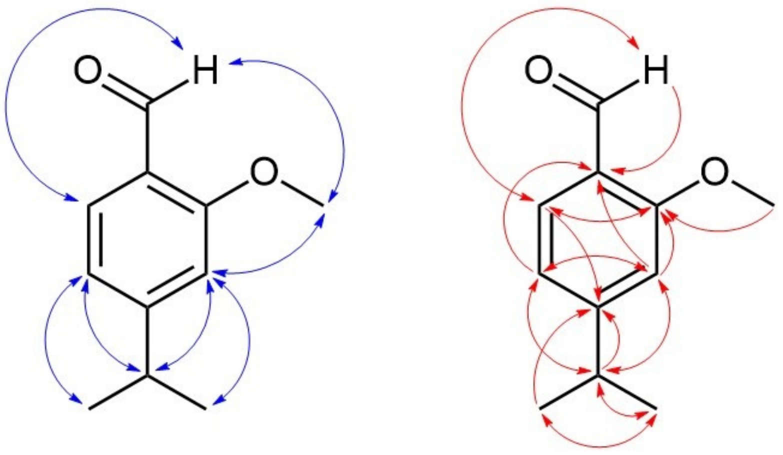

Figure S17. NOESY (blue arrows) and HMBC (red arrows) interactions of 2-methoxycuminaldehyde (**2**)

Table S5. ^{1}H (400 MHz) and ^{13}C (100.6 MHz) NMR data of 2-methoxycuminol (chloroform-*d*), NMR parameters are derived from manual iterative full spin analysis, along with the observed gHMBC and NOESY correlations

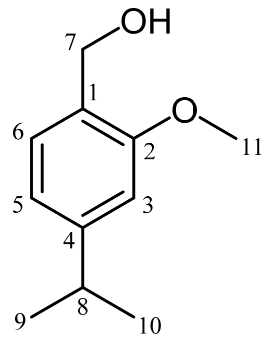

Position	δ_H (m, J (Hz), Integration)	δ_C	HMBC[a]	NOESY[b]
1	/	126.58	/	/
2	/	157.64	/	/
3	6.7549 (dddt, $^{4}J_{3,5}$ = 1.6, $^{4}J_{3,8}$ = -0.6, $^{5}J_{3,6}$ = 0.3, $^{5}J_{3,7}$ = 0.3, 1 H)	108.80	1,2,5,8	8,9,10,11
4	/	150.53	/	/
5	6.8141 (dddt, $^{3}J_{5,6}$ = 7.6, $^{4}J_{3,5}$ = 1.6, $^{4}J_{5,8}$ = -0.6, $^{5}J_{5,7}$ = 0.3, 1 H)	118.50	1,3,8	6,8,9,10
6	7.1799 (dtdd, $^{3}J_{5,6}$ = 7.6, $^{4}J_{6,7}$ = -0.6, $^{5}J_{3,6}$ = 0.3, $^{5}J_{6,8}$ = 0.3, 1 H)	128.99	2,4,7	5,7
7	4.6505 (ddd, $^{4}J_{6,7}$ = -0.6, $^{5}J_{3,7}$ = 0.3, $^{5}J_{5,7}$ = 0.3, 2 H)	62.27	1,2,6	5
8	2.9004 (septddd, $^{3}J_{8,9/10}$ = 6.9, $^{4}J_{3,8}$ = -0.6, $^{4}J_{5,8}$ = -0.6, $^{5}J_{6,8}$ = 0.3, 1 H)	34.49	3,4,5,9,10	3,5,9,10
9 and 10	1.2552 (d, $^{3}J_{8,9/10}$ = 6.9, 6 H)	24.18	4,8,9,10	3,5,8
11	3.8786 (s, 3 H)	55.33	2	3
OH	2.2814 (brs, 1 H)	/	/	/

[a] gHMBC correlations observed between the hydrogen in this row and the carbon in the listed position.
[b] Cross-peaks observed in the NOESY spectrum.

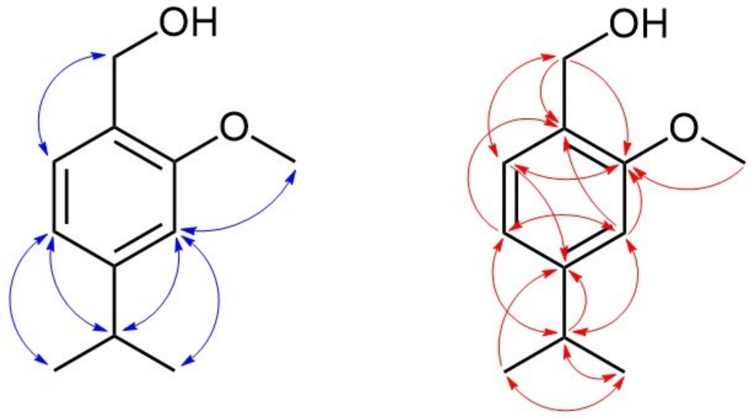

Figure S18. NOESY (blue arrows) and HMBC (red arrows) interactions of 2-methoxycuminol

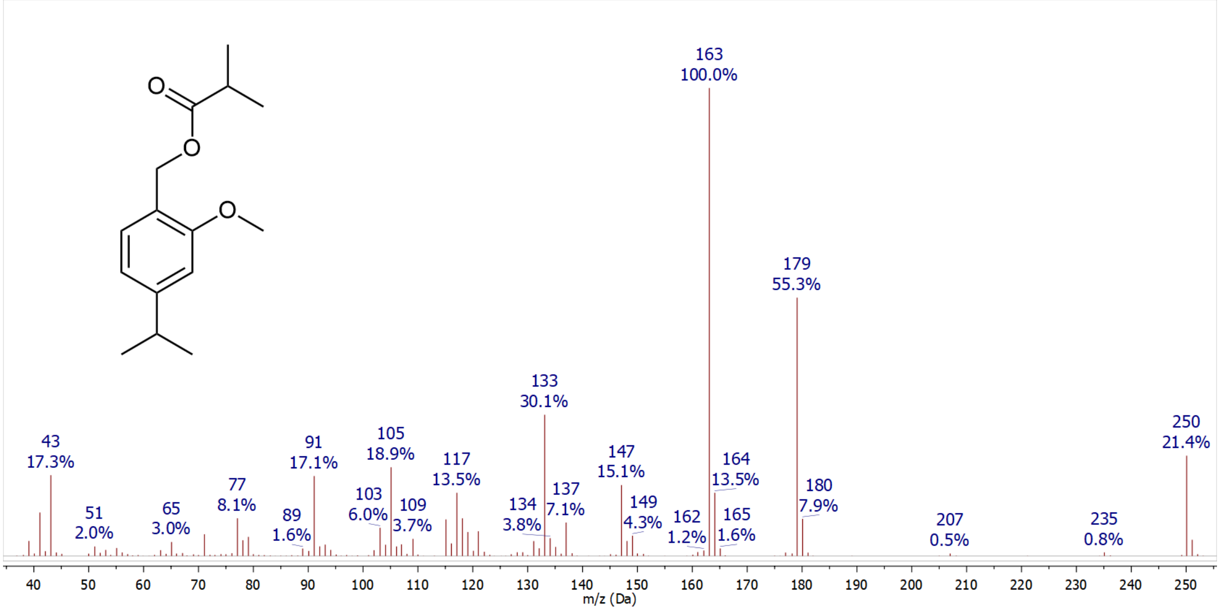

Figure S19. Mass spectrum (EI, 70 eV) of 2-methoxycuminyl isobutyrate (**3**)

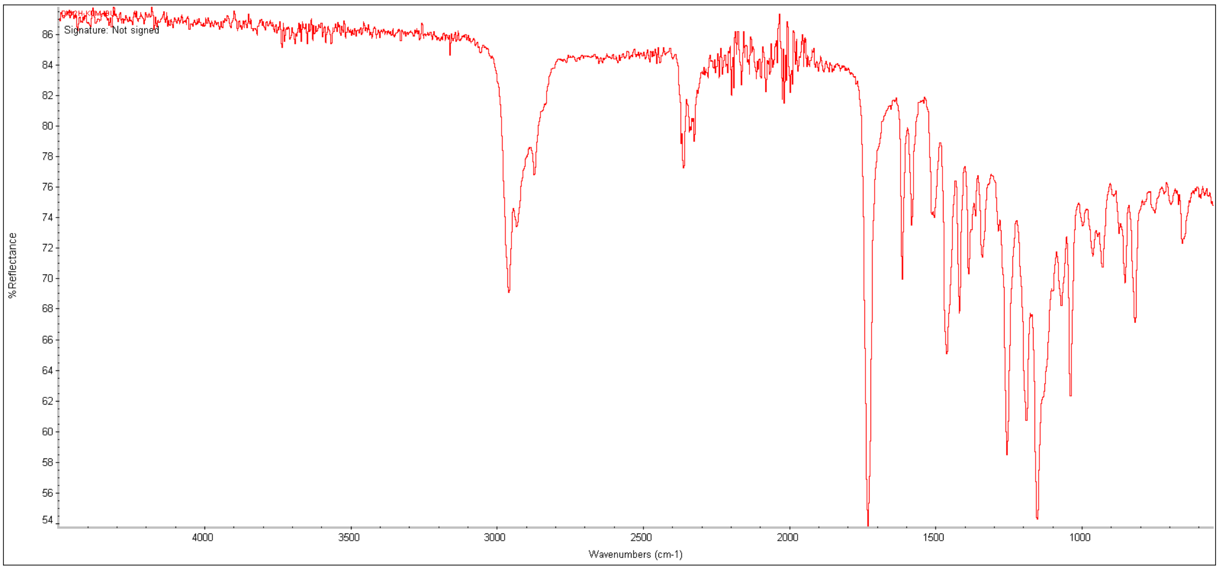

Figure S20. IR spectrum of 2-methoxycuminyl isobutyrate (**3**)

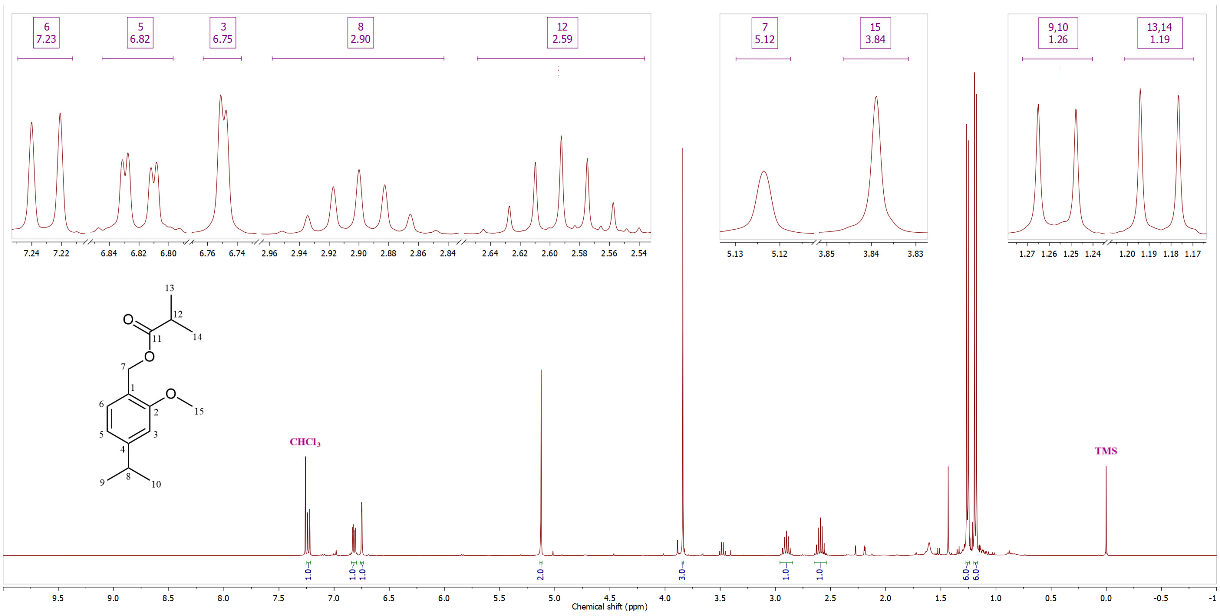

Figure S21. ^{1}H NMR (400 MHz, $CDCl_3$) spectrum of 2-methoxycuminyl isobutyrate (**3**) and the corresponding expansions with signal assignment

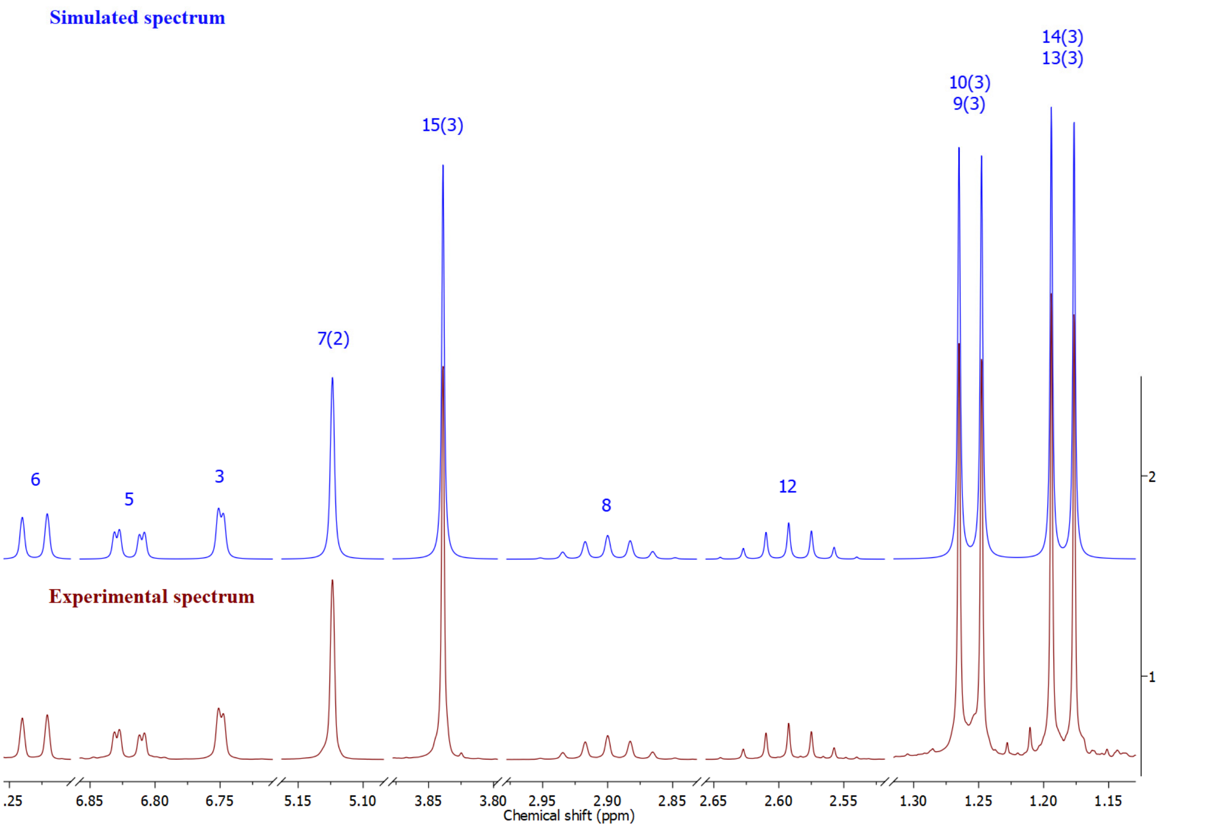

Figure S22. Simulated (manual iterative full spin, MestreNova 11.0.3) and experimental ^{1}H NMR spectrum of 2-methoxycuminyl isobutyrate (**3**)

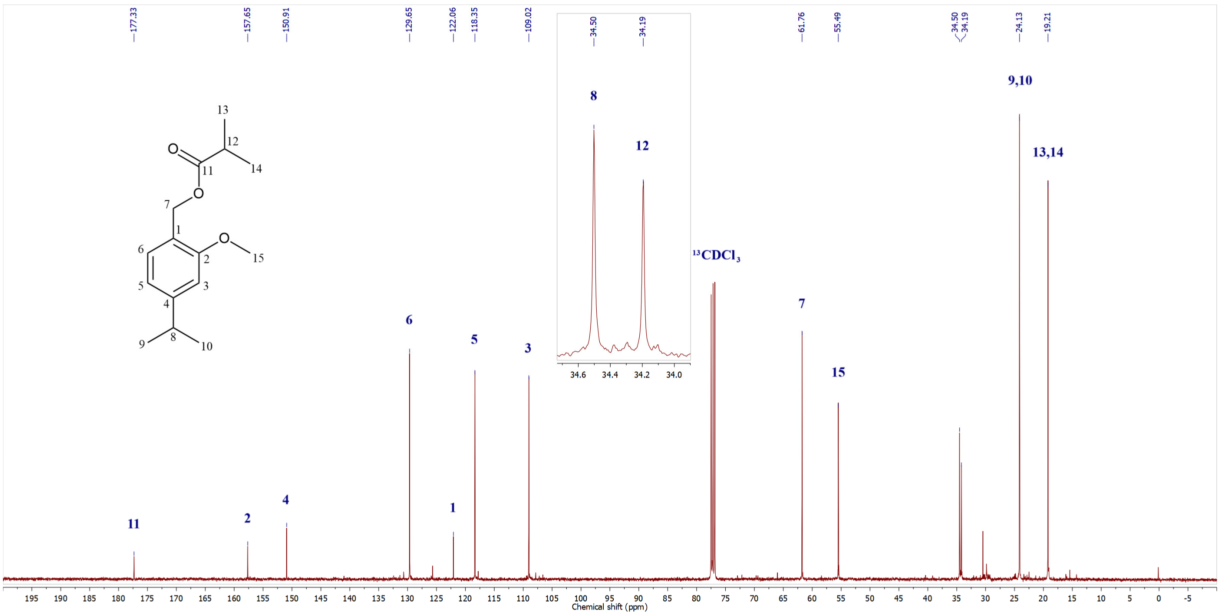

Figure S23. ^{13}C NMR (100.6 MHz, $CDCl_3$) spectrum of 2-methoxycuminyl isobutyrate (**3**)

Table S6. ^{1}H (400 MHz) and ^{13}C (100.6 MHz) NMR data of 2-methoxycuminyl isobutyrate (**3**) (chloroform-*d*), NMR parameters are derived from manual iterative full spin analysis, along with the observed gHMBC and NOESY correlations

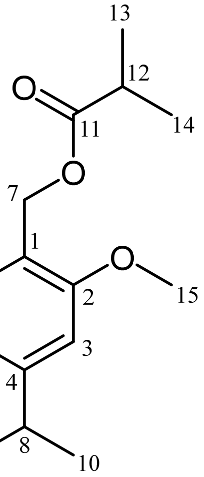

Position	δ_H (m, J (Hz), Integration)	δ_C	HMBC[a]	NOESY[b]
1	/	122.06	/	/
2	/	157.65	/	/
3	6.7494 (ddt, $^4J_{3,5}$ = 1.6, $^4J_{3,8}$ = -0.6, $^5J_{3,7}$ = 0.3, 1 H)	109.02	1,2,5,8	8,9,10,15
4	/	150.91	/	/
5	6.8199 (dddt, $^3J_{5,6}$ = 7.7, $^4J_{3,5}$ = 1.6, $^4J_{5,8}$ = -0.5, $^5J_{5,7}$ = 0.3, 1 H)	118.35	1,3,8	6,8,9,10
6	7.2303 (dtd, $^3J_{5,6}$ = 7.7, $^4J_{6,7}$ = -0.5, $^5J_{6,8}$ = 0.3, 1 H)	129.65	2,4,7	5,7
7	5.1236 (ddd, $^4J_{6,7}$ = -0.5, $^5J_{3,7}$ = 0.3, $^5J_{5,7}$ = 0.3, 2 H)	61.76	1,2,6,11	6,15
8	2.8997 (septddd, $^3J_{8,9/10}$ = 6.9, $^4J_{3,8}$ = -0.6, $^4J_{5,8}$ = -0.5, $^5J_{6,8}$ = 0.3, 1 H)	34.50	3,4,5,9,10	3,5,9,10
9 and 10	1.2565 (d, $^3J_{8,9/10}$ = 6.9, 6 H)	24.13	4,8,9,10	3,5,8
11	/	177.33	/	/
12	2.5921 (sept, $^3J_{12,13/14}$ = 7.0, 1 H)	34.19	11,13,14	13,14

13 and 14	1.1854 (d, $^3J_{12,13/14}$ = 7.0, 6 H)	19.21	11,12,13,14	12
15	3.8389 (s, 3 H)	55.49	3	3,7

[a] gHMBC correlations observed between the hydrogen in this row and the carbon in the listed position.
[b] Cross-peaks observed in the NOESY spectrum.

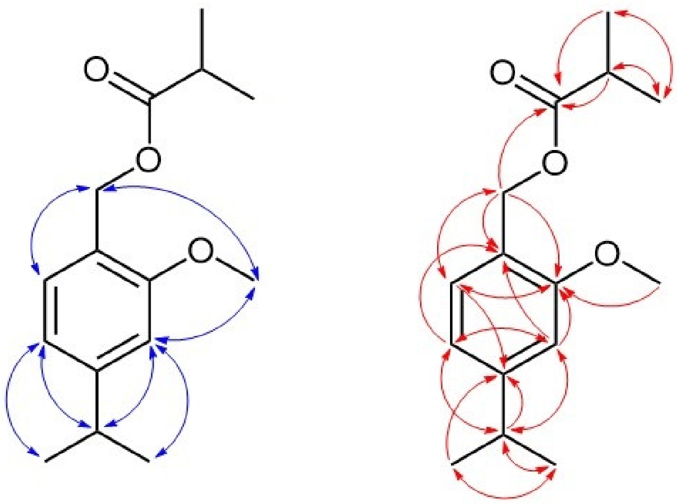

Figure S24. NOESY (blue arrows) and HMBC (red arrows) interactions of 2-methoxycuminyl isobutyrate (**3**)

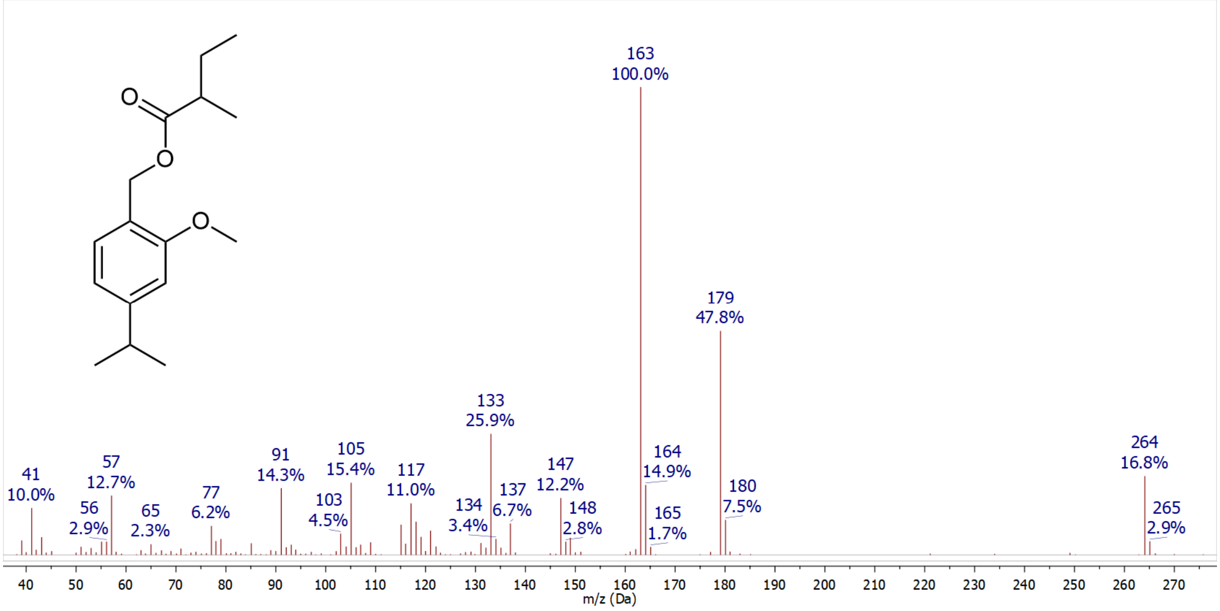

Figure S25. Mass spectrum (EI, 70 eV) of 2-methoxycuminyl 2-methylbutyrate (**4**)

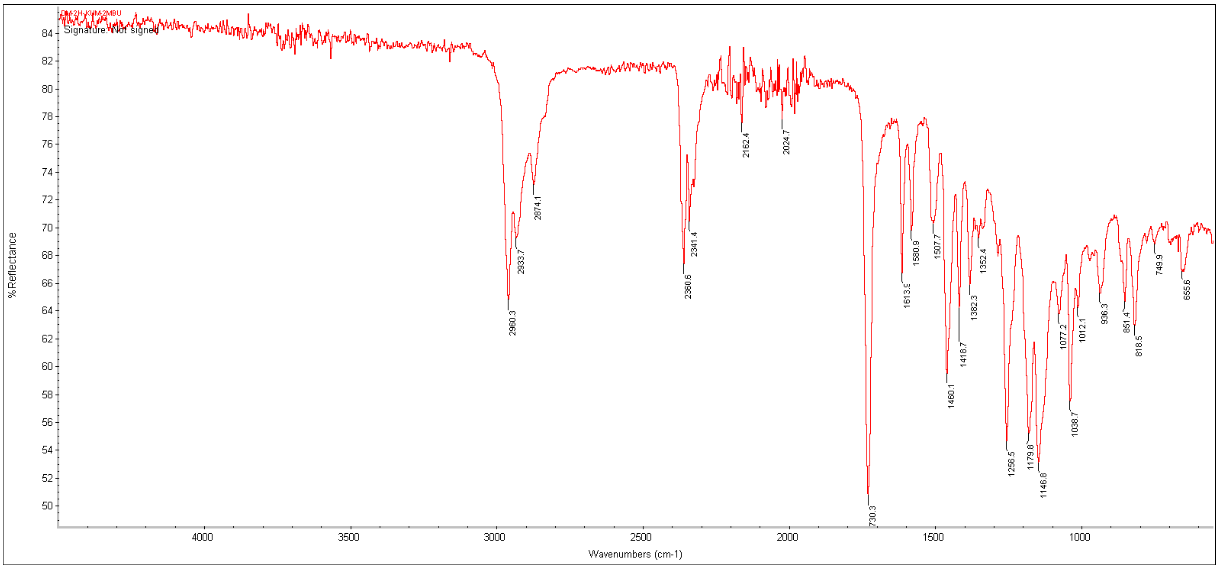

Figure S26. IR spectrum of 2-methoxycuminyl 2-methylbutyrate (**4**)

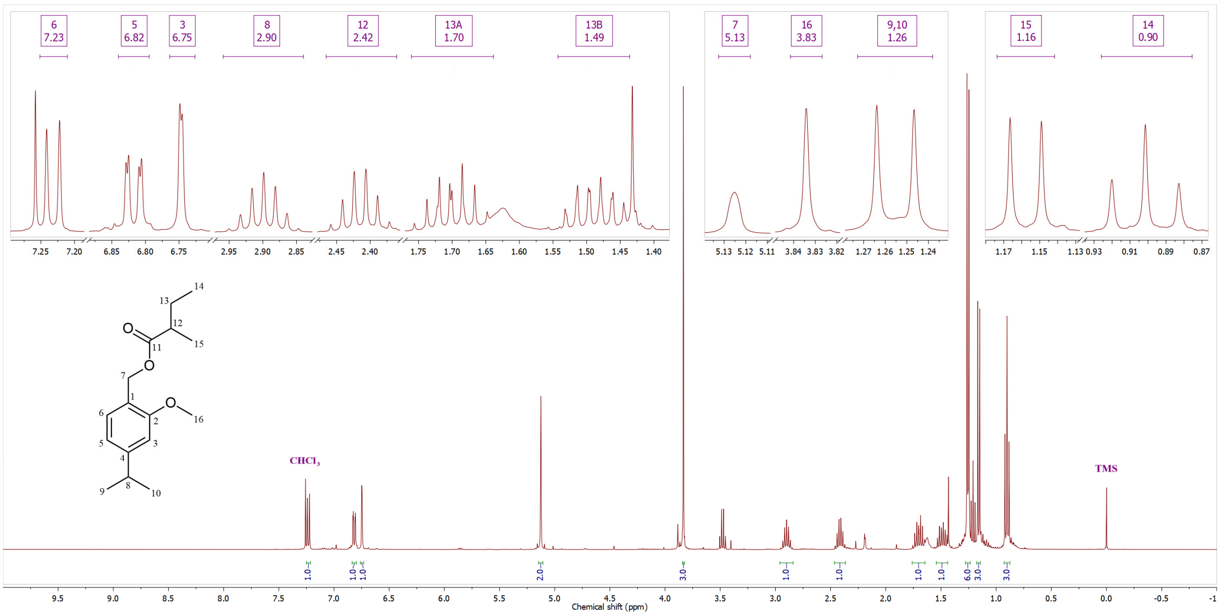

Figure S27. ^{1}H NMR (400 MHz, $CDCl_3$) spectrum of 2-methoxycuminyl 2-methylbutyrate (**4**) and the corresponding expansions with signal assignment

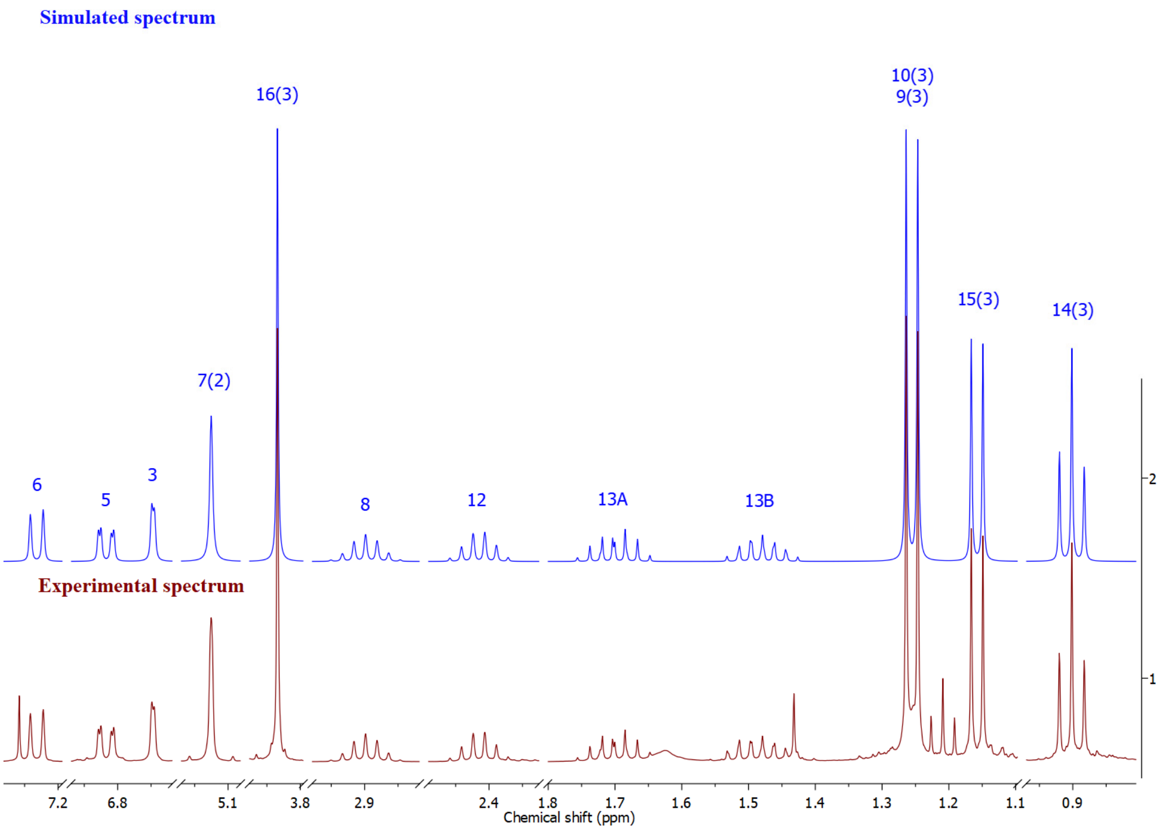

Figure S28. Simulated (manual iterative full spin, MestreNova 11.0.3) and experimental ^{1}H NMR spectrum of 2-methoxycuminyl 2-methylbutyrate (**4**)

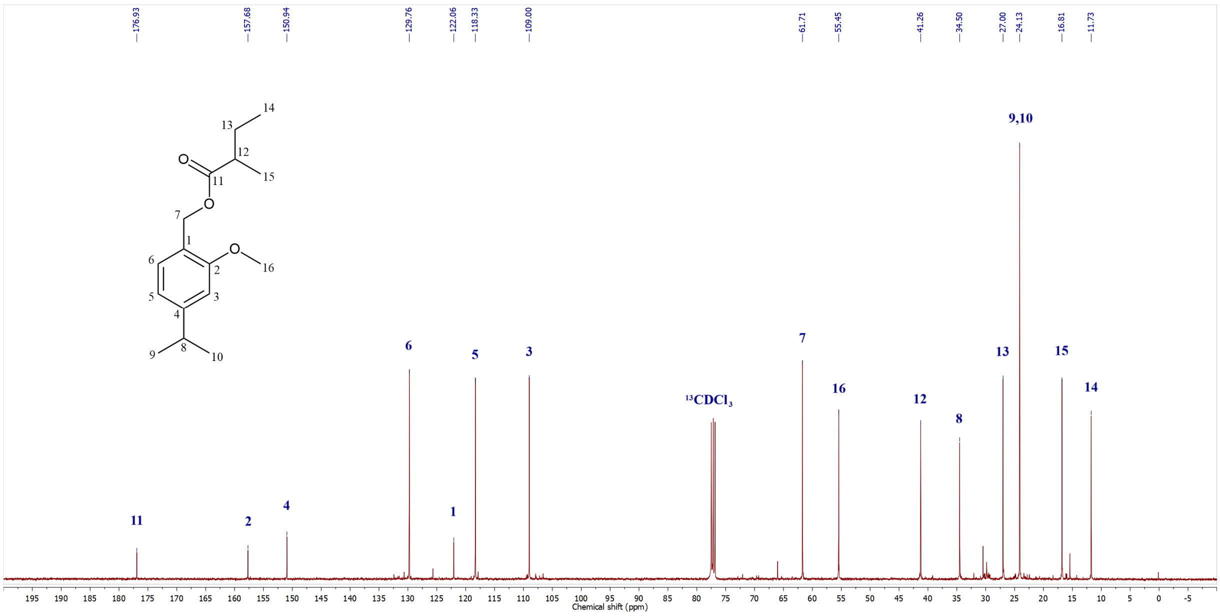

Figure S29. ^{13}C NMR (100.6 MHz, $CDCl_3$) spectrum of 2-methoxycuminyl 2-methylbutyrate (**4**)

Table S7. ^{1}H (400 MHz) and ^{13}C (100.6 MHz) NMR data of 2-methoxycuminyl 2-methylbutyrate (**4**) (chloroform-*d*), NMR parameters are derived from manual iterative full spin analysis, along with the observed gHMBC and NOESY correlations

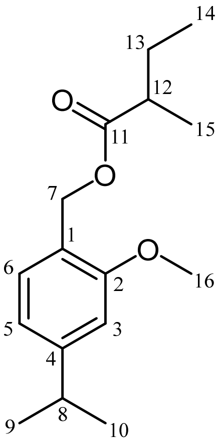

Position	δ_H (m, *J* (Hz), Integration)	δ_C	HMBC[a]	NOESY[b]
1	/	122.06	/	/
2	/	157.68	/	/
3	6.7470 (ddt, $^4J_{3,5}$ = 1.6, $^4J_{3,8}$ = -0.6, $^5J_{3,7}$ = 0.3, 1 H)	109.00	1,2,5,8	8,9,10,16
4	/	150.94	/	/
5	6.8175 (dddt, $^3J_{5,6}$ = 7.7, $^4J_{3,5}$ = 1.6, $^4J_{5,8}$ = -0.5, $^5J_{5,7}$ = 0.3, 1 H)	118.33	1,3,8	6,8,9,10
6	7.2316 (dtd, $^3J_{5,6}$ = 7.7, $^4J_{6,7}$ = -0.5, $^5J_{6,8}$ = 0.3, 1 H)	129.76	2,4,7	5,7
7	5.1252 (ddd, $^4J_{6,7}$ = -0.5, $^5J_{3,7}$ = 0.3, $^5J_{5,7}$ = 0.3, 2 H)	61.71	1,2,6,11	6,16
8	2.8983 (septddd, $^3J_{8,9/10}$ = 6.9, $^4J_{3,8}$ = -0.6, $^4J_{5,8}$ = -0.5, $^5J_{6,8}$ = 0.3, 1 H)	34.50	3,4,5,9,10	3,5,9,10
9 and 10	1.2553 (d, $^3J_{8,9/10}$ = 6.9, 6 H)	24.13	4,8,9,10	3,5,8
11	/	176.93	/	/
12	2.4147 (dqdq, $^3J_{12,13A}$ = 7.5, $^3J_{12,15}$ = 7.0, $^3J_{12,13B}$ = 6.5, $^4J_{12,14}$ = 0.3, 1 H)	41.26	11,13,14,15	13A,13B,14,15
13A	1.7004 (ddq, $^2J_{13A,13B}$ = -13.7, $^3J_{12,13A}$ = 7.5, $^3J_{13A,14}$ = 7.5, 1 H)	27.00	11,12,14,15	12,14,15
13B	1.4801(dqd, $^2J_{13A,13B}$ = -13.7, $^3J_{13B,14}$ = 7.5, $^3J_{12,13B}$ = 6.5, 1 H)	27.00	11,12,14,15	12,14,15
14	0.9017 (ddd, $^3J_{13A,14}$ = 7.5, $^3J_{13B,14}$ = 7.5, $^4J_{12,14}$ = 0.3, 3 H)	11.73	12,13	12,13A,13B
15	1.1578 (d, $^3J_{12,15}$ = 7.0, 3 H)	16.81	11,12,13	12,13A,13B
16	3.8342 (s, 3 H)	55.45	2	3,7

[a] gHMBC correlations observed between the hydrogen in this row and the carbon in the listed position.
[b] Cross-peaks observed in the NOESY spectrum.

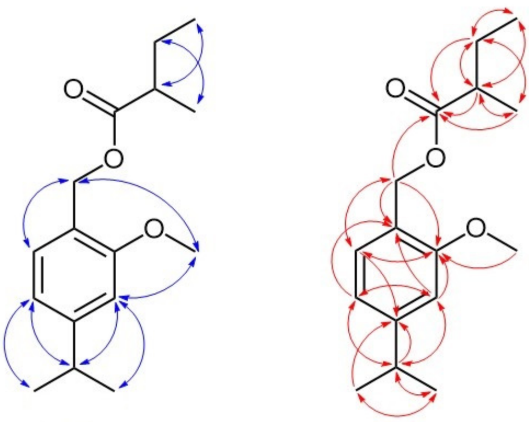

Figure S30. NOESY (blue arrows) and HMBC (red arrows) interactions of 2-methoxycuminyl 2-methylbutyrate (**4**)

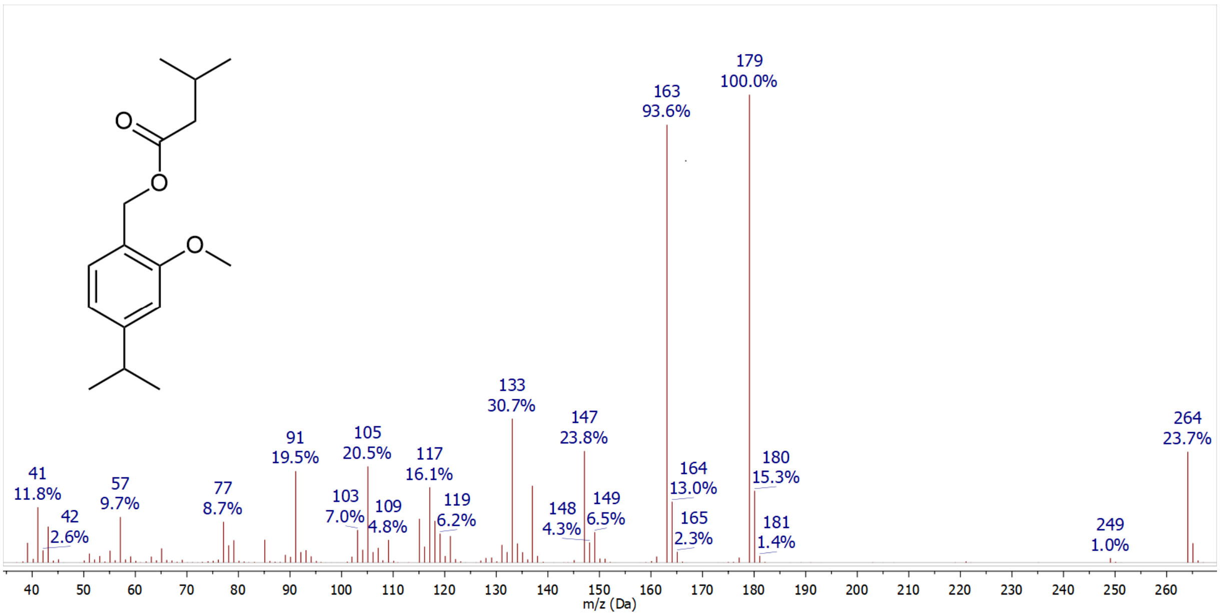

Figure S31. Mass spectrum (EI, 70 eV) of 2-methoxycuminyl isovalerate (**5**)

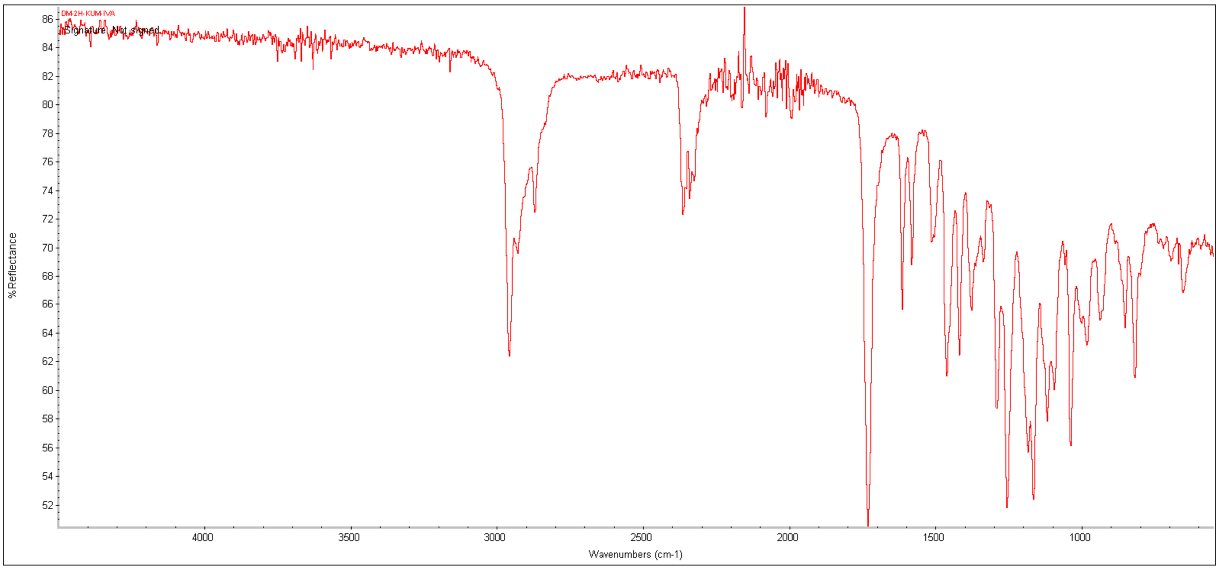

Figure S32. IR spectrum of 2-methoxycuminyl isobutyrate (**5**)

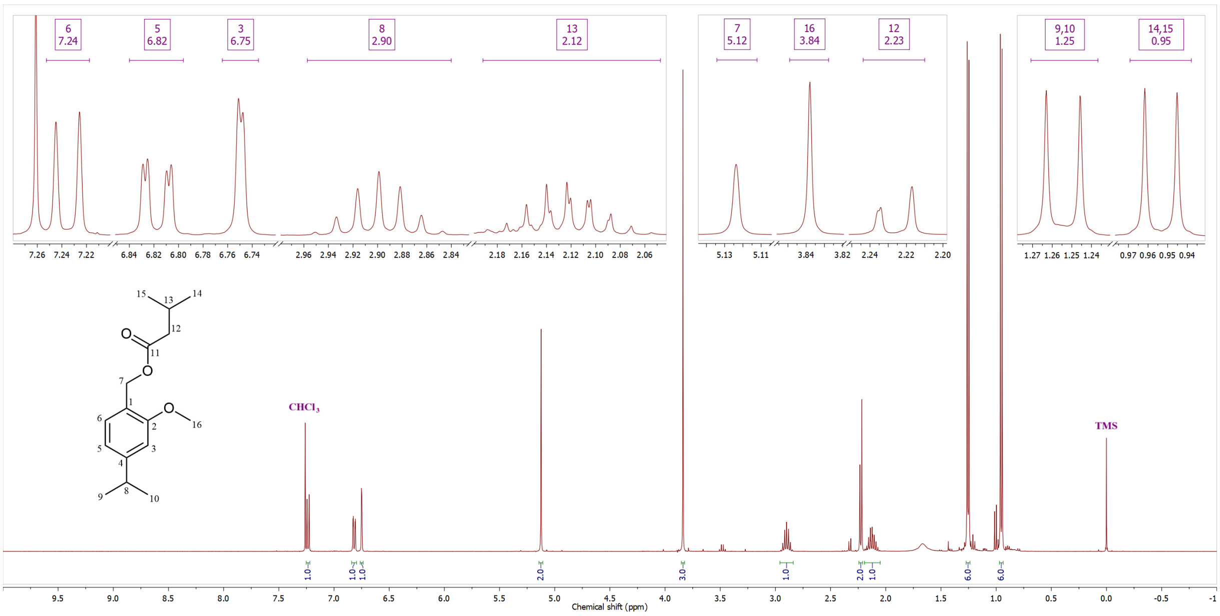

Figure S33. ^{1}H NMR (400 MHz, $CDCl_3$) spectrum of 2-methoxycuminyl isovalerate (**5**) and the corresponding expansions with signal assignment

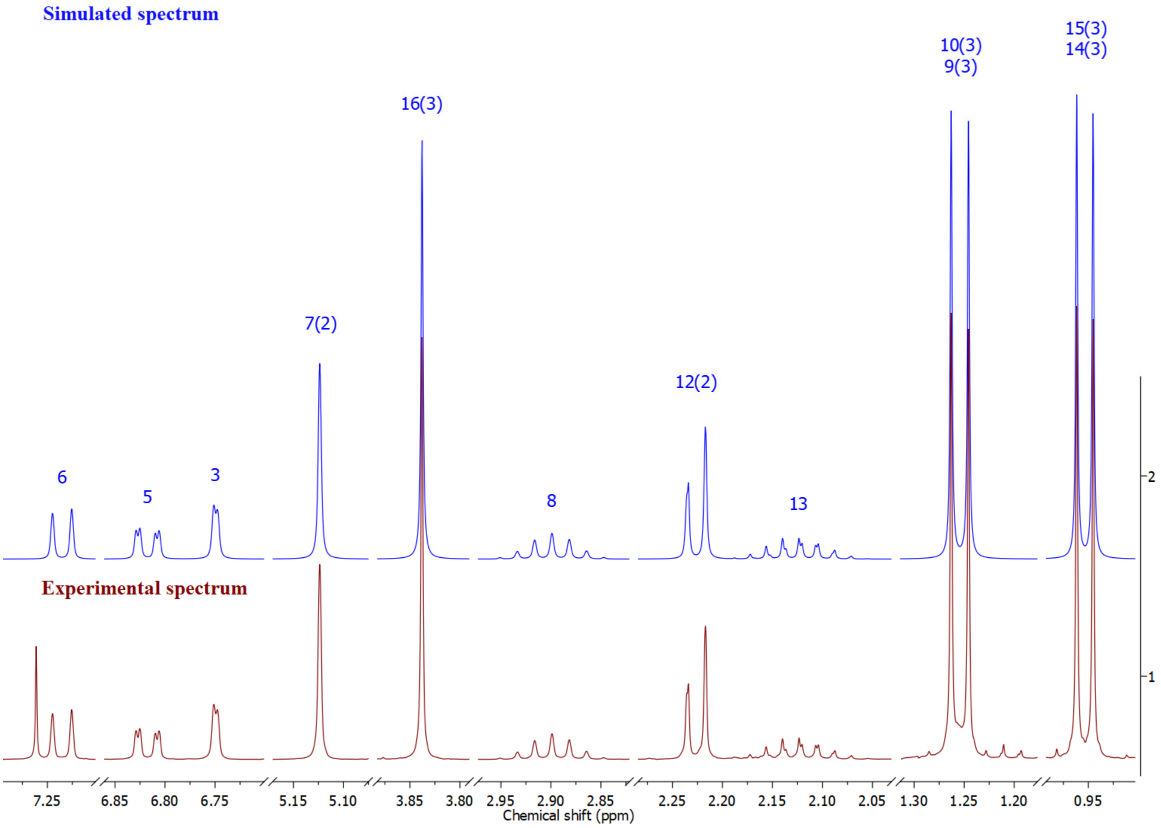

Figure S34. Simulated (manual iterative full spin, MestreNova 11.0.3) and experimental ^{1}H NMR spectrum of 2-methoxycuminyl isovalerate (**5**)

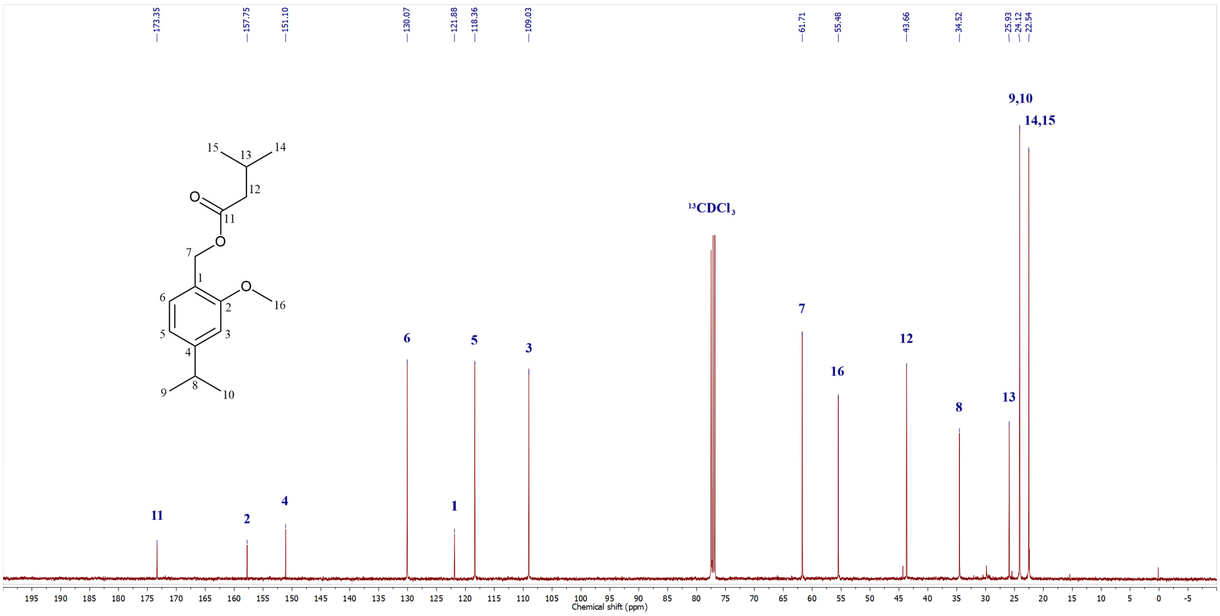

Figure S35. ^{13}C NMR (100.6 MHz, $CDCl_3$) spectrum of 2-methoxycuminyl isovalerate (**5**)

Table S8. ^{1}H (400 MHz) and ^{13}C (100.6 MHz) NMR data of 2-methoxycuminyl isovalerate (**5**) (chloroform-*d*), NMR parameters are derived from manual iterative full spin analysis, along with the observed gHMBC and NOESY correlations

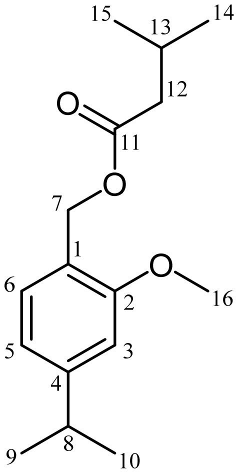

Position	δ_H (m, J (Hz), Integration)	δ_C	HMBC[a]	NOESY[b]
1	/	121.88	/	/
2	/	157.75	/	/
3	6.7494 (ddt, $^4J_{3,5}$ = 1.6, $^4J_{3,8}$ = -0.6, $^5J_{3,7}$ = 0.3, 1 H)	109.03	1,2,5,8	8,9,10,16
4	/	151.10	/	/
5	6.8176 (dddt, $^3J_{5,6}$ = 7.7, $^4J_{3,5}$ = 1.6, $^4J_{5,8}$ = -0.5, $^5J_{5,7}$ = 0.3, 1 H)	118.36	1,3,8	6,8,9,10
6	7.2350 (dtd, $^3J_{5,6}$ = 7.7, $^4J_{6,7}$ = -0.5, $^5J_{6,8}$ = 0.3, 1 H)	130.07	2,4,7	5,7
7	5.1237 (ddd, $^4J_{6,7}$ = -0.5, $^5J_{3,7}$ = 0.3, $^5J_{5,7}$ = 0.3, 2 H)	61.71	1,2,6,11	6,16
8	2.8987 (septddd, $^3J_{8,9/10}$ = 6.9, $^4J_{3,8}$ = -0.6, $^4J_{5,8}$ = -0.5, $^5J_{6,8}$ = 0.3, 1 H)	34.52	3,4,5,9,10	3,5,9,10
9 and 10	1.2544 (d, $^3J_{8,9/10}$ = 6.9, 6 H)	24.12	4,8,9,10	3,5,8
11	/	173.35	/	/

12	2.2252 (d, $^{3}J_{12,13}$ = 7.2, 2 H)	43.66	11,13,14,15	13,14,15
13	2.1232 (tsept, $^{3}J_{12,13}$ = 7.2, $^{3}J_{13,14/15}$ = 6.7, 1 H)	25.93	11,12,14,15	12,14,15
14 and 15	0.9535 (d, $^{3}J_{13,14/15}$ = 6.7, 6 H)	22.54	12,13,14,15	12,13
16	3.8380 (s, 3 H)	55.48	2	3,7

[a] gHMBC correlations observed between the hydrogen in this row and the carbon in the listed position.
[b] Cross-peaks observed in the NOESY spectrum.

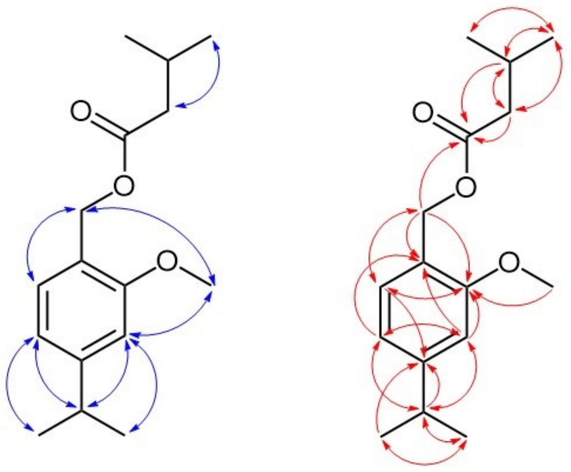

Figure S36. NOESY (blue arrows) and HMBC (red arrows) interactions of 2-methoxycuminyl isovalerate (**5**)

Table S9. ^{1}H (400 MHz) and ^{13}C (100.6 MHz) NMR data of 6-hydroxythymyl isobutyrate (**8**) (chloroform-*d*), NMR parameters are derived from manual iterative full spin analysis, along with the observed gHMBC and NOESY correlations

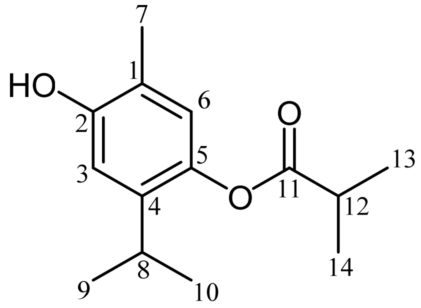

Position	δH (m, J (Hz), Integration)	δC	HMBC[a]	NOESY[b]
1	/	122.10	/	/
2	/	151.80	/	/
3	6.7164 (ddq, $^{4}J_{3,8}$ = -0.6, $^{5}J_{3,6}$ = 0.3, $^{5}J_{3,7}$ = 0.3, 1 H)	112.83	1,2,5,8	9,10,OH
4	/	138.88	/	/
5	/	141.40	/	/
6	6.6855 (qdd, $^{4}J_{6,7}$ = -0.7, $^{5}J_{3,6}$ = 0.3, $^{5}J_{6,8}$ = 0.3, 1 H)	124.26	2,4,5,7	7
7	2.1716 (dd, $^{4}J_{6,7}$ = -0.7, $^{5}J_{3,7}$ = 0.3, 3 H)	15.53	1,2,6	6,OH
8	2.9050 (septdd, $^{3}J_{8,9/10}$ = 6.9, $^{4}J_{3,8}$ = -0.6, $^{5}J_{6,8}$ = 0.3, 1 H)	27.22	3,4,5,9,10	9,10
9 and 10	1.1603 (d, $^{3}J_{8,9/10}$ = 6.9, 6 H)	23.03	4,8,9,10	3,8
OH	4.8221 (brs, 1 H)	/	2	3,7
11	/	176.41	/	/
12	2.8132 (sept, $^{3}J_{13,14/15}$ = 7.0, 1 H)	34.36	11,13,14	13,14
13 and 14	1.3271 (d, $^{3}J_{13,14/15}$ = 7.0, 6 H)	19.18	11,12,13,14	12

[a] gHMBC correlations observed between the hydrogen in this row and the carbon in the listed position.
[b] Cross-peaks observed in the NOESY spectrum.

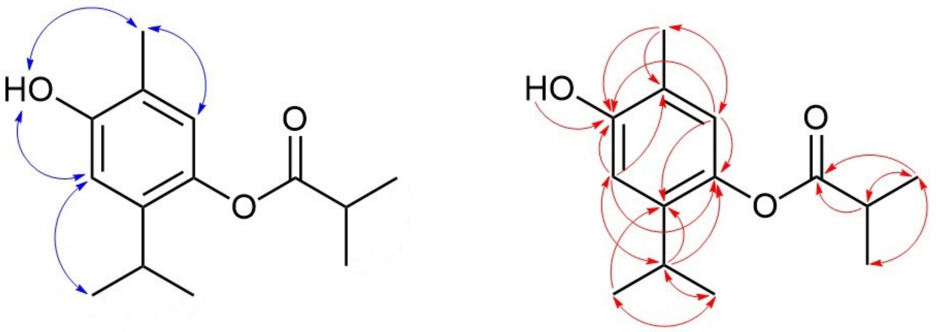

Figure S37. NOESY (blue arrows) and HMBC (red arrows) interactions of 6-hydroxythymyl isobutyrate (**8**)

Table S10. ^{1}H (400 MHz) and ^{13}C (100.6 MHz) NMR data of 6-hydroxythymyl 2-methylbutyrate (**9**) (chloroform-*d*), NMR parameters are derived from manual iterative full spin analysis, along with the observed gHMBC and NOESY correlations

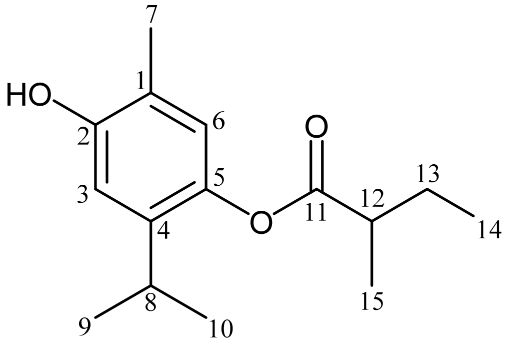

Position	δ_H (m, *J* (Hz), Integration)	δ_C	HMBC[a]	NOESY[b]
1	/	122.08	/	/
2	/	151.79	/	/
3	6.6864 (ddq, $^4J_{3,8}$ = -0.6, $^5J_{3,6}$ = 0.3, $^5J_{3,7}$ = 0.3, 1 H)	112.81	1,2,5,8	9,10,OH
4	/	138.93	/	/
5	/	141.38	/	/
6	6.7059 (qdd, $^4J_{6,7}$ = -0.8, $^5J_{3,6}$ = 0.3, $^5J_{6,8}$ = 0.3, 1 H)	124.26	2,4,5,7	7
7	2.1721 (dd, $^4J_{6,7}$ = -0.8, $^5J_{3,7}$ = 0.3, 3 H)	15.53	1,2,6	6,OH
8	2.9189 (septdd, $^3J_{8,9/10}$ = 6.9, $^4J_{3,8}$ = -0.6, $^5J_{6,8}$ = 0.3, 1 H)	27.16	3,4,5,9,10	9,10
9 and 10	1.1607 (d, $^3J_{8,9/10}$ = 6.9, 6 H)	23.08	4,8,9,10	3,8
OH	4.7275 (brs, 1 H)	/	2	3,7
11	/	175.99	/	/
12	2.6268 (dqdq, $^3J_{12,13A}$ = 7.5, $^3J_{12,15}$ = 7.0, $^3J_{12,13B}$ = 6.5, $^4J_{12,14}$ = 0.3, 1 H)	41.45	11,13,14,15	13A,13B,15
13A	1.8575 (ddq, $^2J_{13A,13B}$ = -13.7, $^3J_{12,13A}$ = 7.5, $^3J_{13A,14}$ = 7.5, 1 H)	26.89	11,12,14,15	12,14,15
13B	1.6132 (dqd, $^2J_{13A,13B}$ = -13.7, $^3J_{13B,14}$ = 7.5, $^3J_{12,13B}$ = 6.5, 1 H)	26.89	11,12,14,15	12,14,15
14	1.0299 (ddd, $^3J_{13A,14}$ = 7.5, $^3J_{13B,14}$ = 7.5, $^4J_{12,14}$ = 0.3, 3 H)	11.89	12,13	13A,13B
15	1.3062 (d, $^3J_{12,15}$ = 7.0, 3 H)	16.88	11,12,13	12,13A,13B

[a] gHMBC correlations observed between the hydrogen in this row and the carbon in the listed position.
[b] Cross-peaks observed in the NOESY spectrum.

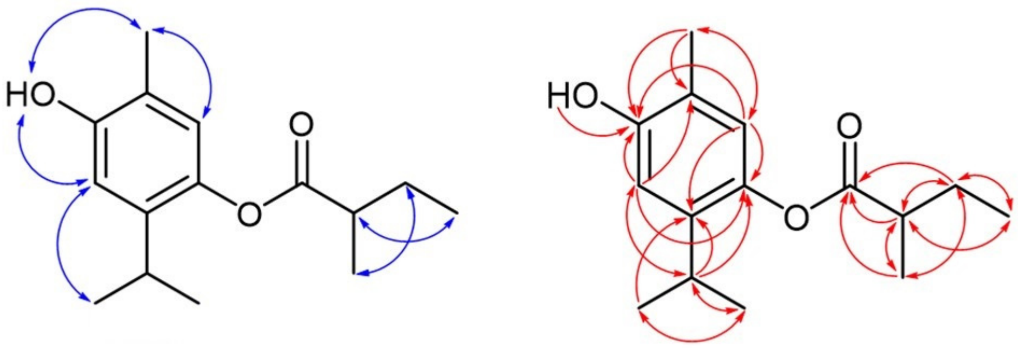

Figure S38. NOESY (blue arrows) and HMBC (red arrows) interactions of 6-hydroxythymyl 2-methylbutyrate (**9**)

Table S11. ^{1}H (400 MHz) and ^{13}C (100.6 MHz) NMR data of 6-isobutyryloxythymol (**10**) (chloroform-*d*), NMR parameters are derived from manual iterative full spin analysis, along with the observed gHMBC and NOESY correlations

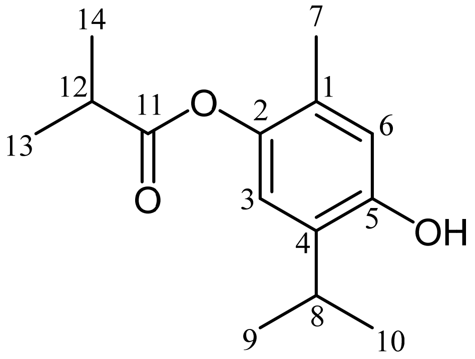

Position	δ_H (m, *J* (Hz), Integration)	δ_C	HMBC[a]	NOESY[b]
1	/	127.61	/	/
2	/	142.77	/	/
3	6.7308 (ddq, $^{4}J_{3,8}$ = -0.6, $^{5}J_{3,6}$ = 0.3, $^{5}J_{3,7}$ = 0.3, 1 H)	119.31	1,2,5,8	9,10
4	/	133.44	/	/
5	/	150.70	/	/
6	6.4482 (qdd, $^{4}J_{6,7}$ = -0.7, $^{5}J_{3,6}$ = 0.3, $^{5}J_{6,8}$ = 0.3, 1 H)	117.58	2,4,5,7	7,OH
7	2.0268 (dd, $^{4}J_{6,7}$ = -0.7, $^{5}J_{3,7}$ = 0.3, 3 H)	15.84	1,2,6	6
8	3.1200 (septdd, $^{3}J_{8,9/10}$ = 6.9, $^{4}J_{3,8}$ = -0.6, $^{5}J_{6,8}$ = 0.3, 1 H)	26.90	3,4,5,9,10	9,10,OH
9 and 10	1.1853 (d, $^{3}J_{8,9/10}$ = 6.9, 6 H)	22.58	4,8,9,10	3,8,OH
OH	5.5035 (brs, 1 H)	/	5	6,8,9,10
11	/	176.49	/	/
12	2.8238 (sept, $^{3}J_{12,13/14}$ = 7.0, 1 H)	34.32	12,14,15	13,14
13 and 14	1.3347 (d, $^{3}J_{12,13/14}$ = 7.0, 6 H)	19.22	12,13,14,15	12

[a] gHMBC correlations observed between the hydrogen in this row and the carbon in the listed position.
[b] Cross-peaks observed in the NOESY spectrum.

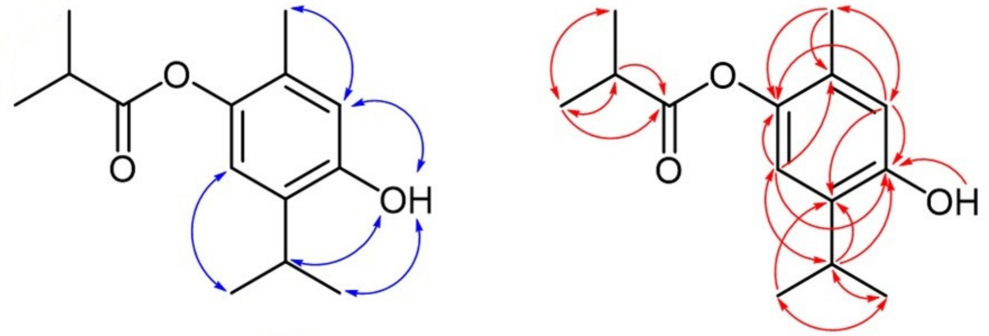

Figure S39. NOESY (blue arrows) and HMBC (red arrows) interactions of 6-isobutyryloxythymol (**10**)

Table S12. ^{1}H (400 MHz) and ^{13}C (100.6 MHz) NMR data of 6-(2-methylbutyryloxy)thymol (**11**) (chloroform-*d*), NMR parameters are derived from manual iterative full spin analysis, along with the observed gHMBC and NOESY correlations

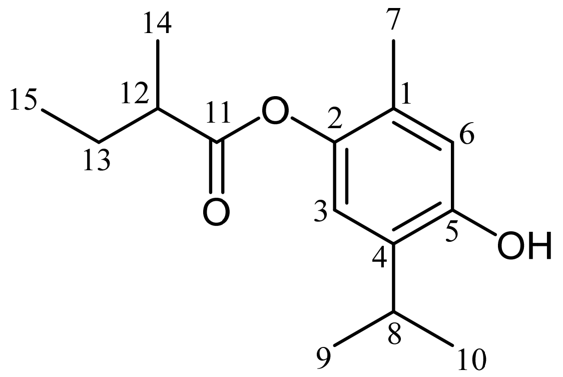

Position	δ_H (m, *J* (Hz), Integration)	δ_C	HMBC[a]	NOESY[b]
1	/	127.39	/	/
2	/	142.70	/	/
3	6.7498 (dq, $^{4}J_{3,8}$ = -0.6, $^{5}J_{3,7}$ = 0.3, 1 H)	119.41	1,2,5,8	9,10
4	/	135.69	/	/
5	/	154.30	/	/
6	6.5653 (qd, $^{4}J_{6,7}$ = -0.7, $^{5}J_{6,8}$ = 0.3, 1 H)	112.75	2,4,5,7	7,OH
7	2.0693 (dd, $^{4}J_{6,7}$ = -0.7, $^{5}J_{3,7}$ = 0.3, 3 H)	16.28	1,2,6	6
8	3.1252 (septdd, $^{3}J_{8,9/10}$ = 6.9, $^{4}J_{3,8}$ = -0.6, $^{5}J_{6,8}$ = 0.3, 1 H)	26.59	3,4,5,9,10	9,10,OH
9 and 10	1.2178 (d, $^{3}J_{8,9/10}$ = 6.9, 6 H)	22.60	4,8,9,10	3,8,OH
OH	4.7304 (brs, 1 H)	55.68	4,5,6	6,8,9,10
11	/	175.25	/	/
12	2.6302 (dqdq, $^{3}J_{12,13A}$ = 7.5, $^{3}J_{12,15}$ = 7.0, $^{3}J_{12,13B}$ = 6.5, $^{4}J_{12,14}$ = 0.3, 1 H)	41.23	11,13,14,15	13A,13B,15
13A	1.8686 (ddq, $^{2}J_{13A,13B}$ = -13.7, $^{3}J_{12,13A}$ = 7.5, $^{3}J_{13A,14}$ = 7.5, 1 H)	26.78	11,13,14,15	12,14,15
13B	1.6175 (dqd, $^{2}J_{13A,13B}$ = -13.7, $^{3}J_{13B,14}$ = 7.5, $^{3}J_{12,13B}$ = 6.5, 1 H)	26.78	11,12,14,15	12,14,15
14	1.0354 (ddd, $^{3}J_{13A,14}$ = 7.5, $^{3}J_{13B,14}$ = 7.5, $^{4}J_{12,14}$ = 0.3, 3 H)	11.78	12,13	13A,13B
15	1.3133 (d, $^{3}J_{12,15}$ = 7.0, 3 H)	16.78	11,12,13	12,13A,13B

[a] gHMBC correlations observed between the hydrogen in this row and the carbon in the listed position.
[b] Cross-peaks observed in the NOESY spectrum.

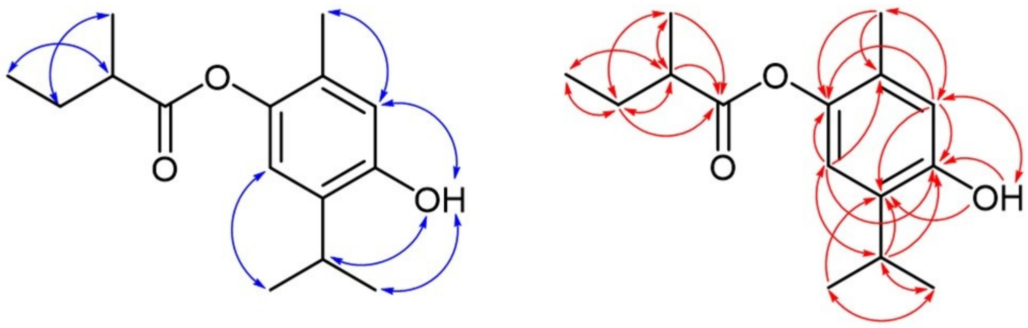

Figure S40. NOESY (blue arrows) and HMBC (red arrows) interactions of 6-(2-methylbutyryloxy)thymol (**11**)

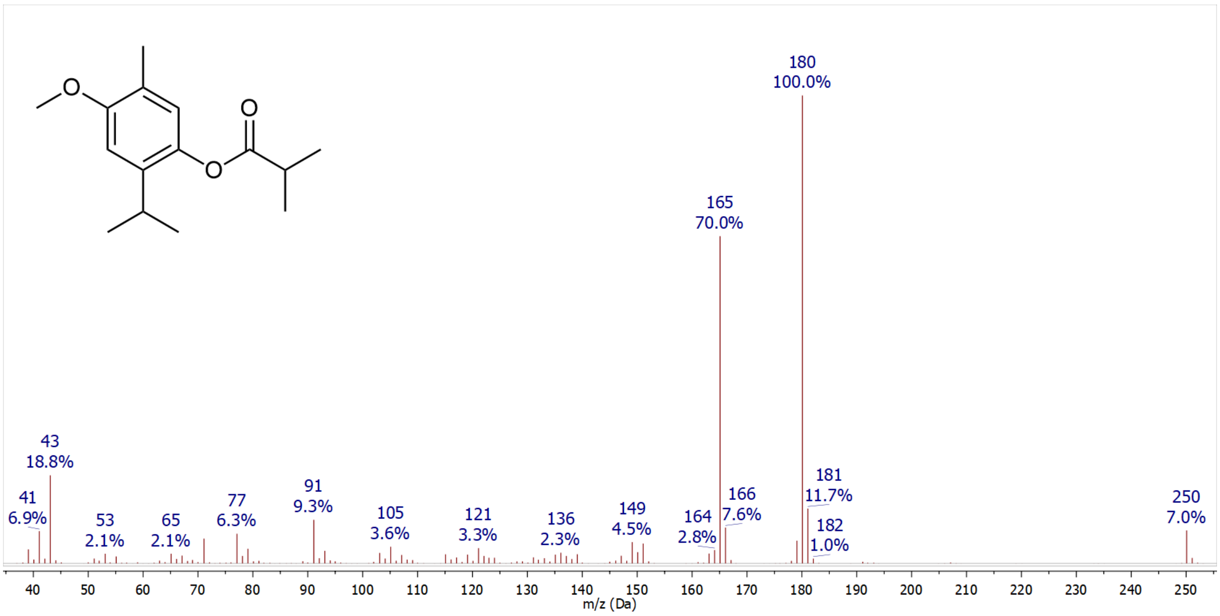

Figure S41. Mass spectrum (EI, 70 eV) of 6-methoxythymyl isobutyrate (**12**)

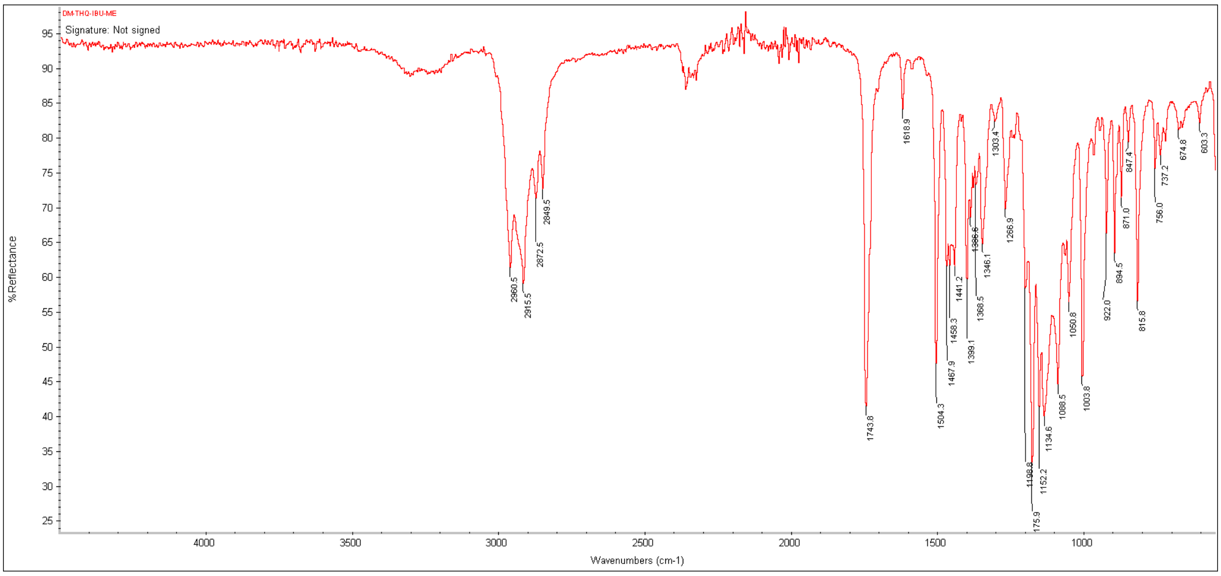

Figure S42. IR spectrum of 6-methoxythymyl isobutyrate (**12**)

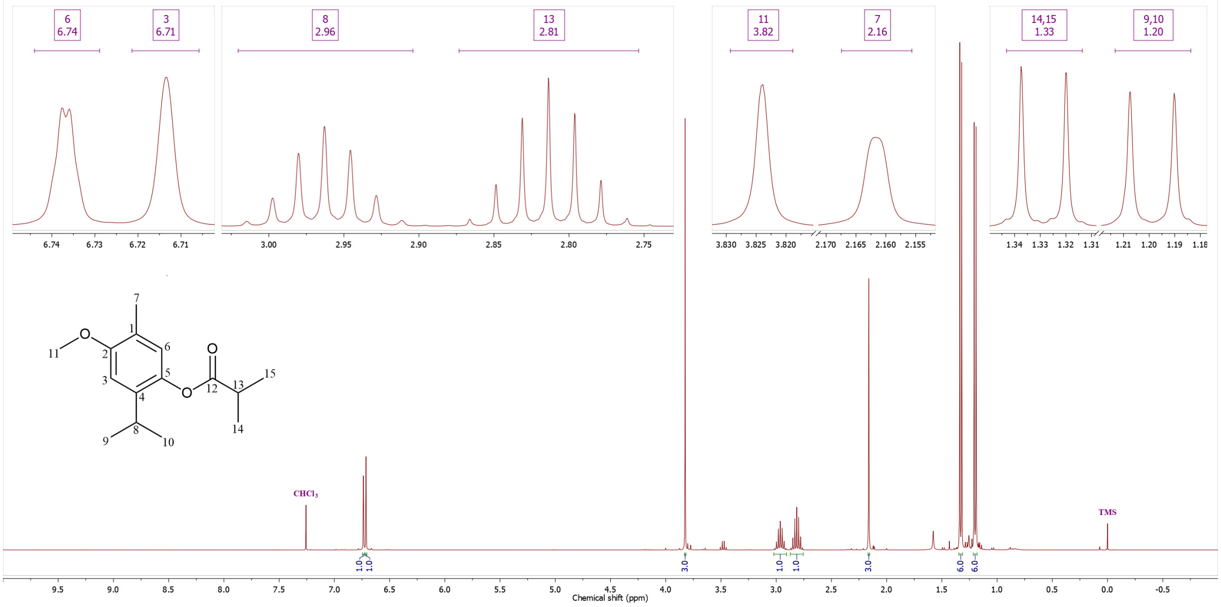

Figure S43. ^{1}H NMR (400 MHz, $CDCl_3$) spectrum of 6-methoxythymyl isobutyrate (**12**) and the corresponding expansions with signal assignment

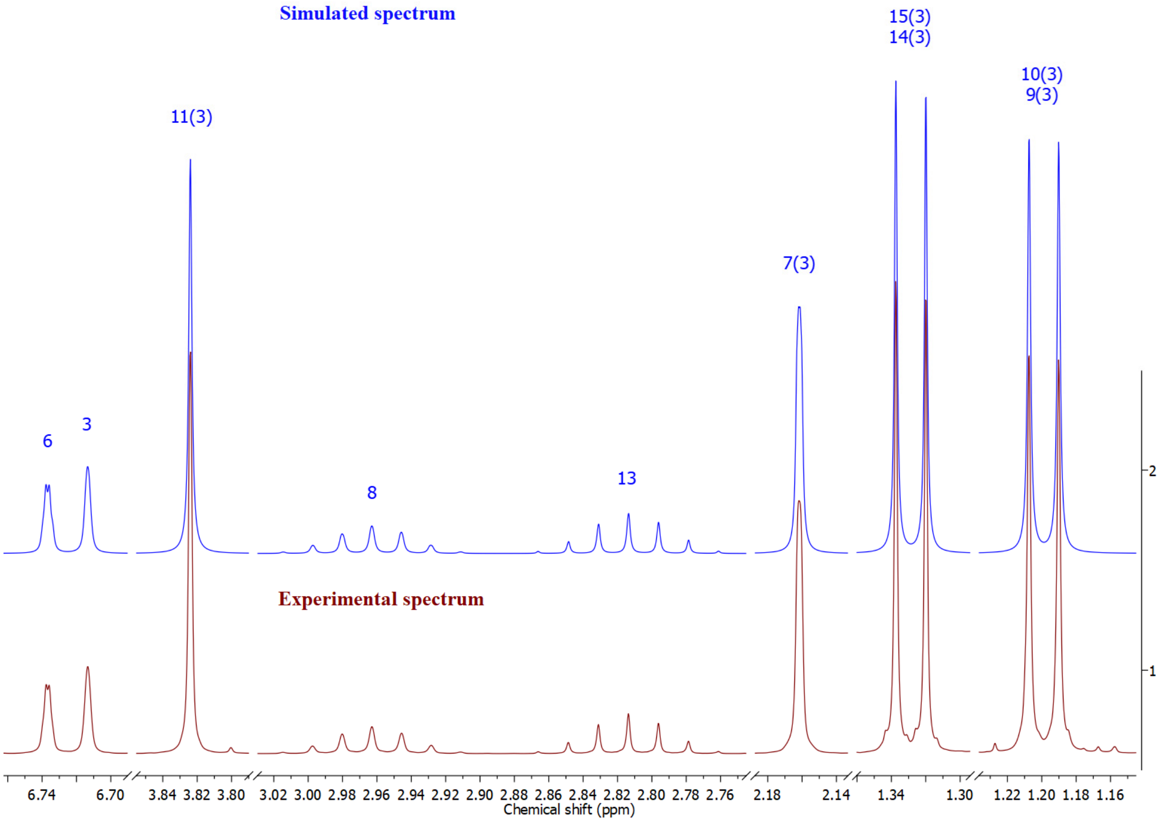

Figure S44. Simulated (manual iterative full spin, MestreNova 11.0.3) and experimental ^{1}H NMR spectrum of 6-methoxythymyl isobutyrate (**12**)

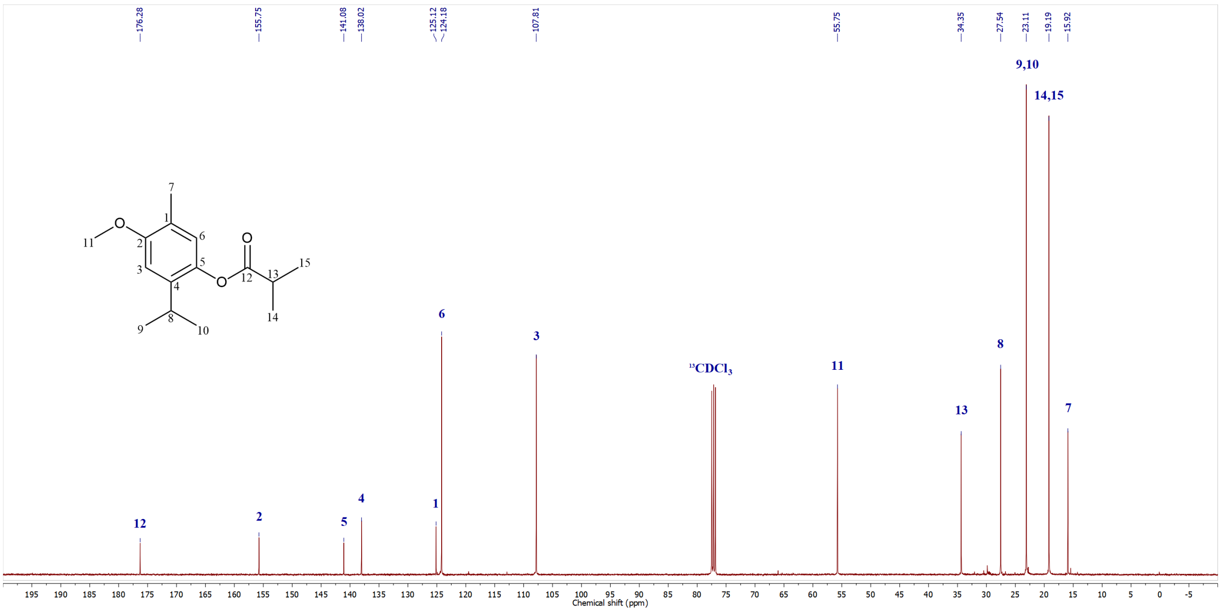

Figure S45. ^{13}C NMR (100.6 MHz, CDCl$_3$) spectrum of 6-methoxythymyl isobutyrate (**12**)

Table S13. ^{1}H (400 MHz) and ^{13}C (100.6 MHz) NMR data of 6-methoxythymyl isobutyrate (**12**) (chloroform-*d*), NMR parameters are derived from manual iterative full spin analysis, along with the observed gHMBC and NOESY correlations

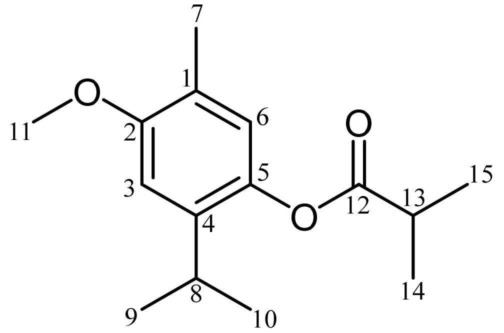

Position	δ_H (m, *J* (Hz), Integration)	δ_C	HMBC[a]	NOESY[b]
1	/	125.12	/	/
2	/	155.75	/	/
3	6.7135 (dq, $^{4}J_{3,8}$ = -0.5, $^{5}J_{3,7}$ = 0.4, 1 H)	107.81	1,2,5,8	9,10,11
4	/	138.02	/	/
5	/	141.08	/	/
6	6.7368 (q, $^{4}J_{6,7}$ = -0.8, 1 H)	124.18	2,4,5,7	7
7	2.1616 (dd, $^{4}J_{6,7}$ = -0.8, $^{5}J_{3,7}$ = 0.4, 3 H)	15.92	1,2,6	6
8	2.9627 (septd, $^{3}J_{8,9/10}$ = 6.9, $^{4}J_{3,8}$ = -0.5, 1 H)	27.54	3,4,5,9,10	9,10
9 and 10	1.1988 (d, $^{3}J_{8,9/10}$ = 6.9, 6 H)	23.11	4,8,9,10	3,8
11	3.8240 (s, 3H)	55.75	2	3
12	/	176.28	/	/
13	2.8133 (sept, $^{3}J_{13,14/15}$ = 7.0, 1 H)	34.35	12,14,15	14,15
14 and 15	1.3287 (d, $^{3}J_{13,14/15}$ = 7.0, 6 H)	19.19	12,13,14,15	13

[a] gHMBC correlations observed between the hydrogen in this row and the carbon in the listed position.
[b] Cross-peaks observed in the NOESY spectrum.

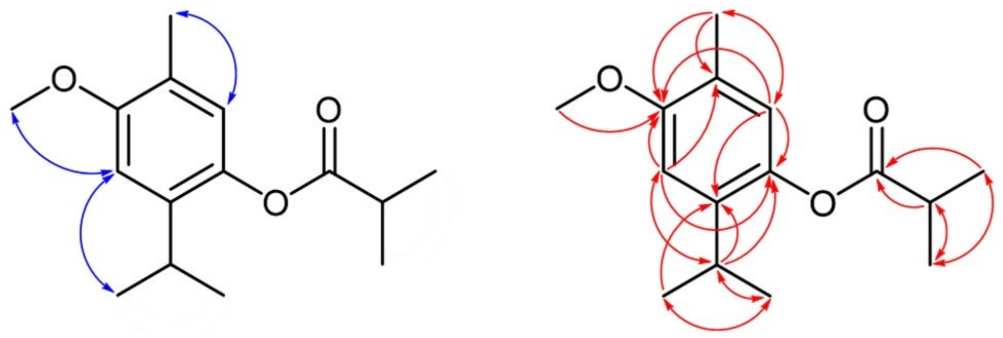

Figure S46. NOESY (blue arrows) and HMBC (red arrows) interactions of 6-methoxythymyl isobutyrate (**12**)

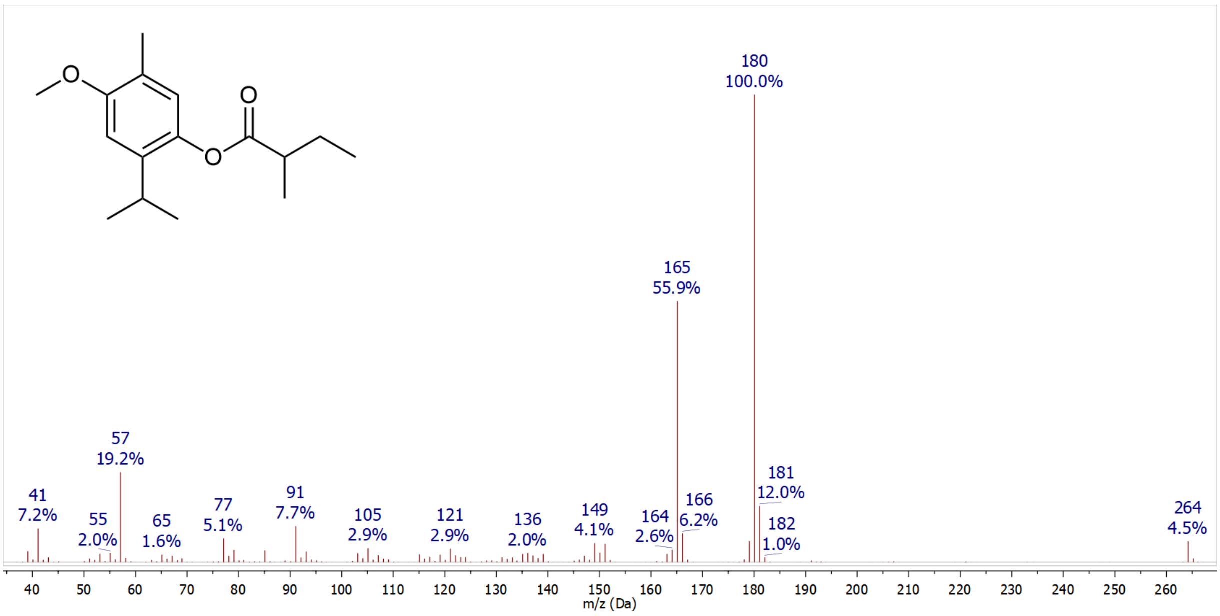

Figure S47. Mass spectrum (EI, 70 eV) of 6-methoxythymyl 2-methylbutyrate (**13**)

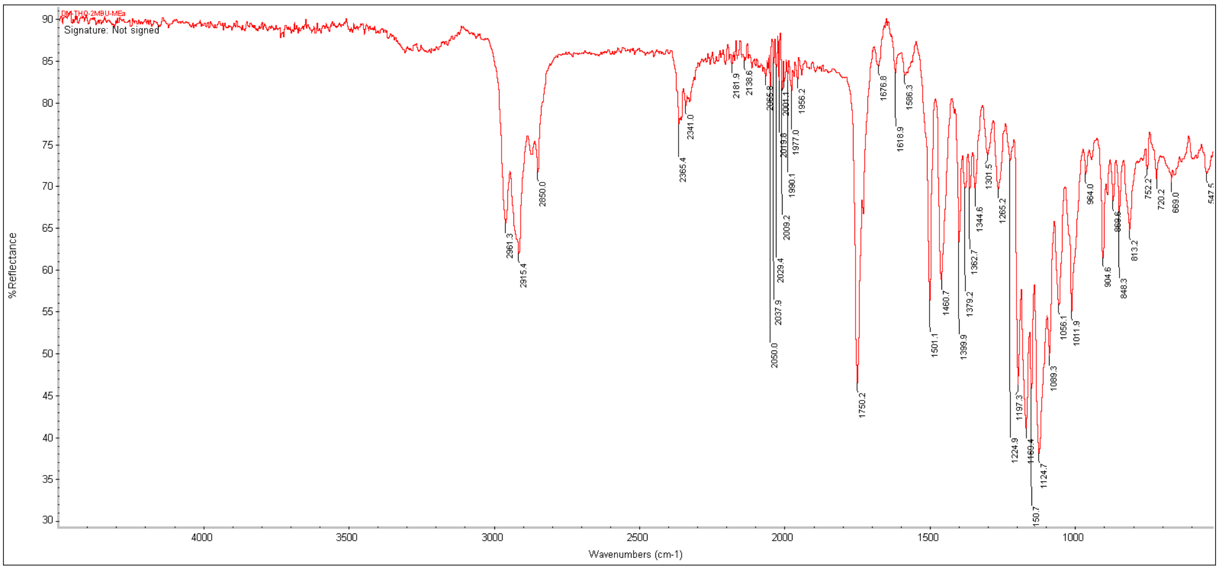

Figure S48. IR spectrum of 6-methoxythymyl 2-methylbutyrate (**13**)

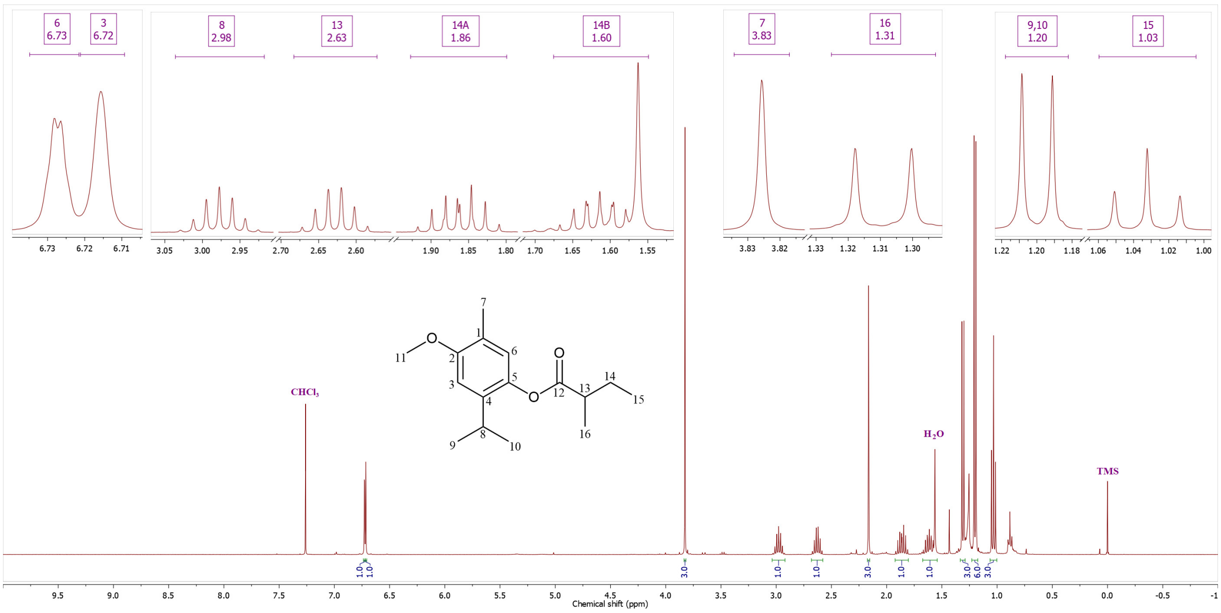

Figure S49. ^{1}H NMR (400 MHz, $CDCl_3$) spectrum of 6-methoxythymyl 2-methylbutyrate (**13**) and the corresponding expansions with signal assignment

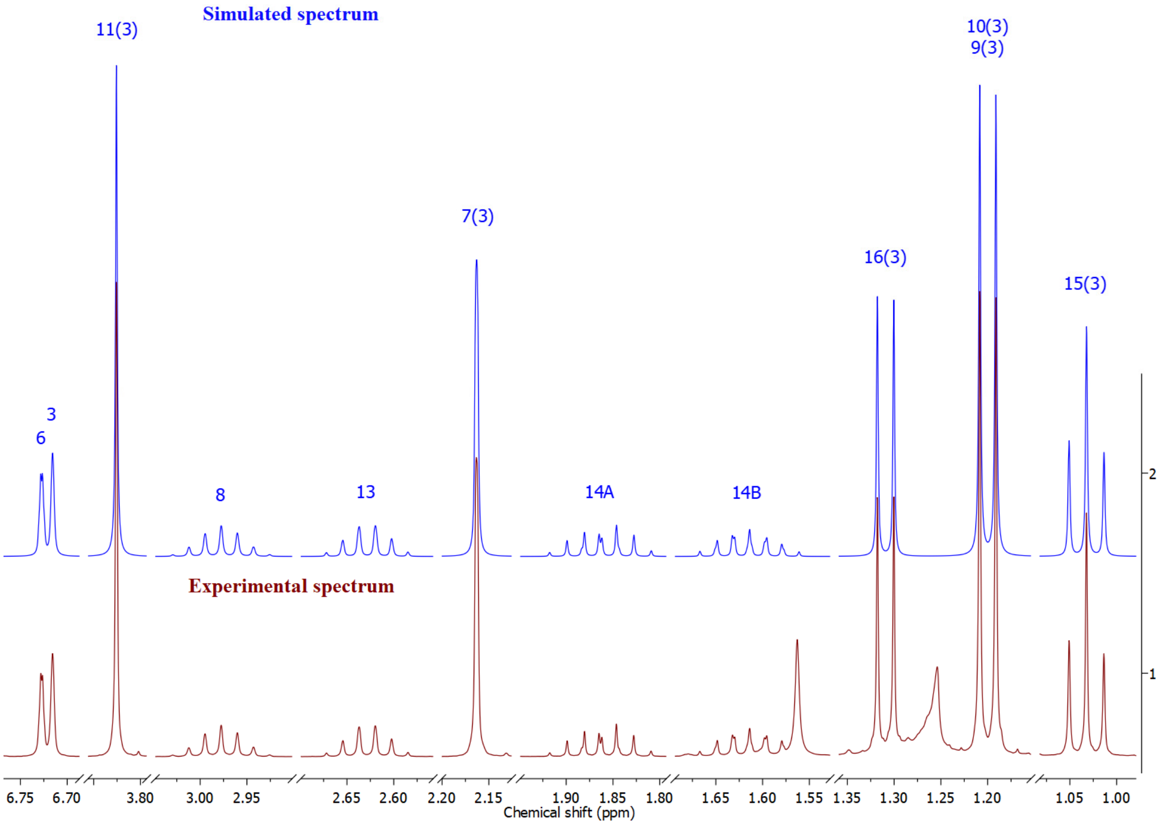

Figure S50. Simulated (manual iterative full spin, MestreNova 11.0.3) and experimental ^{1}H NMR spectrum of 6-methoxythymyl 2-methylbutyrate (**13**)

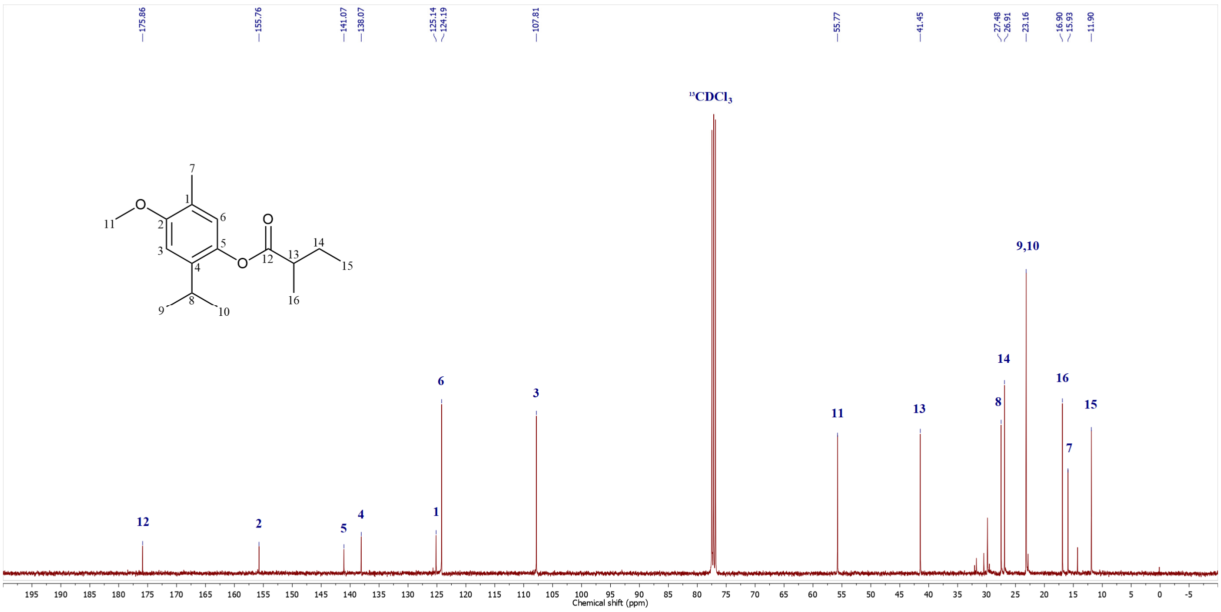

Figure S51. ^{13}C NMR (100.6 MHz, CDCl$_3$) spectrum of 6-methoxythymyl 2-methylbutyrate (**13**)

Table S14. ^{1}H (400 MHz) and ^{13}C (100.6 MHz) NMR data of 6-methoxythymyl 2-methylbutyrate (**13**) (chloroform-*d*), NMR parameters are derived from manual iterative full spin analysis, along with the observed gHMBC and NOESY correlations

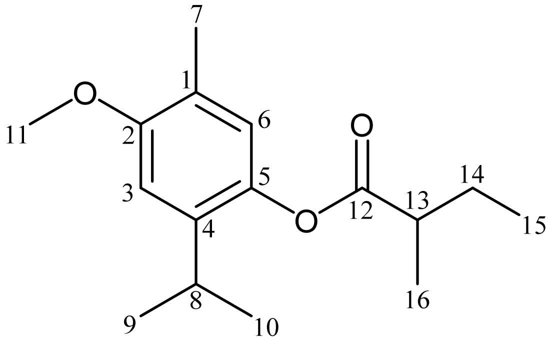

Position	δ_H (m, *J* (Hz), Integration)	δ_C	HMBC[a]	NOESY[b]
1	/	125.14	/	/
2	/	155.76	/	/
3	6.7157 (dq, $^4J_{3,8}$ = -0.6, $^5J_{3,7}$ = 0.4, 1 H)	107.81	1,2,5,8	9,10,11
4	/	138.07	/	/
5	/	141.07	/	/
6	6.7274 (q, $^4J_{6,7}$ = -0.8, 1 H)	124.19	2,4,5,7	7
7	2.1631 (dd, $^4J_{6,7}$ = -0.8, $^5J_{3,7}$ = 0.4, 3 H)	15.93	1,2,6	6
8	2.9772 (septd, $^3J_{8,9/10}$ = 6.9, $^4J_{3,8}$ = -0.6, 1 H)	27.48	3,4,5,9,10	9,10
9 and 10	1.1999 (d, $^3J_{8,9/10}$ = 6.9, 6 H)	23.16	4,8,9,10	3,8
11	3.8256 (s, 3 H)	55.77	2	3
12	/	175.86	/	/
13	2.6280 (dqdq, $^3J_{13,14A}$ = 7.5, $^3J_{13,16}$ = 7.0, $^3J_{13,14B}$ = 6.5, $^4J_{13,15}$ = 0.3, 1 H)	41.45	12,14,15,16	14A,14B,16
14A	1.8620 (ddq, $^2J_{14A,14B}$ = -13.7, $^3J_{13,14A}$ = 7.5, $^3J_{14A,15}$ = 7.5, 1 H)	26.91	12,13,15,16	13,15,16
14B	1.6148 (dqd, $^2J_{14A,14B}$ = -13.7, $^3J_{14B,15}$ = 7.5, $^3J_{13,14B}$ = 6.5, 1 H)	26.91	12,13,15,16	13,15,16
15	1.0325 (ddd, $^3J_{14A,15}$ = 7.5, $^3J_{14B,15}$ = 7.5, $^4J_{13,15}$ = 0.3, 3 H)	11.90	13,14	14A,14B

16	1.3092 (d, $^{3}J_{13,16}$ = 7.0, 3 H)	16.90	12,13,14	13,14A,14B

[a] gHMBC correlations observed between the hydrogen in this row and the carbon in the listed position.
[b] Cross-peaks observed in the NOESY spectrum.

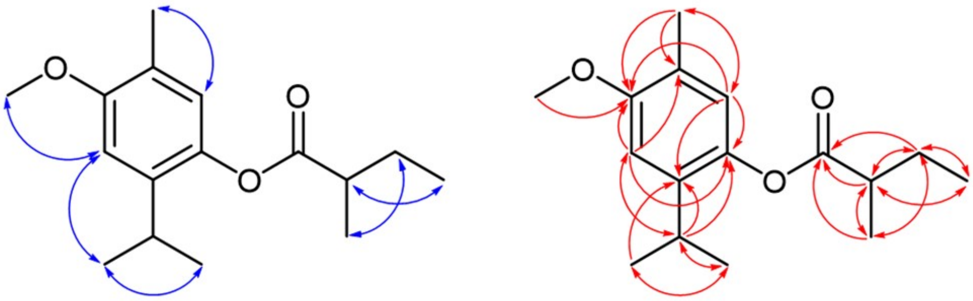

Figure S52. NOESY (blue arrows) and HMBC (red arrows) interactions of 6-methoxythymyl 2-methylbutyrate (**13**)

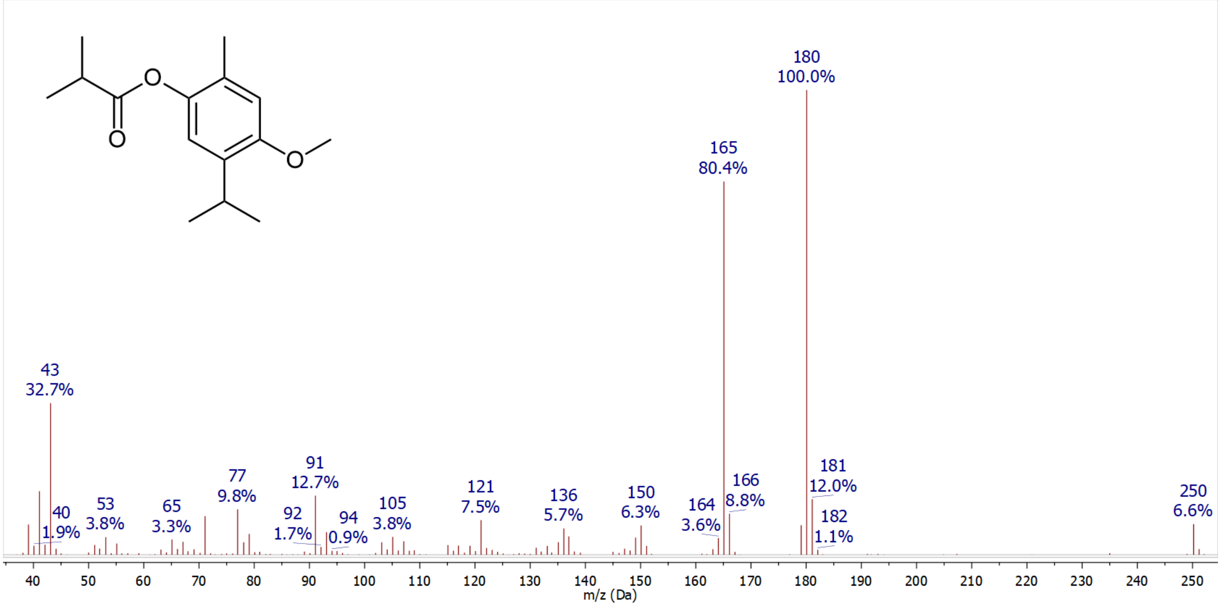

Figure S53. Mass spectrum (EI, 70 eV) of 6-isobutyryloxythymyl methyl ether (**14**)

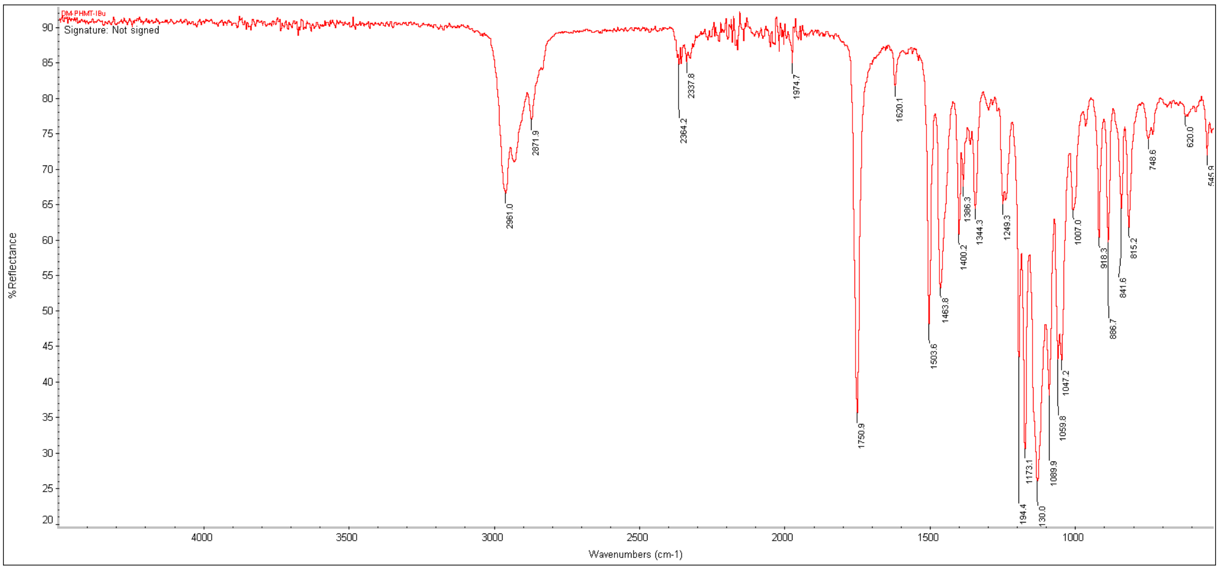

Figure S54. IR spectrum of 6-isobutyryloxythymyl methyl ether (**14**)

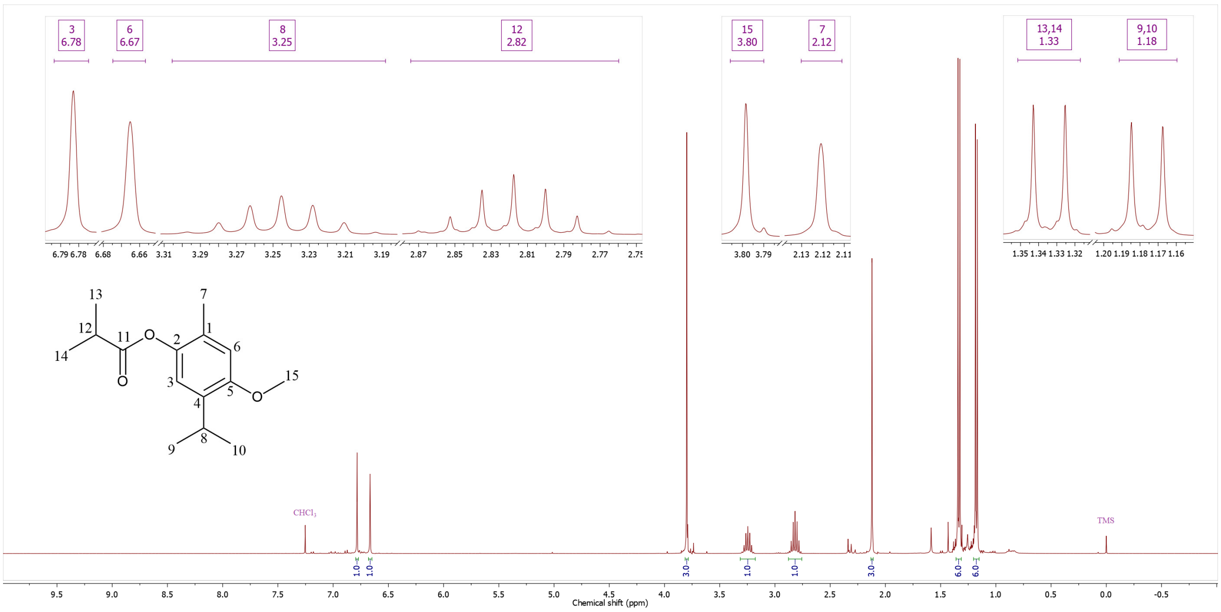

Figure S55. ^{1}H NMR (400 MHz, $CDCl_3$) spectrum of 6-isobutyryloxythymyl methyl ether (**14**) and the corresponding expansions with signal assignment

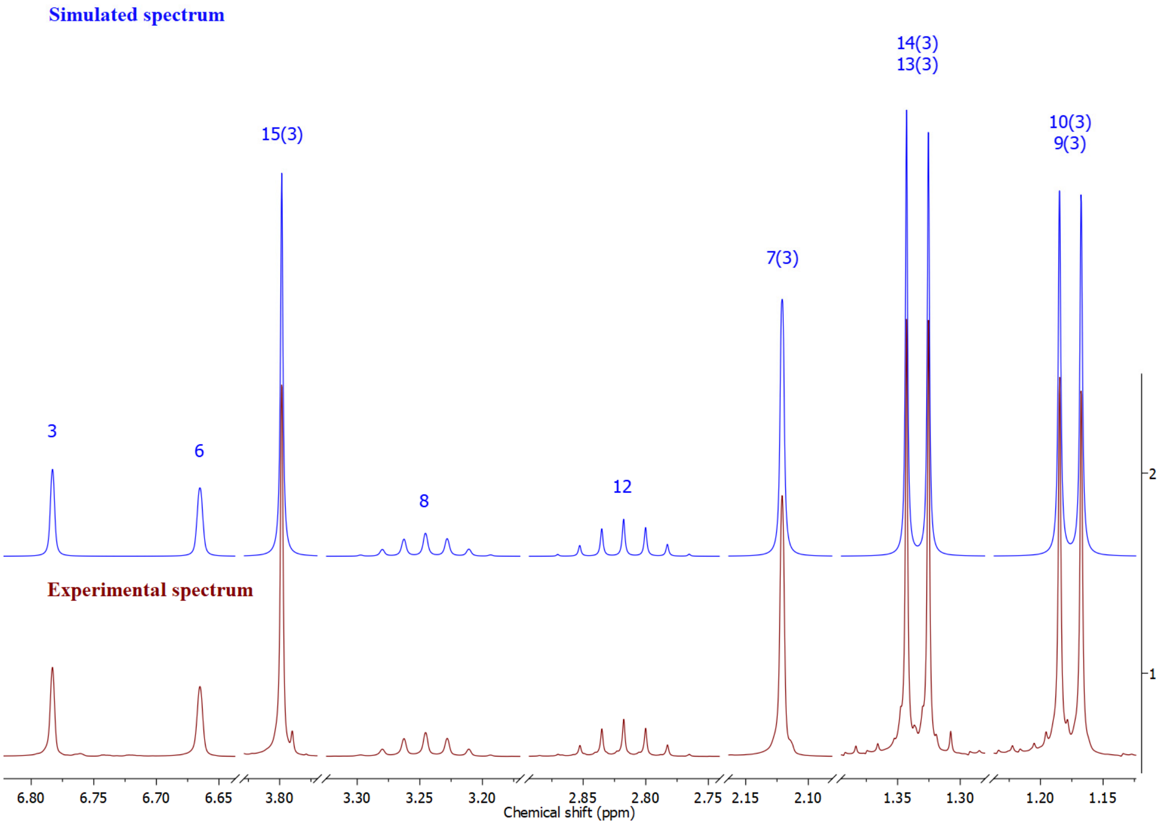

Figure S56. Simulated (manual iterative full spin, MestreNova 11.0.3) and experimental ^{1}H NMR spectrum of 6-isobutyryloxythymyl methyl ether (**14**)

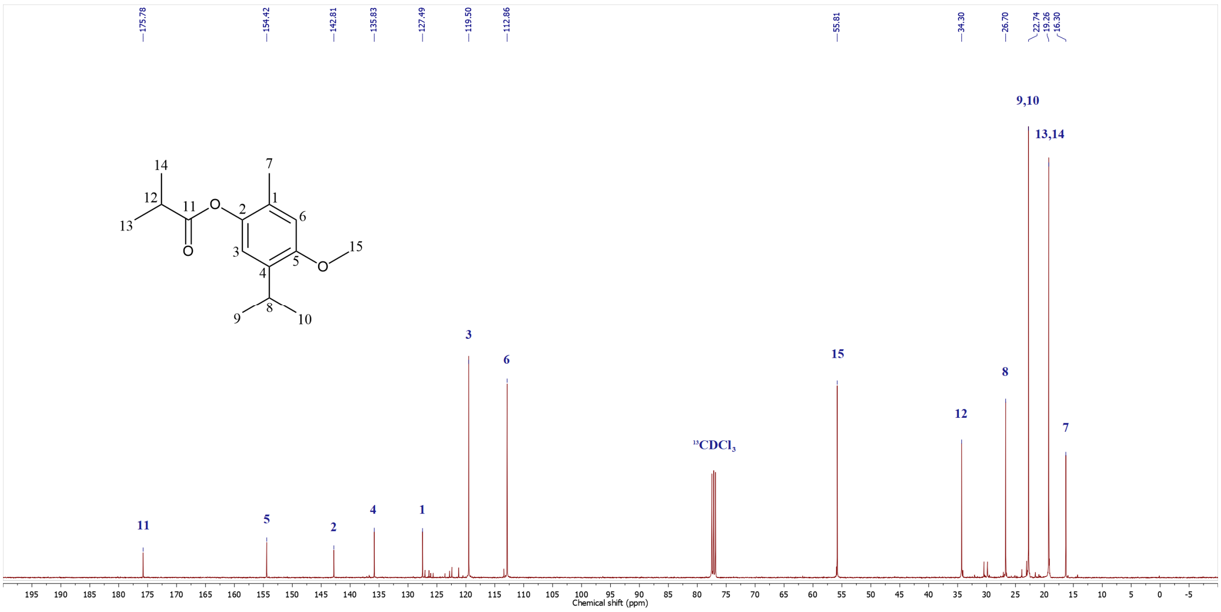

Figure S57. ^{13}C NMR (100.6 MHz, CDCl$_3$) spectrum of 6-isobutyryloxythymyl methyl ether (**14**)

Table S15. ^{1}H (400 MHz) and ^{13}C (100.6 MHz) NMR data of 6-isobutyryloxythymyl methyl ether (**14**) (chloroform-*d*), NMR parameters are derived from manual iterative full spin analysis, along with the observed gHMBC and NOESY correlations

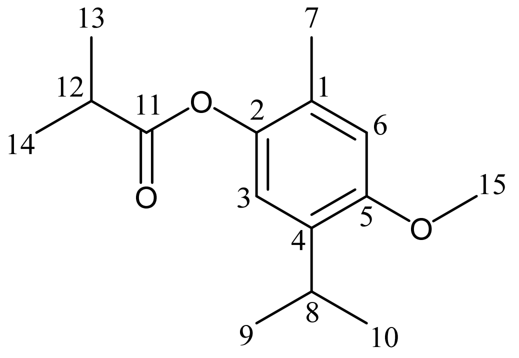

Position	δ_H (m, *J* (Hz), Integration)	δ_C	HMBC[a]	NOESY[b]
1	/	127.49	/	/
2	/	142.81	/	/
3	6.7830 (ddq, $^{4}J_{3,8}$ = -0.6, $^{5}J_{3,6}$ = 0.3, $^{5}J_{3,7}$ = 0.3, 1 H)	119.50	1,2,5,8	9,10
4	/	135.83	/	/
5	/	154.42	/	/
6	6.6652 (qdd, $^{4}J_{6,7}$ = -0.7, $^{5}J_{3,6}$ = 0.3, $^{5}J_{6,8}$ = 0.3, 1 H)	112.86	2,4,5,7	7,15
7	2.1210 (dd, $^{4}J_{6,7}$ = -0.7, $^{5}J_{3,7}$ = 0.3, 3 H)	16.30	1,2,6	6
8	3.2452 (septdd, $^{3}J_{8,9/10}$ = 6.9, $^{4}J_{3,8}$ = -0.6, $^{5}J_{6,8}$ = 0.3, 1 H)	26.70	3,4,5,9,10	9,10
9 and 10	1.1762 (d, $^{3}J_{8,9/10}$ = 6.9, 6 H)	22.74	4,8,9,10	3,8,15
11	/	175.78	/	/
12	2.8174 (sept, $^{3}J_{12,13/14}$ = 7.0, 1 H)	34.30	11,13,14	13,14
13 and 14	1.3342 (d, $^{3}J_{12,13/14}$ = 7.0, 6 H)	19.26	11,12,13,14	12
15	3.7984 (s, 3H)	55.81	5	6,9,10

[a] gHMBC correlations observed between the hydrogen in this row and the carbon in the listed position.
[b] Cross-peaks observed in the NOESY spectrum.

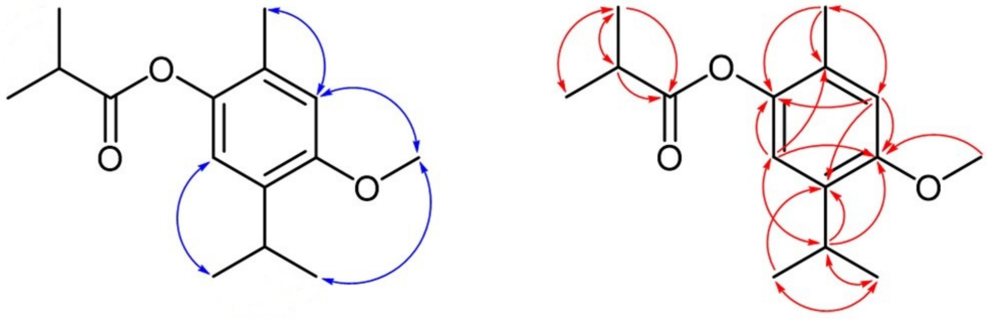

Figure S58. NOESY (blue arrows) and HMBC (red arrows) interactions of 6-isobutyryloxythymyl methyl ether (**14**)

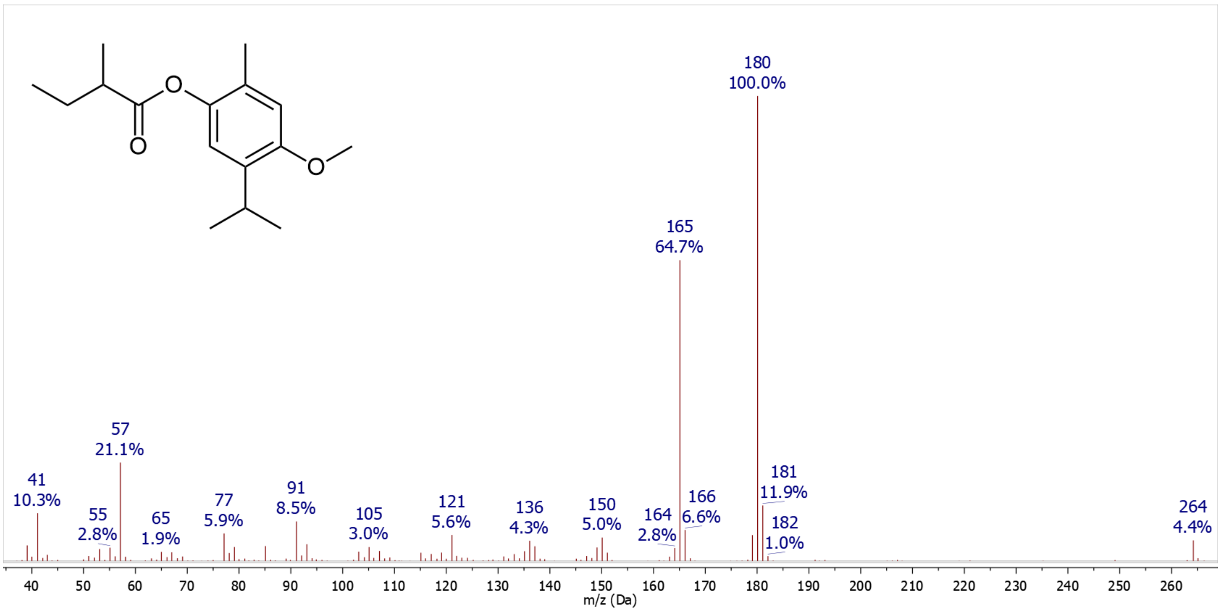

Figure S59. Mass spectrum (EI, 70 eV) of 6-(2-methylbutyryloxy)thymyl methyl ether (**15**)

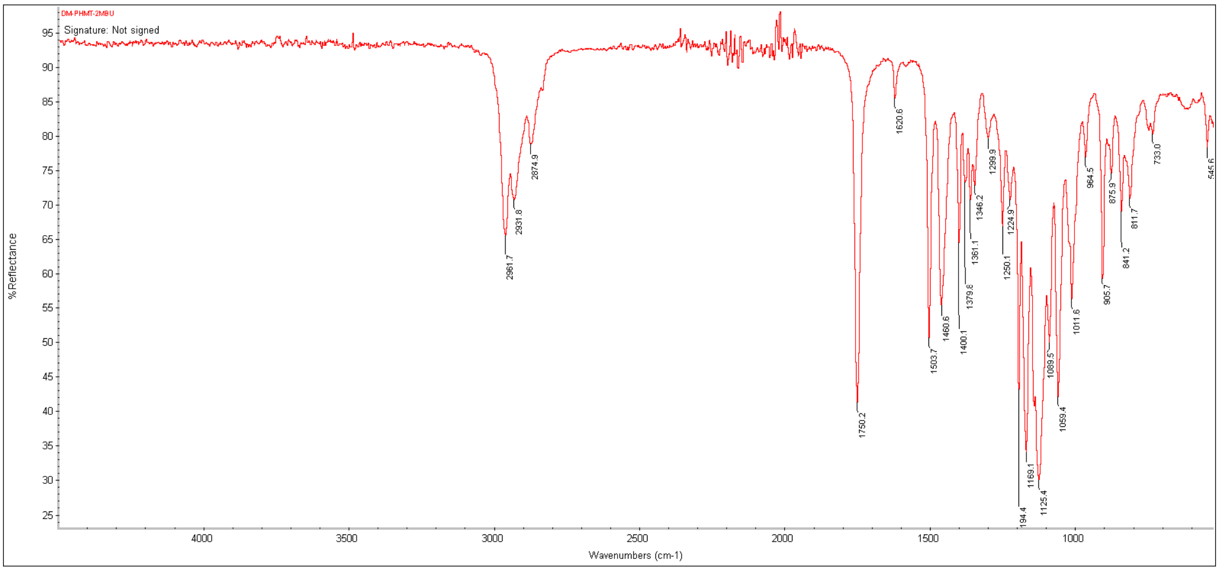

Figure S60. IR spectrum of 6-(2-methylbutyryloxy)thymyl methyl ether (**15**)

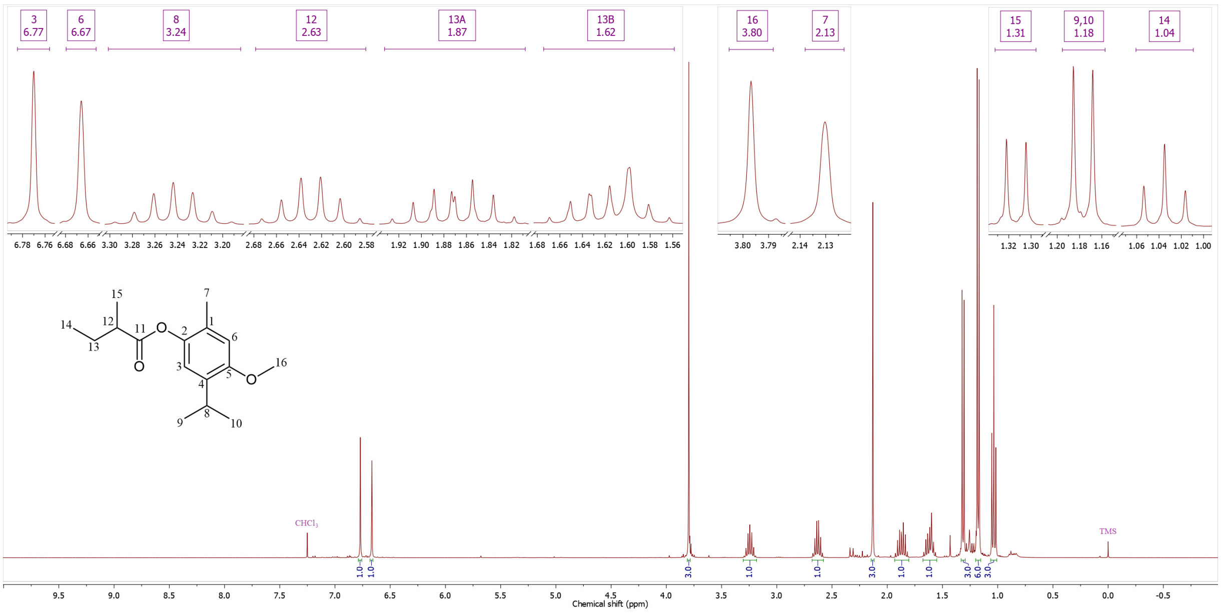

Figure S61. ^{1}H NMR (400 MHz, $CDCl_3$) spectrum of 6-(2-methylbutyryloxy)thymyl methyl ether (**15**) and the corresponding expansions with signal assignment

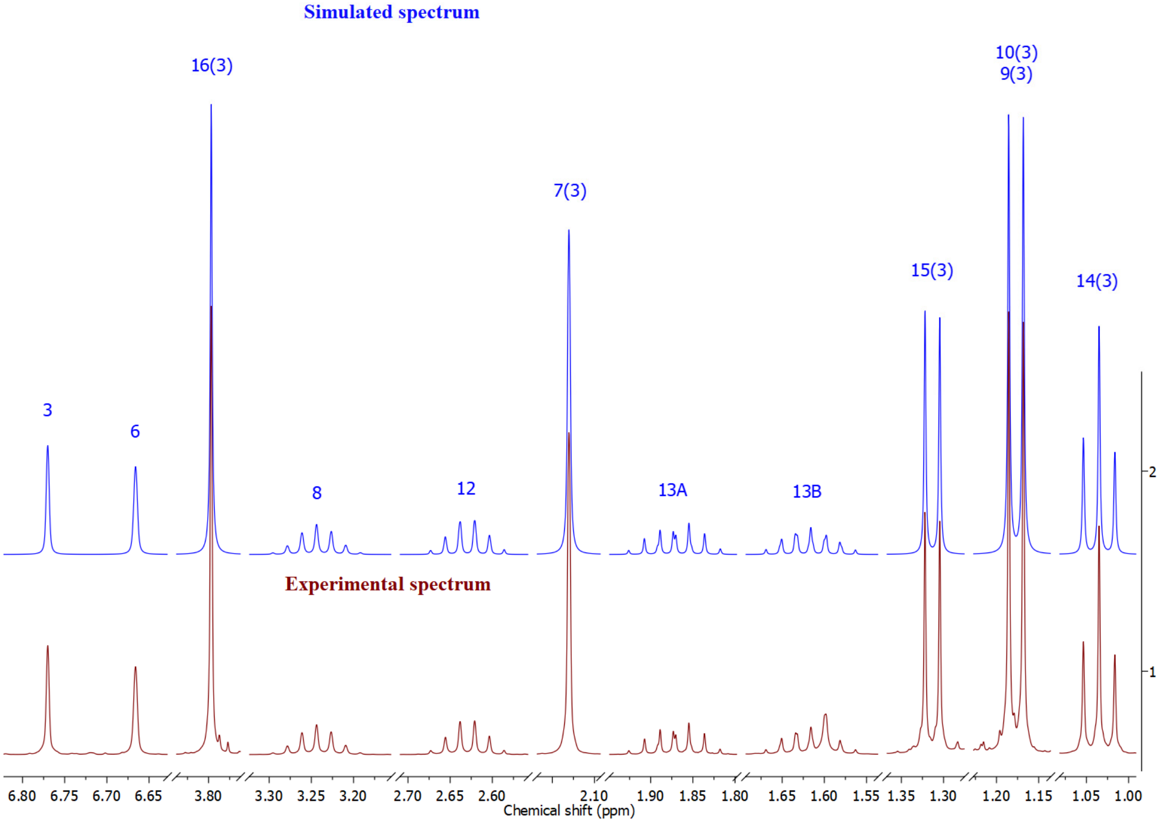

Figure S62. Simulated (manual iterative full spin, MestreNova 11.0.3) and experimental ^{1}H NMR spectrum of 6-(2-methylbutyryloxy)thymyl methyl ether (**15**)

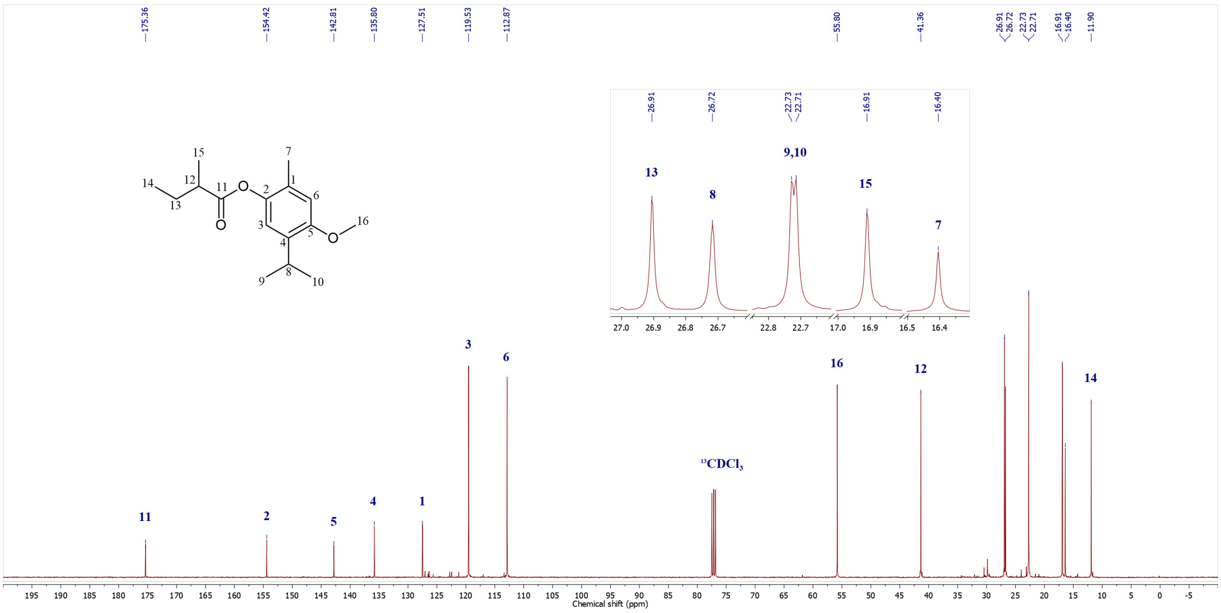

Figure S63. ^{13}C NMR (100.6 MHz, $CDCl_3$) spectrum of 6-(2-methylbutyryloxy)thymyl methyl ether (**15**)

Table S16. ^{1}H (400 MHz) and ^{13}C (100.6 MHz) NMR data of 6-(2-methylbutyryloxy)thymyl methyl ether (**15**) (chloroform-*d*), NMR parameters are derived from manual iterative full spin analysis, along with the observed gHMBC and NOESY correlations

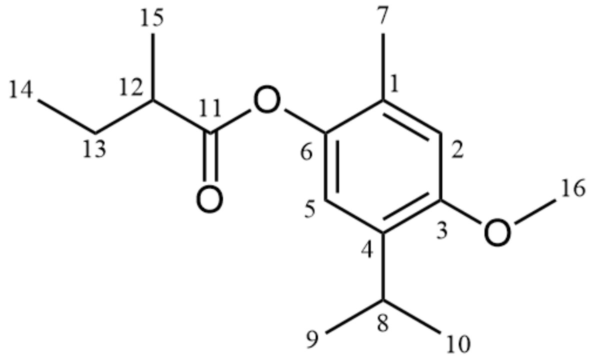

Position	δ_H (m, *J* (Hz), Integration)	δ_C	HMBC[a]	NOESY[b]
1	/	127.51	/	/
2	/	142.81	/	/
3	6.7701 (dqd, $^4J_{3,8}$ = -0.6, $^5J_{3,7}$ = 0.4, $^5J_{3,6}$ = 0.3, 1 H)	119.53	1,2,5,8	9,10
4	/	135.80	/	/
5	/	154.42	/	/
6	6.6660 (qdd, $^4J_{6,7}$ = -0.7, $^5J_{6,8}$ = 0.4, $^5J_{3,6}$ = 0.3, 1 H)	112.87	2,4,5,7	7,16
7	2.1303 (dd, $^4J_{6,7}$ = -0.7, $^5J_{3,7}$ = 0.4, 3 H)	16.40	1,2,6	6
8	3.2436 (septetdd, $^3J_{8,9/10}$ = 6.9, $^4J_{3,8}$ = -0.6, $^5J_{6,8}$ = 0.4, 1 H)	26.72	3,4,5,9,10	9,10
9 and 10	1.1769 (d, $^3J_{8,9/10}$ = 6.9, 6 H)	22.71/22.73	4,8,9,10	3,8,16
11	/	175.36	/	/
12	2.6293 (dqdq, $^3J_{12,13A}$ = 7.5, $^3J_{12,15}$ = 7.0, $^3J_{12,13B}$ = 6.5, $^4J_{12,14}$ = 0.3, 1 H)	41.36	11,13,14,15	13A,13B,15
13A	1.8704 (ddq, $^2J_{13A,13B}$ = -13.7, $^3J_{12,13A}$ = 7.5, $^3J_{13A,14}$ = 7.5, 1 H)	26.91	11,12,14,15	12,14,15

13B	1.6169 (dqd, $^2J_{13A,13B}$ = -13.7, $^3J_{13B,14}$ = 7.5, $^3J_{12,13B}$ = 6.5, 1 H)	26.91	11,12,14,15	12,14,15
14	1.0352 (ddd, $^3J_{13A,14}$ = 7.5, $^3J_{13B,14}$ = 7.5, $^4J_{12,14}$ = 0.3, 3 H)	11.90	12,13	13A,13B
15	1.3133 (d, $^3J_{12,15}$ = 7.0, 3 H)	16.91	11,12,13	14,15A,15B
16	3.7968 (s, 3 H)	55.80	5	6,9,10

[a] gHMBC correlations observed between the hydrogen in this row and the carbon in the listed position.
[b] Cross-peaks observed in the NOESY spectrum.

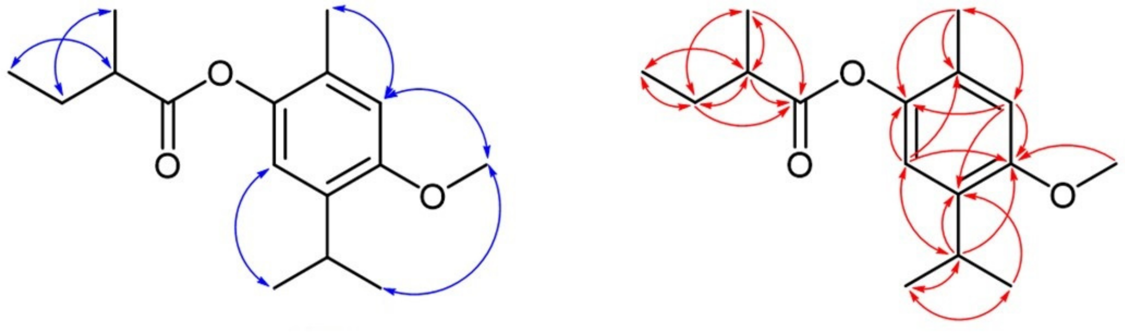

Figure S64. NOESY (blue arrows) and HMBC (red arrows) interactions of 6-(2-methylbutyryloxy)thymyl methyl ether (**15**)

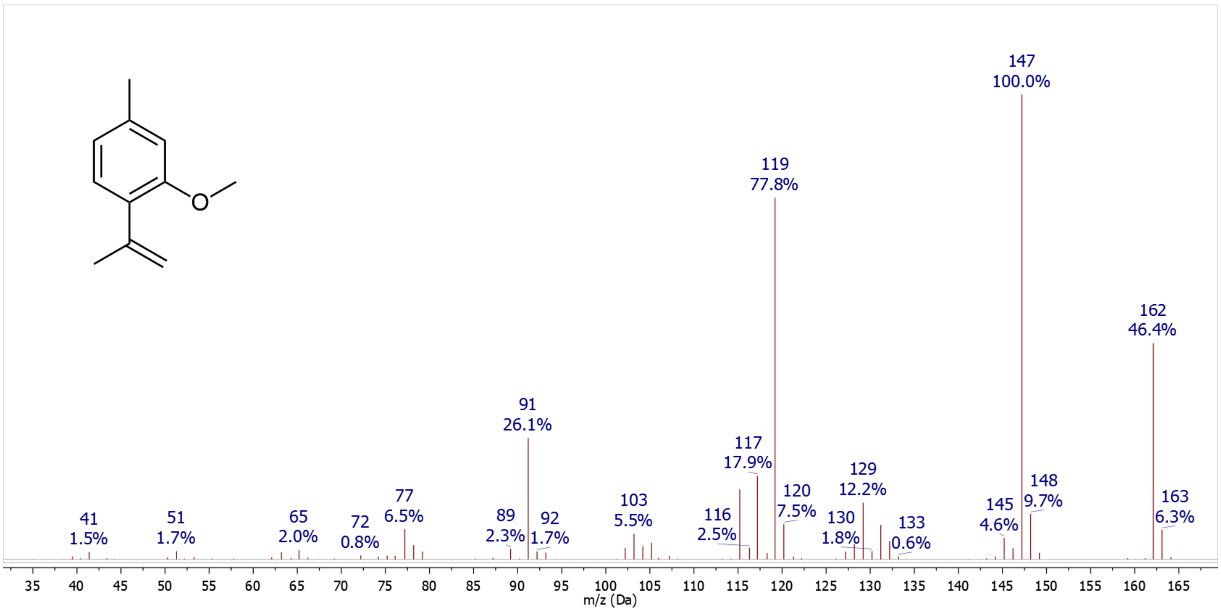

Figure S65. Mass spectrum (EI, 70 eV) of 8,9-dehydrothymyl methyl ether (**18**)

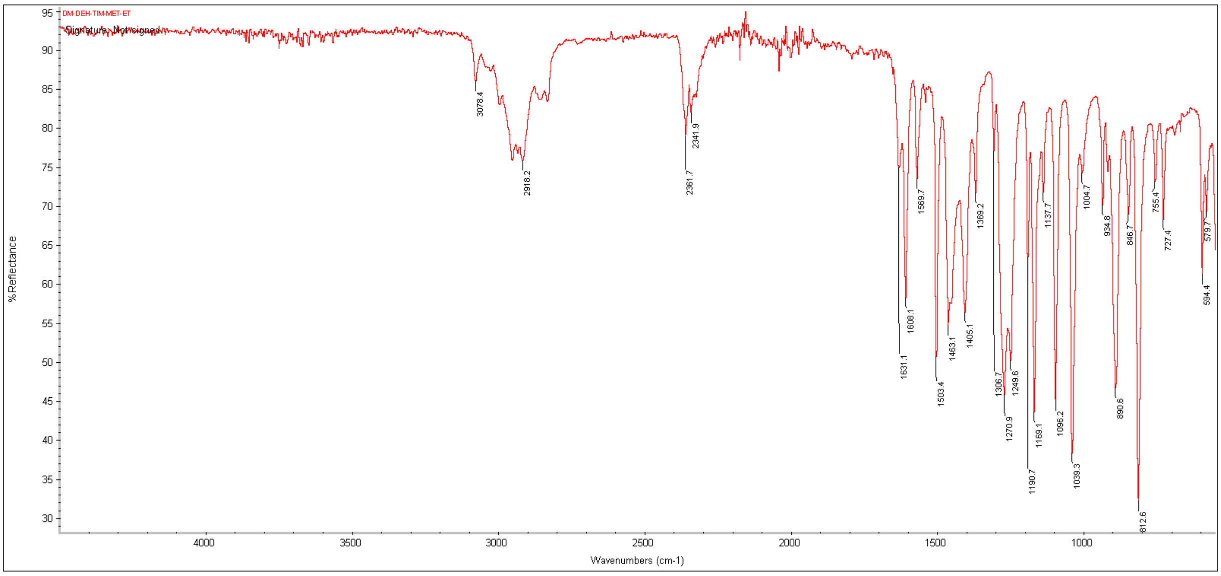

Figure S66. IR spectrum of 8,9-dehydrothymyl methyl ether (**18**)

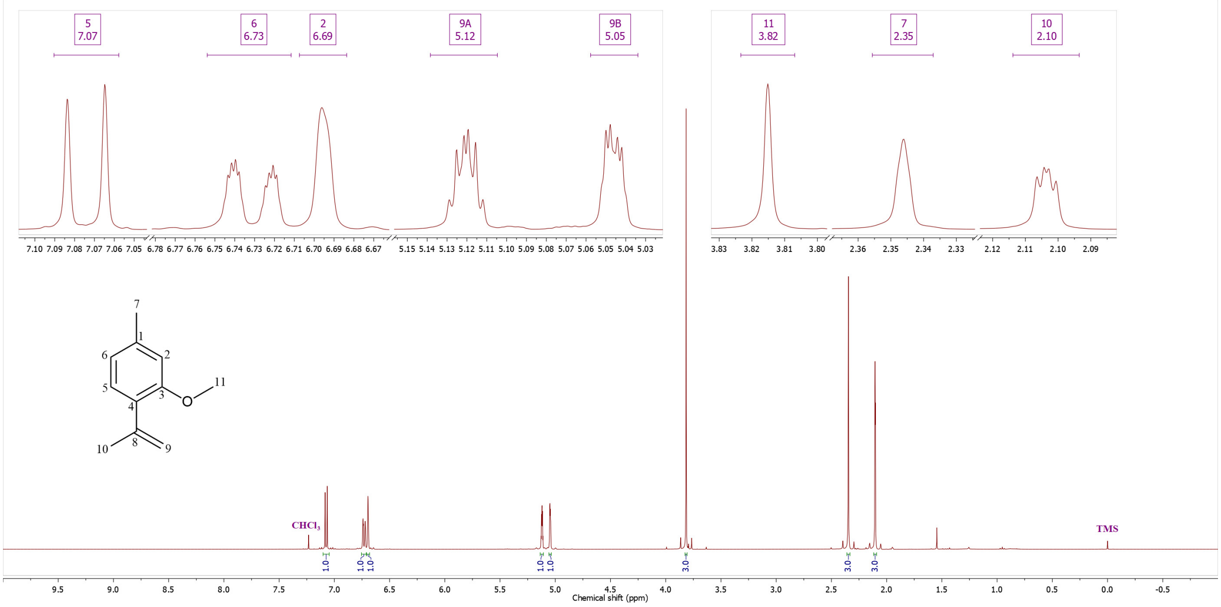

Figure S67. ¹H NMR (400 MHz, $CDCl_3$) spectrum of 8,9-dehydrothymyl methyl ether (**18**) and the corresponding expansions with signal assignment

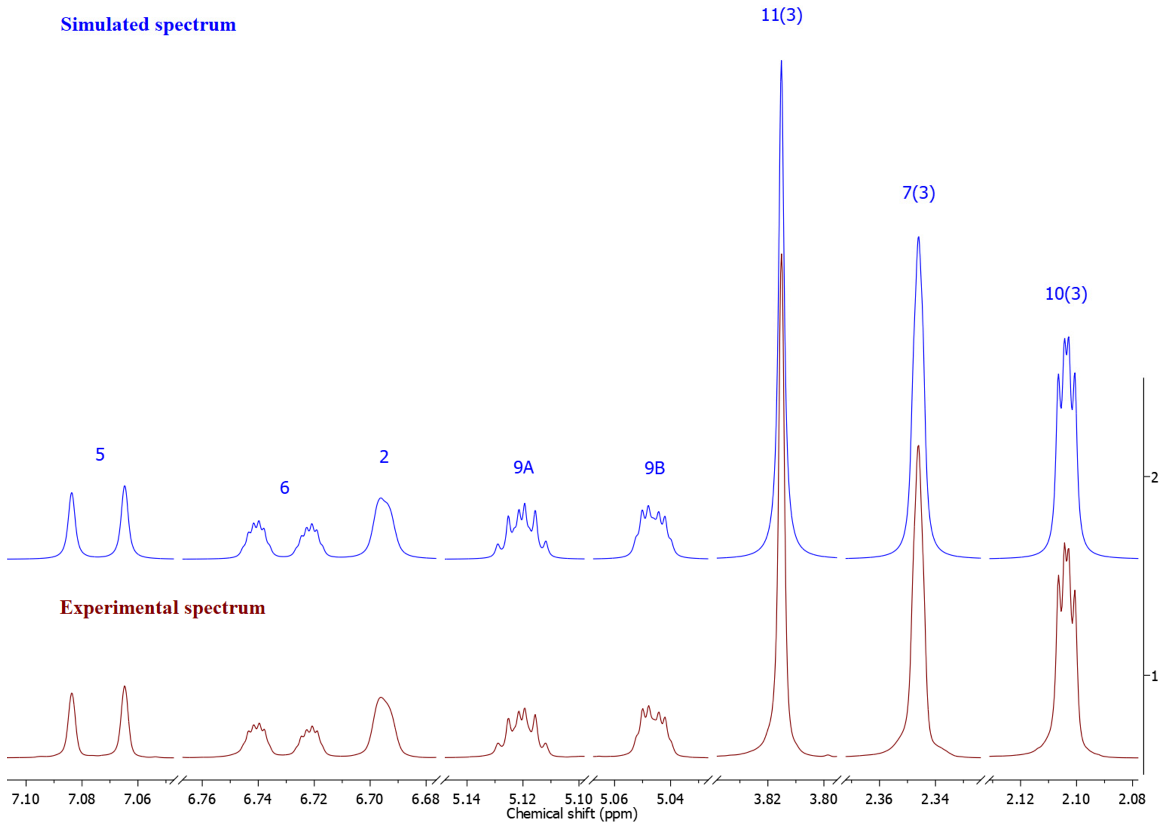

Figure S68. Simulated (manual iterative full spin, MestreNova 11.0.3) and experimental ^{1}H NMR spectrum of 8,9-dehydrothymyl methyl ether (**18**)

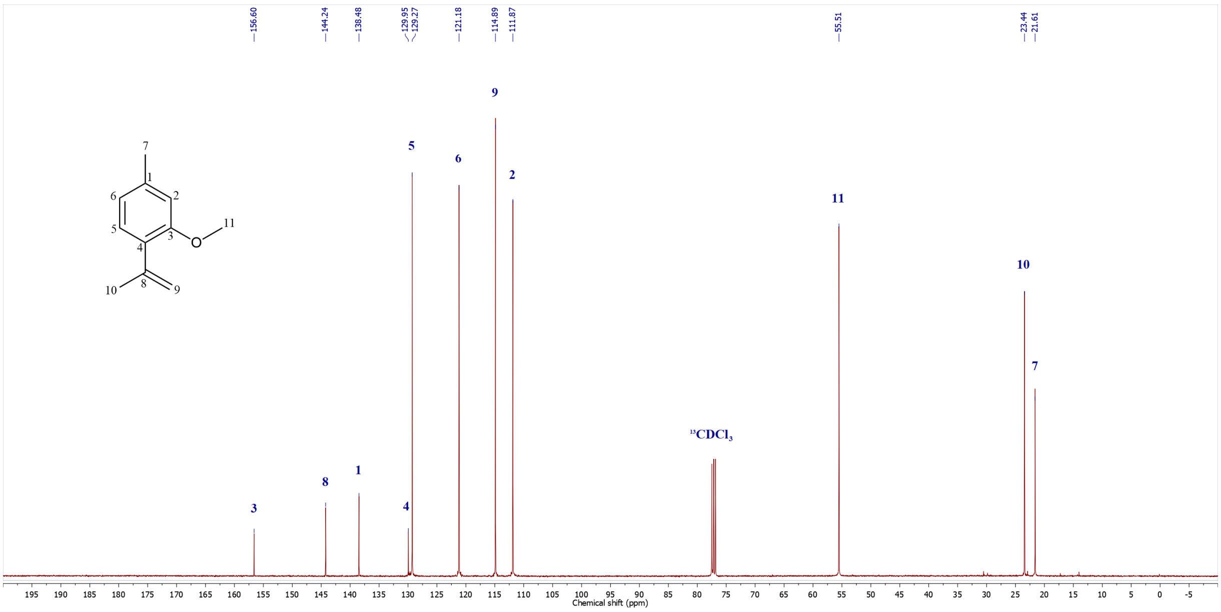

Figure S69. ^{13}C NMR (100.6 MHz, $CDCl_3$) spectrum of 8,9-dehydrothymyl methyl ether (**18**)

Table S17. ^{1}H (400 MHz) and ^{13}C (100.6 MHz) NMR data of 8,9-dehydrothymyl methyl ether (**18**) (chloroform-*d*), NMR parameters are derived from manual iterative full spin analysis, along with the observed gHMBC and NOESY correlations

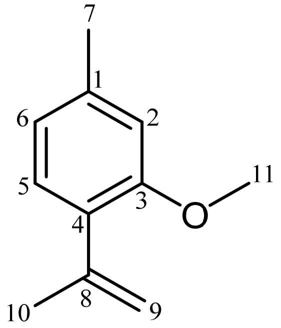

Position	δ_H (m, J (Hz), Integration)	δ_C	HMBC[a]	NOESY[b]
1	/	138.48	/	/
2	6.6951 (dq, $^4J_{2,6}$ = 1.5, $^4J_{2,7}$ = -0.6, 1 H)	111.87	3,4,6,7	7,11
3	/	156.60	/	/
4	/	129.95	/	/
5	7.0739 (dq, $^3J_{5,6}$ = 7.6, $^5J_{5,7}$ = 0.3, 1 H)	129.27	1.3,8	6,9B,10
6	6.7314 (ddq, $^3J_{5,6}$ = 7.6, $^4J_{2,6}$ = 1.5, $^4J_{6,7}$ = -0.8, 1 H)	121.18	2,4,7	5,7
7	2.3461 (ddd, $^4J_{6,7}$ = -0.8, $^4J_{2,7}$ = -0.6, $^5J_{5,7}$ = 0.3, 3 H)	21.61	1,2,6	2,6
8	/	144.24	/	/
9A	5.1203 (dq, $^2J_{9A,9B}$ = 2.4, $^3J_{9A,10}$ = -1.5, 1 H)	114.89	4,10	10
9B	5.0462 (dq, $^2J_{9A,9B}$ = 2.4, $^3J_{9B,10}$ = -0.9, 1 H)	114.89	4,8,10	5
10	2.1036 (dd, $^3J_{9A,10}$ = -1.5, $^3J_{9B,10}$ = -0.9, 3 H)	23.44	4,8,9	9A,11
11	3.8152 (s, 3 H)	55.51	3	2,10

[a] gHMBC correlations observed between the hydrogen in this row and the carbon in the listed position.
[b] Cross-peaks observed in the NOESY spectrum.

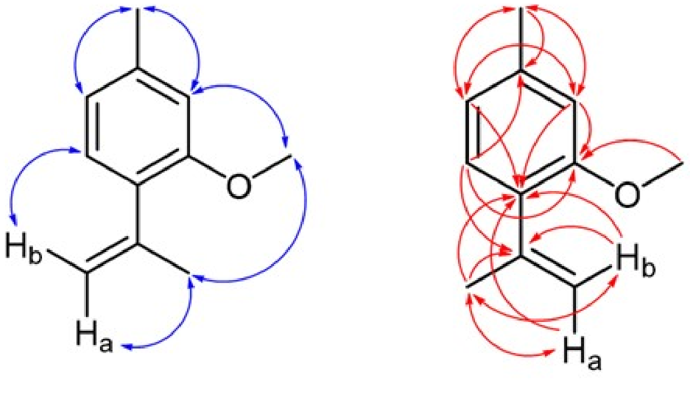

Figure S70. NOESY (blue arrows) and HMBC (red arrows) interactions of 8,9-dehydrothymyl methyl ether (**18**)

Table S18. ^{1}H (400 MHz) and ^{13}C (100.6 MHz) NMR data of 9-hydroxythymyl methyl ether (**19**) (chloroform-*d*), NMR parameters are derived from manual iterative full spin analysis, along with the observed gHMBC and NOESY correlations

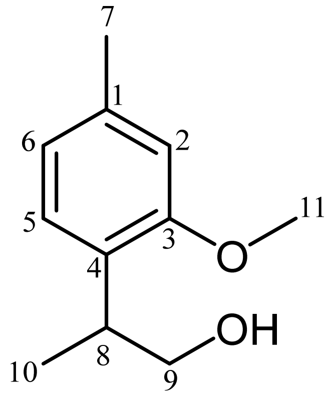

Position	δ_H (m, J (Hz), Integration)	δ_C	HMBC[a]	NOESY[b]
1	/	137.47	/	/
2	6.7008 (dqd, $^4J_{2,6}$ = 1.6, $^4J_{2,7}$ = -0.6, $^5J_{2,8}$ = 0.4, 1 H)	111.73	3,4,6,7	7,11
3	/	157.33	/	/
4	/	128.78	/	/
5	7.0788 (ddq, $^3J_{5,6}$ = 7.7, $^4J_{5,8}$ = -0.5, $^5J_{5,7}$ = 0.3, 1 H)	127.26	1.3,8	6,9,10
6	6.7706 (ddqd, $^3J_{5,6}$ = 7.7, $^4J_{2,6}$ = 1.6, $^4J_{6,7}$ = -0.7, $^5J_{6,8}$ = 0.4, 1 H)	121.49	2,4,7	5,7
7	2.3376 (ddd, $^4J_{6,7}$ = -0.7, $^4J_{2,7}$ = -0.6, $^5J_{5,7}$ = 0.3, 3 H)	21.56	1,2,6	2,6
8	3.3846 (qdddd, $^3J_{8,10}$ = 7.1, $^3J_{8,9A}$ = 7.1, $^3J_{8,9B}$ = 6.1, $^4J_{5,8}$ = -0.5, $^5J_{2,8}$ = 0.4, $^5J_{6,8}$ = 0.4, 1 H)	35.08	3,4,5,9,10	9,10
9A	3.7077 (ddd, $^2J_{9A,9B}$ = -10.5, $^3J_{8,9A}$ = 7.1, $^3J_{9A,OH}$ = 0.5, 1 H)	68.08	4,8,10	8,10,OH
9B	3.6713 (ddd, $^2J_{9A,9B}$ = -10.5, $^3J_{8,9B}$ = 6.1, $^3J_{9B,OH}$ = 0.5, 1 H)	68.08	4,8,10	8,10,OH
10	1.2410 (d, $^3J_{8,10}$ = 7.1, 3 H)	16.77	4,8,9	5,8,9A,9B
11	3.8137 (s, 3 H)	55.49	3	2
OH	1.5521 (dd, $^3J_{9A,OH}$ = 0.5, $^3J_{9B,OH}$ = 0.5, 1 H)	/	/	9A,9B

[a] gHMBC correlations observed between the hydrogen in this row and the carbon in the listed position.
[b] Cross-peaks observed in the NOESY spectrum.

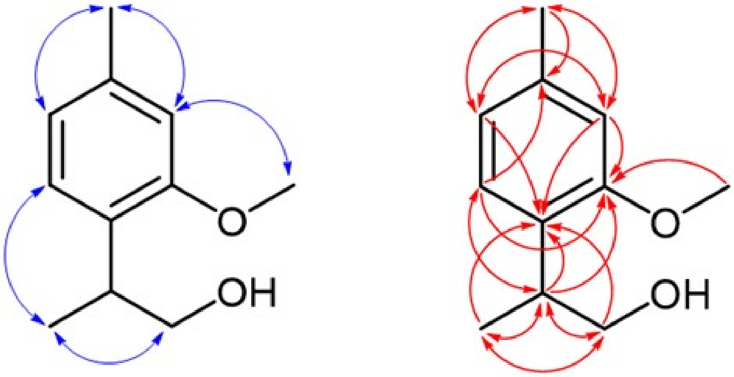

Figure S71. NOESY (blue arrows) and HMBC (red arrows) interactions of 9-hydroxythymyl methyl ether (**19**)

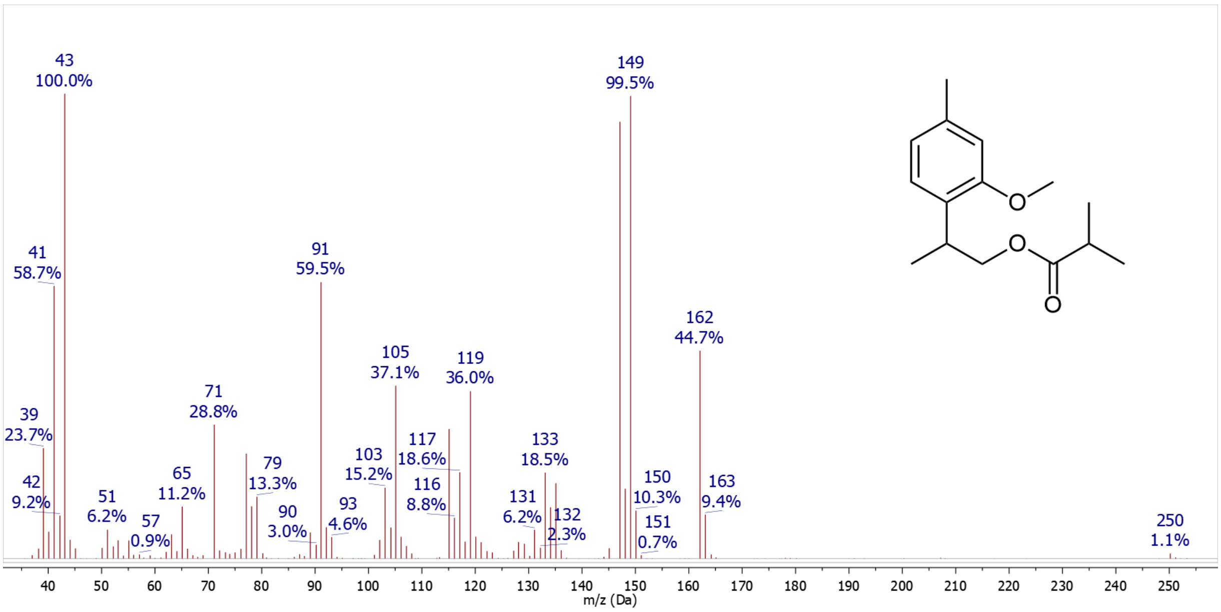

Figure S72. Mass spectrum (EI, 70 eV) of 9-isobutyryloxyoxythymyl methyl ether (**20**)

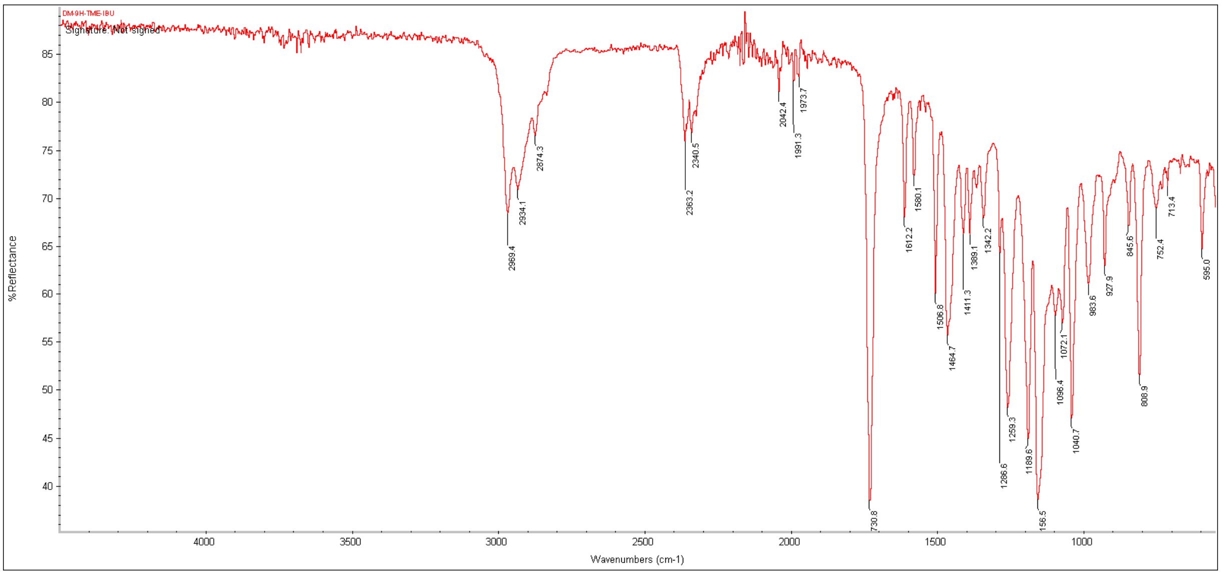

Figure S73. IR spectrum of 9-isobutyryloxyoxythymyl methyl ether (**20**)

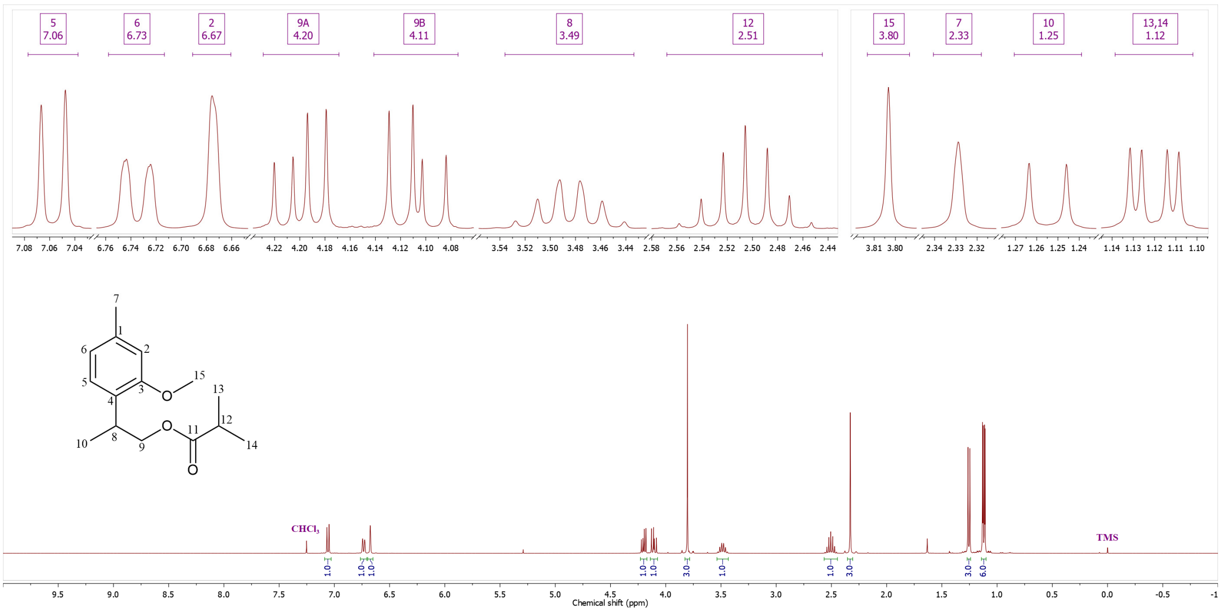

Figure S74. ^{1}H NMR (400 MHz, $CDCl_3$) spectrum of 9-isobutyryloxyoxythymyl methyl ether (**20**) and the corresponding expansions with signal assignment

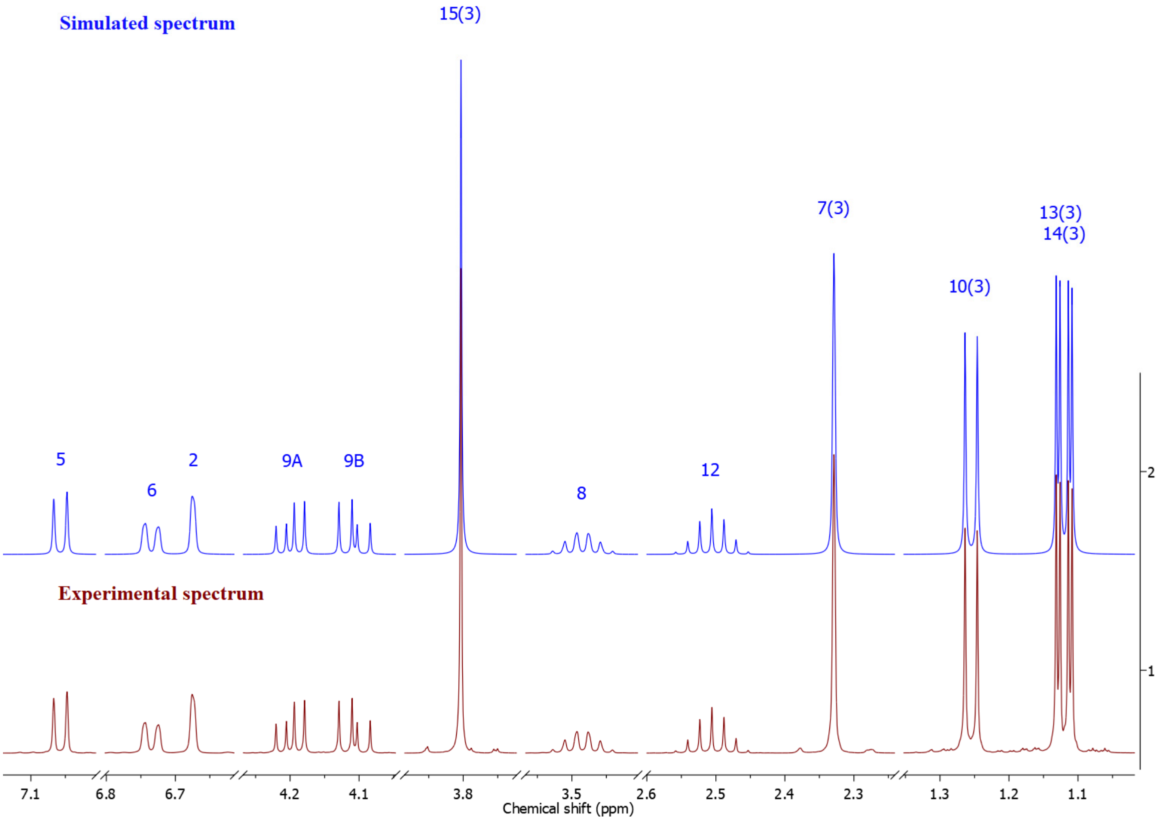

Figure S75. Simulated (manual iterative full spin, MestreNova 11.0.3) and experimental ^{1}H NMR spectrum of 9-isobutyryloxyoxythymyl methyl ether (**20**)

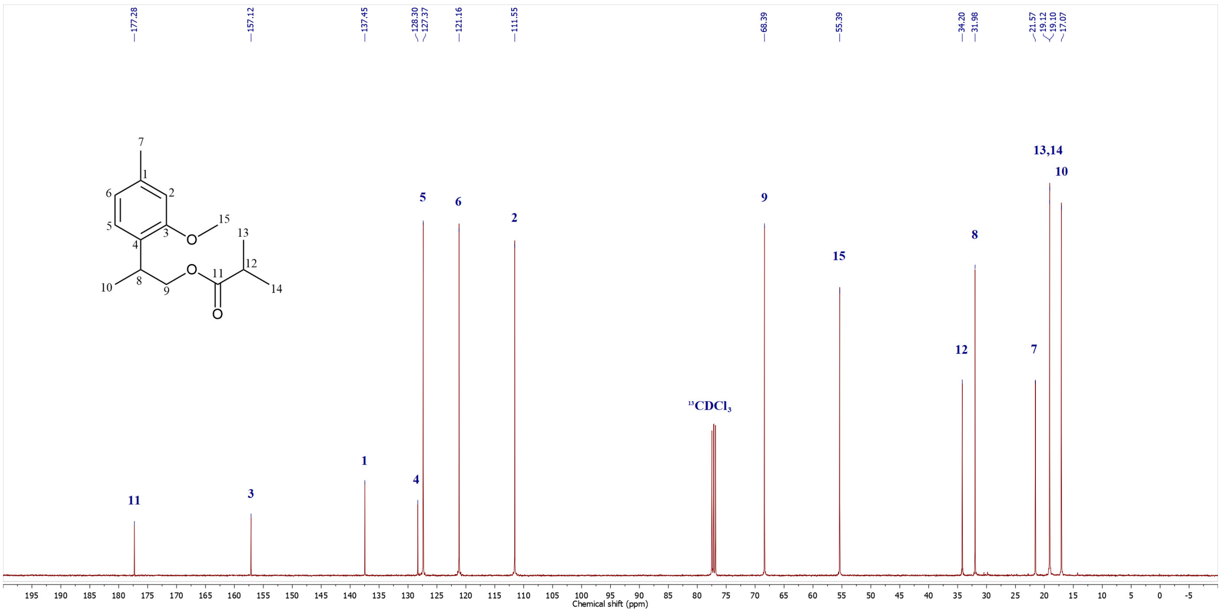

Figure S76. ^{13}C NMR (100.6 MHz, $CDCl_3$) spectrum of 9-isobutyryloxyoxythymyl methyl ether (**20**)

Table S19. ^{1}H (400 MHz) and ^{13}C (100.6 MHz) NMR data of 9-isobutyryloxyoxythymyl methyl ether (**20**) (chloroform-*d*), NMR parameters are derived from manual iterative full spin analysis, along with the observed gHMBC and NOESY correlations

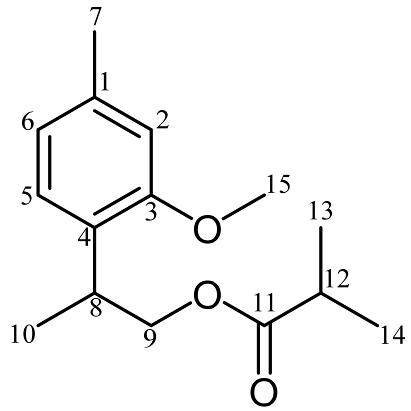

Position	δ_H (m, J (Hz), Integration)	δ_C	HMBC[a]	NOESY[b]
1	/	137.45	/	/
2	6.6744 (dqd, $^4J_{2,6}$ = 1.6, $^4J_{2,7}$ = -0.6, $^5J_{2,8}$ = 0.4, 1 H)	111.55	3,4,6,7	7,15
3	/	157.12	/	/
4	/	128.30	/	/
5	7.0569 (ddq, $^3J_{5,6}$ = 7.7, $^4J_{5,8}$ = -0.5, $^5J_{5,7}$ = 0.3, 1 H)	127.35	1.3,8	6,9,10
6	6.7352 (ddqd, $^3J_{5,6}$ = 7.7, $^4J_{2,6}$ = 1.6, $^4J_{6,7}$ = -0.7, $^5J_{6,8}$ = 0.3, 1 H)	121.16	2,4,7	5,7
7	2.3289 (ddd, $^4J_{6,7}$ = -0.7, $^4J_{2,7}$ = -0.6, $^5J_{5,7}$ = 0.3, 3 H)	21.57	1,2,6	2,6
8	3.4845 (dqdddd, $^3J_{8,9B}$ = 7.7, $^3J_{8,10}$ = 7.0, $^3J_{8,9A}$ = 5.9, $^4J_{5,8}$ = -0.5, $^5J_{2,8}$ = 0.4, $^5J_{6,8}$ = 0.3, 1 H)	31.98	3,4,5,9,10	9,10
9A	4.1977 (dd, $^2J_{9A,9B}$ = -10.6, $^3J_{8,9A}$ = 5.9, 1 H)	68.39	4,8,10,11	5,8,10,15
9B	4.1083 (dd, $^2J_{9A,9B}$ = -10.6, $^3J_{8,9B}$ = 7.7, 1 H)	68.39	4,8,10,11	5,8,10,15
10	1.2548 (d, $^3J_{8,10}$ = 7.0, 3 H)	17.07	4,8,9	5,8,9A,9B,15
11	/	177.28	/	/
12	2.5054 (sept, $^3J_{12,13/14}$ = 7.0, 1 H)	34.20	11,13,14	13,14

13 and 14	1.1228/1.1174 (d, $^{3}J_{12,13/14}$ = 7.0, 3 H)	19.12/19.10	11,12,14	12
15	3.8033 (s, 3 H)	55.39	3	2,9A,9B,10

[a] gHMBC correlations observed between the hydrogen in this row and the carbon in the listed position.
[b] Cross-peaks observed in the NOESY spectrum.

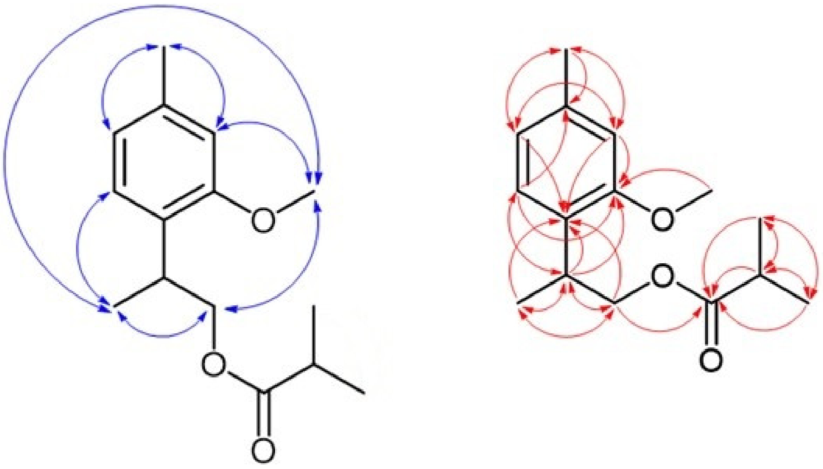

Figure S77. NOESY (blue arrows) and HMBC (red arrows) interactions 9-isobutyryloxyoxythymyl methyl ether (**20**)

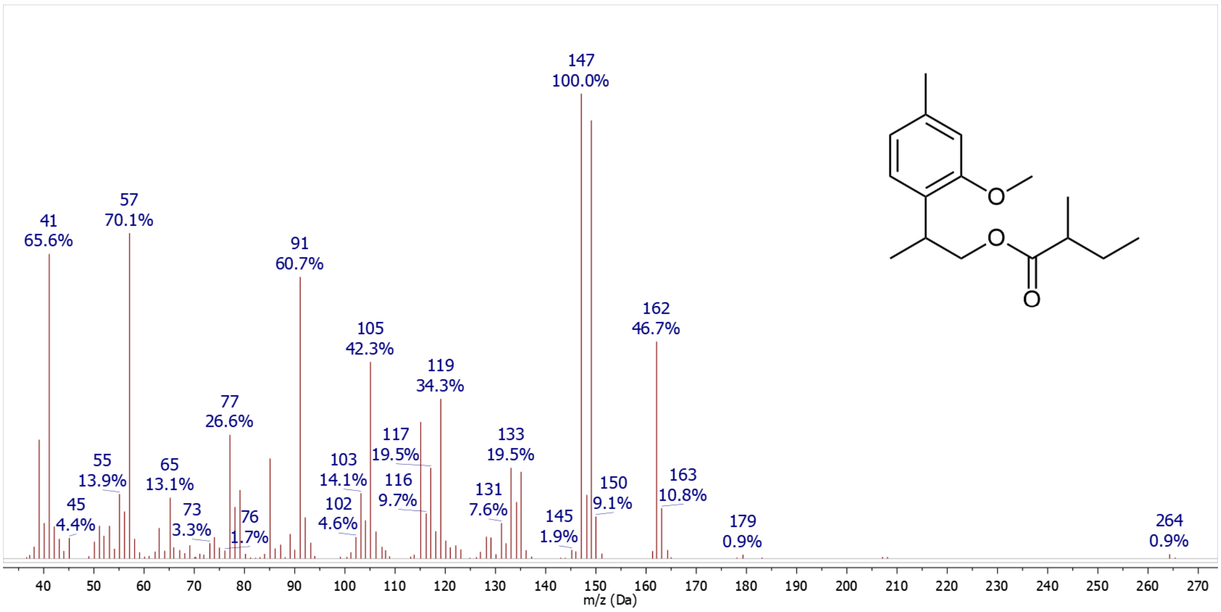

Figure S78. Mass spectrum (EI, 70 eV) of 9-(2-methylbutyryloxy)thymyl methyl ether (**21**)

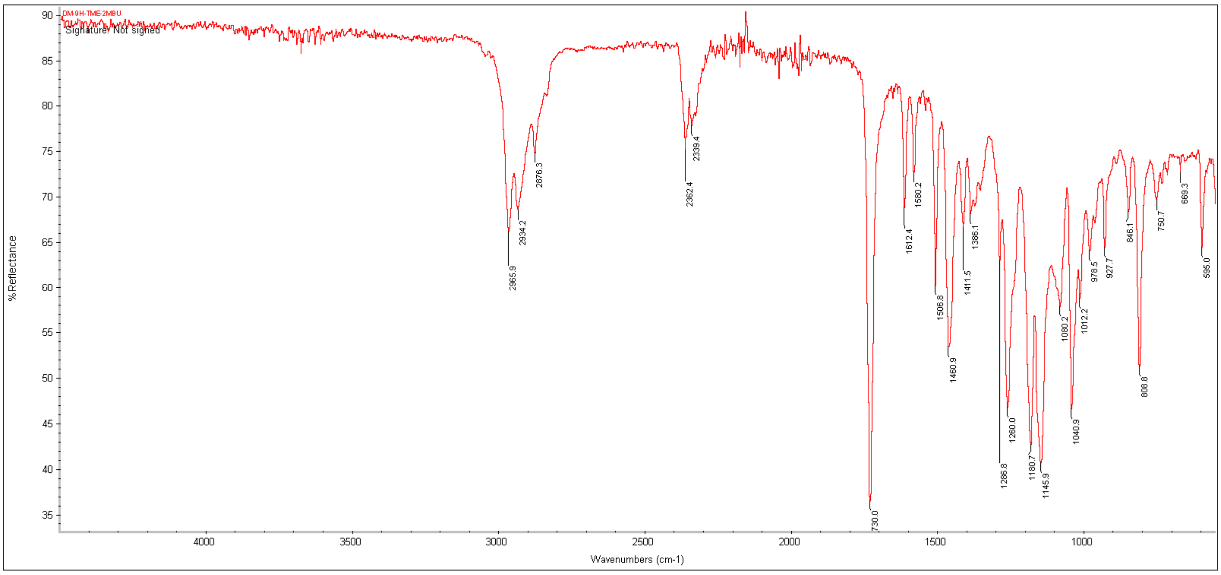

Figure S79. IR spectrum of 9-(2-methylbutyryloxy)thymyl methyl ether (**21**)

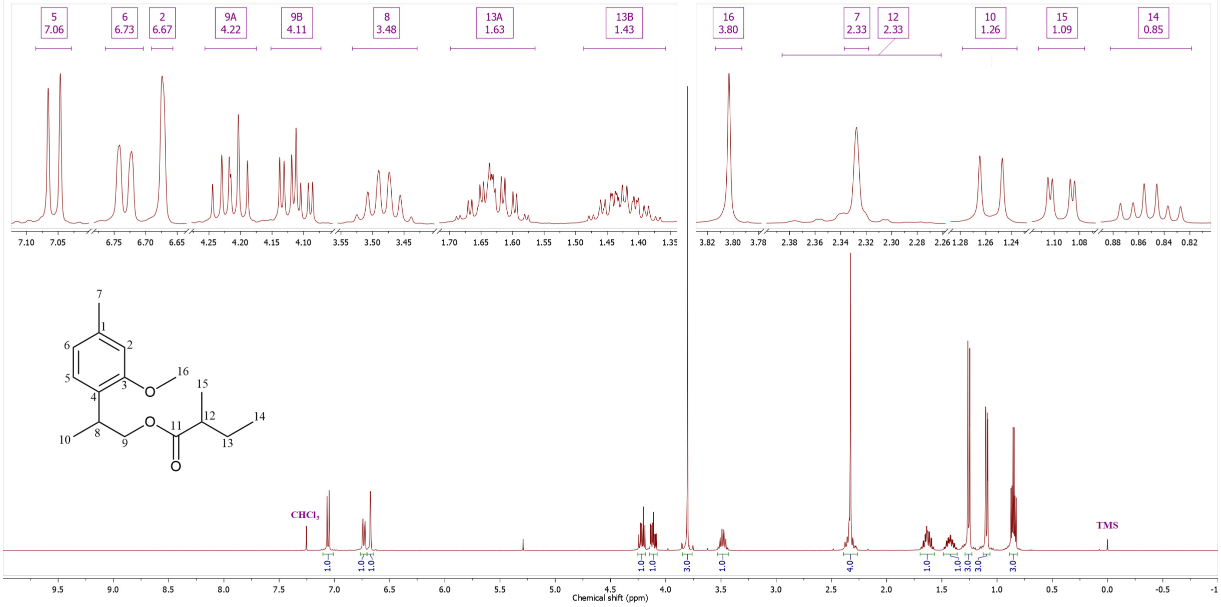

Figure S80. ^{1}H NMR (400 MHz, $CDCl_3$) spectrum of 9-(2-methylbutyryloxy)thymyl methyl ether (**21**) and the corresponding expansions with signal assignment

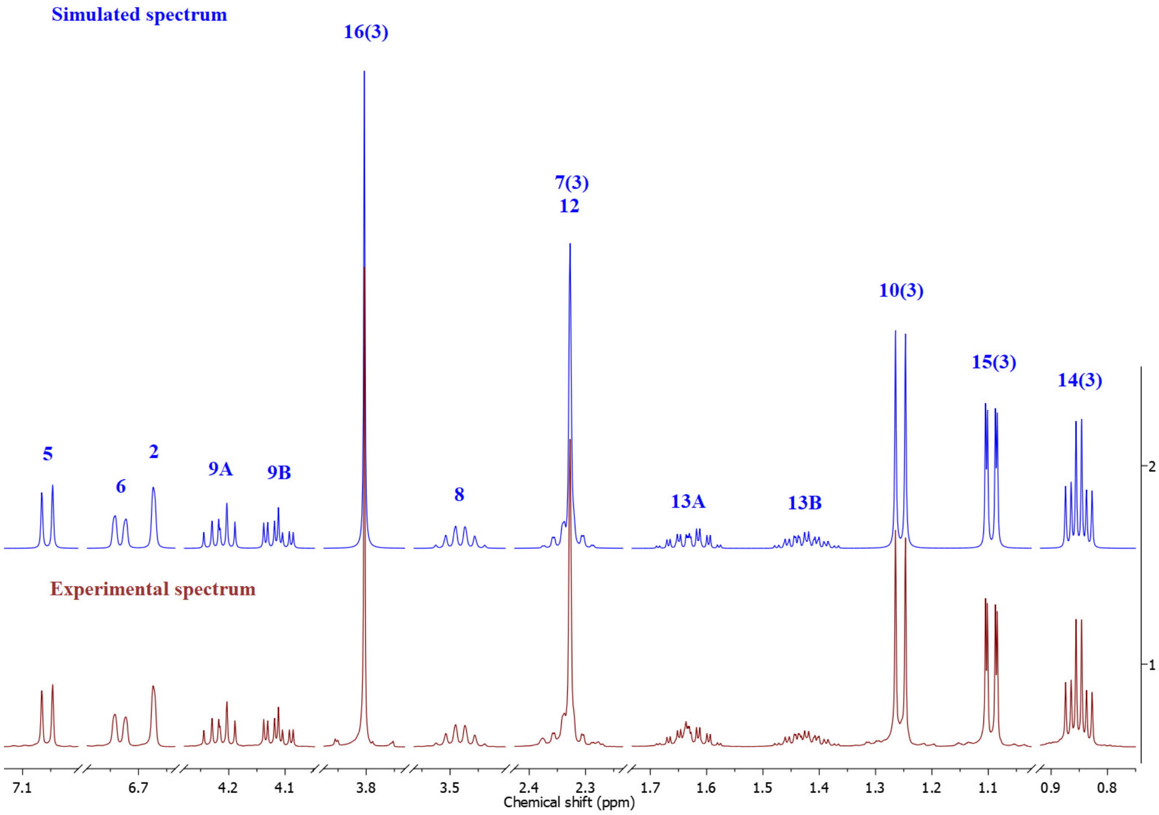

Figure S81. Simulated (manual iterative full spin, MestreNova 11.0.3) and experimental ^{1}H NMR spectrum of 9-(2-methylbutyryloxy)thymyl methyl ether (**21**)

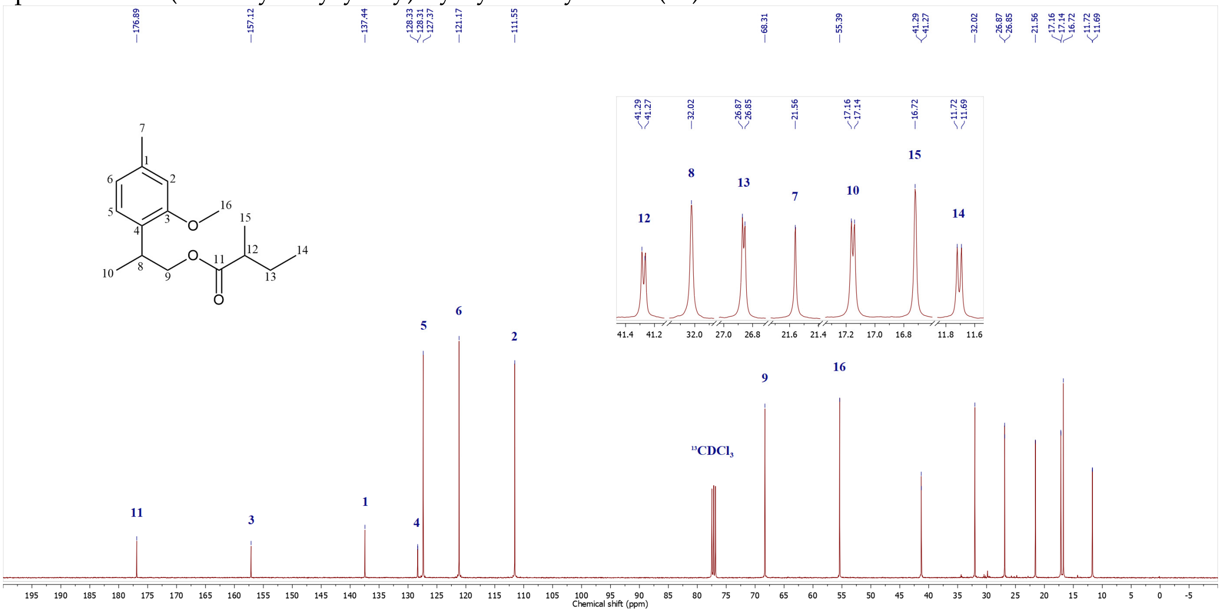

Figure S82. ^{13}C NMR (100.6 MHz, $CDCl_3$) spectrum of 9-(2-methylbutyryloxy)thymyl methyl ether (**21**)

Table S20. ^{1}H (400 MHz) and ^{13}C (100.6 MHz) NMR data of 9-(2-methylbutyryloxy)thymyl methyl ether (**21**) (chloroform-*d*), NMR parameters are derived from manual iterative full spin analysis, along with the observed gHMBC and NOESY correlations

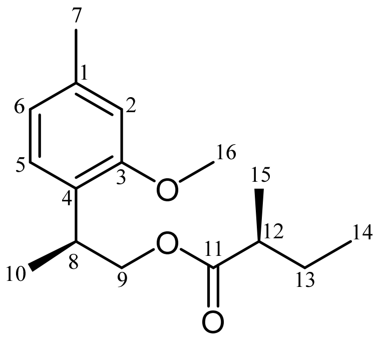

(±)

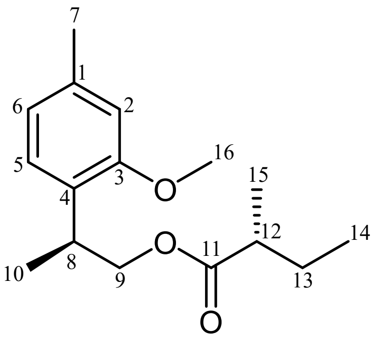

(±)

Position	δ_H (m, *J* (Hz), Integration)	δ_C	HMBC[a]	NOESY[b]
1	/	137.44	/	/
2	6.6729 (dqd, $^4J_{2,6}$ = 1.6, $^4J_{2,7}$ = -0.6, $^5J_{2,8}$ = 0.4, 1 H)	111.55	3,4,6,7	7,16
3	/	157.12	/	/
4	/	128.33/128.31	/	/
5	7.0559 (ddq, $^3J_{5,6}$ = 7.7, $^4J_{5,8}$ = -0.5, $^5J_{5,7}$ = 0.3, 1 H)	127.37	1.3,8	6,8,9,10
6	6.7331 (ddqd, $^3J_{5,6}$ = 7.7, $^4J_{2,6}$ = 1.6, $^4J_{6,7}$ = -0.7, $^5J_{6,8}$ = 0.3, 1 H)	121.17	2,4,7	5,7
7	2.3276 (ddd, $^4J_{6,7}$ = -0.7, $^4J_{2,7}$ = -0.6, $^5J_{5,7}$ = 0.3, 3 H)	21.56	1,2,6	2,6
8	3.4820 (dqdddd, $^3J_{8,9B}$ = 7.7, $^3J_{8,10}$ = 7.0, $^3J_{8,9A}$ = 6.0, $^4J_{5,8}$ = -0.5, $^5J_{2,8}$ = 0.4, $^5J_{6,8}$ = 0.3, 1 H)	32.02	3,4,5,9,10	5,9,10
9A	4.2219/4.2078 (dd, $^2J_{9A,9B}$ = -10.6, $^3J_{8,9A}$ = 6.0, 1 H)	68.31	4,8,10,11	5,8,10
9B	4.1167/4.1099 (dd, $^2J_{9A,9B}$ = -10.6, $^3J_{8,9B}$ = 7.7, 1 H)	68.31	4,8,10,11	5,8,10
10	1.2559 (d, $^3J_{8,10}$ = 7.0, 3 H)	17.16/17.14	4,8,9	5,8,9A,9B
11	/	176.89	/	/
12	2.3326/2.3288 (dqdq, $^3J_{12,13A}$ = 7.5, $^3J_{12,15}$ = 7.0, $^3J_{12,13B}$ = 6.5, $^4J_{12,14}$ = 0.3, 1 H)	41.29/41.27	11,13,14,15	13A,13B,15
13A	1.6333/1.6276 (ddq, $^2J_{13A,13B}$ = -13.7, $^3J_{12,13A}$ = 7.5, $^3J_{13A,14}$ = 7.5, 1 H)	26.87/26.85	11,12,14,15	12,14,15
13B	1.4272/1.4199 (dqd, $^2J_{13A,13B}$ = -13.7, $^3J_{13B,14}$ = 7.5, $^3J_{12,13B}$ = 6.5, 1 H)	26.87/26.85	11,12,14,15	12,14,15
14	0.8560/0.8460 (ddd, $^3J_{13A,14}$ = 7.5, $^3J_{13B,14}$ = 7.5, $^4J_{12,14}$ = 0.3, 3 H)	11.72/11.69	12,13	13A,13B
15	1.0962/1.0927 (d, $^3J_{12,15}$ = 7.0, 3 H)	16.72	11,12,13	12,13A,13B
16	3.8032 (s, 3 H)	55.39	3	2

[a] gHMBC correlations observed between the hydrogen in this row and the carbon in the listed position.
[b] Cross-peaks observed in the NOESY spectrum.

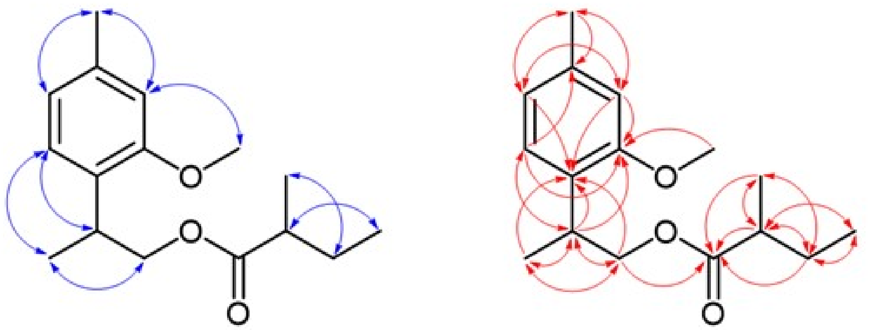

Figure S83. NOESY (blue arrows) and HMBC (red arrows) interactions 9-(2-methylbutyryloxy)thymyl methyl ether (**21**)

Table S21. ^{1}H (400 MHz) and ^{13}C (100.6 MHz) NMR data of 3-methoxycumic acid (chloroform-*d*), NMR parameters are derived from manual iterative full spin analysis, along with the observed gHMBC and NOESY correlations

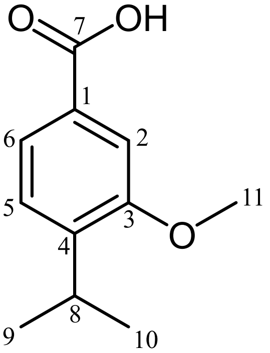

Position	δ_H (m, *J* (Hz), Integration)	δ_C	HMBC[a]	NOESY[b]
1	/	127.65	/	/
2	7.5560 (ddd, $^{4}J_{2,6}$ = 1.6, $^{5}J_{2,8}$ = 0.5, $^{5}J_{2,5}$ = 0.3, 1 H)	111.43	3,4,6,7	11
3	/	156.84	/	/
4	/	143.89	/	/
5	7.3036 (ddd, $^{3}J_{5,6}$ = 7.9, $^{4}J_{5,8}$ = -0.5, $^{5}J_{2,5}$ = 0.3, 1 H)	126.18	1,3,8	6,8,9,10
6	7.7109 (ddd, $^{3}J_{5,6}$ = 7.9, $^{4}J_{2,6}$ = 1.6, $^{5}J_{6,8}$ = 0.5, 1 H)	123.13	2,4,7	5
7	/	171.73	/	/
8	3.3770 (septddd, $^{3}J_{8,9/10}$ = 6.9, $^{5}J_{2,8}$ = 0.5, $^{4}J_{5,8}$ = -0.5, $^{5}J_{6,8}$ = 0.5, 1 H)	27.19	3,4,5,9,10	5,9,10
9 and 10	1.2315 (d, $^{3}J_{8,9/10}$ = 6.9, 6 H)	22.51	4,8,9,10	5,8,11
11	3.9016 (s, 3 H)	55.66	3	2,9,10
COOH	11.5910 (s, 1 H)	/	/	/

[a] gHMBC correlations observed between the hydrogen in this row and the carbon in the listed position.
[b] Cross-peaks observed in the NOESY spectrum.

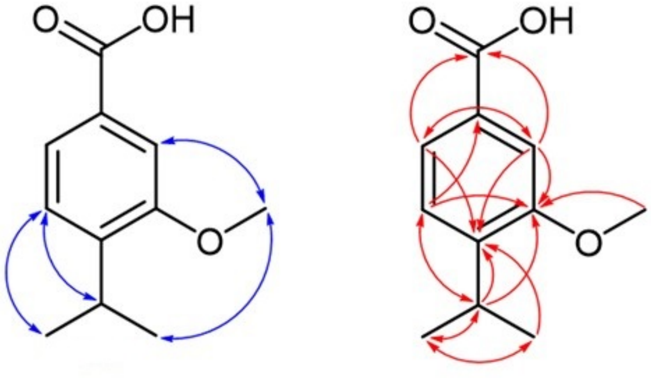

Figure S84. NOESY (blue arrows) and HMBC (red arrows) interactions 3-methoxycumic acid

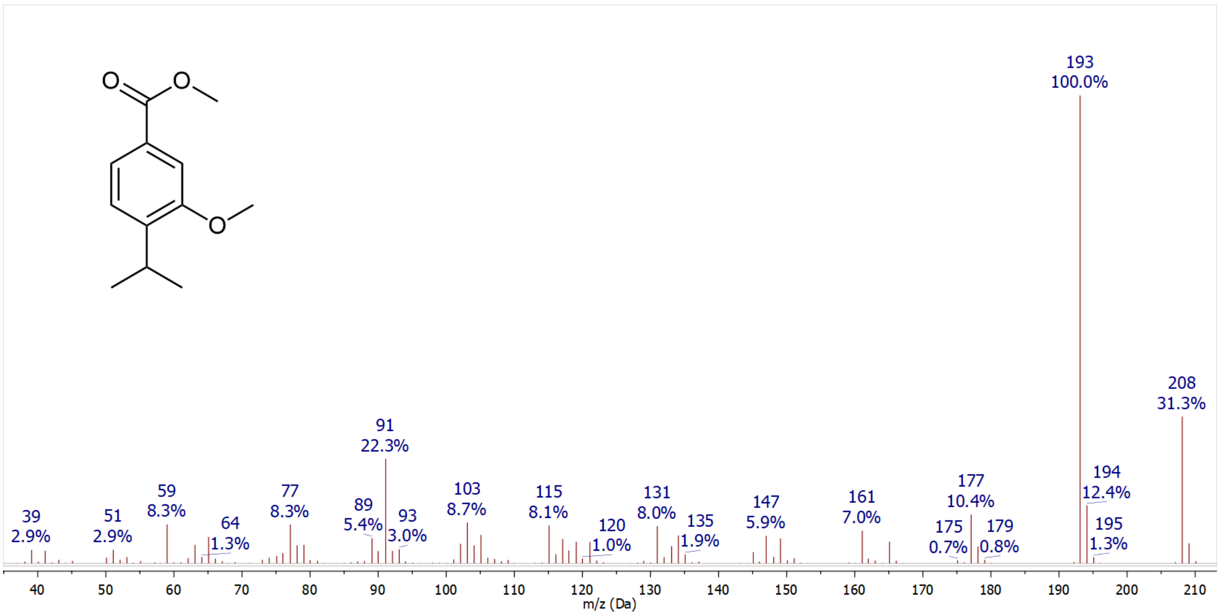

Figure S85. Mass spectrum (EI, 70 eV) of methyl 3-methoxycuminate (**23**)

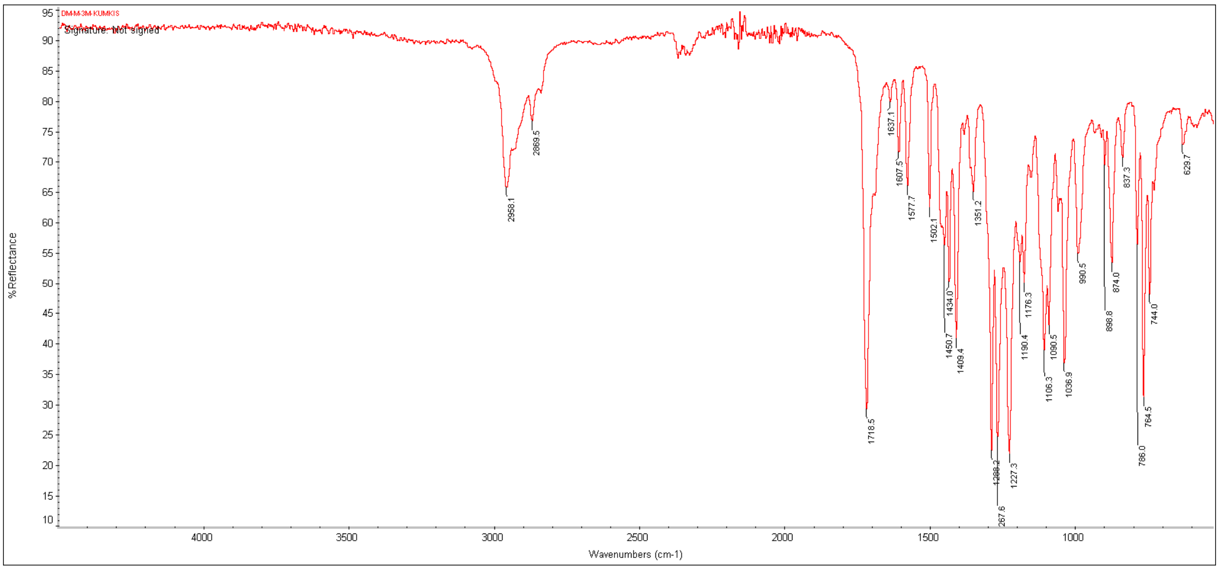

Figure S86. IR spectrum of methyl 3-methoxycuminate (**23**)

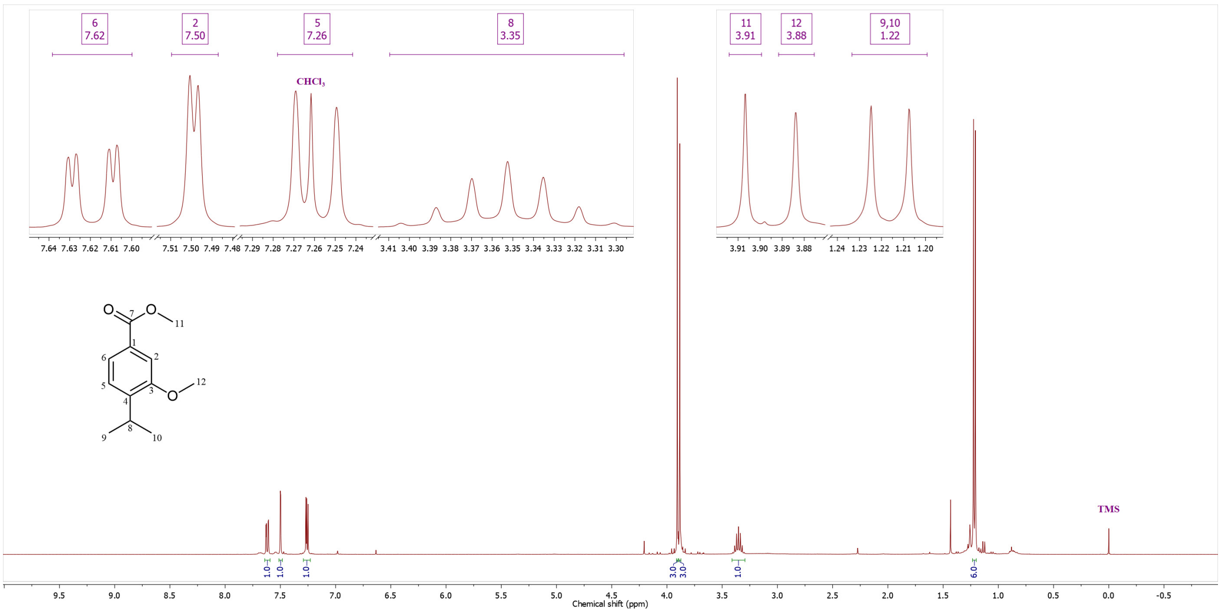

Figure S87. ^{1}H NMR (400 MHz, $CDCl_3$) spectrum of methyl 3-methoxycuminate (**23**) and the corresponding expansions with signal assignment

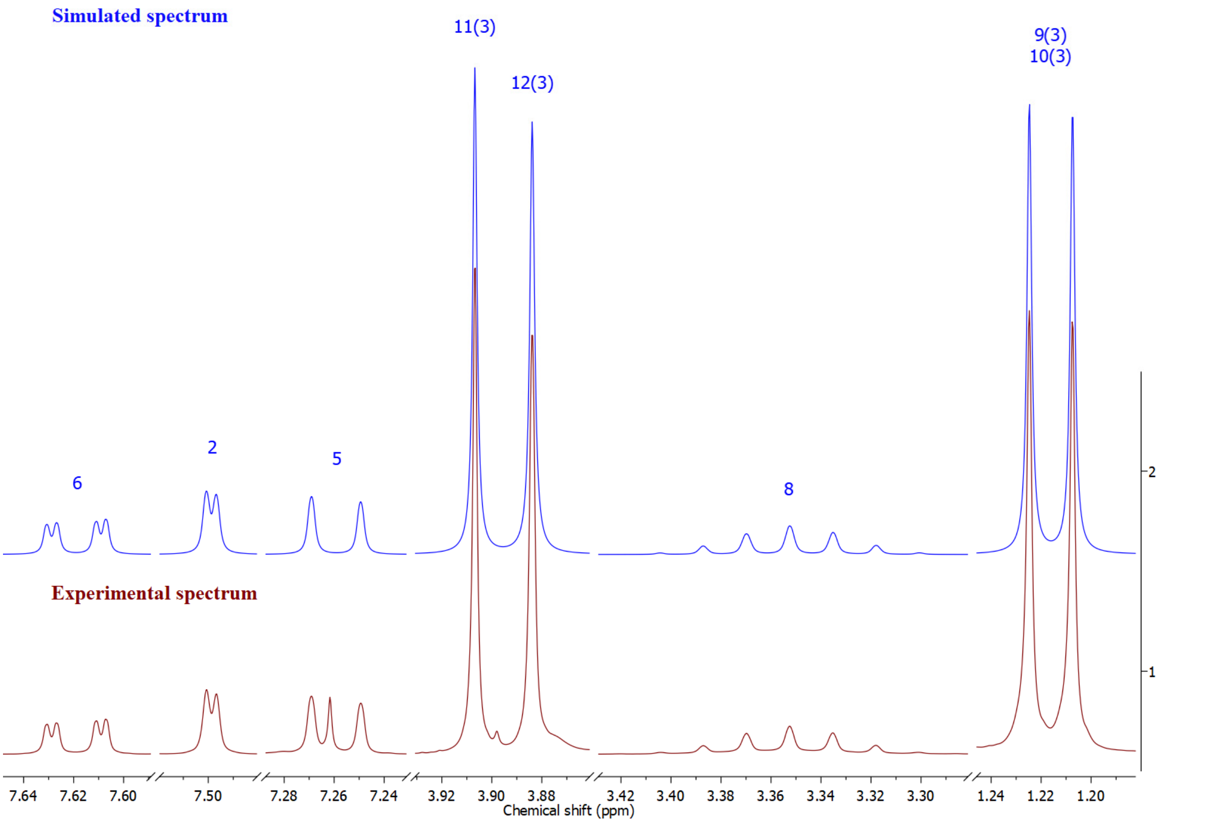

Figure S88. Simulated (manual iterative full spin, MestreNova 11.0.3) and experimental ^{1}H NMR spectrum of methyl 3-methoxycuminate (**23**)

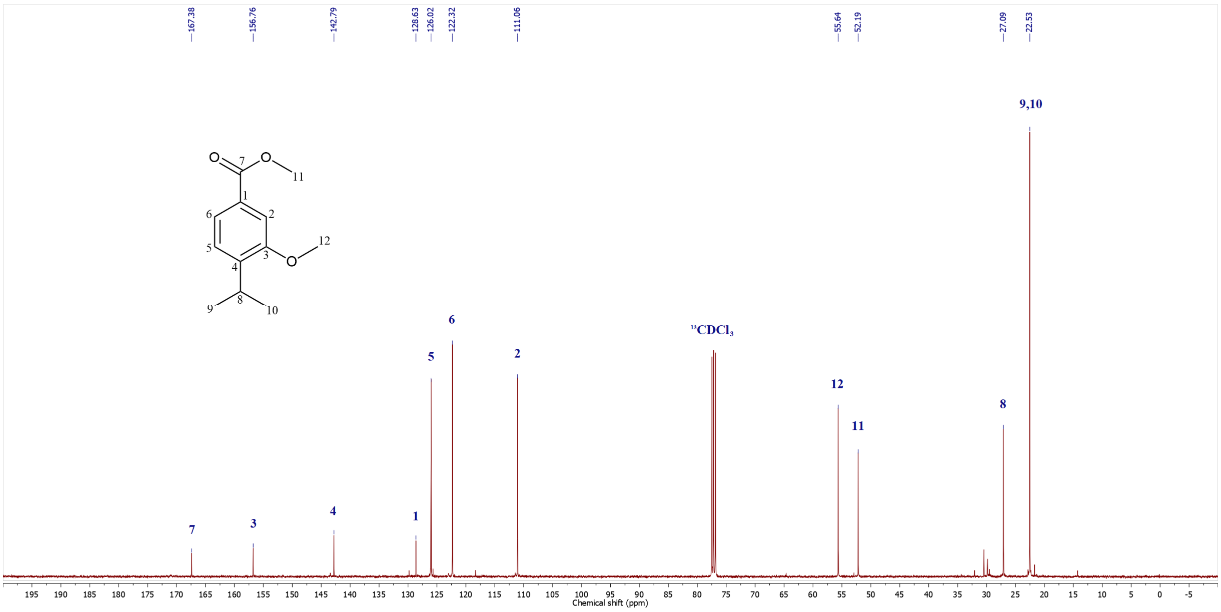

Figure S89. ^{13}C NMR (100.6 MHz, $CDCl_3$) spectrum of methyl 3-methoxycuminate (**23**)

Table S22. ^{1}H (400 MHz) and ^{13}C (100.6 MHz) NMR data of methyl 3-methoxycuminate (**23**) (chloroform-*d*), NMR parameters are derived from manual iterative full spin analysis, along with the observed gHMBC and NOESY correlations

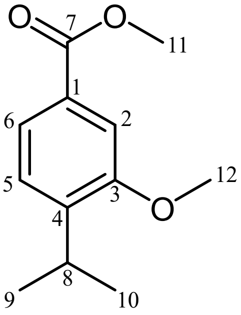

Position	δ_H (m, *J* (Hz), Integration)	δ_C	HMBC[a]	NOESY[b]
1	/	128.63	/	/
2	7.4988 (ddd, $^4J_{2,6}$ = 1.6, $^5J_{2,8}$ = 0.5, $^5J_{2,5}$ = 0.3, 1 H)	111.06	3,4,6,7	12
3	/	156.76	/	/
4	/	142.79	/	/
5	7.2595 (ddd, $^3J_{5,6}$ = 7.9, $^4J_{5,8}$ = -0.5, $^5J_{2,5}$ = 0.3, 1 H)	126.02	1,3,8	6,9,10
6	7.6186 (ddd, $^3J_{5,6}$ = 7.9, $^4J_{2,6}$ = 1.6, $^5J_{6,8}$ = 0.5, 1 H)	122.32	2,4,7	5,12
7	/	167.38	/	/
8	3.3523 (septddd, $^3J_{8,9/10}$ = 6.9, $^4J_{5,8}$ = -0.5, $^5J_{6,8}$ = 0.5, $^5J_{2,8}$ = 0.5, 1 H)	27.09	3,4,5,9,10	9,10
9 and 10	1.2161 (d, $^3J_{8,9/10}$ = 6.9, 6 H)	22.53	4,8,9,10	5,8
11	3.9067 (s, 3 H)	52.19	1	/
12	3.8837 (s, 3 H)	55.64	3	2,6

[a] gHMBC correlations observed between the hydrogen in this row and the carbon in the listed position.
[b] Cross-peaks observed in the NOESY spectrum.

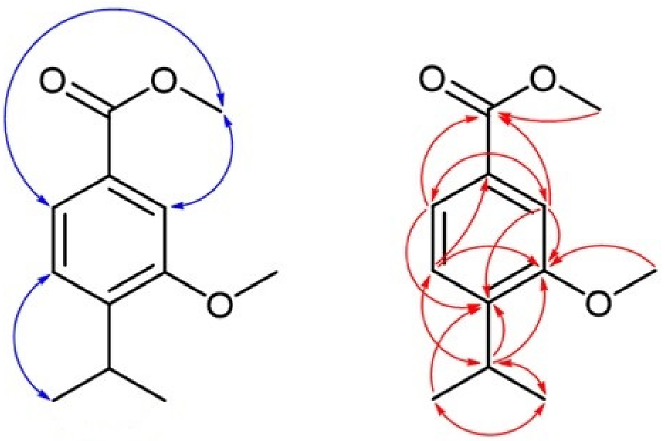

Figure S90. NOESY (blue arrows) and HMBC (red arrows) interactions methyl 3-methoxycuminate (**23**)

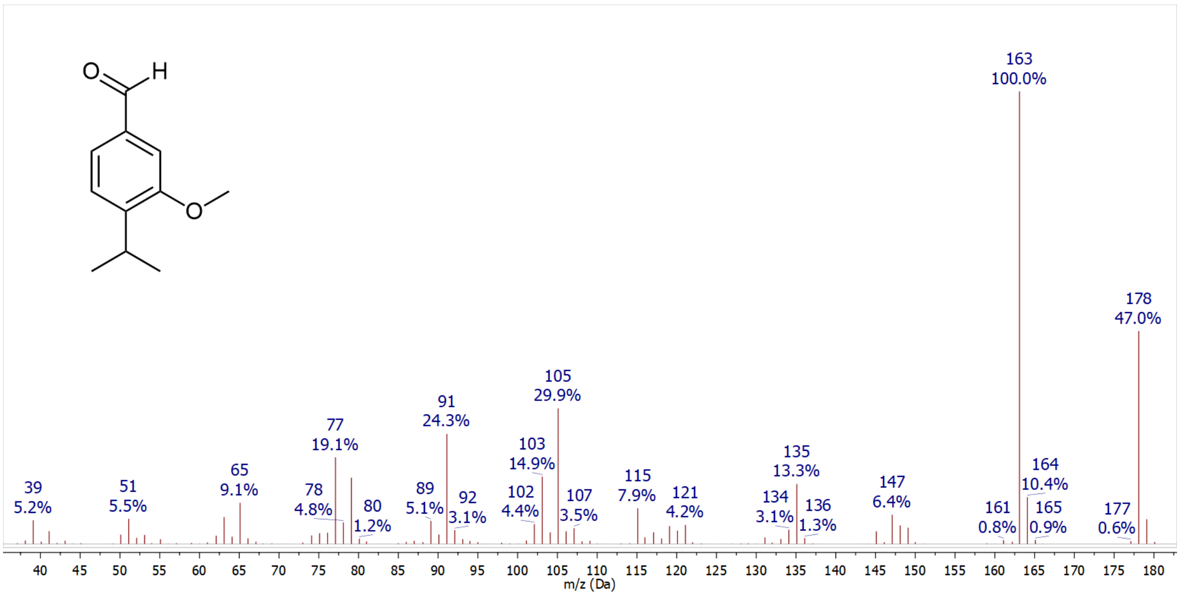

Figure S91. Mass spectrum (EI, 70 eV) of 3-methoxycuminaldehyde (**24**)

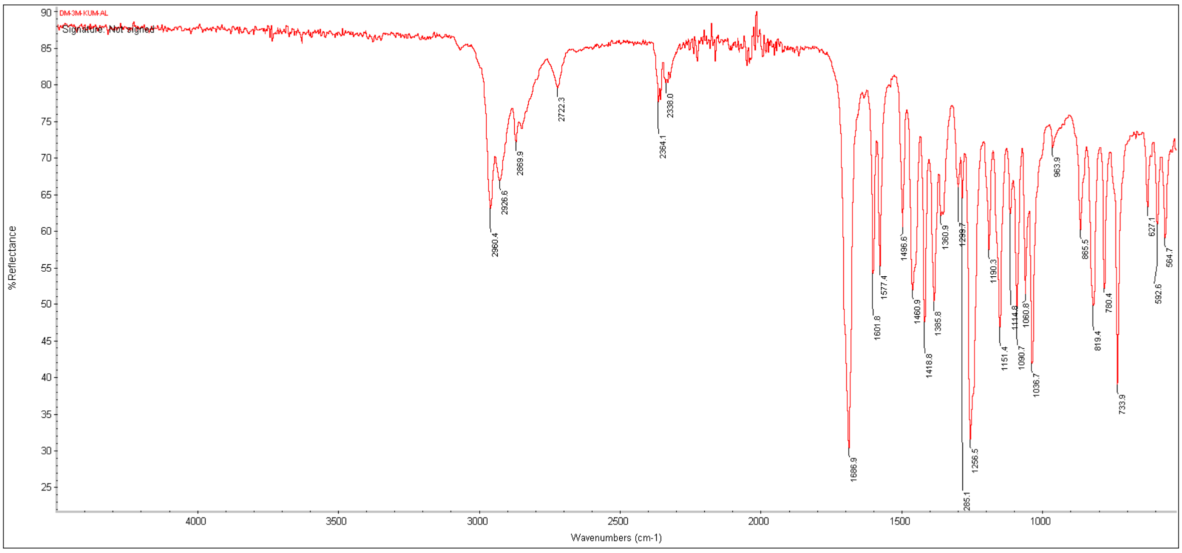

Figure S92. IR spectrum of 3-methoxycuminaldehyde (**24**)

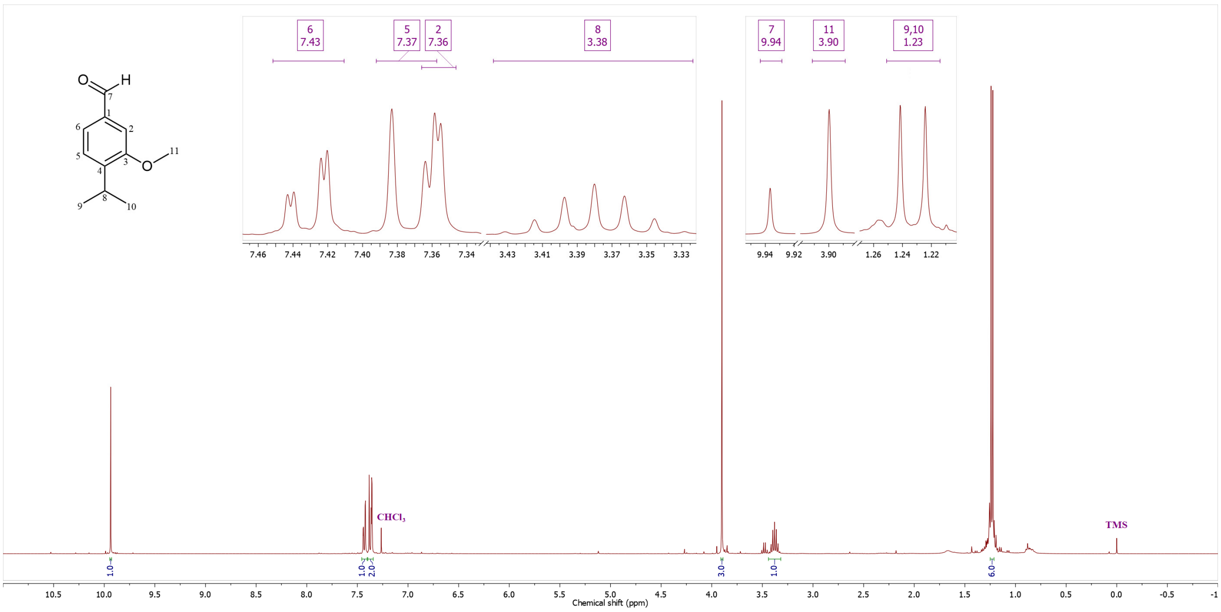

Figure S93. ^1H NMR (400 MHz, $CDCl_3$) spectrum of 3-methoxycuminaldehyde (**24**) and the corresponding expansions with signal assignment

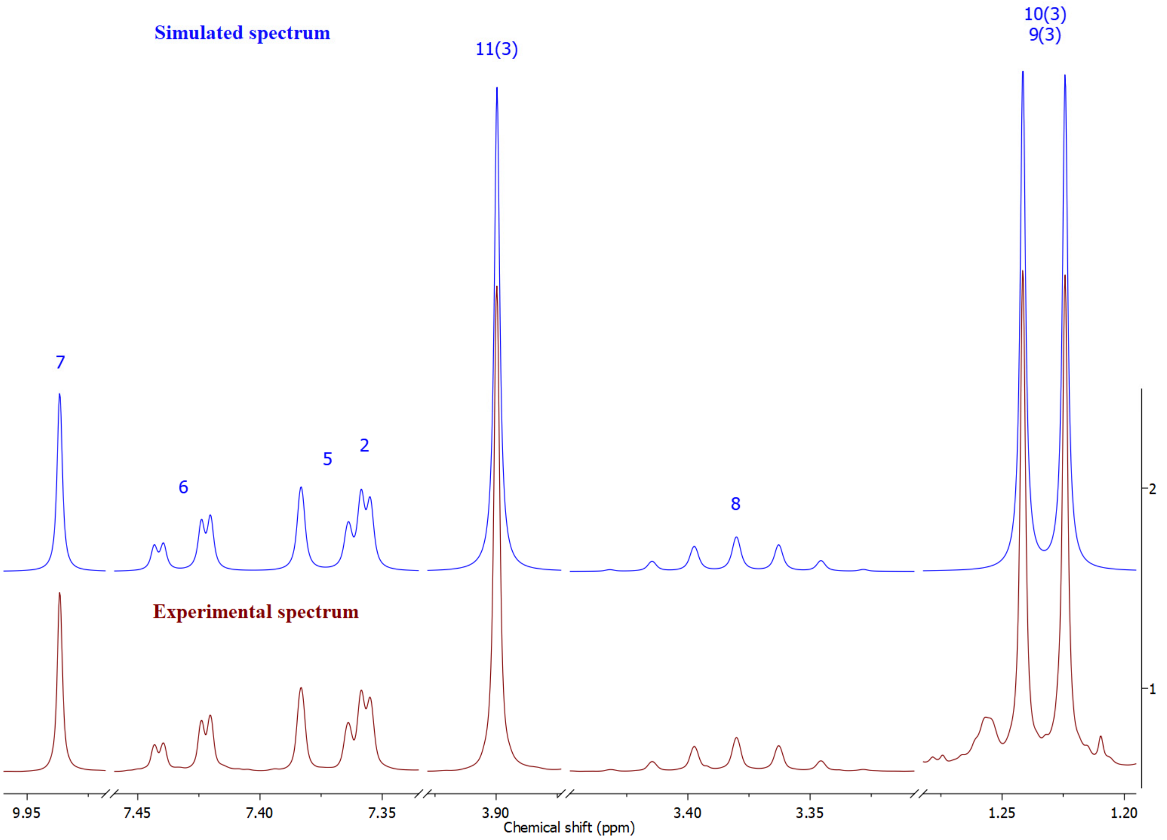

Figure S94. Simulated (manual iterative full spin, MestreNova 11.0.3) and experimental ^{1}H NMR spectrum of 3-methoxycuminaldehyde (**24**)

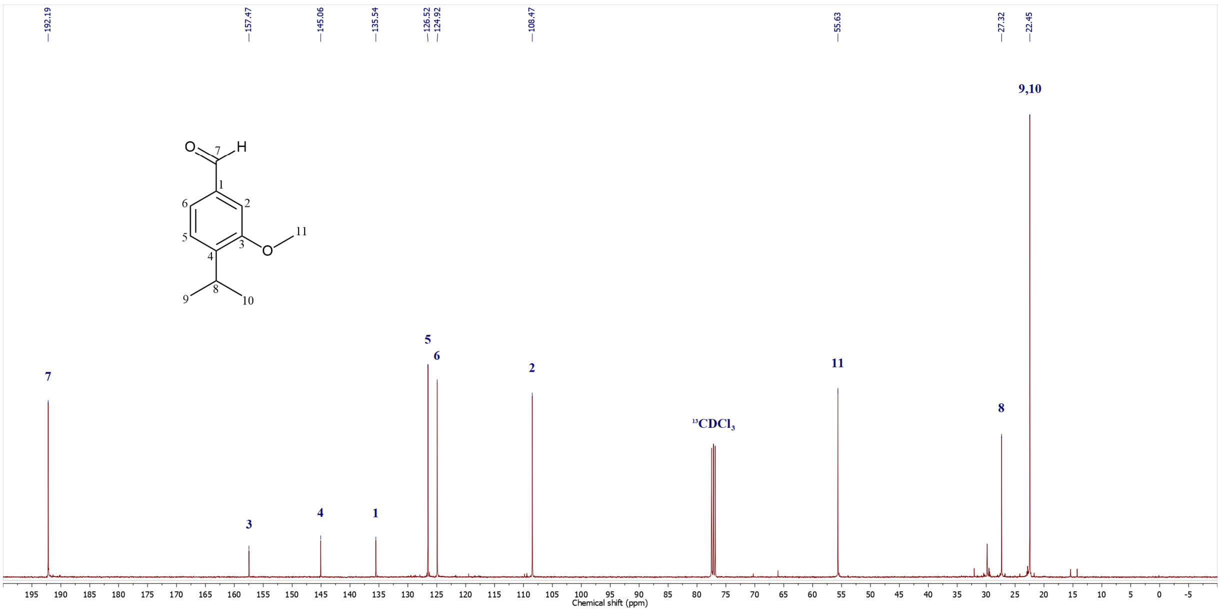

Figure S95. ^{13}C NMR (100.6 MHz, $CDCl_3$) spectrum of 3-methoxycuminaldehyde (**24**)

Table S23. ^{1}H (400 MHz) and ^{13}C (100.6 MHz) NMR data of 3-methoxycuminaldehyde (**24**) (chloroform-*d*), NMR parameters are derived from manual iterative full spin analysis, along with the observed gHMBC and NOESY correlations

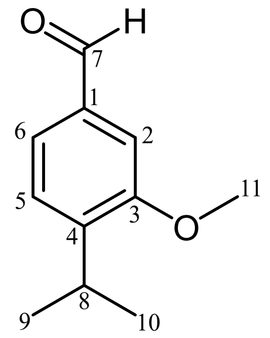

Position	δ_H (m, *J* (Hz), Integration)	δ_C	HMBC[a]	NOESY[b]
1	/	135.54	/	/
2	7.3568 (dddd, $^{4}J_{2,6}$ = 1.6, $^{5}J_{2,5}$ = 0.3, $^{4}J_{2,7}$ = -0.3, $^{5}J_{2,8}$ = 0.3, 1 H)	108.47	3,4,6,7	7,11
3	/	157.47	/	/
4	/	145.06	/	/
5	7.3752 (ddd, $^{3}J_{5,6}$ = 7.7, $^{4}J_{5,8}$ = -0.5, $^{5}J_{2,5}$ = 0.3, 1 H)	126.52	1,3,8	6,9,10
6	7.4301 (dddd, $^{3}J_{5,6}$ = 7.7, $^{4}J_{2,6}$ = 1.6, $^{4}J_{6,7}$ = -0.3, $^{5}J_{6,8}$ = 0.3, 1 H)	124.92	2,4,7	5,7
7	9.9366 (dd, $^{4}J_{2,7}$ = -0.3, $^{4}J_{6,7}$ = -0.3, 1 H)	192.19	1,2,6	2,6
8	3.3799 (septddd, $^{3}J_{8,9/10}$ = 6.9, $^{4}J_{5,8}$ = -0.5, $^{5}J_{2,8}$ = 0.3, $^{5}J_{6,8}$ = 0.3, 1 H)	27.32	3,4,5,9,10	9,10
9 and 10	1.2329 (d, $^{3}J_{8,9/10}$ = 6.9, 6 H)	22.45	4,8,9,10	5,8
11	3.8997 (s, 3 H)	55.63	3	2

[a] gHMBC correlations observed between the hydrogen in this row and the carbon in the listed position.
[b] Cross-peaks observed in the NOESY spectrum.

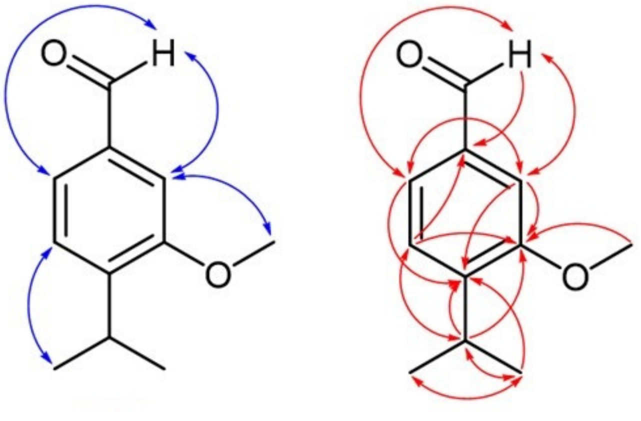

Figure S96. NOESY (blue arrows) and HMBC (red arrows) interactions 3-methoxycuminaldehyde (**24**)
